# Supplementary material for: Investigation of the demand for a 7-day (extended access) primary care service: an observational study from pilot schemes in England
Source: BMJ Open. 2019 Sep 5;9(9):e028138. doi: 10.1136/bmjopen-2018-028138 (PMC6731947; doi:10.1136/bmjopen-2018-028138)
Supplement: Supplementary data [file bmjopen-2018-028138supp001.pdf]

Supplementary Table S1 Summary of 7 Day Access Implementation by Area

Clinical Commissioning Group	Live date (2016)	Service	Self-referral	Monday-Friday opening	Saturday opening	Sunday opening
**CCG1**						
**Hub 1**	2 April	GP and nurse	No	No	09:00-13:00	10:00-13:00
**Hub 2**	2 April	GP and nurse	No	No	09:00-13:00	10:00-13:00
**CCG2**						
**Hub 1**	11 January	GP and nurse	Yes	18:30-21:00	08:00-18:00	10:00-13:00
**Hub 2**	1 January	GP and nurse	Yes	18:30-21:00	08:00-18:00	10:00-13:00
**Hub 3**	2 January	GP and nurse	Yes	18:30-21:00	08:00-18:00	10:00-13:00
**Hub 4**	2 January	GP and nurse	Yes	18:30-21:00	08:00-18:00	10:00-13:00
**CCG3**						
**Hub 1**	5 January	GP only	Yes	18:30-20:00	10:00-14:00	10:00-14:00
**Hub 2**	10 August	GP only	Yes	18:30-20:00	10:00-14:00	No
**CCG4**						
**Hub 1**	3 January	GP only	No	18:30-20:00	09:00-11:45	09:00-12:00
**Hub 2**	30 January	GP only	No	18:15-20:00 (no Thursday or Friday)	09:00-11:45	09:00-11:45
**Hub 3**	25 February	GP only	No	18:30-20:00	09:00-11:45	09:00-12:00
**CCG5**						
**Hub 1**	16 January	GP and nurse	No	No	09:00-13:00	No
**Hub 2**	16 January	GP and nurse	No	No	09:00-13:00	No
