# Supplementary material for: Investigation of the demand for a 7-day (extended access) primary care service: an observational study from pilot schemes in England
Source: BMJ Open. 2019 Sep 5;9(9):e028138. doi: 10.1136/bmjopen-2018-028138 (PMC6731947; doi:10.1136/bmjopen-2018-028138)
Supplement: Supplementary data [file bmjopen-2018-028138supp002.pdf]

Supplementary Table S2 Activity Capture Minimum Dataset

Data requested from Clinical Commissioning Group	Format
Volume of appointments available	
Date	DD/MM/YYYY
Time of appointment	HH:MM
Discipline	GP/Nurse
Planned/pre-booked vs same day/urgent	Pre-booked/Same day
Method of appointment (face-to-face/phone)	Face-to-face/phone
Volume of appointments booked and booked and attended	
Date	DD/MM/YYYY
Time of appointment	HH:MM
Discipline	GP/Nurse
Age	10 year age bands: 0-9 to 90+
Gender	Male/Female
Registered GP practice	Practice code identifier
Postcode (first part)	First half
Planned/pre-booked vs same day/urgent	Pre-booked/Same day
Method of appointment (face-to-face/phone)	Face-to-face/phone
Method of appointment (face-to-face/phone)	Face-to-face/phone
