# Supplementary material for: Investigation of the demand for a 7-day (extended access) primary care service: an observational study from pilot schemes in England
Source: BMJ Open. 2019 Sep 5;9(9):e028138. doi: 10.1136/bmjopen-2018-028138 (PMC6731947; doi:10.1136/bmjopen-2018-028138)
Supplement: Supplementary data [file bmjopen-2018-028138supp011.pdf]

GM PRIMARY CARE 7 DAY ACCESS EVALUATION: RESEARCH PROTOCOL

Background

In June 2014, the 12 Greater Manchester Clinical Commissioning Groups (CCGs) agreed to the Healthier Together Primary Care Standard that by the end of 2015, “everyone in Greater Manchester who needs medical help will have same-day access to primary care services, supported by diagnostic tests, seven days a week”[1]. At the same time seven day access has been made one of eight early implementation priorities under the Greater Manchester devolution agreement[2].

This protocol describes the proposed evaluation of seven day access to primary care to be conducted on behalf of NHS England (Greater Manchester) by CLAHRC Greater Manchester. CLAHRC GM previously conducted the evaluation of the NHS England Primary Care Demonstrator services, and the proposed evaluation seeks to build on the strengths of the previous evaluation. The proposed mixed methods evaluation will combine a focus on **activity**, **process** and **outcomes** in order to provide a comprehensive assessment of the implementation and impact of the new services. The evaluation will focus primarily on the 7 CCG areas receiving support from NHS England as part of the devolution agreement. The remaining 5 CCG areas have pre-existing funding and support to provide 7 day access via either the Wave 1 Prime Minister’s Challenge Fund (PMCF) or Wave 2 General Practice Access Fund (GPAF) (see **Table 1**). The majority of these are already ‘live’; therefore while they will not make up part of the formal evaluation, intelligence gathered in the PMCF/GPAF areas can be used to support learning and development in the seven areas where 7-day primary care access is now being introduced.

Table 1: 7-Day Access Areas in Greater Manchester

PMCF/GPAF Areas (not evaluated)	New 7-Day Access Areas (to be evaluated)
Bury	Bolton
Wigan	Heywood, Middleton and Rochdale
North Manchester	Oldham
Central Manchester	Salford
South Manchester	Stockport
	Trafford
	Tameside & Glossop

[1] https://healthiertogethergm.nhs.uk/what-healthier-together/primary-care/
[2] http://www.gmhealthandsocialcaredevo.org.uk/assets/GM-Strategic-Plan-Final.pdf p14

The 7 CCGs submitted their initial plans for the delivery of seven day primary care access to NHS England in mid-2015. The schemes were expected to start in December 2015, although some have indicated a later start date. All areas are adopting a 'hub' approach to providing additional hours, in which the CCG area is split into geographic areas, and the extended access services are provided from a 'hub' location in each area. CCG plans submitted to NHS England promise 43 hubs (including PMCF/GPAF hubs) operational by December 2015 with a further 9 hubs live in 2016 (see figure 1).

Figure 1

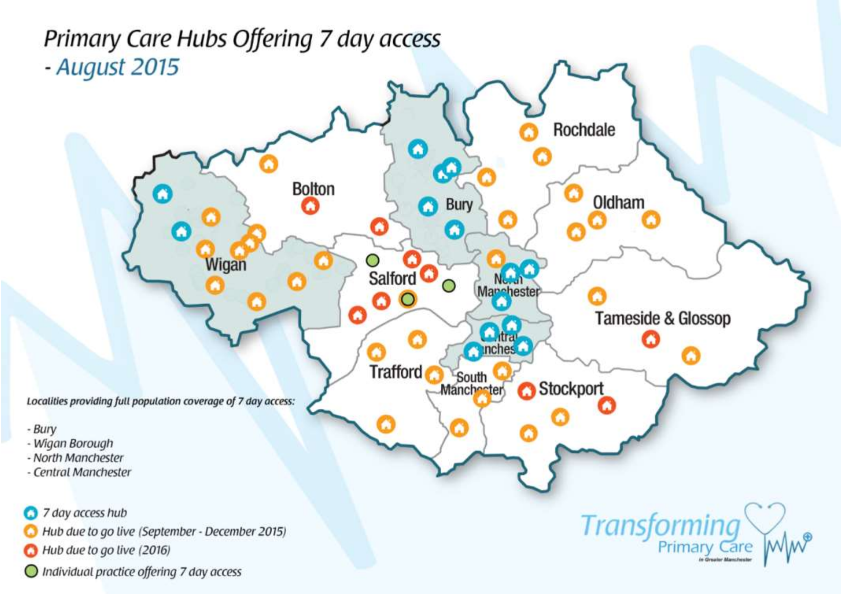

Description of Evaluation

The evaluation seeks to understand the implementation and impact of seven day primary care services across Greater Manchester. The evaluation will combine qualitative and quantitative analysis, supported by a measure of activity across the 7 areas to be evaluated.

The evaluation will seek to answer the following questions (see **Table 2**);

Table 2: Evaluation Questions and Modes of Evaluation

1. What new activity is associated with seven day primary care services in each area, and at what cost?	**ACTIVITY**
2. How are these new services utilised by patients? What is the nature of demand for these new services?	
3. How is the uptake of 7 day primary care affected by the socio-economic characteristics of the patient population?	
4. What organisational and operational issues arise in the implementation of 7 day access to primary care, and how have areas addressed these issues?	**PROCESS**

5. What is the impact of 7 day access to primary care on;<br>a. Identified prevalence of disease,<br>b. Attendances/admissions at Accident & Emergency,<br>c. Use of Out-of-Hours services?	**OUTCOME**
6. How has the change in access been received by<br>a. Patients,<br>b. Staff in primary care?	

As is indicated above, questions 1-3 relate to the **activity** analysis (i.e. what was done). Question 4 relates to the **process** analysis (i.e. how was it done). Questions 5 and 6 relate to the **outcome** analysis (i.e. what was the impact of the new activity).

It should be emphasised that the ability to answer these questions depends directly on the availability of relevant data. It is important that data can be captured by the evaluation team in a timely manner, can be presented in a format which allows for analysis, and that this can be done subject to current information governance regulation. Some data is routinely collected, other data can be collected by the evaluation team with the facilitation of the commissioners and providers in each area, and some can only be produced by the providers themselves (see **Table 3**);

Table 3: Evaluation Questions and Data Sources

1. What new activity is associated with seven day primary care services in each area, and at what cost?	**Activity data supplied by each area**
2. How are these new services utilised by patients? What is the nature of demand for these new services?	**Activity data supplied by each area**
3. How is the uptake of 7 day primary care affected by the socio-economic characteristics of the patient population?	**Public data combined with activity data.**
4. What organisational and operational issues arise in the implementation of 7 day access to primary care, and how have areas addressed these issues?	**Evaluation generated data. Source: qualitative interviews.**
5. What is the impact of 7 day access to primary care on;<br>a. Identified prevalence of disease,<br>b. Attendances/admissions at Accident & Emergency,<br>c. Use of Out-of-Hours services?	**Routinely collected data. Source:<br>(a) QOF, via CCGs,<br>(b) SUS, via AHSN,<br>(c) OOH activity, supplied by NHS England/CCGs.**
6. How has the change in access been received by<br>a. Patients,<br>b. Staff in primary care?	**Evaluation generated data. Source:<br>(a) GPPS, via HSCIC.<br>(b) staff survey administered by evaluation team via CCGs.**

The evaluation period will begin at the point at which the NHS England hubs go live on 1st January 2016 and continue for 12 months.

The evaluation has conducted preliminary work to establish reliable sources of data, in discussion with NHS England (Greater Manchester) and the Greater Manchester Academic Health Science Network (AHSN). The provision of activity data from each area has not yet been agreed: - this research protocol document is intended to provide information and thereby initiate the process of agreeing the content and process of activity capture in dialogue with each area.

Further details on the data capture and analysis is provided below.

1. Activity Capture

The activity capture in the present evaluation has been designed in order to minimise the additional workload that data extraction places on individual sites, while also seeking to ensure that the data collected can be collected reliably and consistently across all 7 areas.

To this end, a minimum data set (**Table 4** below) has been defined, to be agreed with all participating CCGs. Data should be reported according to a monthly schedule, which will support formative evaluation and shared learning across sites. Also data will be needed from NHS England/CCGs on relevant DES arrangements in each area, to establish activity baselines.

Table 4: Activity Capture Minimum Dataset

**1. Utilisation:**
a. No. of appointments available
i. By day
1. Time of appointment
ii. By discipline
iii. Planned/pre-booked vs same day/urgent
iv. Method of appointment (face-to-face/phone)
b. No. of appointments booked
i. By day
1. Time of appointment
ii. By discipline
iii. Age
iv. Gender
v. Registered GP practice
vi. Postcode
vii. Planned/pre-booked vs same day/urgent
viii. Method of appointment (face-to-face/phone)
c. No. of appointments used (Utilisation)
i. By day
1. Time of appointment
ii. By discipline
iii. Age
iv. Gender
v. Registered GP practice
vi. Postcode (first part)
vii. Planned/pre-booked vs same day/urgent
viii. Method of appointment (face-to-face/phone)

**2. Practice-level population data:**	
a.	Practice level population
b.	Practice codes
c.	Whether a GP practice or not
d.	Registered patient age band
e.	Deprivation of practice population
f.	Gender breakdown of practice population

The aim of the activity capture evaluation is to:

1. **Assess the ability of each area to provide additional appointments i.e.**
    a. **How many additional appointments were available, by whom and by what means?**
    b. **How many additional appointments were booked, urgent, and how many resulted in DNA?**
2. **Assess the types of patients utilizing additional appointments i.e.**
    a. **Were patients attending/DNA representative of the practice's total list (in terms of age, gender, and IMD)?**

For utilisation data (Items 1a-1c, Table 3), the evaluation relies on the ability of each area to collect, collate and provide data routinely. Preliminary conversations with NHS England and some local CCGs suggest that this data should be available – however, this requires confirmation with all areas.[3]

CCG level population data (Item 2, Table 3) can be extracted by the CLAHRC evaluation team from individual practice registration data.

2. Process Evaluation

Semi-structured interviews will be conducted with senior leadership (CCG lead + GP lead in each area). 2 leads x 7 areas= 14 interviews, conducted at start and end of intervention period = 28 interviews. These will be supplemented with key informant interviews (estimated 6-7), including leads in the PMCF/GPAF areas, and, where appropriate, additional interviews with key individuals in each area.

The process evaluation will seek to understand the organisational and operational issues in the implementation of 7 day access to primary care faced in each area, and also how each area addressed these issues. A brief interview schedule can be found in **Appendix 1**.

[3] In addition, the following data would enhance the analysis by providing insight into the reasons for and nature of the additional consultations.

1. Reason for attendance
2. Outcome of consultation
    a. Discharged with advice
    b. Referral to ….. (list providers)
    c. Treatment/prescription issued
    d. Follow-up appointment
    e. Diagnostics
    f. Other

However, we recognise this may be difficult or impossible to provide without incurring substantial and unrealistic data collection costs on the areas.

3. Outcome Evaluation

The outcome evaluation will concentrate on four areas; (A) Health Outcomes, (B) Impact on Service Utilisation, (C) Staff Experience and (D) Patient Experience.

A. **Impact on Health Outcomes** will be primarily measured using selected QOF data on disease prevalence. QOF data on disease prevalence will be analysed to examine the effect of the intervention on the identification and management of long-term conditions. This data will be supplied by local CCGs. Data will be captured for three years prior to the intervention (2013-16) and at two points during the intervention itself. A full list of QOF data categories can be found in **Appendix 2**.

B. **Impact on Service Utilisation** will be measured using routinely-collected SUS data on hospital activity, plus activity data from relevant out-of-hours providers supplied by NHS England/CCGs.

SUS data will be extracted for all areas of Greater Manchester (seven 7 day access sites and 5 PMCF/GPAF areas) on a quarterly basis. The specific items to be analysed can be found in **Appendix 3.**

The aim of the service utilisation evaluation is to:

(1). Assess the effects of additional appointments on hospital activity:

a) A&E attendances:

i) Minor patient-initiated A&E attendances

ii) Total A&E attendances

iii) Waiting time to assessment, treatment, and total A&E duration

b) Admissions:

i) Total admissions

ii) Admissions for ambulatory care-sensitive conditions

(2). Assess whether (1) varies by dosage/volume of additional appointments per 1000

(3). Assess whether (1) varies by additional appointments' users characteristics (age, gender, deprivation)

(1) will be measured using SUS data for the intervention period (quarters 1 to 4 of 2016) compared to 2015 i.e. a before and after analysis. All activity will be measured at the practice level, by quarter, and per 1000 of a practices registered population. A pooled cross-sectional dataset will be used. For each type of hospital activity the dependent variable will be regressed on binary year dummies. The estimated coefficient on the intervention (2016) dummy provide a statistical test for significant differences in the intervention period. Count models will be estimated that recognise and incorporate the over-dispersed distribution of hospital activity. Sensitivity analysis using alternative model specifications will be carried out.

The diagnosis code (DIAG_NN) in the admissions data will be used to identify ambulatory care sensitive conditions as follows:

- Ambulatory care-sensitive conditions admissions (ICD)
- Vaccine-preventable:
  Influenza and pneumonia; Other vaccine-preventable conditions
- Chronic:
  Asthma; Congestive heart failure; Diabetes complications; Chronic obstructive pulmonary disease (COPD); Angina; Iron-deficiency anaemia; Hypertension; Nutritional deficiencies

- Acute:

  Dehydration and gastroenteritis; Pyelonephritis; Perforated/bleeding ulcer; Cellulitis; Pelvic inflammatory disease; Ear, nose and throat infections; Dental conditions; Convulsions and epilepsy; Gangrene

Where available, the difference in hospital activity will be compared against comparator practices and tests for significant differences measured using a *controlled* before and after analysis framework (difference-in-differences).

Items (2) and (3) depend on the quality and coverage of the activity capture. Using data from the activity capture we will generate a measure of intensity of treatment (dosage - additional appointments per 1000 of a practice's registered population) and replicate (1) by dosage. Information on the types of patients using additional appointments from the activity capture will be used to identify the groups where hospital activity may be most concentrated. For example, if additional appointments are concentrated amongst females then it may be that female hospital activity is where the impacts of additional appointments may be seen.

C. **Staff Experience** The evaluation team will develop an online survey to capture information on the experience of primary care staff. This administration of this survey will require the cooperation of local provider organisations.
D. **Patient Experience** We will analyse General Practice Patient Survey (GPPS) data, adopting a difference-in-difference (DiD) methodology, focusing on survey items addressing overall quality of care and convenience, subject to data availability (see note below). Individual CCGs may choose to conduct their own patient surveys, and guidance can be provided by the evaluation team on the administration of these.[4]

[4] Note: While the relevant period of GPPS data will be July-September 2016, which should be released in early 2017. If the release of this data is delayed by HSCIC, to avoid delaying report submission at end of March 2017 then patient experience analysis may have to be supplied latter as a separate addendum to the report.

Evaluation Timeline

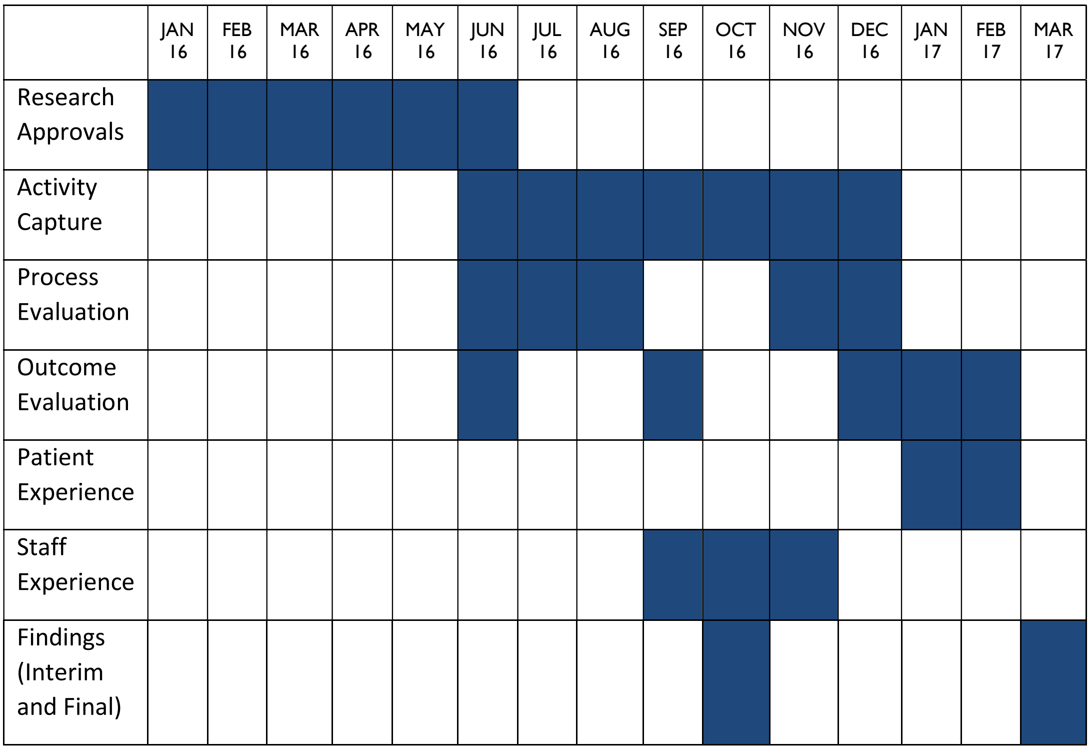

This timeline assumes that;

- The minimum activity dataset can be provided on a monthly basis by CCGs.
- Information on relevant DES activity can be supplied by NHS England/CCGs.
- The evaluation team has access to key individuals in each area for interviews.
- CCGs are able to provide selected QOF data on disease prevalence.
- The AHSN is able to supply regular extracts of SUS data in the appropriate format.
- NHSE is able to supply data on OOH activity in the appropriate format.
- CCGs are able to assist with the dissemination of an online staff survey.
- GP Patient Survey results are released by HSCIC in a timely manner.

Appendix 1

Interview Schedule

1. Describe the primary care access arrangements in your area outside standard hours prior to this initiative, and describe the 7-day access arrangements in place now.
2. What is your role in delivering this change?
3. What is/was required to establish 7-day access to primary care in your locality?
4. What steps have been taken so far?
5. How have you communicated the changes to patients?
6. What challenges have been encountered (IT, IG, communications and engagement, workforce, finance, infrastructure)?
7. How have you tackled these challenges?
8. What do you expect will be the impact of this change in access (on patients, staff and other parts of the health and social care system)?
9. How would you measure 'success' in this change?
10. How sustainable are the changes made in your area?

Appendix 2

AST001. The contractor establishes and maintains a register of patients with asthma, excluding patients with asthma who have been prescribed no asthma-related drugs in the preceding 12 months.

AF001. The contractor establishes and maintains a register of patients with atrial fibrillation.

CAN001. The contractor establishes and maintains a register of all cancer patients defined as a "register of patients with a diagnosis of cancer excluding non-melanotic skin cancers diagnosed on or after 1 April 2003".

CKD001. The contractor establishes and maintains a register of patients aged 18 or over with CKD (US National Kidney Foundation: Stage 3 to 5 CKD).

COPD001. The contractor establishes and maintains a register of patients with COPD.

DEM001. The contractor establishes and maintains a register of patients diagnosed with dementia.

DEP003. The percentage of patients aged 18 or over with a new diagnosis of depression in the preceding 1 April to 31 March, who have been reviewed not earlier than 10 days after and not later than 56 days after the date of diagnosis.

DM017. The contractor establishes and maintains a register of all patients aged 17 or over with diabetes mellitus, which specifies the type of diabetes where a diagnosis has been confirmed.

EP001. The contractor establishes and maintains a register of patients aged 18 or over receiving drug treatment for epilepsy.

HF001. The contractor establishes and maintains a register of patients with heart failure.

HYP001. The contractor establishes and maintains a register of patients with established hypertension.

LD003. The contractor establishes and maintains a register of patients with learning disabilities.

MH001. The contractor establishes and maintains a register of patients with schizophrenia, bipolar affective disorder and other psychoses and other patients on lithium therapy.

OST004. The contractor establishes and maintains a register of patients: 1. Aged 50 or over and who have not attained the age of 75 with a record of a fragility fracture on or after 1 April 2012 and a diagnosis of osteoporosis confirmed on DXA scan, and 2. Aged 75 or over with a record of a fragility fracture on or after 1 April 2014 and a diagnosis of osteoporosis.

PC001. The contractor establishes and maintains a register of all patients in need of palliative care/support irrespective of age.

PAD001. The contractor establishes and maintains a register of patients with peripheral arterial disease.

RA001. The contractor establishes and maintains a register of patients aged 16 or over with rheumatoid arthritis.

CHD001. The contractor establishes and maintains a register of patients with coronary heart disease.

STIA001. The contractor establishes and maintains a register of patients with stroke or TIA.

Appendix 3

Relevant SUS data to be analysed

Derived variable	SUS variable name	SUS variable code
**A&E DATA**		
**KEY BASE DATA**		
**A&E department type**	A&E department type	AEDEPTTYPE
**Provider code**	Provider code	PROCODET
**Arrival date**	A&E arrival date	ARRIVALDATE
**Arrival time**	A&E arrival time	ARRIVALTIME
**Arrival mode**	Arrival mode	AEARRIVALMODE
**Where incident took place**	Incident location type	AEINCLOCTYPE
**GP practice**	Code of GP practice	GPPRAC
**CCG of GP practice**	CCG of patient's GP practice	CCG_GP_PRACTICE
**CCG of Treatment**	CCG of Treatment	CCG_TREATMENT
**Self-referrals for minor conditions**	Referral mode,<br>SUS PbR derived healthcare resource group code	AEREFSOURCE<br>SUSHRG
**Cost**	Trust derived HRG value	HRGNHS
**GP referrals**	Referral mode	AEREFSOURCE
**Arrival age**	Age on arrival	ARRIVALAGE
**Sex**	Sex of patient	SEX
**Ethnicity**	Ethnic category	ETHNOS
**Deprivation**	Lower Super Output Area (to obtain IMD)	LSOA11
**Diagnosis**	A&E diagnosis	DIAG_NN
**EFFECT ON ADMISSIONS/WORKLOAD**		
**Admissions from A&E**	Attendance disposal	AEATTENDISP
**A&E Waiting times**		
**A&E time to assessment (waiting time)**	A&E Duration to assessment	INITDUR
**A&E time to treatment (waiting time)**	A&E Duration to treatment	TRETDUR
**A&E total duration**	A&E duration to departure	DEPDUR
**ADMISSIONS DATA**		
**KEY BASE DATA**		
**Provider code**	Provider code	PROCODET
**Arrival date**	Date of admission	ADMIDATE
**Discharge date**	Date of discharge	DISDATE
**GP practice**	Code of GP practice	GPPRAC
**CCG of GP practice**	CCG of patient's GP practice	CCG_GP_PRACTICE
**CCG of Treatment**	CCG of Treatment	CCG_TREATMENT
**Cost**	Trust derived HRG value	HRGNHS
**Arrival age**	Age on arrival	ADMIAGE
**Sex**	Sex of patient	SEX
**Ethnicity**	Ethnic category	ETHNOS
**Deprivation**	Lower Super Output Area (to obtain IMD)	LSOA11
**Ambulatory care-sensitive conditions admissions (ICD)**	All Diagnosis codes	DIAG_NN
