# Supplementary material for: Investigation of the demand for a 7-day (extended access) primary care service: an observational study from pilot schemes in England
Source: BMJ Open. 2019 Sep 5;9(9):e028138. doi: 10.1136/bmjopen-2018-028138 (PMC6731947; doi:10.1136/bmjopen-2018-028138)
Supplement: Supplementary data [file bmjopen-2018-028138supp012.pdf]

Supplementary Text S2

(i) Uptake

Patients could book an appointment which could then be attended or not. Focussing on booked appointments alone would give an indication of the demand for the service, whilst assessments of appointments actually used shed light on realised demand and is more insightful for evaluating actual impacts on service use. We estimated two separate sets of probability models:

1) the probability an appointment was booked, and
2) the probability an appointment was booked and attended ('used')

We assessed differences in the rate of uptake by estimating probability models (probit regressions) of appointment use (y_{ij}):

$$y_{ij} = \beta_1 D_{ij} + \beta_2 M_{ij} + \beta_3 CCG_j + e_{ij} \quad [1]$$

where the unit of analysis is available appointment i in CCG j.

Regression models were estimated to enable adjustment for CCG effects which may be important given the variation in schemes across CCGs (Supplementary Table 1), Probit models were used to estimate differences in the probability (rate) of uptake across categories of the predictor variables. The estimates give absolute percentage point differences across predictor variables which is more informative than relative odds-ratios for understanding the scale of uptake.

To assess whether uptake of the service varied across day of the week we included day of week dummies in the regression (D_i), with the estimates ($\hat{\beta}_1$) providing the differences in the rate of uptake relative to the base week day (Monday); this enables a test for whether uptake is lower on particular days. To assess whether there is evidence of assimilation to the service we include calendar month dummies (M_i). If assimilation is evident the estimates on the calendar month dummies should be monotonically increasing over the period. To account for potential confounding caused by variations in the provision and delivery of services across schemes we include CCG dummies (fixed-effects) in the regression (CCG_j). Standard errors are clustered at CCG level.

Estimates are presented as average marginal effects (using Stata's margins command), which give the average percentage point effect of the variable relative to the base category across the sample.

(ii) Analysis of demographics of patients booking an extended access appointment

Our second set of analyses concentrated on booked appointments and exploited variations in service delivery to test whether there were associations in the demand for booking particular types of services (booking type (pre-booked or same day) and appointment type (GP or nurse)) and for whether an appointment was used or DNA; with day of week, calendar month, the patient booking the appointments age and gender, and CCG. We estimated three separate sets of probability models:

1) The probability an appointment was pre-booked versus same day;
2) The probability an appointment was for a GP versus for a nurse practitioner;
3) The probability an appointment was used or not attended (DNA)

We assessed differences in the rate of appointment type by estimating probability models (probit regressions) of appointment type (y_{ij}):

$$y_{ij} = \beta_1 D_{ij} + \beta_2 M_{ij} + \beta_3 DNA_{ij} + \beta_4 Female_{ij} + \beta_5 Age_{ij} + \beta_6 CCG_j + e_{ij} \qquad [2]$$

where the unit of analysis is the type of booked appointment i in CCG j. $Female_{ij}$ is a dummy variable indicating the gender of the patient booked for appointment i in CCG j, and Age_{ij} is a set of 10 age-band dummies indicating the age of the patient. For all models estimated, estimates are presented as average marginal effects and standard errors are clustered at GP practice level where the practice is the patient who has booked the appointments registered practice. Schemes that exclusively provided a certain service (e.g. only GP appointments) are excluded from the relevant analysis since uptake of the alternative is necessarily zero.
