# Supplementary material for: Investigation of the demand for a 7-day (extended access) primary care service: an observational study from pilot schemes in England
Source: BMJ Open. 2019 Sep 5;9(9):e028138. doi: 10.1136/bmjopen-2018-028138 (PMC6731947; doi:10.1136/bmjopen-2018-028138)
Supplement: Supplementary data [file bmjopen-2018-028138supp013.pdf]

Supplementary Text S3

CLAHRC Patient Panel Protocol Review
Collated Feedback and Responses

**Feedback from panel**	**Project team response**
The interview document [referring to the interview schedule] seems ok.	No response required.
The survey document I would change slightly as per the attachment:<br><br>5. Which of the following do you feel (may have-deleted) has benefited from the implementation of seven day access to primary care?<br><br>6. Remove mentions of 'efforts to' from the response options e.g. efforts to reduce demand on A&E services.	
This is a very comprehensive, well thought out document. It is a very comprehensive protocol indeed.<br><br>Appointments must be booked in advance, but what if changes take place on the part of either the patient or the GP hub nearer the time of the appointment?<br><br>Evidence of use and pattern of use at weekends is crucial! Evenings will prove popular, Saturday mornings will indicate some demand but I am unsure about the demand on Saturday afternoons and especially Sundays. I am referring to planned appointments and not Emergency appointments!	No response required.<br><br>No change to protocol as this situation is not specific to the additional availability, as appointments are also booked in advance within core services.<br><br>Agree that utilisation by day of week and time of day is important. This will be monitored via the activity data.
The 'don't know' response option should be removed, in all cases, from the staff survey.	No change to staff survey, as 'don't know' response is required to ensure that those who select an alternative option do so as there is an element of certainty about their choice. Important for data quality.
The following questions are suggested to be added to the the<br>interview schedule:<br><br>a. How effective has the 7 day access scheme been in terms of number of patients visiting during extended hours?<br>b. Describe the patterns of demand during the extended hours and the average cost per 10 minute slot?	No change to interview schedule, as captured via the activity data.<br><br>No change to interview schedule, as captured via the activity data.

c. Indicate staff morale during the extended hours?	No change to interview schedule, as captured via the staff survey.
