# Supplementary material for: Investigation of the demand for a 7-day (extended access) primary care service: an observational study from pilot schemes in England
Source: BMJ Open. 2019 Sep 5;9(9):e028138. doi: 10.1136/bmjopen-2018-028138 (PMC6731947; doi:10.1136/bmjopen-2018-028138)
Supplement: Supplementary data [file bmjopen-2018-028138supp003.pdf]

Supplementary Table S3 Rates of appointment use

	Unadjusted appointment booked (95% CI)	Unadjusted appointment booked and used (95% CI)
**Day of week**		
**Monday (base category)**		
**Tuesday**	11.54 (7.90, 15.19)	9.52 (6.51, 12.53)
**Wednesday**	10.77 (3.57, 17.76)	8.20 (1.75, 14.65)
**Thursday**	12.76 (9.45, 16.08)	7.14 (3.96, 10.31)
**Friday**	9.92 (7.19, 12.65)	4.02 (0.62, 7.42)
**Saturday**	1.76 (-6.96, 10.49)	-2.00 (-9.26, 5.27)
**Sunday**	-20.35 (-36.51, -4.19)	-19.21 (-34.36, -4.05)
**Calendar month**		
**January (base category)**		
**February**	23.93 (15.15, 32.71)	20.46 (13.88, 27.04)
**March**	23.93 (17.22, 30.64)	19.52 (15.68, 23.37)
**April**	28.96 (14.72, 43.19)	24.44 (13.63, 35.25)
**May**	23.65 (11.06, 36.24)	19.82 (10.34, 29.29)
**June**	24.29 (8.32, 40.26)	21.43 (8.97, 33.89)
**July**	29.84 (17.46, 42.23)	24.18 (14.12, 34.24)
**August**	23.99 (6.93, 41.06)	20.12 (6.88, 33.37)
**September**	31.72 (13.78, 49.67)	25.77 (11.45, 40.09)
**October**	32.99 (17.24, 48.74)	28.24 (15.93, 40.56)
**November**	31.32 (14.84, 47.80)	25.69 (12.83, 38.54)
**December**	31.22 (10.11, 52.32)	23.52 (7.00, 40.04)
**CCG scheme**		
**CCG1***		
**CCG2**	-16.17 (-16.17, -16.17)	-8.46 (-8.46, -8.46)
**CCG3**	-32.83 (-32.83, -32.83)	-23.02 (-23.02, -23.02)
**CCG4**	-12.17 (-12.17, -12.17)	-8.62 (-8.62, -8.62)
**CCG5 (base category)**		
**Sample size**	42,472	42,472

Unadjusted estimates obtained via probit regression of appointment status against each set of dummies (estimated separately): day of week, calendar month, and CCG scheme.
Estimates are presented as average marginal effects which give the percentage point effect (when multiplied by 100) of the variable relative to the base category.
*CCG1 did not provide data to enable identification of whether a booked appointment was subsequently attended so does not feature in the analysis
