# Supplementary material for: Investigation of the demand for a 7-day (extended access) primary care service: an observational study from pilot schemes in England
Source: BMJ Open. 2019 Sep 5;9(9):e028138. doi: 10.1136/bmjopen-2018-028138 (PMC6731947; doi:10.1136/bmjopen-2018-028138)
Supplement: Supplementary data [file bmjopen-2018-028138supp004.pdf]

Supplementary Table S4 Comparisons of probit and logit probability model estimates

	Probit model: Probability model for appointment booked^ (95% CI)	Logit model: Probability model for appointment booked^^ (95% CI)	Probit model: Probability model for appointment used^ (95% CI)	Logit model: Probability model for appointment used^^ (95% CI)
**Day of week**				
**Monday (base category)**				
**Tuesday**	11.45 (8.51, 14.38)	2.16 (1.72, 2.71)	9.60 (7.45, 11.75)	1.63 (1.48, 1.80)
**Wednesday**	11.06 (5.89, 16.24)	2.08 (1.44, 3.01)	8.45 (3.32, 13.58)	1.53 (1.18, 1.98)
**Thursday**	12.58 (10.05 ,15.10)	2.38 (2.02, 2.80)	7.04 (4.64, 9.44)	1.42 (1.27, 1.58)
**Friday**	10.05 (7.12, 12.98)	1.91 (1.56, 2.34)	4.19 (0.88, 7.51)	1.23 (1.05, 1.43)
**Saturday**	-0.10 (-8.44, 8.24)	0.99 (0.62, 1.57)	-3.47 (-10.97, 4.01)	0.85 (0.61, 1.20)
**Sunday**	-18.93 (-35.03, -2.83)	0.41 (0.20, 0.85)	-18.07 (-32.46, -3.68)	0.46 (0.25, 0.85)
**Calendar month**				
**January (base category)**				
**February**	22.73 (13.25, 32.20)	2.98 (2.26, 3.94)	19.80 (13.15, 26.45)	2.33 (1.85, 2.93)
**March**	20.92 (11.80, 30.04)	2.70 (2.06, 3.55)	17.56 (12.25, 22.88)	2.11 (1.76, 2.52)
**April**	26.84 (11.48, 42.20)	3.91 (1.99, 7.68)	23.42 (11.91, 34.94)	2.78 (1.74, 4.43)
**May**	22.76 (7.90, 37.62)	3.01 (1.65, 5.50)	19.21 (8.32, 30.10)	2.28 (1.41, 3.49)
**June**	22.45 (5.40, 39.50)	2.96 (1.43, 6.13)	20.45 (7.37, 33.52)	2.41 (1.42, 4.08)
**July**	29.08 (16.09, 42.07)	4.43 (2.75, 7.13)	24.13 (13.98, 34.27)	2.86 (1.94, 4.22)
**August**	22.60 (4.12, 41.07)	2.97 (1.33, 6.63)	19.11 (4.95, 33.28)	2.26 (1.27, 4.02)
**September**	30.54 (12.66, 48.43)	4.89 (2.07, 11.57)	25.26 (11.19, 39.32)	3.02 (1.68, 5.47)
**October**	32.26 (15.89, 48.62)	5.60 (2.61, 12.01)	28.11 (15.33, 40.89)	3.51 (2.06, 5.98)
**November**	29.71 (12.79, 46.62)	4.67 (2.15, 10.14)	24.75 (11.59, 37.91)	2.96 (1.72, 5.10)
**December**	29.42 (6.82, 52.01)	4.62 (1.52, 14.08)	23.01 (5.75, 40.26)	2.73 (1.32, 5.64)
**CCG scheme**				
**CCG1***				
**CCG2**	-16.63 (-20.09, -13.17)	0.35 (0.30, 0.40)	-10.43 (-13.17, -7.68)	0.61 (0.54, 0.68)
**CCG3**	-32.02 (-35.24, -28.80)	0.14 (0.13, 0.15)	-23.85 (-26.36, -21.35)	0.32 (0.30, 0.35)
**CCG4**	-11.28 (-14.69, -7.87)	0.46 (0.38, 0.54)	-9.97 (-12.55, -7.38)	0.62 (0.55, 0.69)
**CCG5 (base category)**				
**Sample size**	42,472	42,472	42,472	42,472

Appointments booked are appointments booked, appointments used are appointments that were booked and subsequently attended. Probability models are probit and logit regressions of appointment status against day of week, calendar month, and CCG scheme. Standard errors are clustered at the CCG-level.
^Estimates are presented as average marginal effects which give the percentage point effect of the variable relative to the base category.
^^Estimates are presented as odds-ratios which give the odds relative to the base category.
*CCG1 did not provide data to enable identification of whether a booked appointment was subsequently attended so does not feature in the analysis
