# Supplementary material for: Investigation of the demand for a 7-day (extended access) primary care service: an observational study from pilot schemes in England
Source: BMJ Open. 2019 Sep 5;9(9):e028138. doi: 10.1136/bmjopen-2018-028138 (PMC6731947; doi:10.1136/bmjopen-2018-028138)
Supplement: Supplementary data [file bmjopen-2018-028138supp005.pdf]

**Supplementary Table S5 Probability models for appointment use with Sunday interaction with calendar month**

| | Appointment booked (95% CI) | Appointment used (95% CI) |
|---|---|---|
| **Day of week (base: Monday)** | | |
| **Tuesday** | 0.430 0.320,0.539) | 0.288 (0.237,0.339) |
| **Wednesday** | 0.410 (0.232,0.588) | 0.249 (0.104,0.394) |
| **Thursday** | 0.481 (0.408,0.553) | 0.204 (0.144,0.265) |
| **Friday** | 0.367 (0.268,0.466) | 0.119 (0.031,0.207) |
| **Saturday** | -0.010 (-0.278,0.258) | -0.098 (-0.303,0.108) |
| **Sunday** | -0.560 (-0.950,-0.170) | -0.659 (-1.127,-0.191) |
| **Calendar month (base: January)** | | |
| **February** | 0.729 (0.580,0.878) | 0.562 (0.436,0.687) |
| **March** | 0.563 (0.377,0.748) | 0.418 (0.313,0.524) |
| **April** | 0.764 (0.437,1.091) | 0.591 (0.377,0.805) |
| **May** | 0.599 (0.298,0.900) | 0.451 (0.248,0.655) |
| **June** | 0.684 (0.324,1.044) | 0.552 (0.297,0.807) |
| **July** | 0.929 (0.755,1.103) | 0.636 (0.488,0.785) |
| **August** | 0.664 (0.266,1.061) | 0.493 (0.211,0.775) |
| **September** | 0.938 (0.515,1.362) | 0.650 (0.373,0.928) |
| **October** | 0.986 (0.604,1.367) | 0.708 (0.434,0.981) |
| **November** | 0.889 (0.495,1.283) | 0.622 (0.341,0.903) |
| **December** | 0.895 (0.311,1.479) | 0.578 (0.195,0.961) |
| **Sunday*Calendar month** | | |
| **February** | -0.407 (-0.685,-0.128) | -0.229 (-0.475,0.017) |
| **March** | 0.238 (-0.036,0.512) | 0.334 (0.026,0.642) |
| **April** | 0.213 (-0.502,0.929) | 0.260 (-0.486,1.006) |
| **May** | 0.307 (-0.420,1.033) | 0.368 (-0.349,1.086) |
| **June** | -0.205 (-0.874,0.464) | -0.044 (-0.708,0.619) |
| **July** | -0.197 (-1.042,0.648) | 0.107 (-0.670,0.884) |
| **August** | -0.078 (-0.841,0.686) | 0.084 (-0.670,0.838) |
| **September** | 0.013 (-0.693,0.719) | 0.214 (-0.573,1.002) |
| **October** | 0.131 (-0.439,0.701) | 0.389 (-0.198,0.975) |
| **November** | 0.099 (-0.522,0.721) | 0.303 (-0.290,0.895) |
| **December** | -0.001 (-0.984,0.981) | 0.290 (-0.573,1.154) |
| **CCG scheme* (base: CCG5)** | | |
| **CCG2** | -0.590 (-0.703,-0.477) | -0.298 (-0.373,-0.223) |
| **CCG3** | -1.134 (-1.214,-1.053) | -0.678 (-0.742,-0.614) |
| **CCG4** | -0.395 (-0.497,-0.292) | -0.281 (-0.347,-0.214) |
| **Constant** | 0.516 (-0.046,1.077) | 0.210 (-0.205,0.625) |
| **Sample size** | 42,472 | 42,472 |

Appointments booked are appointments booked, appointments booked and used are appointments that were booked and subsequently attended.

Probit regression of appointment status against day of week, calendar month, Sunday interaction with calendar month, and CCG scheme. Standard errors are clustered at the CCG-level.
Estimates are not presented as average marginal effects due to the inability to identify the marginal effect of an interaction term.
*CCG1 did not provide data to enable identification of whether a booked appointment was subsequently attended so does not feature in the analysis
