# Supplementary material for: Investigation of the demand for a 7-day (extended access) primary care service: an observational study from pilot schemes in England
Source: BMJ Open. 2019 Sep 5;9(9):e028138. doi: 10.1136/bmjopen-2018-028138 (PMC6731947; doi:10.1136/bmjopen-2018-028138)
Supplement: Supplementary data [file bmjopen-2018-028138supp006.pdf]

Supplementary Table S6 Missing appointment data for booked appointments

CCG		Total booked	Missing patient gender	Missing patient age	Missing appointment type	Missing booking type	Missing patient's GP practice	Any missing appointment data
**CCG2**		23,345	73 (0.31%)	246 (1.05%)	0 (0.00%)	0 (0.00%)	1,902 (8.15%)	2,041 (8.74%)
	**Hub 1**	5,220	7 (0.13%)	76 (1.46%)	0 (0.00%)	0 (0.00%)	416 (7.97%)	461 (8.83%)
	**Hub 2**	6,435	37 (0.57%)	66 (1.03%)	0 (0.00%)	0 (0.00%)	504 (7.83%)	545 (8.47%)
	**Hub 3**	5,229	16 (0.31%)	70 (1.34%)	0 (0.00%)	0 (0.00%)	470 (8.99%)	493 (9.43%)
	**Hub 4**	6,461	13 (0.20%)	34 (0.53%)	0 (0.00%)	0 (0.00%)	512 (7.92%)	542 (8.39%)
**CCG4**		4,463	227 (5.09%)	227 (5.09%)	345 (7.73%)	0 (0.00%)	457 (10.24%)	759 (17.01%)
	**Hub 1**	1,878	90 (4.79%)	90 (4.79%)	141 (7.51%)	0 (0.00%)	225 (11.98%)	355 (18.90%)
	**Hub 2**	1,060	60 (5.66%)	60 (5.66%)	83 (7.83%)	0 (0.00%)	104 (9.81%)	176 (16.60%)
	**Hub 3**	1,525	77 (5.05%)	77 (5.05%)	121 (7.93%)	0 (0.00%)	128 (8.39%)	228 (14.95%)
**CCG5**		2,183	11 (0.50%)	51 (2.34%)	0 (0.00%)	3 (0.14%)	0 (0.00%)	58 (2.66%)
	**Hub 1**	1,094	8 (0.73%)	19 (1.74%)	0 (0.00%)	0 (0.00%)	0 (0.00%)	22 (2.01%)
	**Hub 2**	1,089	3 (0.28%)	32 (2.94%)	0 (0.00%)	3 (0.28%)	0 (0.00%)	36 (3.31%)
**CCG3**		2,050	0 (0.00%)	0 (0.00%)	0 (0.00%)	0 (0.00%)	23 (1.12%)	23 (1.12%)
	**Hub 1**	1,806	0 (0.00%)	0 (0.00%)	0 (0.00%)	0 (0.00%)	16 (0.89%)	16 (0.89%)
	**Hub 2**	244	0 (0.00%)	0 (0.00%)	0 (0.00%)	0 (0.00%)	7 (2.87%)	7 (2.87%)
**Total**		32,041	311 (0.97%)	524 (1.64%)	345 (1.08%)	3 (0.01%)	2,382 (7.43%)	2,881 (8.99%)
