# Supplementary material for: Investigation of the demand for a 7-day (extended access) primary care service: an observational study from pilot schemes in England
Source: BMJ Open. 2019 Sep 5;9(9):e028138. doi: 10.1136/bmjopen-2018-028138 (PMC6731947; doi:10.1136/bmjopen-2018-028138)
Supplement: Supplementary data [file bmjopen-2018-028138supp007.pdf]

Supplementary Table S7 Probability model for appointment containing missing data

	Missing appointment data^ (95% CI)
**Day of week (base: Monday)**	
**Tuesday**	0.49 (-1.51, 2.49)
**Wednesday**	1.24 (0.05, 2.42)
**Thursday**	0.22 (-0.40, 0.85)
**Friday**	0.87 (-0.29, 2.02)
**Saturday**	0.74 (-0.08, 1.55)
**Sunday**	1.43 (0.49, 2.37)
**Calendar month (base: January)**	
**February**	-0.54 (-0.93, -0.16)
**March**	-3.11 (-3.99, -2.23)
**April**	-2.53 (-4.06, -0.99)
**May**	-1.41 (-3.13, 0.30)
**June**	-0.87 (-2.64, 0.90)
**July**	-2.73 (-5.85, 0.38)
**August**	-4.11 (-5.17, -3.05)
**September**	-2.30 (-7.49, 2.88)
**October**	-3.10 (-6.15, -0.05)
**November**	-3.18 (-8.20, 1.84)
**December**	-3.40 (-9.87, 3.07)
**CCG scheme (base: CCG5)**	
**CCG2**	-6.76 (-7.12, -6.40)
**CCG3**	-
**CCG4**	-21.90 (-21.98, -21.82)
**Sample size**	29,858

^ CCG3 had 100% data completion (n=2,183)
Probit regression of missing data indicator (gender, age, appointment type, booking type, DNA status, patient's GP practice) against day of week, calendar month, and CCG scheme.
Estimates are presented as average marginal effects which give the percentage point effect (when multiplied by 100) of the variable relative to the base category.
