# Supplementary material for: Investigation of the demand for a 7-day (extended access) primary care service: an observational study from pilot schemes in England
Source: BMJ Open. 2019 Sep 5;9(9):e028138. doi: 10.1136/bmjopen-2018-028138 (PMC6731947; doi:10.1136/bmjopen-2018-028138)
Supplement: Supplementary data [file bmjopen-2018-028138supp008.pdf]

Supplementary Table S8 Booked appointment data: summary statistics

Appointment characteristic	Frequency	Percent	Cumulative percentage
**Day of week**			
**Monday**	3,145	10.79	10.79
**Tuesday**	3,335	11.44	22.22
**Wednesday**	2,970	10.19	32.41
**Thursday**	3,101	10.63	43.04
**Friday**	3,064	10.51	53.55
**Saturday**	10,744	36.84	90.39
**Sunday**	2,801	9.61	100.00
**Calendar month**			
**January**	1,310	4.49	4.49
**February**	2,115	7.25	11.75
**March**	2,522	8.65	20.39
**April**	2,530	8.68	29.07
**May**	2,522	8.65	37.72
**June**	2,240	7.68	45.40
**July**	2,695	9.24	54.64
**August**	2,487	8.53	63.17
**September**	2,568	8.81	71.98
**October**	2,936	10.07	82.05
**November**	2,710	9.29	91.34
**December**	2,525	8.66	100.00
**CCG**			
**CCG2**	21,304	73.06	73.06
**CCG3**	2,027	6.95	80.01
**CCG4**	3,704	12.70	92.71
**CCG5**	2,125	7.29	100.00
**Appointment type**			
**GP**	24,068	82.54	82.54
**Nurse**	5,092	17.46	100.00
**Booking type**			
**Pre-booked**	13,538	46.43	46.43
**Same-day**	15,622	53.57	100.00
**Attended status**			
**DNA**	3,548	12.17	12.17
**Attended**	25,612	87.83	100.00
**Gender**			
**Male**	12,071	41.40	41.40
**Female**	17,089	58.60	100.00
**Age**			
**0-9**	3,233	11.09	11.09
**10-19**	2,939	10.08	21.17
**20-29**	5,299	18.17	39.34
**30-39**	4,832	16.57	55.91
**40-49**	4,542	15.58	71.48
**50-59**	4,133	14.17	85.66
**60-69**	2,530	8.68	94.33
**70-79**	1,169	4.01	98.34
**80-89**	443	1.52	99.86
**90+**	40	0.14	100.00
**Total**	29,160	100.00	

Appointment characteristics for appointments booked and used with complete (non-missing) data
