# Supplementary material for: Investigation of the demand for a 7-day (extended access) primary care service: an observational study from pilot schemes in England
Source: BMJ Open. 2019 Sep 5;9(9):e028138. doi: 10.1136/bmjopen-2018-028138 (PMC6731947; doi:10.1136/bmjopen-2018-028138)
Supplement: Supplementary data [file bmjopen-2018-028138supp009.pdf]

Supplementary Table S9 Booked appointment data: summary statistics by appointment type

Appointment characteristic	Pre-booked	%	Same-day	%	GP	%	Nurse	%	Used	%	DNA	%
**Day of week**												
**Monday**	650	5.70	2,495	15.97	1,893	10.32	476	9.35	2,854	11.16	281	7.92
**Tuesday**	1,380	12.09	1,955	12.51	1,975	10.77	546	10.72	3,001	11.74	332	9.36
**Wednesday**	1,401	12.28	1,569	10.04	1,940	10.58	414	8.13	2,651	10.37	317	8.93
**Thursday**	1,437	12.59	1,664	10.65	1,907	10.40	559	10.98	2,667	10.43	432	12.18
**Friday**	1,449	12.70	1,593	10.20	1,945	10.61	458	8.99	2,606	10.19	455	12.82
**Saturday**	3,862	33.84	4,779	30.59	7,076	38.59	2,189	43.17	9,283	36.30	1,444	40.70
**Sunday**	1,234	10.81	1,567	10.03	1,601	8.73	441	8.66	2,510	9.82	287	8.09
**Calendar month**												
**January**	404	3.54	798	5.11	992	5.41	41	0.81	1,157	4.52	151	4.26
**February**	785	6.88	1,156	7.40	1,643	8.96	66	1.30	1,857	7.26	256	7.22
**March**	1,162	10.18	1,204	7.71	1,905	10.39	57	1.12	2,183	8.54	338	9.53
**April**	1,055	9.24	1,270	8.13	1,569	8.56	478	9.39	2,210	8.64	318	8.96
**May**	986	8.64	1,357	8.69	1,532	8.35	532	10.45	2,192	8.57	323	9.10
**June**	869	7.61	1,202	7.69	1,368	7.46	488	9.58	1,982	7.75	253	7.13
**July**	1,036	9.08	1,427	9.13	1,615	8.81	629	12.35	2,320	9.07	374	10.54
**August**	935	8.19	1,369	8.76	1,454	7.93	610	11.98	2,168	8.48	313	8.82
**September**	1,096	9.60	1,289	8.25	1,482	8.08	628	12.33	2,267	8.87	299	8.43
**October**	1,085	9.51	1,626	10.41	1,623	8.85	685	13.45	2,635	10.30	296	8.34
**November**	999	8.75	1,545	9.89	1,562	8.52	549	10.78	2,409	9.42	298	8.40
**December**	1,001	8.77	1,379	8.83	1,592	8.68	329	6.46	2,192	8.57	329	9.27
**CCG**												
**CCG2**	8,922	78.17	12,382	79.26	17,057	93.02	4,247	83.41	18,640	72.89	2,632	74.18
**CCG3**	519	4.55	1,508	9.65	-	-	-	-	1,828	7.15	197	5.55
**CCG4**	1,972	17.28	1,732	11.09	-	-	-	-	3,297	12.89	404	11.39
**CCG5**	-	-			1,280	6.98	845	16.59	1,807	7.07	315	8.88
**Appointment type**												
**GP**	9,181	80.44	13,607	87.10	-	-	-	-	21,082	82.44	2,960	83.43
**Nurse**	2,232	19.56	2,015	12.90	-	-	-	-	4,490	17.56	588	16.57
**Booking type**												
**Pre-booked**	-	-	-	-	7,970	43.46	3,077	60.43	11,279	44.11	2,244	63.25

**Same-day**	-	-	-	-	10,367	56.54	2,015	39.57	14,293	55.89	1,304	36.75
**Attended status**												
**DNA**	1,929	16.90	1,304	8.35	2,359	12.86	588	11.55	-	-	-	-
**Used**	9,484	83.10	14,318	91.65	15,978	87.14	4,504	88.45	-	-	-	-
**Gender**												
**Male**	4,775	41.84	6,547	41.91	7,782	42.44	1,803	35.41	10,609	41.49	1,450	40.87
**Female**	6,638	58.16	9,075	58.09	10,555	57.56	3,289	64.59	14,963	58.51	2,098	59.13
**Age**												
**0-9**	831	7.28	2,202	14.10	2,293	12.50	73	1.43	2,825	11.05	408	11.50
**10-19**	1,158	10.15	1,594	10.20	1,961	10.69	302	5.93	2,508	9.81	431	12.15
**20-29**	2,178	19.08	2,731	17.48	3,424	18.67	885	17.38	4,343	16.98	956	26.94
**30-39**	1,962	17.19	2,445	15.65	2,978	16.24	934	18.34	4,093	16.01	739	20.83
**40-49**	1,859	16.29	2,329	14.91	2,733	14.90	965	18.95	4,054	15.85	488	13.75
**50-59**	1,783	15.62	2,041	13.06	2,515	13.72	893	17.54	3,813	14.91	320	9.02
**60-69**	991	8.68	1,382	8.85	1,502	8.19	583	11.45	2,418	9.46	112	3.16
**70-79**	462	4.05	645	4.13	671	3.66	302	5.93	1,106	4.33	63	1.78
**80-89**	177	1.55	228	1.46	239	1.30	141	2.77	412	1.61	31	0.87
**90+**	12	0.11	25	0.16	21	0.11	14	0.27	-	-	-	-
**Total**	11,413	100.00	15,662	100.00	18,337	100.00	5,092	100.00	25,572	100.00	3,548	100.00

Appointment characteristics for appointments booked and used with complete (non-missing) data
