# Supplementary material for: Investigation of the demand for a 7-day (extended access) primary care service: an observational study from pilot schemes in England
Source: BMJ Open. 2019 Sep 5;9(9):e028138. doi: 10.1136/bmjopen-2018-028138 (PMC6731947; doi:10.1136/bmjopen-2018-028138)
Supplement: Supplementary data [file bmjopen-2018-028138supp010.pdf]

Supplementary Table S10 Demographic distributions of extended access appointment users

	Males			Females			All
	Frequency	%	Cumulative %	Frequency	%	Cumulative %	Cumulative %
**Extended access**							
**Age**							
**20-29**	1,446	17.71	17.71	2,897	23.93	23.93	21.43
**30-39**	1,575	19.30	37.01	2,513	20.75	44.68	41.60
**40-49**	1,617	19.81	56.82	2,435	20.11	64.79	61.59
**50-59**	1,690	20.71	77.53	2,123	17.53	82.32	80.40
**60-69**	1,144	14.02	91.55	1,273	10.51	92.83	92.32
**70-79**	481	5.89	97.44	624	5.15	97.98	97.77
**80-89**	197	2.41	99.85	215	1.78	99.76	99.80
**90+**	12	0.15	100.00	28	0.23	100.00	100.00
**Total**	8,162	100.00		12,108	100.00		
**GP Patient Survey users***							
**Age**							
**18-24**	298	8.45	8.45	514	10.53	10.53	9.55
**25-34**	472	13.39	21.83	798	16.35	26.88	24.51
**35-44**	618	17.52	39.36	812	16.64	43.52	41.56
**45-54**	720	20.43	59.79	912	18.69	62.21	61.07
**55-64**	608	17.26	77.04	691	14.15	76.36	76.68
**65-74**	493	14.00	91.04	578	11.85	88.21	89.54
**75-84**	251	7.11	98.15	383	7.85	96.06	97.04
**85+**	65	1.85	100.00	192	3.94	100.00	100.00
**Total**	3,524	100.00		4,881			

*GPPS weighted responses for patients from practices in CCGs 2 to 5, rounded to nearest whole number
