# Supplementary material for: Investigation of the demand for a 7-day (extended access) primary care service: an observational study from pilot schemes in England
Source: BMJ Open. 2019 Sep 5;9(9):e028138. doi: 10.1136/bmjopen-2018-028138 (PMC6731947; doi:10.1136/bmjopen-2018-028138)
Supplement: Author's manuscript [file bmjopen-2018-028138.draft_revisions.pdf]

BMJ Open

BMJ Open is committed to open peer review. As part of this commitment we make the peer review history of every article we publish publicly available.

When an article is published we post the peer reviewers' comments and the authors' responses online. We also post the versions of the paper that were used during peer review. These are the versions that the peer review comments apply to.

The versions of the paper that follow are the versions that were submitted during the peer review process. They are not the versions of record or the final published versions. They should not be cited or distributed as the published version of this manuscript.

BMJ Open is an open access journal and the full, final, typeset and author-corrected version of record of the manuscript is available on our site with no access controls, subscription charges or pay-per-view fees (http://bmjopen.bmj.com).

If you have any questions on BMJ Open's open peer review process please email info.bmjopen@bmj.com

BMJ Open

An investigation of the demand for a 7-day (extended access) primary care service: an observational study from pilot schemes in England

Journal:	*BMJ Open*
Manuscript ID	bmjopen-2018-028138
Article Type:	Research
Date Submitted by the Author:	23-Nov-2018

Complete List of Authors:	Whittaker, William; University of Manchester, Manchester Centre for Health Economics<br>Anselmi, Laura; University of Manchester, Manchester Centre for Health Economics<br>Nelson, Pauline; University of Manchester Alliance Manchester Business School; NIHR CLAHRC Greater Manchester, UK<br>O'Donnell, Caroline; NIHR CLAHRC Greater Manchester, UK<br>Ross, Natalie; University of Manchester Alliance Manchester Business School<br>Rothwell, Katy; NIHR CLAHRC Greater Manchester, UK<br>Hodgson, Damian; University of Manchester Alliance Manchester Business School; NIHR CLAHRC Greater Manchester, UK

Keywords:	PRIMARY CARE, ACCESS, HEALTH ECONOMICS, Health policy < HEALTH SERVICES ADMINISTRATION & MANAGEMENT

SCHOLARONE™
Manuscripts

An investigation of the demand for a 7-day (extended access) primary care service: an observational study from pilot schemes in England

Whittaker W[1], Anselmi L[1], Nelson P[2,3], O'Donnell C[3], Ross N[2], Rothwell K[3], Hodgson D[2,3].

[1]Manchester Centre for Health Economics, University of Manchester, UK
[2]Alliance Manchester Business School, University of Manchester, UK
[3]NIHR CLAHRC Greater Manchester, UK

Correspondence to:
William.whittaker@manchester.ac.uk
Manchester Centre for Health Economics, 4.320 Jean McFarlane Building, University of Manchester, Oxford Road, Manchester, M13 9PL

Word count: 4,450

For peer review only

For peer review only - http://bmjopen.bmj.com/site/about/guidelines.xhtml

ABSTRACT

Objectives - To understand how the uptake of an extended primary care service in the evenings and weekend varied by day of week and over time. Secondary objectives were to understand patient demographics of users of the service and how these varied by type of appointment and to core hour users.

Design – Observational study.

Setting - Primary care extended access appointments data in 13 centres in Greater Manchester, England during 2016.

Participants - 1,261,326 participants registered with a family practitioner in five of ten Clinical Commissioning Group geographic areas.

Main outcome measures – Primary outcome measure was whether an appointment was used (booked and attended), secondary outcome measures included whether used appointments were pre-booked or booked the same-day, and delivered by a family or nurse practitioner. Additional analyses compared participant demographics to patients reporting the use of core-hour primary care services.

Results - 65.33% of 42,472 appointments were booked and attended (used). Uptake of appointments was lowest on a Sunday at 46.73% (18.07 percentage points lower uptake than on Mondays (95%CI -32.46 to -3.68)). Uptake increased over the 2016 calendar year. Pre-booked appointments participants were less likely to be female (-0.66 percentage points, 95%CI: -1.08 to -0.25) and more likely to be amongst age groups 0 to 9 and 90 years and over. Family practitioner appointments were increasingly less likely with age in comparison to nurse appointments. Participants attending extended access appointments tended to be younger in comparison to core hour patients.

Conclusions - There is spare capacity in the extended access service. However, uptake improved over time, for all week days. Patients demographics suggest the service may be reaching a previously unmet need. In addition, patient demographics varied with the types of appointment provided. This may help healthcare providers improve uptake by tailoring appointment provision to local populations.

Strengths and limitations of this study

- The first study assessing same-day and pre-bookable appointments at evenings and weekends in general practice
- The first study comparing demographics of patients using evening and weekend appointments to patients using core hour appointments
- The first study assessing uptake of evening and weekend appointments over time
- The study covers five areas with differing provision, but may not be generalisable to other settings
- No information on child use of core hours was available, limiting an assessment of this group of the population

For peer review only - http://bmjopen.bmj.com/site/about/guidelines.xhtml

What this paper adds

Section 1: What is already known on this subject

- Health economies worldwide see patients reporting poor access to primary care, resulting in patient dissatisfaction and subsequent use of capacity-constrained secondary care
- Extending primary care appointments into the evening and weekend reduces accident and emergency use but these reductions do not offset the expense of the extended service
- There is a concern that extended primary care appointments are not being used, raising questions on the value of the service

Section 2: What this study adds

- Data from a regional pilot in 2016 saw 65.33% of available extended general practice appointments booked and attended; uptake varied over the week with Sunday uptake considerably smaller, putting the aim of improving primary care access into question. However, uptake improved over time, for all week days
- Patients using the service were younger and more likely to be female than those using core primary care services, which may signal the service is reaching a previously unmet need
- In addition, the types of appointment (pre-bookable, same-day, GP or nurse) were used differentially based on patient demographics. This may help healthcare providers improve uptake by tailoring appointment provision to local populations.

For peer review only

INTRODUCTION

Providing access to health care services when needed is a key target for health systems worldwide.[1] However, patients commonly report difficulties in accessing primary care, particularly outside of core working hours.[2,3] Constrained access to primary care has been a reason for increased patient dissatisfaction and use of emergency care in a variety of countries and settings.[4] Rising use of emergency department services and patient dissatisfaction with access have highlighted a need for better access to primary care.

In England primary care core hour services are provided by General Practitioners (GP) 8am to 5pm Monday to Friday either by pre-booking or calling on the same day. The National Health Service (NHS) in England aims to extend access to primary care services in the evening and at weekends as part of their strategy for delivering primary care by 2020/2021.[5] The strategy seeks to enable local commissioners of health care to redesign primary care services and commission extra capacity so that by 2020 *'everyone has access to GP services, including sufficient routine appointments at evenings and weekends to meet locally determined demand, alongside effective access to out-of-hours (OoH) and urgent care services'*.[5, page 47] Extended access has been piloted nationally by NHS England since 2013 through the Prime Minister's Challenge Fund, and its successor the GP Access Fund. Wave 1 covered 7.5m people in 2014, rising to 18m (a third of the population of England) by 2015 in Wave 2. In parallel, NHS England Greater Manchester (NHSEGM), now the GM Health and Social Care Partnership (GMHSCP), have also piloted extended access appointments with an initial wave in 2014[6], followed by a roll-out throughout Greater Manchester from 2016 in line with the region's devolution and health and social care strategy[7]. A description of extended access appointments in Greater Manchester is provided in Box 1.

Box 1: Description of Greater Manchester extended access appointments in primary care

<u>What is the service?</u>
Extended access appointments are appointments in primary care outside of core hours (typically 8am to 5pm). Appointments are provided in addition to usual care outside of core family practitioner hours which includes: Walk in Centres, Out-of-Hour services, Accident and Emergency/secondary care, NHS Direct ('111'). The services may have been designed to replace existing Direct Enhanced Services arrangements (additional hours of opening contractually agreed with NHS England).

<u>Who provided the service?</u>
Extended access appointments are appointments with either a GP or nurse practitioner.

<u>How is the service delivered?</u>
Extended access appointments are typically face to face appointments delivered individually.

For peer review only

For peer review only - http://bmjopen.bmj.com/site/about/guidelines.xhtml

Where is the service delivered?
Extended access appointments are usually provided through one practice or similar premises which acts as a local 'hub' covering several practices in a given geographic area, with between 1-4 hubs serving the patient population. Hub locations were decided by local health care commissioners (Clinical Commissioning Groups, CCGs). CCGs are responsible for the commissioning of health care to a local, geographically defined population comprising of a number of family practitioner (GP) practices.

When and how much of the service is delivered?
Extended access appointments under this study became operational at varying times over the 2016 calendar year, this varied by CCG.

Tailoring of the service
The type, referral route(s) and timing of appointments are decided by the local CCG. Variations between schemes existed by referral route, whether pre-bookable appointments were available, discipline of appointment (GP or nurse, all face to face), time and day of appointments.

Extended access evaluations to date have found reductions in emergency service use for patients with minor injuries/issues in national[8], sub-national[9], and Greater Manchester[6, 10] schemes. However, the reductions in emergency service use alone may not sufficiently offset the cost of providing an extended service, raising concerns that an extended access service is not a cost-effective use of health system resources.[10] Concerns of opportunity cost (the net benefits of services that could have been provided instead of extended access) are particularly pertinent in times of increasing financial/workforce pressures for many health systems. However, in addition to impacts on secondary care, and particularly in the current absence of an understanding of the long-term health benefits of the service; there are additional aspects of extended access that need to be considered when assessing the value of the service. First, there is a lack of knowledge concerning how the service is being used with little assessment of how best to operationalise extended appointments efficiently (which would have implications for cost-effectiveness). One way to measure efficiency could be uptake of the service, with unbooked appointments signaling inefficient use of resources. Uptake of extended access appointments has been reported as 71% with lower uptake on weekends, particularly Sundays.[8, 11] A sub-national evaluation of 3 schemes found uptake was lower where appointments were not pre-bookable, and lower on Sundays.[11] An evaluation of the initial wave of Greater Manchester 'demonstrators' found uptake of 65.5% and lower uptake on weekends, particularly on Sundays.[6] Studies of uptake have concentrated on high level aggregated descriptive statistics with no formal statistical tests conducted to infer whether there is a statistically significant difference in uptake over time and/or by day of week. Since service provision varies across pilots it is also important to understand how uptake varies across services delivered. Commissioners are tasked with delivering extended access services in light of patient demand[5, page 48], the lack of evidence to inform

For peer review only - http://bmjopen.bmj.com/site/about/guidelines.xhtml

efficient provision is a substantial gap in knowledge. Second, it is currently unknown what the patient demographics are for an extended access service delivering pre-bookable and same-day appointments with existing analyses being for small scale schemes[11] or urgent care appointments only[12], it is also unclear whether patients using the service differ from patients using core hours general practice, understanding these may help inform service provision and infer whether the service is hitting an unmet need and/or reducing pressures in core hours.

Our aim in this paper is threefold. First, using data from Greater Manchester UK schemes throughout 2016 we sought to understand whether the service is being used (uptake) and whether this varies by day of week and over time. Second, we sought to understand whether there were differences in the services being used by particular age and gender groups. Third, we aimed to compare the users of extended access appointments to users of general practice core hours. The findings will help inform considerations about extending access to general practice and how such a service might be better targeted to particular population groups.

METHODS

Data

The study evaluated the second wave of Greater Manchester schemes that delivered extended access throughout 2016. Out of Greater Manchester's ten Clinical Commissioning Group (CCG) areas, three were already delivering extended access under the national GPAF scheme during the study period (see Figure 1). In line with the GM Health and Social Care Partnership Primary Care Strategy, the other seven CCG areas, not previously in receipt of Wave 1 PMCF or Wave 2 GPAF funding, received financial support from GMHSCP to implement extended access. However, only five of these seven CCGs were included in the evaluation because two of the CCGs were not captured in the data collection process: one had not implemented the service in 2016; the other did not provide data. A table summarising the five CCG schemes with extended access can be found in Supplementary Table S1. The evaluation was carried out by the National Insitute for Health Rearch Collaboration for Leadership in Applied Health Research and Care Greater Manchester (NIHR CLAHRC GM).

<Figure 1 here>

We used administrative data on extended access appointments available, booked and attended in CCG schemes in Greater Manchester by calendar date over the year 2016. The NIHR CLAHRC GM evaluation team requested a minimum dataset from each CCG (Supplementary Table S2). The aim of

For peer review only - http://bmjopen.bmj.com/site/about/guidelines.xhtml

For peer review only

this data collection was to inform what new appointment activity was associated with the extended access schemes, whether this new activity was used, and to understand the types of patients using the service (a protocol for the evaluation can be found in Supplementary Text S1). Data on each appointment made available were collected and made available by the responsible CCG. CCGs submitted the data to NIHR CLAHRC GM throughout the period. The data included information on date, use (volume available, booked, booked and used), type of appointment (family practitioner or nurse, pre-booked or booked same-day), and patient characteristics (the patient's registered practice, age and gender) for booked and attended appointments. To maintain anonymity, patient age was given in 10-year age bands. Deprivation statistics were considered although, due to data confidentiality, we were only able to obtain partial patient postcodes. Since partial postcodes do not enable a suitable deprivation measure to be derived, no deprivation analysis was conducted for the analyses.

Appointment data for each CCG area was collected and analysed once a hub became operational. Live dates varied across CCGs, and within CCGs there was variation in hub start dates. A patient residing within their CCG could use any hub services within the CCG.

Data on general practice core hour use was sourced from the the GP Patient Survey (GPPS).[13] This is a cross-sectional survey of randomly selected patients from each practice within England. Patients need to be registered with a practice, have an NHS number, and be aged 18 and over to be sampled. Patients are asked questions concerning the use of primary care, perceptions of primary care, and patient demographics.

Analysis

All estimation was conducted in Stata (version 14). We assessed uptake, variations in patient demographics for different types of extended access services, and made comparisons of extended access appointment users to core hour users as follows:

(i) Uptake

Patients could book an appointment which could then be attended or not. Focussing on booked appointments alone would give an indication of the demand for the service, whilst assessments of appointments actually used shed light on actual impacts on service use. We estimated two separate sets of probability models:

1) the probability an appointment was booked, and;
2) the probability an appointment was booked and attended ('used').

For peer review only - http://bmjopen.bmj.com/site/about/guidelines.xhtml

Each model was estimated via probit regressions (see Suppementary Text S2 for a technical description of the methods). Probit models were chosen as these give the absolute variation in uptake. To assess whether uptake of the service varied across day of the week we included day of week dummies in the regression, with the estimates providing the differences in the rate of uptake relative to the base week day (Monday). This enabled a test for whether uptake was lower on particular days. To assess whether there was evidence of assimilation to the service we included calendar month dummies, if assimilation is evident the estimates on the calendar month dummies should be monotonically increasing over the period. To account for potential confounding caused by variations in the provision and delivery of services across schemes, we included CCG dummies (fixed-effects) in the regression. Robust standard errors were estimated to address concerns of heteroscedasticity and clustered at CCG. Estimates are presented as average marginal effects, which give the average percentage point effect on uptake of the variable relative to the base category across the sample.

Analyses were restricted to four CCG schemes due to the inability to differentiate whether a booked appointment had been attended in one CCG (CCG1).

(ii) Patient demographics analysis

Our second set of analyses concentrated on booked appointments and exploited variations in service delivery to test whether there were variations in patient demographics for particular types of services. We estimated two separate sets of probability models:

1) The probability an appointment was pre-booked versus same day;
2) The probability an appointment was for a GP versus a nurse

In each model, we included dummies for gender (female), 10-year age bands, day of the week of the appointment, month of the appointment, a flag for whether the patient did not attend (DNA) the appointment, and CCG. Each model was estimated via probit regressions (see Supplementary Text S2 for a technical description of the methods). For all models, estimates are presented as average marginal effects, with standard errors clustered at CCG. Schemes that exclusively provided a certain service (e.g. only GP appointments) were excluded as a result of uptake of the alternative being necessarily zero.

Missing data on booked appointments were treated as missing at random. To evaluate whether or not missing data might have been associated with the covariates in our models (and hence potentially bias our estimates) we generated a binary missing data indicator for each appointment used. The

For peer review only - http://bmjopen.bmj.com/site/about/guidelines.xhtml

binary missing data indicator was coded as 1 where missing data existed for either: age, gender, appointment type, or booking type. This was regressed via a probit model against day of the week, calendar month, and CCG indicators to test whether missing data was related to particular days or months of provision and/or the unit of delivery (CCG).

(iii) Comparisons of extended access appointment users to core hour users

Finally, to inform the potential implications of the service on equity of access to primary care, the demographics of extended access patients that booked and attended their appointment were compared to age and gender distributions of patients reporting using their practice within the last year from the January-March 2013 wave of the GP Patient Survey.[13] Patients reporting in the January to March 2013 wave were selected to proxy for patient demographics in usual care. Later waves of the GPPS could cause potential bias as patients reporting having used primary care may have used an extended access service which had been available in areas of Greater Manchester since 2014. The GPPS sample was reduced to patients registered with a practice within one of the four CCG areas under evaluation and further reduced to those patients using primary care over the last year. These patients were determined via responses to Question 1 of the survey: "*When did you last see or speak to a GP from your GP surgery?*", with patients reporting "*In the past 3 months*", "*Between 3 and 6 months ago*", and "*Between 6 and 12 months ago*". The age groupings of these respondents did not align with the age groupings in the appointment data. As such, to ease comparisons, population pyramids are presented to give an overall feel for similarities and/or differences between the two groups of patients. The GPPS may suffer from response bias. We therefore weight practice patient use measures in this survey by the sampling weights to adjust for non-response provided by the GPPS which accounts for non-response related to age and gender.[13]

Patient and public involvement

A panel of patients reviewed an earlier draft of the protocol, where feasible the protocol was adapted in light of patient feedback. The response from patients in relation to the activity analyses focussed on being able to capture variations by day of week and type of appointment, no changes in data capture was necessary following patient responses as these concerns were anticipated to be addressed in the appointment activity analyses (see Supplementary Text S3).

RESULTS

Uptake

For peer review only - http://bmjopen.bmj.com/site/about/guidelines.xhtml

A total of 51,806 extended access appointments were provided throughout 2016 by the five CCGs under the Greater Manchester scheme (Table 1), 41.07 appointments per 1,000 of the population (the population covered by these CCGs is 1,261,326). 2,315 of these, across two CCGs, were 'blocked' and not bookable by patients resulting in a total of 49,491 bookable appointments. CCG1 was removed from the analysis since no data on whether the patient attended was provided leaving a sample of 42,472 (49,491 appointments – 7,019 CCG1 appointments). 75.44% of 42,472 appointments were booked and 65.33% were booked and attended. Provision and uptake varied across the CCGs, with CCG2 dominating provision and scale per 1,000 of the population and CCG5 having highest percentage uptake. The types of appointments differed across the CCG schemes (Table 2). Two of the four schemes provided both GP and nurse appointments, the other GP only, and one scheme provided only pre-bookable appointments. Of all appointments booked, 53.27% were pre-booked and 81.42% were for a GP appointment.

Table 1 Volume of appointments bookable, booked, and DNA

Provider	Appointments	Blocked appointments	Appointments available	Appointments not booked*	Appointments booked*	% appointments available	Booked DNAs**	% appointments available	Appointments booked and used	% appointments available
**CCG1**										
**Hub 1**	3,597	0	3,597	698	2,899	80.59				
**Hub 2**	3,422	0	3,422	802	2,620	76.56				
**Total**	7,019	0	7,019	1,500	5,519	78.63				
**Total per 1,000**	23.23 (P=302,163)									
**CCG2**										
**Hub 1**	7,971	496	7,475	2,255	5,220	69.83	567	10.86	4,649	62.19
**Hub 2**	8,414	208	8,206	1,771	6,435	78.42	1,006	15.63	5,429	66.16
**Hub 3**	8,131	774	7,357	2,128	5,229	71.08	577	11.03	4,650	63.21
**Hub 4**	8,177	433	7,744	1,283	6,461	83.43	809	12.52	5,652	72.99
**Total**	32,693	1,911	30,782	7,437	23,345	75.84	2,959	12.68	20,380	66.05
**Total per 1,000**	143.77 (P=227,391)									
**CCG3**										
**Hub 1**	3,053	0	3,053	1,247	1,806	59.15	185	10.24	1,621	53.10
**Hub 2**	584	0	584	340	244	41.78	17	6.97	227	38.87
**Total**	3,637	0	3,637	1,587	2,050	56.37	202	9.85	1,848	50.81
**Total per 1,000**	14.63 (P=248,667)									
**CCG4**										
**Hub 1**	2,270	0	2,270	392	1,878	82.73	338	18.00	1,538	67.75
**Hub 2**	1,296	0	1,296	236	1,060	81.79	181	17.08	877	67.67
**Hub 3**	2,034	0	2,034	509	1,525	74.98	239	15.67	1,284	63.13
**Total**	5,600	0	5,600	1,137	4,463	79.70	758	16.98	3,699	66.05
**Total per 1,000**	22.92 (P=244,325)									
**CCG5**										
**Hub 1**	1,429	203	1,226	132	1,094	89.23	178	16.27	916	74.71
**Hub 2**	1,428	201	1,227	138	1,089	88.75	184	16.90	904	73.68
**Total**	2,857	404	2,453	270	2,183	88.99	362	16.58	1,820	74.19

For peer review only - http://bmjopen.bmj.com/site/about/guidelines.xhtml

**Total per 1,000**	11.96 (P=238,780)									
**All Schemes Combined**										
	51,806	2,315	49,491	11,931	37,560	75.89	4,281	10.08^	27,747	65.33^
**Total per 1,000**	41.07 (P=1,261,326)									
**Schemes CCG2 to CCG5**										
	44,787	2,315	42,472	10,431	32,041	75.44	4,281	10.08	27,747	65.33
**Total per 1,000**	46.84 (P=956,163)									

CCG5 blocked are for administrative purposes.
*Booked are appointments that were used (can be either pre-booked or booked same-day).
**CCG1 did not record DNAs we assume all attended for the GM % booked and used; Missing data on DNA evident for 6 appointments in CCG2, 6 in CCG4, and 1 in CCG5.
^as a percentage of appointments where attendance was captured (42,472 appointments: 49,491 appointments minus 7,019 CCG1 appointments)

Table 2 Volume of appointments by appointment type (GP/nurse) and booking type (pre-booked/same-day)

Provider	Appointments available	GP*	% GP	Nurse*	% Nurse	Appointments booked	Pre-booked**	% Pre-booked	Same-day**	% Same-day
**CCG2**										
**Hub 1**	7,475	5,906	79.01	1,569	20.99	5,220	2,080	39.85	3,140	60.15
**Hub 2**	8,206	6,466	78.80	1,740	21.20	6,435	3,184	49.48	3,251	50.52
**Hub 3**	7,357	6,157	83.69	1,200	16.31	5,229	1,844	35.26	3,385	64.74
**Hub 4**	7,744	5,723	73.90	2,021	26.10	6,461	2,697	41.74	3,764	58.26
**Total**	30,782	24,252	78.79	6,530	21.21	23,345	9,805	42.00	13,540	58.00
**CCG3**										
**Hub 1**	3,053	3,053	100.00	0	0.00	1,806	446	24.70	1,360	75.30
**Hub 2**	584	584	100.00	0	0.00	244	78	31.97	166	68.03
**Total**	3,637	3,637	100.00	0	0.00	2,050	524	25.56	1,526	74.44
**CCG4**										
**Hub 1**	2,270	2,129	93.79	0	0.00	1,878	1,164	61.98	714	38.02
**Hub 2**	1,296	1,213	93.60	0	0.00	1,060	693	65.38	367	34.62
**Hub 3**	2,034	1,913	94.05	0	0.00	1,525	603	39.54	922	60.46
**Total**	5,600	5,255	93.84	0	0.00	4,463	2,460	55.12	2,003	44.88
**CCG5**										
**Hub 1**	1,226	713	58.16	513	41.84	1,094	1,094	100.00	0	0.00
**Hub 2**	1,227	725	59.09	502	40.91	1,089	1,086	99.72	0	0.00
**Total**	2,453	1,438	58.62	1,015	41.38	2,183	2,180	99.86	0	0.00
**Schemes CCG2 to CCG5**										
	42,472	34,582	81.42	7,545	17.76	32,041	14,969	46.72	17,069	53.27

Information on pre-booked or same-day only available for booked appointments
*GP/Nurse contains missings for CCG4 (345: 141 at Hub 1; 83 at Hub 2; 121 at Hub 3)
** CCG5 have 3 appointments with no data on booked time.

Figure 2 plots appointments booked and used (‘used’), booked and not attended (‘DNA’) and not booked over the 2016 calendar year. Uptake appears to have improved slightly over time. 15,575 appointments (36.67%) occurred on a Saturday, and Sunday was the second most active day (5,878, 13.84%) (Figure 3). 52.93% of appointments on a Sunday were booked (46.73% used), and 75.06% (63.94% used) on a Saturday. Weekday attendance was generally higher at 73.29-86.06% (65.94-75.46% used).

For peer review only

<Figure 2 here>

<Figure 3 here>

The results from the probability models of uptake are contained in Table 3 (unadjusted analyses are presented in Supplementary Table S3). The results broadly confirm the summary statistics with Monday having the lowest percentage of appointments booked of other weekdays (Tuesday-Friday), and Sunday having lowest percentage of appointments booked overall (-18.9 percentage points compared to Monday (95% CI: -35.0 to -2.8)). The results are similar for appointments used. Uptake over the year was greater for all months compared to January and greater in the second half of the year. There were variations in uptake at CCG level with all CCGs having lower uptake compared to CCG5.

Table 3 Rates and probability models for appointment use

	% booked	Appointment booked (95% CI)	% used	Appointment used (95% CI)
**Day of week**				
**Monday (base category)**	73.29		65.94	
**Tuesday**	84.84	11.45 (8.51, 14.38)	75.46	9.60 (7.45, 11.75)
**Wednesday**	84.06	11.06 (5.89, 16.24)	74.14	8.45 (3.32, 13.58)
**Thursday**	86.06	12.58 (10.05 ,15.10)	73.07	7.04 (4.64, 9.44)
**Friday**	83.21	10.05 (7.12, 12.98)	69.96	4.19 (0.88, 7.51)
**Saturday**	75.06	-0.10 (-8.44, 8.24)	63.94	-3.47 (-10.97, 4.01)
**Sunday**	52.94	-18.93 (-35.03, -2.83)	46.73	-18.07 (-32.46, -3.68)
**Calendar month**				
**January (base category)**	49.52		43.87	
**February**	73.45	22.73 (13.25, 32.20)	64.33	19.80 (13.15, 26.45)
**March**	73.45	20.92 (11.80, 30.04)	63.39	17.56 (12.25, 22.88)
**April**	78.48	26.84 (11.48, 42.20)	68.31	23.42 (11.91, 34.94)
**May**	73.17	22.76 (7.90, 37.62)	63.69	19.21 (8.32, 30.10)
**June**	73.82	22.45 (5.40, 39.50)	65.31	20.45 (7.37, 33.52)
**July**	79.37	29.08 (16.09, 42.07)	68.05	24.13 (13.98, 34.27)
**August**	73.52	22.60 (4.12, 41.07)	64.00	19.11 (4.95, 33.28)
**September**	81.25	30.54 (12.66, 48.43)	69.64	25.26 (11.19, 39.32)
**October**	82.25	32.26 (15.89, 48.62)	72.12	28.11 (15.33, 40.89)
**November**	80.85	29.71 (12.79, 46.62)	69.56	24.75 (11.59, 37.91)
**December**	80.74	29.42 (6.82, 52.01)	67.40	23.01 (5.75, 40.26)
**CCG scheme**				
**CCG1***				
**CCG2**	75.48	-16.63 (-20.09, -13.17)	66.21	-10.43 (-13.17, -7.68)

For peer review only - http://bmjopen.bmj.com/site/about/guidelines.xhtml

**CCG3**	56.37	-32.02 (-35.24, -28.80)	50.81	-23.85 (-26.36, -21.35)
**CCG4**	79.70	-11.28 (-14.69, -7.87)	66.05	-9.97 (-12.55, -7.38)
**CCG5 (base category)**	88.99		74.19	
**Sample size**	42,472	42,472	42,472	42,472

Appointments booked are appointments booked, appointments used are appointments that were booked and subsequently attended.
Probit regression of appointment status against day of week, calendar month, and CCG scheme.
Estimates are presented as average marginal effects which give the percentage point effect of the variable relative to the base category.
*CCG1 did not provide data to enable identification of whether a booked appointment was subsequently attended so does not feature in the analysis

To test whether Sunday uptake improved over and above the general trend over the period we replicated our analysis with the inclusion of Sunday-calendar month interaction terms (Supplementary Table S4). The interaction terms were not significantly different from zero suggesting any change over time was equivalent to that observed Monday to Saturday.

Patient demographics analysis

The analyses concerning variations in patient demographics for particular types of appointments is limited to booked appointments (Figure 1). Of the 37,560 booked appointments, 5,519 were removed due to data reporting by CCG1, giving 32,041 appointments over the remaining four CCGs. Missing data on appointment type, booking type, and patient's gender and age amounted to a further 887 appointments being excluded from the analyses (Supplementary Table S5). Missing data varied by CCG but was similar within CCGs by hub. One CCG provided 100% complete data. Missing data does not appear to be associated with calendar month though was slightly higher on Friday and Saturday appointments (0.9 and 0.7 percentage points respectively) compared to Monday appointments (Supplementary Table S6). Missing data was associated with CCG.

Of the 31,154 booked appointments with complete data, 46.30% of booked appointments were pre-booked, 82.81% were for a GP, and 12.25% resulted in a patient not attending (Supplementary Table S7). 58.55% of booked appointments were made by female patients. 56.06% of users were aged under 39 (71.88% under 49). Estimates from our second set of analyses concerning patient demographics for types of appointments booked are contained in Table 4. Across all models there were significant CCG effects; this is likely to reflect variations in service delivery due to the variations in relative scale of booking type and appointment type (Table 2).

For peer review only - http://bmjopen.bmj.com/site/about/guidelines.xhtml

Table 4 Patient demographics by booking and appointment type

	% same-day	% pre-booked	Pre-booked v Same-day^ (95% CI)	% Nurse	% GP	GP v nurse^^ (95% CI)
**Gender**						
**Male (base category)**	57.69	42.31		18.40	81.60	
**Female**	57.60	42.40	-0.66 (-1.08, -0.25)	23.28	76.72	-4.24 (-9.12, 0.63)
**Age band (years)**						
**0-9 (base category)**	72.45	27.55		2.98	97.02	
**10-19**	58.19	41.81	12.94 (10.78, 15.10)	13.03	86.97	-19.25 (-35.52, -2.98)
**20-29**	55.29	44.71	14.63 (12.13, 17.13)	19.68	80.32	-25.85 (-41.56, -10.15)
**30-39**	55.32	44.68	14.64 (13.09, 16.19)	23.14	76.86	-28.90 (-42.78, -15.02)
**40-49**	55.63	44.37	14.86 (12.52, 17.20)	25.56	74.44	-31.54 (-47.20, -15.88)
**50-59**	53.41	46.59	17.45 (15.84, 19.07)	25.82	74.18	-31.69 (-48.53, -14.84)
**60-69**	58.24	41.76	13.15 (11.68, 14.63)	27.79	72.21	-34.18 (-49.83, -18.53)
**70-79**	58.20	41.80	12.62 (8.81, 16.43)	30.65	69.35	-37.06 (-52.49, -21.63)
**80-89**	55.71	44.29	14.53 (10.57, 18.48)	37.47	62.53	-41.04 (-56.40, -25.67)
**90+**	68.42	31.58	7.50 (-15.21, 30.22)	41.67	58.33	-42.45 (-65.41, -19.49)
**Day of week**						
**Monday (base category)**	79.25	20.75		19.07	80.93	
**Tuesday**	58.56	41.44	20.33 (16.47, 24.18)	21.53	78.47	-1.60 (-2.15, -1.05)
**Wednesday**	52.38	47.62	27.27 (24.10, 30.44)	17.62	82.38	2.28 (1.71, 2.84)
**Thursday**	53.16	46.84	24.82 (17.48, 32.16)	22.11	77.89	-2.69 (-3.59, -1.78)
**Friday**	52.60	47.40	25.28 (20.63, 29.93)	18.43	81.57	1.36 (0.51, 2.21)
**Saturday**	55.31	44.69	24.06 (14.95, 33.17)	23.26	76.74	1.59 (1.51, 1.67)
**Sunday**	55.53	44.47	25.05 (10.20, 39.89)	21.28	78.72	0.05 (-0.06, 0.16)
**Appointment type**						
**Nurse (base category)**	47.51	52.49				
**GP**	59.50	42.36	-12.76 (-15.16, -10.36)			
**DNA status**						
**Attended (base category)**	60.00	40.00		21.56	78.44	
**DNA**	40.41	59.59	18.85 (16.88, 20.83)	19.27	80.73	2.13 (-0.17, 4.44)
**Booking type**						
**Same-day (base category)**				15.99	84.01	
**Pre-booked**				27.28	78.73	-7.27 (-7.67, -6.87)
**CCG scheme**						
**CCG2**	58.12	41.88	-15.39 (-16.18, -14.60)	19.57	80.43	15.45 (11.33, 19.57)
**CCG3**	74.44	25.56	-27.73 (-28.43, -27.03)			-
**CCG4**			-			Base
**CCG5**	46.00	54.00	Base			-

For peer review only

For peer review only - http://bmjopen.bmj.com/site/about/guidelines.xhtml

**Sample size**			29,029			25,176

Probit regression of appointment against day of week, calendar month, appointment characteristics, patient age and gender, and CCG scheme. Month estimates omitted from the output but available upon request.
Estimates are presented as average marginal effects which give the percentage point effect of the variable relative to the base category.
^CCG5 had 100% pre-booked and GP appointments (n=2,183, 2,125 with complete data) and was omitted from this analysis giving a sample of 3,154-2,125=29,029.
^^CCG4 had 100% GP appointments (n=4,463, 3,928 with complete data) and CCG3 had 100% GP appointments (n-2,050, 2,050 with complete data) and both were omitted from this analysis giving a sample of 31,154-3,928-2,050=25,176.

42.31% of appointments for males patients were for pre-booked appointments, there was evidence that females were relatively less likely to pre-book (-0.66 percentage points, 95%CI: -1.08 to -0.25). A greater percentage of appointments were pre-booked across age groups 10 to 19 to 80 to 89 in comparison to age group 0 to 9 years and 90 years and over. The probability that an appointment was pre-booked compared to same-day was greater for each day of the week compared to Monday. GP appointments were less likely to be pre-booked in comparison to nurse appointments (-12.76 percentage points, 95%CI: -15.16 to -10.36). DNA appointments were more likely to be pre-booked than attended appointments (18.85 percentage points, 95%CI: 16.88 to 20.83)).

81.60% of male appointments were with a GP, there was no significant difference found for females (-4.24 percentage points compared to males, 95%CI: -9.12 to 0.63). The proportion of appointments that were with a GP decreased monotonically with age group. The proportion of appointments with a GP compared to a nurse varied by day of week and by whether the appointment was pre-booked or same-day (pre-booked appointments were less likely to be with a GP). There was no significant difference in the proportion of appointments with a GP or nurse by DNA status (2.1 percentage points compared to patients attending (95% CI: -0.2 to 4.4)).

Comparisons of extended access appointment to core hour users

In total, 84.88% of appointment users were aged under 60 years of age (Supplementary Table S8). Patients booking and attending extended access appointments were compared to patients reporting attendance at their practice within the past year in the GPPS (Figure 4). Patients booking and attending extended access appointments tended to be younger in comparison to core hour patients with a more equal distribution of users aged between 20 to 59 compared to GPPS core hour users. For female users, the peak in the distribution of extended access users occured at age 20-29, compared to 45 to 54 for users in the GPPS. As the age bands do not align in the datasets, these differences could not be tested, and comparisons should be treated with caution. To better reflect the sampling frame of the GPPS (patients aged 18 and over), Table 5 provides the percentages of users in extended access appointments from age 20 plus. 81.00% of extended access appointments users aged 20 plus were under 60 years, whilst the equivalent percentage in the GPPS data lies above age 65.

For peer review only

<Figure 4 here>

Table 5 Demographic distributions of extended access users aged 20+ and GP Patient Survey users

	Males			Females			All
	Frequency	%	Cumulative %	Frequency	%	Cumulative %	Cumulative %
**Extended access**							
**Age**							
**20-29**	1,540	17.55	17.55	3,129	24.11	24.11	21.46
**30-39**	1,739	19.82	37.38	2,758	21.25	45.36	42.14
**40-49**	1,795	20.46	57.84	2,603	20.06	65.41	62.36
**50-59**	1,807	20.60	78.43	2,248	17.32	82.73	81.00
**60-69**	1,187	13.53	91.96	1,335	10.29	93.02	92.59
**70-79**	494	5.63	97.59	649	5.00	98.02	97.85
**80-89**	199	2.27	99.86	228	1.76	99.78	99.81
**90+**	12	0.14	100.00	29	0.22	100.00	100.00
**Total**	8,773	100.00		12,979	100.00		
**GP Patient Survey users***							
**Age**							
**18-24**	298	8.45	8.45	514	10.53	10.53	9.55
**25-34**	472	13.39	21.83	798	16.35	26.88	24.51
**35-44**	618	17.52	39.36	812	16.64	43.52	41.56
**45-54**	720	20.43	59.79	912	18.69	62.21	61.07
**55-64**	608	17.26	77.04	691	14.15	76.36	76.68
**65-74**	493	14.00	91.04	578	11.85	88.21	89.54
**75-84**	251	7.11	98.15	383	7.85	96.06	97.04
**85+**	65	1.85	100.00	192	3.94	100.00	100.00
**Total**	3,524	100.00		4,881			

*GPPS weighted responses, rounded to nearest whole number

DISCUSSION

Principal findings

We found evidence of spare capacity in the extended access service. However, uptake improved over the calendar year, suggesting an embedding of the service over time. Sunday uptake was the lowest of all the days of the week, but this also increased over the year. There were variations in patient demographics for particular types of extended access services. Pre-booked appointments were relatively less likely to be female patients, and more likely to be amongst age groups 0 to 9 and 90 years and over in comparison to same-day appointments. GP appointments were increasingly less likely with age in comparison to nurse appointments. In comparison to core hour patients, we found some evidence that extended access appointment users were of a younger age relative to patients using primary care services before the scheme. The most obvious difference was found in females aged 20-29 who accounted for almost a quarter of extended access appointments users (aged 20+).

For peer review only

For peer review only - http://bmjopen.bmj.com/site/about/guidelines.xhtml

Strengths and weaknesses

The study has a number of strengths and weaknesses. Strengths lie in the unique dataset which enabled a detailed look at uptake and users of the service and how this varied with types of appointments provided. Missing data was small and unassociated with particular calendar months or hubs but was associated with CCGs and a small effect of day of week. In addition, our results are focussed on several Greater Manchester schemes offering variation in delivery which enables comparisons of different service models. However, weaknesses are that the findings may not be generalizable to other areas, we were unable to obtain patient deprivation/ethnicity characteristics to enrich understanding of service use. The GPPS comparison is particularly flawed because children are not included in the survey and age bands did not match with those obtained for appointment data. Finally, as noted above, access depended on a range of factors, and without further details on CCG provision related to awareness campaigns and buy-in from providers, we can only distinguish such effects by the CCG fixed-effects adjustment factor in our models.

Implications

The presence of spare capacity for a service seeking to improve access to meet excess demand in core hours raises the question of whether or not extended access appointments really are improving access to primary care. However, of the appointments that were used, our findings suggest that the service may be either meeting previously unmet need for younger age groups, or diverting demand for core hours for this age group, thereby potentially reducing pressure in core hours.

The reasoning behind spare capacity in the scheme may be informed by three domains of access: availability, acceptability, affordability.[14] These domains of access highlight the complex contributory factors at play in access, involving both patient and practitioner. The extended access schemes provide additional *availability* in the sense of new appointments. However, new appointments alone may not be sufficient to improve access, should patients or practitioners not be prepared to *accept* the service (this could be due to cultural factors and/or patient preferences for example). Identification is needed of whether poor uptake is due to a lack of patient demand or, for of particular concern in areas where practice referrals are made, whether a lack of receptionist/practitioner buy-in is influencing patient (and receptionist) awareness of the service (see for example: Healthwatch Manchester, 2017[15]). Policy needs to consider these additional aspects of access to encourage uptake and adapt provision accordingly.

For peer review only

Our findings shed light on a number of aspects relevant for determining cost-effectiveness of the extended access service. First, assuming core hours remain at capacity, it is likely that extended access services will result in some positive measure of benefit to patients. Second, a large proportion of extended appointments are pre-booked, suggesting a large amount of activity in the service is for non-urgent care. As such, existing evaluations, that focus on emergency care implications, paint only a partial picture of the impacts of extended access. Third, providers seeking to determine the types of services available under extended access schemes may consider several factors to more efficiently provide the service in relation to our results : (i) whilst uptake was lowest on a Sunday, the volume of booked appointments was similar to those during the week, this suggests uptake on a Sunday can be improved by reducing the volume of Sunday appointments (ii) a completely pre-bookable service may reduce access for the very young, elderly, and for females (although pre-bookable appointments were more likely to result in DNAs). (iii) nurse appointments may help address issues of staffing, since DNAs were no greater for this type of appointment and did not differ on the basis of gender uptake. However, nurse appointments were used increasingly with age suggesting a potential need for a GP service to address the needs of younger age patients (who dominate service use).

The evaluation of the national pilots have found uptake of 71% with reportedly lower uptake on weekends, particularly on Sundays.[8] A more detailed evaluation of three schemes under the PMCF pilots found uptake varied by type of service (same day or pre-bookable) available.[11] Uptake was higher in a scheme providing pre-bookable and same day appointments (87% compared to 22% and 57%). In the scheme providing pre-bookable and same day appointments during weekends only (three practices with one hub) uptake was reported as 87% on Saturdays and 78% on Sundays. The demographics comprised 3% aged under 5, and 57% aged between 20 and 65. The findings in this paper also found pre-bookable appointments had a higher uptake (for appointments attended: 54.55% compared to 45.44% (Table 2)). We also found uptake was lower on Sundays (also in line with the national evaluation). However, in contrast we found age group 20-29 to be the most prevalent group of patients using the service (17.08%) and a higher proportion of patients from the 20-69 age group (73.68% compared to 57% aged 20-65). These differences may be due to differences in the patient population, breadth of week covered or scale of the schemes (the relevant scheme evaluated by Windrum et al (2017)[11] was much smaller with three practices and one hub). Evaluations of patient demographics using extended access appointments for urgent care in Sheffield, UK, found users were predominantly female (60.0%) and aged under 60 (85.5%), with 19.05% aged under 5 and 29.35% aged under 16 and whilst ethnicity data had a high proportion of missing data, deprivation was captured finding users from deprived areas dominated use.[12] These findings may reflect the

For peer review only - http://bmjopen.bmj.com/site/about/guidelines.xhtml

urgent care service delivered in comparison to the current study which offered both same-day and pre-bookable appointments.

Future work

We have not been able to identify implications for core hours or assess whether deprivation or ethnicity are a leading factor of uptake. Further research is needed to investigate the impacts on demand for core hours services and for deprived populations to shed light on potential implications for access.

Given the pilot nature of these schemes, uptake may have also reflected supply-side buy-in, and reservations concerning promotion of the schemes may have arisen due to uncertainty in future funding. Future evaluations of uptake, once clarity is provided on long-term securement of the extended access service, may help identify the scale of this possibility.

Competing interests statement:
All authors have completed the ICMJE uniform disclosure form at www.icmje.org/coi_disclosure.pdf and have the following competing interests: funding support from NIHR and from Greater Manchester Health and Social Care Partnership for this work; WW reports grant funding for other work from the Department of Health Policy Research Programme and NIHR. No other relationships or activities that could appear to have influenced the submitted work are declared.

Contributors:
WW is guarantor for the work. WW cleaned the data, conceived and conducted the analysis, interpreted the data and composed the first draft of the work.
LA critically revised the manuscript, participated in the design of the study, and approved the final version to be published.
PN contributed to interpretation of data, revised the initial and subsequent drafts of the paper critically for content and approved the final version to be published.
CO cleaned the activity data in preparation for the analyses, assisted in interpretation of the findings, and approved the final version to be published.
NR contributed to interpretation of data, revised earlier drafts of the paper, and approved the final version to be published.
KR contributed to interpretation of data, revised the initial and subsequent drafts of the paper critically for content and approved the final version to be published.
DH designed and led the overall project, was directly involved in interpretation of data and approved the final version to be published.
All authors had full access to all of the data (including statistical reports and tables) in the study and can take responsibility for the integrity of the data and the accuracy of the data analysis.

Patient consent:
Patient consent was not required as the study reports no identifiable patient data.

For peer review only

Ethics statement:

The study was reviewed by the University of Manchester's internal University Research Ethics Committee and approved under the low risk procedure and thus did not require UREC approval (ID: AMBS/16/05). The study involves anonymised administrative data and did not impact on the type of care patients received, consent by patients using the service was not required.

Funding statement:

This project was funded by the National Institute for Health Research Collaboration for Leadership in Applied Health Research and Care (NIHR CLAHRC) Greater Manchester and the Greater Manchester Health and Social Care Partnership. The NIHR CLAHRC Greater Manchester is a partnership between providers and commissioners from the NHS, industry and the third sector, as well as clinical and research staff from the University of Manchester. The views expressed in this article are those of the author(s) and not necessarily those of the NHS, NIHR or the Department of Health and Social Care.

Transparency declaration:

WW affirms that the manuscript is an honest, accurate, and transparent account of the study being reported; that no important aspects of the study have been omitted; and that any discrepancies from the study as planned have been explained.

Copyright statement:

The Corresponding Author has the right to grant on behalf of all authors and does grant on behalf of all authors, *a worldwide licence* to the Publishers and its licensees in perpetuity, in all forms, formats and media (whether known now or created in the future), to i) publish, reproduce, distribute, display and store the Contribution, ii) translate the Contribution into other languages, create adaptations, reprints, include within collections and create summaries, extracts and/or, abstracts of the Contribution, iii) create any other derivative work(s) based on the Contribution, iv) to exploit all subsidiary rights in the Contribution, v) the inclusion of electronic links from the Contribution to third party material where-ever it may be located; and, vi) licence any third party to do any or all of the above.

Data sharing statement:

Appointment data: No additional data available.

GP Patient Survey: The GP Patient Survey data are available at https://gp-patient.co.uk/SurveysAndReports

Reporting statement:

A STROBE checklist for observational studies has been adhered to with checklist provided as supplementary material.

For peer review only

References:

[1] World Health Organisation. The world health report: health systems financing: the path to universal coverage. [cited 2010]. Available from: http://www.who.int/whr/2010/en/ [Accessed 01/11/2018]

[2] Schoen C, Osborn R, Doty MM, Bishop M, Peugh J, Murukutla N. Towards Higher-Performance Health Systems: Adults' Health Care Experiences In Seven Countries, 2007. *Health Affairs* 2007;26(6):w717-34.

[3] Schoen C, Osborn R, Squires D, Doty MM. Access, Affordability, And Insurance Complexity Are Often Worse In The United States Compared To Ten Other Countries. *Health Affairs* 2013;32(12):2205-2215.

[4] Kraaijvanger N, van Leeuwen H, Rijpsma D, Edwards M. Motives for self-referral to the emergency department: a systematic review of the literature. *BMC Health Serv Res* 2016;16:685

[5] NHS England. General Practice Forward View. [cited 2016 April]. Available from: https://www.england.nhs.uk/wp-content/uploads/2016/04/gpfv.pdf [Accessed 01/11/2018]

[6] NIHR CLAHRC. NHS Greater Manchester Primary Care Demonstrators Evaluation. [cited 2015 June]. Available from: https://www.research.manchester.ac.uk/portal/files/32297227/FULL_TEXT.PDF [Accessed 01/11/2018]

[7] Greater Manchester Combined Authority. Delivering Integrated Care across Greater Manchester: The Primary Care Contribution. Our Primary Care Strategy 2016-2021. [cited 2016]. Available from: http://www.gmhsc.org.uk/assets/GMHSC-Partnership-Primary-Care-Strategy.pdf [Accessed 01/11/2018]

[8] MacDonald, SQW. Prime Minister's Challenge Fund: Improving Access to General Practice: Second Evaluation Report to September 2015. [cited 2016]. Available from: https://www.england.nhs.uk/wp-content/uploads/2016/10/gp-access-fund-nat-eval-wave1-sml.pdf [Accessed 01/11/2018]

[9] Dalton P, Pathania V. Can increased primary care access reduce demand for emergency care? Evidence from England's 7-day GP opening. *J Health Econ* 2016;49:193-208.

[10] Whittaker W, Anselmi L, Kristensen S, et al. Associations between extending access to primary care and emergency department visits: a difference-in-differences analysis. *PLoS Med* 2016;13(9):e1002113

[11] Windrum P, Guinery J, Siebert P, et al. CHILL Evaluation of 7-Day GP Access. [cited 2017 Jan 1]. Available from: https://www.nottingham.ac.uk/business/businesscentres/chill/documents/chill-7-day-gp-access-report.pdf [Accessed 01/11/2018]

[12] Kelly SJ, Piercy H, Ibbotson R, et al. Who attends out-of-hours general practice appointments? Analysis of a patient cohort accessing new out-of-hours units. *BMJ Open* 2018. doi:10.1136/bmjopen-2017-020308

For peer review only

[13] Ipsos MORI. Technical Annex for the GP Patient Survey 2012-2013 Annual Report. [cited 2013 June 13]. Available from: https://gp-patient.co.uk/downloads/archive/2013/June/June%202013%20Technical%20Annex.pdf [Accessed 01/11/2018]

[14] McIntyre D, Thiede M, Birch S. Access as a policy-relevant concept in low- and middle-income countries. *Health Econ Policy Law* 2009;4(2):179-193

[15] Healthwatch Manchester. Week spot? Review of Access to the 7 Day GP Service. [cited 2017 November]. Available from: https://d2jlsms9zhgfok.cloudfront.net/2017/12/07142053/Week-Spot-Access-to-7-Day-GP-Service-Review.pdf [Accessed 01/11/2018]

For peer review only

Figures

Figure 1 Flow chart of population coverage

Figure 2 Appointments used, DNA and not booked by month, all schemes

Excludes CCG1 activity

Figure 3 Appointments used, DNA and not booked by day of week, all schemes

Excludes CCG1 activity

Figure 4 Profile of extended appointment users and core hours users, by age and gender

For peer review only - http://bmjopen.bmj.com/site/about/guidelines.xhtml

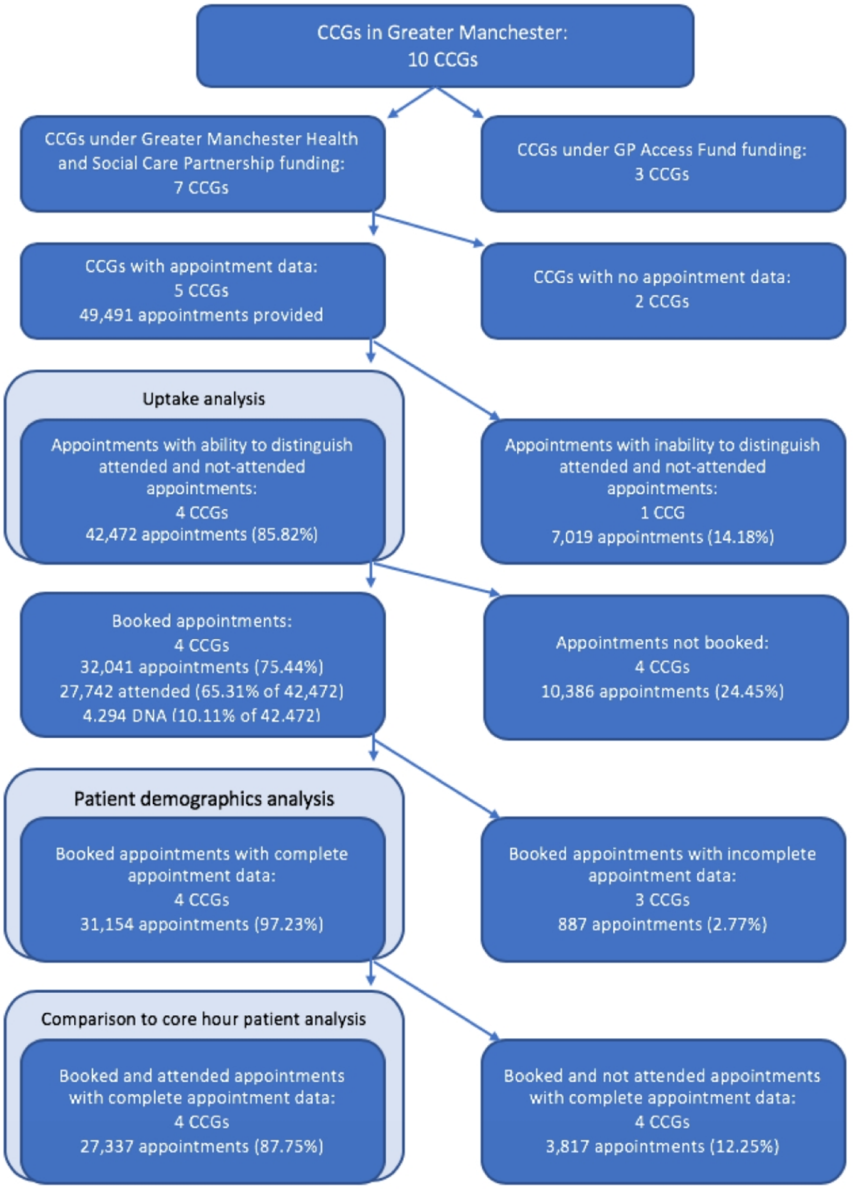

Figure 1 Flow chart of population coverage

112x156mm (300 x 300 DPI)

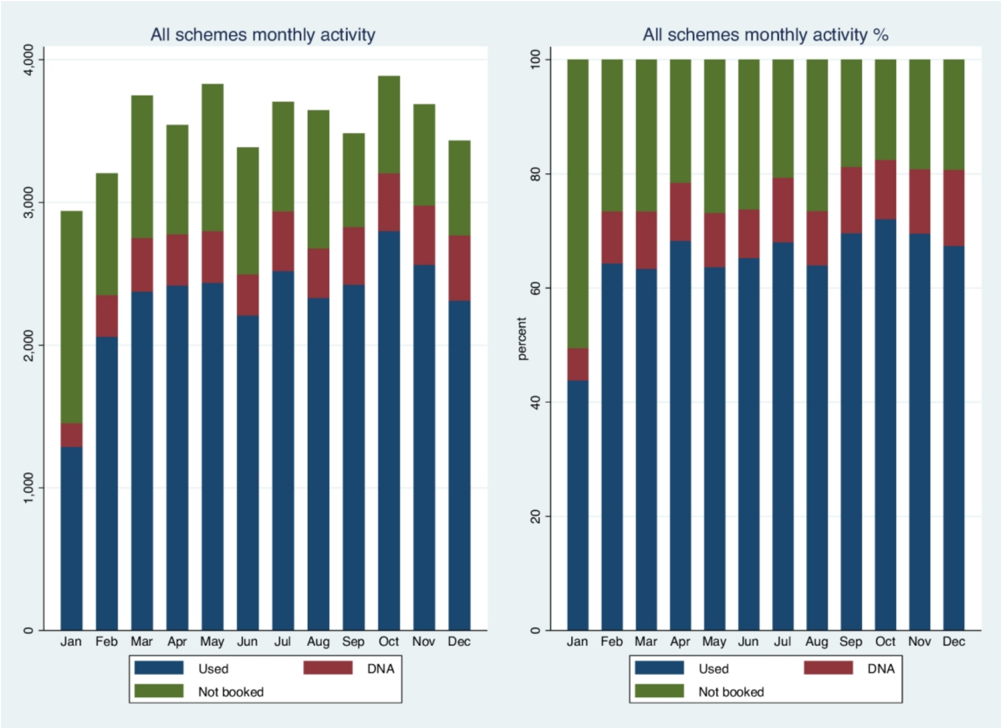

Figure 2 Volume and percentage of appointments used, DNA and not booked by month, all schemes

138x101mm (300 x 300 DPI)

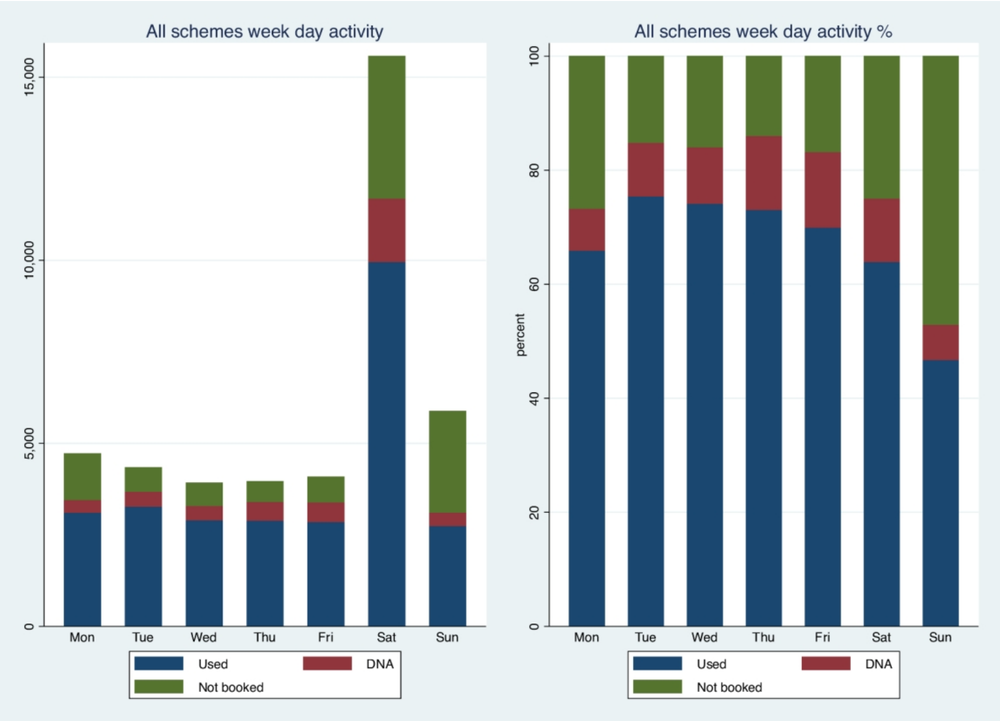

Figure 3 Volume and percentage of appointments used, DNA and not booked by day of week, all schemes

142x102mm (300 x 300 DPI)

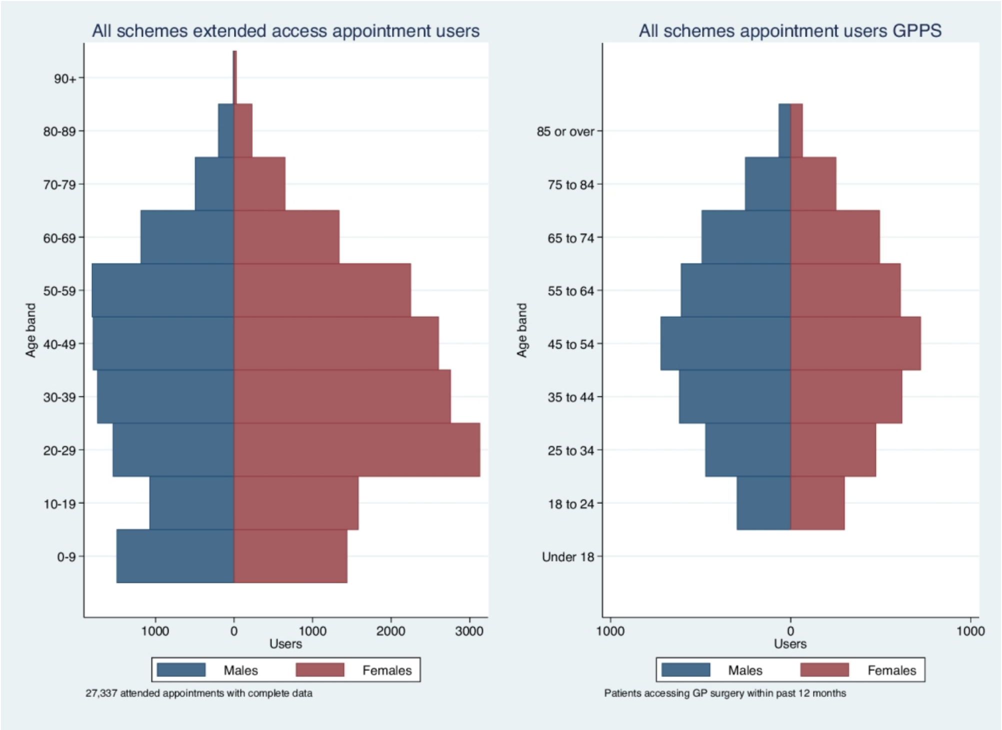

Figure 4 Profile of extended appointment users and core hours users, by age and gender

139x101mm (300 x 300 DPI)

MANCHESTER 1824
The University of Manchester

NHS
National Institute for Health Research
Collaboration for Leadership in Applied Health Research and Care (CLAHRC) for Greater Manchester

GM PRIMARY CARE 7 DAY ACCESS EVALUATION: RESEARCH PROTOCOL

Background

In June 2014, the 12 Greater Manchester Clinical Commissioning Groups (CCGs) agreed to the Healthier Together Primary Care Standard that by the end of 2015, “everyone in Greater Manchester who needs medical help will have same-day access to primary care services, supported by diagnostic tests, seven days a week”[1]. At the same time seven day access has been made one of eight early implementation priorities under the Greater Manchester devolution agreement[2].

This protocol describes the proposed evaluation of seven day access to primary care to be conducted on behalf of NHS England (Greater Manchester) by CLAHRC Greater Manchester. CLAHRC GM previously conducted the evaluation of the NHS England Primary Care Demonstrator services, and the proposed evaluation seeks to build on the strengths of the previous evaluation. The proposed mixed methods evaluation will combine a focus on **activity**, **process** and **outcomes** in order to provide a comprehensive assessment of the implementation and impact of the new services. The evaluation will focus primarily on the 7 CCG areas receiving support from NHS England as part of the devolution agreement. The remaining 5 CCG areas have pre-existing funding and support to provide 7 day access via either the Wave 1 Prime Minister’s Challenge Fund (PMCF) or Wave 2 General Practice Access Fund (GPAF) (see **Table 1**). The majority of these are already ‘live’; therefore while they will not make up part of the formal evaluation, intelligence gathered in the PMCF/GPAF areas can be used to support learning and development in the seven areas where 7-day primary care access is now being introduced.

Table 1: 7-Day Access Areas in Greater Manchester

PMCF/GPAF Areas (not evaluated)	New 7-Day Access Areas (to be evaluated)
Bury	Bolton
Wigan	Heywood, Middleton and Rochdale
North Manchester	Oldham
Central Manchester	Salford
South Manchester	Stockport
	Trafford
	Tameside & Glossop

[1] https://healthiertogethergm.nhs.uk/what-healthier-together/primary-care/
[2] http://www.gmhealthandsocialcaredevo.org.uk/assets/GM-Strategic-Plan-Final.pdf p14

For peer review only

The 7 CCGs submitted their initial plans for the delivery of seven day primary care access to NHS England in mid-2015. The schemes were expected to start in December 2015, although some have indicated a later start date. All areas are adopting a ‘hub’ approach to providing additional hours, in which the CCG area is split into geographic areas, and the extended access services are provided from a ‘hub’ location in each area. CCG plans submitted to NHS England promise 43 hubs (including PMCF/GPAF hubs) operational by December 2015 with a further 9 hubs live in 2016 (see figure 1).

Figure 1

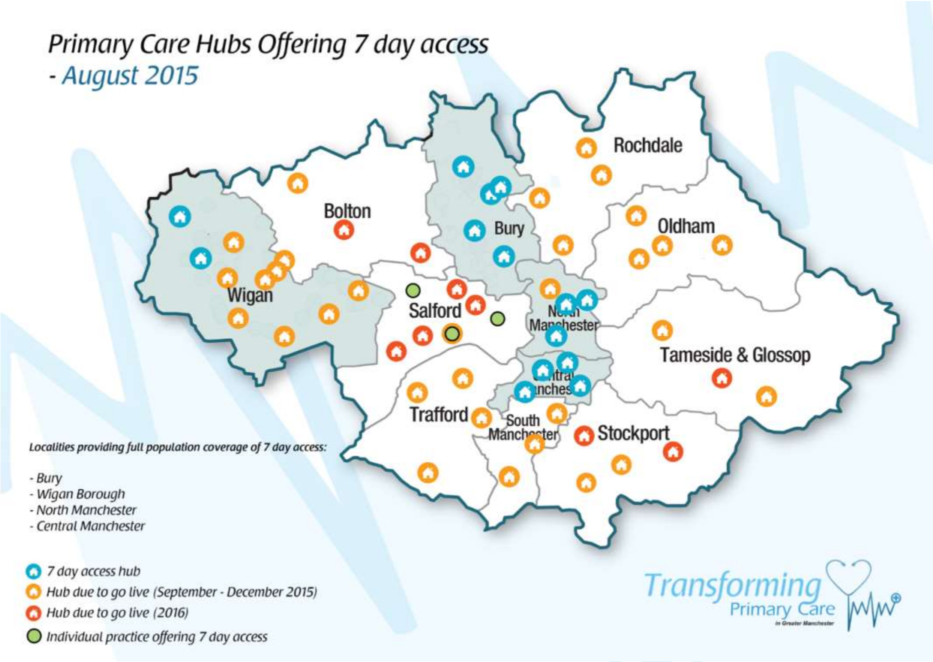

Description of Evaluation

The evaluation seeks to understand the implementation and impact of seven day primary care services across Greater Manchester. The evaluation will combine qualitative and quantitative analysis, supported by a measure of activity across the 7 areas to be evaluated.

The evaluation will seek to answer the following questions (see **Table 2**);

Table 2: Evaluation Questions and Modes of Evaluation

1. What new activity is associated with seven day primary care services in each area, and at what cost?	**ACTIVITY**
2. How are these new services utilised by patients? What is the nature of demand for these new services?	
3. How is the uptake of 7 day primary care affected by the socio-economic characteristics of the patient population?	
4. What organisational and operational issues arise in the implementation of 7 day access to primary care, and how have areas addressed these issues?	**PROCESS**

5. What is the impact of 7 day access to primary care on;<br>a. Identified prevalence of disease,<br>b. Attendances/admissions at Accident & Emergency,<br>c. Use of Out-of-Hours services?	**OUTCOME**
6. How has the change in access been received by<br>a. Patients,<br>b. Staff in primary care?	

As is indicated above, questions 1-3 relate to the **activity** analysis (i.e. what was done). Question 4 relates to the **process** analysis (i.e. how was it done). Questions 5 and 6 relate to the **outcome** analysis (i.e. what was the impact of the new activity).

It should be emphasised that the ability to answer these questions depends directly on the availability of relevant data. It is important that data can be captured by the evaluation team in a timely manner, can be presented in a format which allows for analysis, and that this can be done subject to current information governance regulation. Some data is routinely collected, other data can be collected by the evaluation team with the facilitation of the commissioners and providers in each area, and some can only be produced by the providers themselves (see **Table 3**);

Table 3: Evaluation Questions and Data Sources

1. What new activity is associated with seven day primary care services in each area, and at what cost?	**Activity data supplied by each area**
2. How are these new services utilised by patients? What is the nature of demand for these new services?	**Activity data supplied by each area**
3. How is the uptake of 7 day primary care affected by the socio-economic characteristics of the patient population?	**Public data combined with activity data.**
4. What organisational and operational issues arise in the implementation of 7 day access to primary care, and how have areas addressed these issues?	**Evaluation generated data. Source: qualitative interviews.**
5. What is the impact of 7 day access to primary care on;<br>a. Identified prevalence of disease,<br>b. Attendances/admissions at Accident & Emergency,<br>c. Use of Out-of-Hours services?	**Routinely collected data. Source:**<br>**(a) QOF, via CCGs,**<br>**(b) SUS, via AHSN,**<br>**(c) OOH activity, supplied by NHS England/CCGs.**
6. How has the change in access been received by<br>a. Patients,<br>b. Staff in primary care?	**Evaluation generated data. Source:**<br>**(a) GPPS, via HSCIC.**<br>**(b) staff survey administered by evaluation team via CCGs.**

The evaluation period will begin at the point at which the NHS England hubs go live on 1st January 2016 and continue for 12 months.

For peer review only

The evaluation has conducted preliminary work to establish reliable sources of data, in discussion with NHS England (Greater Manchester) and the Greater Manchester Academic Health Science Network (AHSN). The provision of activity data from each area has not yet been agreed: - this research protocol document is intended to provide information and thereby initiate the process of agreeing the content and process of activity capture in dialogue with each area.

Further details on the data capture and analysis is provided below.

1. Activity Capture

The activity capture in the present evaluation has been designed in order to minimise the additional workload that data extraction places on individual sites, while also seeking to ensure that the data collected can be collected reliably and consistently across all 7 areas.

To this end, a minimum data set (**Table 4** below) has been defined, to be agreed with all participating CCGs. Data should be reported according to a monthly schedule, which will support formative evaluation and shared learning across sites. Also data will be needed from NHS England/CCGs on relevant DES arrangements in each area, to establish activity baselines.

Table 4: Activity Capture Minimum Dataset

**1. Utilisation:**
a. No. of appointments available
i. By day
1. Time of appointment
ii. By discipline
iii. Planned/pre-booked vs same day/urgent
iv. Method of appointment (face-to-face/phone)
b. No. of appointments booked
i. By day
1. Time of appointment
ii. By discipline
iii. Age
iv. Gender
v. Registered GP practice
vi. Postcode
vii. Planned/pre-booked vs same day/urgent
viii. Method of appointment (face-to-face/phone)
c. No. of appointments used (Utilisation)
i. By day
1. Time of appointment
ii. By discipline
iii. Age
iv. Gender
v. Registered GP practice
vi. Postcode (first part)
vii. Planned/pre-booked vs same day/urgent
viii. Method of appointment (face-to-face/phone)

For peer review only

2. Practice-level population data:
a. Practice level population
b. Practice codes
c. Whether a GP practice or not
d. Registered patient age band
e. Deprivation of practice population
f. Gender breakdown of practice population

The aim of the activity capture evaluation is to:

1. **Assess the ability of each area to provide additional appointments i.e.**
    a. **How many additional appointments were available, by whom and by what means?**
    b. **How many additional appointments were booked, urgent, and how many resulted in DNA?**
2. **Assess the types of patients utilizing additional appointments i.e.**
    a. **Were patients attending/DNA representative of the practice's total list (in terms of age, gender, and IMD)?**

For utilisation data (Items 1a-1c, Table 3), the evaluation relies on the ability of each area to collect, collate and provide data routinely. Preliminary conversations with NHS England and some local CCGs suggest that this data should be available – however, this requires confirmation with all areas.[3]

CCG level population data (Item 2, Table 3) can be extracted by the CLAHRC evaluation team from individual practice registration data.

2. Process Evaluation

Semi-structured interviews will be conducted with senior leadership (CCG lead + GP lead in each area). 2 leads x 7 areas= 14 interviews, conducted at start and end of intervention period = 28 interviews. These will be supplemented with key informant interviews (estimated 6-7), including leads in the PMCF/GPAF areas, and, where appropriate, additional interviews with key individuals in each area.

The process evaluation will seek to understand the organisational and operational issues in the implementation of 7 day access to primary care faced in each area, and also how each area addressed these issues. A brief interview schedule can be found in **Appendix 1**.

[3] In addition, the following data would enhance the analysis by providing insight into the reasons for and nature of the additional consultations.

1. Reason for attendance
2. Outcome of consultation
    a. Discharged with advice
    b. Referral to ….. (list providers)
    c. Treatment/prescription issued
    d. Follow-up appointment
    e. Diagnostics
    f. Other

However, we recognise this may be difficult or impossible to provide without incurring substantial and unrealistic data collection costs on the areas.

For peer review only

3. Outcome Evaluation

The outcome evaluation will concentrate on four areas; (A) Health Outcomes, (B) Impact on Service Utilisation, (C) Staff Experience and (D) Patient Experience.

A. **Impact on Health Outcomes** will be primarily measured using selected QOF data on disease prevalence. QOF data on disease prevalence will be analysed to examine the effect of the intervention on the identification and management of long-term conditions. This data will be supplied by local CCGs. Data will be captured for three years prior to the intervention (2013-16) and at two points during the intervention itself. A full list of QOF data categories can be found in **Appendix 2**.

B. **Impact on Service Utilisation** will be measured using routinely-collected SUS data on hospital activity, plus activity data from relevant out-of-hours providers supplied by NHS England/CCGs.

SUS data will be extracted for all areas of Greater Manchester (seven 7 day access sites and 5 PMCF/GPAF areas) on a quarterly basis. The specific items to be analysed can be found in **Appendix 3.**

The aim of the service utilisation evaluation is to:

(1). Assess the effects of additional appointments on hospital activity:

a) A&E attendances:

i) Minor patient-initiated A&E attendances

ii) Total A&E attendances

iii) Waiting time to assessment, treatment, and total A&E duration

b) Admissions:

i) Total admissions

ii) Admissions for ambulatory care-sensitive conditions

(2). Assess whether (1) varies by dosage/volume of additional appointments per 1000

(3). Assess whether (1) varies by additional appointments' users characteristics (age, gender, deprivation)

(1) will be measured using SUS data for the intervention period (quarters 1 to 4 of 2016) compared to 2015 i.e. a before and after analysis. All activity will be measured at the practice level, by quarter, and per 1000 of a practices registered population. A pooled cross-sectional dataset will be used. For each type of hospital activity the dependent variable will be regressed on binary year dummies. The estimated coefficient on the intervention (2016) dummy provide a statistical test for significant differences in the intervention period. Count models will be estimated that recognise and incorporate the over-dispersed distribution of hospital activity. Sensitivity analysis using alternative model specifications will be carried out.

The diagnosis code (DIAG_NN) in the admissions data will be used to identify ambulatory care sensitive conditions as follows:

- Ambulatory care-sensitive conditions admissions (ICD)
- Vaccine-preventable:

  Influenza and pneumonia; Other vaccine-preventable conditions
- Chronic:

  Asthma; Congestive heart failure; Diabetes complications; Chronic obstructive pulmonary disease (COPD); Angina; Iron-deficiency anaemia; Hypertension; Nutritional deficiencies

For peer review only

- Acute:

  Dehydration and gastroenteritis; Pyelonephritis; Perforated/bleeding ulcer; Cellulitis; Pelvic inflammatory disease; Ear, nose and throat infections; Dental conditions; Convulsions and epilepsy; Gangrene

Where available, the difference in hospital activity will be compared against comparator practices and tests for significant differences measured using a *controlled* before and after analysis framework (difference-in-differences).

Items (2) and (3) depend on the quality and coverage of the activity capture. Using data from the activity capture we will generate a measure of intensity of treatment (dosage - additional appointments per 1000 of a practice's registered population) and replicate (1) by dosage. Information on the types of patients using additional appointments from the activity capture will be used to identify the groups where hospital activity may be most concentrated. For example, if additional appointments are concentrated amongst females then it may be that female hospital activity is where the impacts of additional appointments may be seen.

C. **Staff Experience** The evaluation team will develop an online survey to capture information on the experience of primary care staff. This administration of this survey will require the cooperation of local provider organisations.

D. **Patient Experience** We will analyse General Practice Patient Survey (GPPS) data, adopting a difference-in-difference (DiD) methodology, focusing on survey items addressing overall quality of care and convenience, subject to data availability (see note below). Individual CCGs may choose to conduct their own patient surveys, and guidance can be provided by the evaluation team on the administration of these.[4]

[4] Note: While the relevant period of GPPS data will be July-September 2016, which should be released in early 2017. If the release of this data is delayed by HSCIC, to avoid delaying report submission at end of March 2017 then patient experience analysis may have to be supplied latter as a separate addendum to the report.

For peer review only

Evaluation Timeline

	JAN 16	FEB 16	MAR 16	APR 16	MAY 16	JUN 16	JUL 16	AUG 16	SEP 16	OCT 16	NOV 16	DEC 16	JAN 17	FEB 17	MAR 17
Research Approvals	■	■	■	■	■	■									
Activity Capture						■	■	■	■	■	■	■			
Process Evaluation						■	■	■			■	■			
Outcome Evaluation						■			■			■	■	■	
Patient Experience													■	■	
Staff Experience									■	■	■				
Findings (Interim and Final)										■					■

This timeline assumes that;

- The minimum activity dataset can be provided on a monthly basis by CCGs.
- Information on relevant DES activity can be supplied by NHS England/CCGs.
- The evaluation team has access to key individuals in each area for interviews.
- CCGs are able to provide selected QOF data on disease prevalence.
- The AHSN is able to supply regular extracts of SUS data in the appropriate format.
- NHSE is able to supply data on OOH activity in the appropriate format.
- CCGs are able to assist with the dissemination of an online staff survey.
- GP Patient Survey results are released by HSCIC in a timely manner.

For peer review only

Appendix 1

Interview Schedule

1. Describe the primary care access arrangements in your area outside standard hours prior to this initiative, and describe the 7-day access arrangements in place now.
2. What is your role in delivering this change?
3. What is/was required to establish 7-day access to primary care in your locality?
4. What steps have been taken so far?
5. How have you communicated the changes to patients?
6. What challenges have been encountered (IT, IG, communications and engagement, workforce, finance, infrastructure)?
7. How have you tackled these challenges?
8. What do you expect will be the impact of this change in access (on patients, staff and other parts of the health and social care system)?
9. How would you measure 'success' in this change?
10. How sustainable are the changes made in your area?

For peer review only

Appendix 2

AST001. The contractor establishes and maintains a register of patients with asthma, excluding patients with asthma who have been prescribed no asthma-related drugs in the preceding 12 months.

AF001. The contractor establishes and maintains a register of patients with atrial fibrillation.

CAN001. The contractor establishes and maintains a register of all cancer patients defined as a "register of patients with a diagnosis of cancer excluding non-melanotic skin cancers diagnosed on or after 1 April 2003".

CKD001. The contractor establishes and maintains a register of patients aged 18 or over with CKD (US National Kidney Foundation: Stage 3 to 5 CKD).

COPD001. The contractor establishes and maintains a register of patients with COPD.

DEM001. The contractor establishes and maintains a register of patients diagnosed with dementia.

DEP003. The percentage of patients aged 18 or over with a new diagnosis of depression in the preceding 1 April to 31 March, who have been reviewed not earlier than 10 days after and not later than 56 days after the date of diagnosis.

DM017. The contractor establishes and maintains a register of all patients aged 17 or over with diabetes mellitus, which specifies the type of diabetes where a diagnosis has been confirmed.

EP001. The contractor establishes and maintains a register of patients aged 18 or over receiving drug treatment for epilepsy.

HF001. The contractor establishes and maintains a register of patients with heart failure.

HYP001. The contractor establishes and maintains a register of patients with established hypertension.

LD003. The contractor establishes and maintains a register of patients with learning disabilities.

MH001. The contractor establishes and maintains a register of patients with schizophrenia, bipolar affective disorder and other psychoses and other patients on lithium therapy.

OST004. The contractor establishes and maintains a register of patients: 1. Aged 50 or over and who have not attained the age of 75 with a record of a fragility fracture on or after 1 April 2012 and a diagnosis of osteoporosis confirmed on DXA scan, and 2. Aged 75 or over with a record of a fragility fracture on or after 1 April 2014 and a diagnosis of osteoporosis.

PC001. The contractor establishes and maintains a register of all patients in need of palliative care/support irrespective of age.

PAD001. The contractor establishes and maintains a register of patients with peripheral arterial disease.

RA001. The contractor establishes and maintains a register of patients aged 16 or over with rheumatoid arthritis.

CHD001. The contractor establishes and maintains a register of patients with coronary heart disease.

STIA001. The contractor establishes and maintains a register of patients with stroke or TIA.

For peer review only

Appendix 3

Relevant SUS data to be analysed

Derived variable	SUS variable name	SUS variable code
**A&E DATA**		
**KEY BASE DATA**		
**A&E department type**	A&E department type	AEDEPTTYPE
**Provider code**	Provider code	PROCODET
**Arrival date**	A&E arrival date	ARRIVALDATE
**Arrival time**	A&E arrival time	ARRIVALTIME
**Arrival mode**	Arrival mode	AEARRIVALMODE
**Where incident took place**	Incident location type	AEINCLOCTYPE
**GP practice**	Code of GP practice	GPPRAC
**CCG of GP practice**	CCG of patient's GP practice	CCG_GP_PRACTICE
**CCG of Treatment**	CCG of Treatment	CCG_TREATMENT
**Self-referrals for minor conditions**	Referral mode,<br>SUS PbR derived healthcare resource group code	AEREFSOURCE<br>SUSHRG
**Cost**	Trust derived HRG value	HRGNHS
**GP referrals**	Referral mode	AEREFSOURCE
**Arrival age**	Age on arrival	ARRIVALAGE
**Sex**	Sex of patient	SEX
**Ethnicity**	Ethnic category	ETHNOS
**Deprivation**	Lower Super Output Area (to obtain IMD)	LSOA11
**Diagnosis**	A&E diagnosis	DIAG_NN
**EFFECT ON ADMISSIONS/WORKLOAD**		
**Admissions from A&E**	Attendance disposal	AEATTENDISP
**A&E Waiting times**		
**A&E time to assessment (waiting time)**	A&E Duration to assessment	INITDUR
**A&E time to treatment (waiting time)**	A&E Duration to treatment	TRETDUR
**A&E total duration**	A&E duration to departure	DEPDUR
**ADMISSIONS DATA**		
**KEY BASE DATA**		
**Provider code**	Provider code	PROCODET
**Arrival date**	Date of admission	ADMIDATE
**Discharge date**	Date of discharge	DISDATE
**GP practice**	Code of GP practice	GPPRAC
**CCG of GP practice**	CCG of patient's GP practice	CCG_GP_PRACTICE
**CCG of Treatment**	CCG of Treatment	CCG_TREATMENT
**Cost**	Trust derived HRG value	HRGNHS
**Arrival age**	Age on arrival	ADMIAGE
**Sex**	Sex of patient	SEX
**Ethnicity**	Ethnic category	ETHNOS
**Deprivation**	Lower Super Output Area (to obtain IMD)	LSOA11
**Ambulatory care-sensitive conditions admissions (ICD)**	All Diagnosis codes	DIAG_NN

For peer review only

(i) Uptake

Patients could book an appointment which could then be attended or not. Focussing on booked appointments alone would give an indication of the demand for the service, whilst assessments of appointments actually used shed light on realised demand and is more insightful for evaluating actual impacts on service use. We estimated two separate sets of probability models:

1) the probability an appointment was booked, and
2) the probability an appointment was booked and attended ('used')

We assessed differences in the rate of uptake by estimating probability models (probit regressions) of appointment use (y_{ij}):

$$y_{ij} = \beta_1 D_{ij} + \beta_2 M_{ij} + \beta_3 CCG_j + e_{ij} \qquad [1]$$

where the unit of analysis is available appointment i in CCG j.

Probit models were chosen as these give the absolute variation in uptake rather than odds-ratios which is more informative for understanding the scale of uptake. To assess whether uptake of the service varied across day of the week we included day of week dummies in the regression (D_i), with the estimates ($\hat{\beta}_1$) providing the differences in the rate of uptake relative to the base week day (Monday); this enables a test for whether uptake is lower on particular days. To assess whether there is evidence of assimilation to the service we include calendar month dummies (M_i). If assimilation is evident the estimates on the calendar month dummies should be monotonically increasing over the period. To account for potential confounding caused by variations in the provision and delivery of services across schemes we include CCG dummies (fixed-effects) in the regression (CCG_j). Standard errors are clustered at CCG level.

Estimates are presented as average marginal effects (using Stata's margins command), which give the average percentage point effect of the variable relative to the base category across the sample.

(ii) Patient demographics analysis

Our second set of analyses concentrated on booked appointments and exploited variations in service delivery to test whether there were associations in the demand for particular types of services (booking type (pre-booked or same day) and appointment type (GP or nurse)) with day of week, calendar month, patient age and gender, and CCG. We estimated two separate sets of probability models:

1) The probability an appointment was pre-booked versus same day;

For peer review only

2) The probability an appointment was for a GP versus for a nurse

We assessed differences in the rate of uptake by estimating probability models (probit regressions) of appointment type (y_{ij}):

$$y_{ij} = \beta_1 D_{ij} + \beta_2 M_{ij} + \beta_3 DNA_{ij} + \beta_4 Female_{ij} + \beta_5 Age_{ij} + \beta_6 CCG_j + e_{ij} \quad [2]$$

where the unit of analysis is the type of *booked* appointment i in CCG j. $Female_{ij}$ is a dummy variable indicating the gender of the patient booked for appointment i in CCG j, and Age_{ij} is a set of 10 age-band dummies indicating the age of the patient. DNA_{ij} is a dummy variable indicating whether the appointment was attended or not. For all models estimated, estimates are presented as average marginal effects and standard errors are clustered at CCG level. Schemes that exclusively provided a certain service (e.g. only GP appointments) are excluded from the relevant analysis since uptake of the alternative is necessarily zero.

For peer review only

CLAHRC Patient Panel Protocol Review
Collated Feedback and Responses

**Feedback from panel**	**Project team response**
The interview document [referring to the interview schedule] seems ok.	No response required.
The survey document I would change slightly as per the attachment:<br><br>5. Which of the following do you feel (may have-deleted) has benefited from the implementation of seven day access to primary care?<br><br>6. Remove mentions of 'efforts to' from the response options e.g. efforts to reduce demand on A&E services.	
This is a very comprehensive, well thought out document. It is a very comprehensive protocol indeed.<br><br>Appointments must be booked in advance, but what if changes take place on the part of either the patient or the GP hub nearer the time of the appointment?<br><br>Evidence of use and pattern of use at weekends is crucial! Evenings will prove popular, Saturday mornings will indicate some demand but I am unsure about the demand on Saturday afternoons and especially Sundays. I am referring to planned appointments and not Emergency appointments!	No response required.<br><br>No change to protocol as this situation is not specific to the additional availability, as appointments are also booked in advance within core services.<br><br>Agree that utilisation by day of week and time of day is important. This will be monitored via the activity data.
The 'don't know' response option should be removed, in all cases, from the staff survey.	No change to staff survey, as 'don't know' response is required to ensure that those who select an alternative option do so as there is an element of certainty about their choice. Important for data quality.
The following questions are suggested to be added to the<br>interview schedule:<br><br>a. How effective has the 7 day access scheme been in terms of number of patients visiting during extended hours?<br>b. Describe the patterns of demand during the extended hours and the average cost per 10 minute slot?	No change to interview schedule, as captured via the activity data.<br><br>No change to interview schedule, as captured via the activity data.

c. Indicate staff morale during the extended hours?	No change to interview schedule, as captured via the staff survey.

For peer review only

Table S1 Summary of 7 Day Access Implementation by Area

Clinical Commissioning Group	Live date (2016)	Service	Self-referral	Monday-Friday opening	Saturday opening	Sunday opening
**CCG1**						
**Hub 1**	2 April	GP and nurse	No	No	09:00-13:00	10:00-13:00
**Hub 2**	2 April	GP and nurse	No	No	09:00-13:00	10:00-13:00
**CCG2**						
**Hub 1**	11 January	GP and nurse	Yes	18:30-21:00	08:00-18:00	10:00-13:00
**Hub 2**	1 January	GP and nurse	Yes	18:30-21:00	08:00-18:00	10:00-13:00
**Hub 3**	2 January	GP and nurse	Yes	18:30-21:00	08:00-18:00	10:00-13:00
**Hub 4**	2 January	GP and nurse	Yes	18:30-21:00	08:00-18:00	10:00-13:00
**CCG3**						
**Hub 1**	5 January	GP only	Yes	18:30-20:00	10:00-14:00	10:00-14:00
**Hub 2**	10 August	GP only	Yes	18:30-20:00	10:00-14:00	No
**CCG4**						
**Hub 1**	3 January	GP only	No	18:30-20:00	09:00-11:45	09:00-12:00
**Hub 2**	30 January	GP only	No	18:15-20:00 (no Thursday or Friday)	09:00-11:45	09:00-11:45
**Hub 3**	25 February	GP only	No	18:30-20:00	09:00-11:45	09:00-12:00
**CCG5**						
**Hub 1**	16 January	GP and nurse	No	No	09:00-13:00	No
**Hub 2**	16 January	GP and nurse	No	No	09:00-13:00	No

For peer review only

Table S2 Activity Capture Minimum Dataset

Data requested from Clinical Commissioning Group	Format
Volume of appointments available	
Date	DD/MM/YYYY
Time of appointment	HH:MM
Discipline	GP/Nurse
Planned/pre-booked vs same day/urgent	Pre-booked/Same day
Method of appointment (face-to-face/phone)	Face-to-face/phone
Volume of appointments booked and booked and attended	
Date	DD/MM/YYYY
Time of appointment	HH:MM
Discipline	GP/Nurse
Age	10 year age bands: 0-9 to 90+
Gender	Male/Female
Registered GP practice	Practice code identifier
Postcode (first part)	First half
Planned/pre-booked vs same day/urgent	Pre-booked/Same day
Method of appointment (face-to-face/phone)	Face-to-face/phone
Method of appointment (face-to-face/phone)	Face-to-face/phone

For peer review only

Table S3 Rates of appointment use

	Unadjusted appointment booked (95% CI)	Unadjusted appointment used (95% CI)
**Day of week**		
**Monday (base category)**		
**Tuesday**	11.54 (7.90, 15.19)	9.52 (6.51, 12.53)
**Wednesday**	10.77 (3.57, 17.76)	8.20 (1.75, 14.65)
**Thursday**	12.76 (9.45, 16.08)	7.14 (3.96, 10.31)
**Friday**	9.92 (7.19, 12.65)	4.02 (0.62, 7.42)
**Saturday**	1.76 (-6.96, 10.49)	-2.00 (-9.26, 5.27)
**Sunday**	-20.35 (-36.51, -4.19)	-19.21 (-34.36, -4.05)
**Calendar month**		
**January (base category)**		
**February**	23.93 (15.15, 32.71)	20.46 (13.88, 27.04)
**March**	23.93 (17.22, 30.64)	19.52 (15.68, 23.37)
**April**	28.96 (14.72, 43.19)	24.44 (13.63, 35.25)
**May**	23.65 (11.06, 36.24)	19.82 (10.34, 29.29)
**June**	24.29 (8.32, 40.26)	21.43 (8.97, 33.89)
**July**	29.84 (17.46, 42.23)	24.18 (14.12, 34.24)
**August**	23.99 (6.93, 41.06)	20.12 (6.88, 33.37)
**September**	31.72 (13.78, 49.67)	25.77 (11.45, 40.09)
**October**	32.99 (17.24, 48.74)	28.24 (15.93, 40.56)
**November**	31.32 (14.84, 47.80)	25.69 (12.83, 38.54)
**December**	31.22 (10.11, 52.32)	23.52 (7.00, 40.04)
**CCG scheme**		
**CCG1***		
**CCG2**	-16.17 (-16.17, -16.17)	-8.46 (-8.46, -8.46)
**CCG3**	-32.83 (-32.83, -32.83)	-23.02 (-23.02, -23.02)
**CCG4**	-12.17 (-12.17, -12.17)	-8.62 (-8.62, -8.62)
**CCG5 (base category)**		
**Sample size**	42,472	42,472

Unadjusted estimates obtained via probit regression of appointment status against each set of dummies (estimated separately): day of week, calendar month, and CCG scheme.
Estimates are presented as average marginal effects which give the percentage point effect (when multiplied by 100) of the variable relative to the base category.
*CCG1 did not provide data to enable identification of whether a booked appointment was subsequently attended so does not feature in the analysis

For peer review only

Table S4 Probability models for appointment use with Sunday interaction with calendar month

	Appointment booked (95% CI)	Appointment used (95% CI)
**Day of week (base: Monday)**		
**Tuesday**	0.430 0.320,0.539)	0.288 (0.237,0.339)
**Wednesday**	0.410 (0.232,0.588)	0.249 (0.104,0.394)
**Thursday**	0.481 (0.408,0.553)	0.204 (0.144,0.265)
**Friday**	0.367 (0.268,0.466)	0.119 (0.031,0.207)
**Saturday**	-0.010 (-0.278,0.258)	-0.098 (-0.303,0.108)
**Sunday**	-0.560 (-0.950,-0.170)	-0.659 (-1.127,-0.191)
**Calendar month (base: January)**		
**February**	0.729 (0.580,0.878)	0.562 (0.436,0.687)
**March**	0.563 (0.377,0.748)	0.418 (0.313,0.524)
**April**	0.764 (0.437,1.091)	0.591 (0.377,0.805)
**May**	0.599 (0.298,0.900)	0.451 (0.248,0.655)
**June**	0.684 (0.324,1.044)	0.552 (0.297,0.807)
**July**	0.929 (0.755,1.103)	0.636 (0.488,0.785)
**August**	0.664 (0.266,1.061)	0.493 (0.211,0.775)
**September**	0.938 (0.515,1.362)	0.650 (0.373,0.928)
**October**	0.986 (0.604,1.367)	0.708 (0.434,0.981)
**November**	0.889 (0.495,1.283)	0.622 (0.341,0.903)
**December**	0.895 (0.311,1.479)	0.578 (0.195,0.961)
**Sunday*Calendar month**		
**February**	-0.407 (-0.685,-0.128)	-0.229 (-0.475,0.017)
**March**	0.238 (-0.036,0.512)	0.334 (0.026,0.642)
**April**	0.213 (-0.502,0.929)	0.260 (-0.486,1.006)
**May**	0.307 (-0.420,1.033)	0.368 (-0.349,1.086)
**June**	-0.205 (-0.874,0.464)	-0.044 (-0.708,0.619)
**July**	-0.197 (-1.042,0.648)	0.107 (-0.670,0.884)
**August**	-0.078 (-0.841,0.686)	0.084 (-0.670,0.838)
**September**	0.013 (-0.693,0.719)	0.214 (-0.573,1.002)
**October**	0.131 (-0.439,0.701)	0.389 (-0.198,0.975)
**November**	0.099 (-0.522,0.721)	0.303 (-0.290,0.895)
**December**	-0.001 (-0.984,0.981)	0.290 (-0.573,1.154)
**CCG scheme (base: CCG5)**		
**CCG2**	-0.590 (-0.703,-0.477)	-0.298 (-0.373,-0.223)
**CCG3**	-1.134 (-1.214,-1.053)	-0.678 (-0.742,-0.614)
**CCG4**	-0.395 (-0.497,-0.292)	-0.281 (-0.347,-0.214)
**Constant**	0.516 (-0.046,1.077)	0.210 (-0.205,0.625)
**Sample size**	42,472	42,472

Probit regression of appointment status against day of week, calendar month, Sunday interaction with calendar month, and CCG scheme.

For peer review only

Estimates are not presented as average marginal effects due to the inability to identify the marginal effect of an interaction term.

For peer review only

Table S5 Missing appointment data for booked appointments

CCG		Total booked	Missing patient gender	Missing patient age	Missing appointment type	Missing booking type	Any missing appointment data
**CCG2**		23,345	73 (0.31%)	246 (1.05%)	0 (0.00%)	0 (0.00%)	294 (1.26%)
	**Hub 1**	5,220	7 (0.13%)	76 (1.46%)	0 (0.00%)	0 (0.00%)	80 (1.53%)
	**Hub 2**	6,435	37 (0.57%)	66 (1.03%)	0 (0.00%)	0 (0.00%)	92 (1.43%)
	**Hub 3**	5,229	16 (0.31%)	70 (1.34%)	0 (0.00%)	0 (0.00%)	82 (1.57%)
	**Hub 4**	6,461	13 (0.20%)	34 (0.53%)	0 (0.00%)	0 (0.00%)	40 (0.62%)
**CCG4**		4,463	227 (5.09%)	227 (5.09%)	345 (7.73%)	0 (0.00%)	535 (11.99%)
	**Hub 1**	1,878	90 (4.79%)	90 (4.79%)	141 (7.51%)	0 (0.00%)	222 (11.82%)
	**Hub 2**	1,060	60 (5.66%)	60 (5.66%)	83 (7.83%)	0 (0.00%)	133 (12.55%)
	**Hub 3**	1,525	77 (5.05%)	77 (5.05%)	121 (7.93%)	0 (0.00%)	180 (11.80%)
**CCG5**		2,183	11 (0.50%)	51 (2.34%)	0 (0.00%)	3 (0.14%)	58 (2.66%)
	**Hub 1**	1,094	8 (0.73%)	19 (1.74%)	0 (0.00%)	0 (0.00%)	22 (2.01%)
	**Hub 2**	1,089	3 (0.28%)	32 (2.94%)	0 (0.00%)	3 (0.28%)	36 (3.31%)
**CCG3**		2,050	0 (0.00%)	0 (0.00%)	0 (0.00%)	0 (0.00%)	0 (0.00%)
	**Hub 1**	1,806	0 (0.00%)	0 (0.00%)	0 (0.00%)	0 (0.00%)	0 (0.00%)
	**Hub 2**	244	0 (0.00%)	0 (0.00%)	0 (0.00%)	0 (0.00%)	0 (0.00%)
**Total**		32,041	311 (0.97%)	524 (1.64%)	345 (1.08%)	3 (0.01%)	887 (2.77%)

For peer review only

Table S6 Probability model for appointment containing missing data

	Missing appointment data^ (95% CI)
**Day of week (base: Monday)**	
**Tuesday**	0.36 (-0.67, 1.39)
**Wednesday**	0.34 (-0.42, 1.10)
**Thursday**	0.05 (-0.44, 0.55)
**Friday**	0.86 (0.66, 1.06)
**Saturday**	0.72 (0.19, 1.26)
**Sunday**	0.73 (-0.24, 1.70)
**Calendar month (base: January)**	
**February**	1.47 (0.77, 2.17)
**March**	0.03 (-0.89, 0.95)
**April**	0.28 (-1.81, 2.38)
**May**	0.79 (-2.59, 4.17)
**June**	1.24 (-1.08, 3.57)
**July**	1.40 (-1.06, 3.86)
**August**	-0.80 (-3.20, 1.60)
**September**	2.49 (-3.23, 8.20)
**October**	1.77 (-2.11, 5.66)
**November**	2.99 (-1.63, 7.60)
**December**	2.87 (-1.67, 7.42)
**CCG scheme (base: CCG5)**	
**CCG2**	-1.47 (-1.81, -1.13)
**CCG3**	-
**CCG4**	4.72 (4.12, 5.33)
**Sample size**	29,991

^ CCG3 had 100% data completion (n=2,050)
Probit regression of missing data indicator (gender, age, appointment type, booking type, DNA status) against day of week, calendar month, and CCG scheme.
Estimates are presented as average marginal effects which give the percentage point effect (when multiplied by 100) of the variable relative to the base category.

For peer review only

Table S7 Booked appointment data: summary statistics

Appointment characteristic	Frequency	Percent	Cumulative percentage
**Day of week**			
**Monday**	3,369	10.81	10.81
**Tuesday**	3,576	11.48	22.29
**Wednesday**	3,211	10.31	32.60
**Thursday**	3,328	10.68	43.28
**Friday**	3,286	10.55	53.83
**Saturday**	11,366	36.48	90.31
**Sunday**	3,018	9.69	100.00
**Total**	31,154	100.00	
**Calendar month**			
**January**	1,441	4.63	4.63
**February**	2,299	7.38	12.00
**March**	2,711	8.70	20.71
**April**	2,727	8.75	29.46
**May**	2,735	8.78	38.24
**June**	2,434	7.81	46.05
**July**	2,864	9.19	55.24
**August**	2,661	8.54	63.79
**September**	2,716	8.72	72.50
**October**	3,096	9.94	82.44
**November**	2,838	9.11	91.55
**December**	2,632	8.45	100.00
**Total**	31,154	100.00	
**CCG**			
**CCG2**	23,051	73.99	73.99
**CCG3**	2,050	6.58	80.57
**CCG4**	3,928	12.61	93.18
**CCG5**	2,125	6.82	100.00
**Total**	31,154	100.00	
**Appointment type**			
**GP**	25,798	82.81	82.81
**Nurse**	5,356	17.19	100.00
**Total**	31,154	100.00	
**Booking type**			
**Pre-booked**	14,423	46.30	46.30
**Same-day**	16,731	53.70	100.00
**Total**	31,154	100.00	
**Attended status**			
**DNA**	3,817	12.25	12.25
**Attended**	27,337	87.75	100.00
**Total**	31,154	100.00	
**Gender**			
**Male**	12,913	41.45	41.45
**Female**	18,241	58.55	100.00
**Total**	31,154	100.00	
**Age**			
**0-9**	3,361	10.79	10.79
**10-19**	3,112	9.99	20.78
**20-29**	5,700	18.30	39.07
**30-39**	5,291	16.98	56.06
**40-49**	4,929	15.82	71.88

For peer review only

**50-59**	4,409	14.15	86.03
**60-69**	2,645	8.49	94.52
**70-79**	1,208	3.88	98.40
**80-89**	458	1.47	99.87
**90+**	41	0.13	100.00
**Total**	31,154	100.00	

Appointment characteristics for appointments booked and used with complete (non-missing) data

For peer review only

Table S8 Demographic distributions of extended access appointment users and usual care in the GPPS

	Males			Females			All
	Frequency	%	Cumulative %	Frequency	%	Cumulative %	Cumulative %
**Extended access**							
**Age**							
**0-9**	1,492	13.16	13.16	1,437	8.98	8.98	10.71
**10-19**	1,075	9.48	22.64	1,581	9.88	18.87	20.43
**20-29**	1,540	13.58	36.22	3,129	19.56	38.43	37.51
**30-39**	1,739	15.34	51.55	2,758	17.24	55.67	53.96
**40-49**	1,795	15.83	67.38	2,603	16.27	71.94	70.05
**50-59**	1,807	15.93	83.32	2,248	14.05	85.99	84.88
**60-69**	1,187	10.47	93.78	1,335	8.35	94.34	94.11
**70-79**	494	4.36	98.14	649	4.06	98.39	98.29
**80-89**	199	1.75	99.89	228	1.43	99.82	99.85
**90+**	12	0.11	100.00	29	0.18	100.00	100.00
**Total**	11,340	100.00		15,997	100.00		
**GP Patient Survey users**							
**Age**							
**18-24**	105	2.98	2.98	254	5.20	5.20	4.27
**25-34**	217	6.16	9.14	501	10.26	15.47	12.81
**35-44**	377	10.70	19.84	646	13.23	28.70	24.99
**45-54**	616	17.48	37.32	867	17.76	46.47	42.63
**55-64**	806	22.87	60.19	913	18.71	65.17	63.08
**65-74**	842	23.89	84.08	924	18.93	84.10	84.09
**75-84**	452	12.83	96.91	568	11.64	95.74	96.23
**85+**	109	3.09	100.00	208	4.26	100.00	100.00
**Total**	3,524	100.00		4,881			

For peer review only

STROBE Statement—Checklist of items that should be included in reports of ***cohort studies***

	Item No	Recommendation	Location in manuscript
**Title and abstract**	1	(*a*) Indicate the study's design with a commonly used term in the title or the abstract	Title
		(*b*) Provide in the abstract an informative and balanced summary of what was done and what was found	Abstract
**Introduction**			
Background/rationale	2	Explain the scientific background and rationale for the investigation being reported	Introduction: Paragraphs 1, 2 & 3
Objectives	3	State specific objectives, including any prespecified hypotheses	Introduction: Paragraph 4
**Methods**			
Study design	4	Present key elements of study design early in the paper	Introduction: Paragraph 2 Methods: Paragraphs 1, 2, 3, & 4
Setting	5	Describe the setting, locations, and relevant dates, including periods of recruitment, exposure, follow-up, and data collection	Methods: Paragraphs 1, 2, 3, & 12 Figure 1 Supplementary Table S1
Participants	6	(*a*) Give the eligibility criteria, and the sources and methods of selection of participants. Describe methods of follow-up	Methods: Paragraphs 1, 2, 3, & 12 Figure 1
		(*b*) For matched studies, give matching criteria and number of exposed and unexposed	N/A
Variables	7	Clearly define all outcomes, exposures, predictors, potential confounders, and effect modifiers. Give diagnostic criteria, if applicable	Methods: Paragraphs 5, 6, 7, 8, 9, 10, 11 & 12
Data sources/ measurement	8*	For each variable of interest, give sources of data and details of methods of assessment (measurement). Describe comparability of assessment methods if there is more than one group	Methods: Paragraphs 1, 2, 3, & 12 Supplementary Table S2
Bias	9	Describe any efforts to address potential sources of bias	Methods: Paragraphs 7, 10, 11, & 12
Study size	10	Explain how the study size was arrived at	Methods: Paragraphs 1, 3 & 12 Results: Paragraph 1 Figure 1
Quantitative variables	11	Explain how quantitative variables were handled in the analyses. If applicable, describe which groupings were chosen and why	Methods: Paragraphs 6, 7, 8, 9, 10, 11, & 12 Supplementary Table S2
Statistical methods	12	(*a*) Describe all statistical methods, including those used to control for confounding	Methods: Paragraphs 6, 7, 8, 9, 10, 11, & 12

For peer review only

			Supplementary Text S2
		(*b*) Describe any methods used to examine subgroups and interactions	Results: Paragraph 4
		(*c*) Explain how missing data were addressed	Methods: Paragraphs 11
		(*d*) If applicable, explain how loss to follow-up was addressed	N/A
		(*e*) Describe any sensitivity analyses	N/A
**Results**			
Participants	13*	(a) Report numbers of individuals at each stage of study—eg numbers potentially eligible, examined for eligibility, confirmed eligible, included in the study, completing follow-up, and analysed	Results: Paragraph 1, 5, & 9 Tables 1, 2 & 5 Figure 1
		(b) Give reasons for non-participation at each stage	Results: Paragraphs 1 & 5 Tables 1, 2, 3 & 4 Footnotes
		(c) Consider use of a flow diagram	Figure 1
Descriptive data	14*	(a) Give characteristics of study participants (eg demographic, clinical, social) and information on exposures and potential confounders	Results: Paragraphs 6, 7, 8, 9, & 10 Supplementary Tables S7 & S8
		(b) Indicate number of participants with missing data for each variable of interest	Results: Paragraph 5 Supplementary Table S5
		(c) Summarise follow-up time (eg, average and total amount)	N/A
Outcome data	15*	Report numbers of outcome events or summary measures over time	Results: Paragraph 3 Figure 2 & 3 Supplementary Table S3
Main results	16	(*a*) Give unadjusted estimates and, if applicable, confounder-adjusted estimates and their precision (eg, 95% confidence interval). Make clear which confounders were adjusted for and why they were included	Results: Paragraphs 1, 2, 3, 4, 5, 6, 7, 8, & 9 Tables 1, 2, 3, 4 & 5 Supplementary Tables S3, S4, S6, S7 & S8
		(*b*) Report category boundaries when continuous variables were categorized	N/A
		(*c*) If relevant, consider translating estimates of relative risk into absolute risk for a meaningful time period	Methods: Paragraph 7
Other analyses	17	Report other analyses done—eg analyses of subgroups and interactions, and sensitivity analyses	Results: Paragraph 4
**Discussion**			
Key results	18	Summarise key results with reference to study objectives	Discussion: Paragraph 1
Limitations	19	Discuss limitations of the study, taking into account	Discussion:

For peer review only

For peer review only - http://bmjopen.bmj.com/site/about/guidelines.xhtml

		sources of potential bias or imprecision. Discuss both direction and magnitude of any potential bias	Paragraph 2
Interpretation	20	Give a cautious overall interpretation of results considering objectives, limitations, multiplicity of analyses, results from similar studies, and other relevant evidence	Discussion: Paragraphs 3, 4, 5, & 6
Generalisability	21	Discuss the generalisability (external validity) of the study results	Discussion: Paragraph 2
**Other information**			
Funding	22	Give the source of funding and the role of the funders for the present study and, if applicable, for the original study on which the present article is based	Funding statement

*Give information separately for exposed and unexposed groups.

Note: An Explanation and Elaboration article discusses each checklist item and gives methodological background and published examples of transparent reporting. The STROBE checklist is best used in conjunction with this article (freely available on the Web sites of PLoS Medicine at http://www.plosmedicine.org/, Annals of Internal Medicine at http://www.annals.org/, and Epidemiology at http://www.epidem.com/). Information on the STROBE Initiative is available at http://www.strobe-statement.org.

For peer review only

BMJ Open

An investigation of the demand for a 7-day (extended access) primary care service: an observational study from pilot schemes in England

Journal:	*BMJ Open*
Manuscript ID	bmjopen-2018-028138.R1
Article Type:	Research
Date Submitted by the Author:	31-May-2019
Complete List of Authors:	Whittaker, William; University of Manchester, Manchester Centre for Health Economics<br>Anselmi, Laura; University of Manchester, Manchester Centre for Health Economics<br>Nelson, Pauline; University of Manchester Alliance Manchester Business School; NIHR CLAHRC Greater Manchester, UK<br>O'Donnell, Caroline; NIHR CLAHRC Greater Manchester, UK<br>Ross, Natalie; University of Manchester Alliance Manchester Business School<br>Rothwell, Katy; NIHR CLAHRC Greater Manchester, UK<br>Hodgson, Damian; University of Manchester Alliance Manchester Business School; NIHR CLAHRC Greater Manchester, UK
**Primary Subject Heading**:	Health services research
Secondary Subject Heading:	Health policy, Health economics
Keywords:	PRIMARY CARE, ACCESS, HEALTH ECONOMICS, Health policy < HEALTH SERVICES ADMINISTRATION & MANAGEMENT

SCHOLARONE™
Manuscripts

An investigation of the demand for a 7-day (extended access) primary care service: an observational study from pilot schemes in England

Whittaker W[1], Anselmi L[1], Nelson P[2,3], O'Donnell C[3], Ross N[2], Rothwell K[3], Hodgson D[2,3].

[1]Manchester Centre for Health Economics, University of Manchester, UK
[2]Alliance Manchester Business School, University of Manchester, UK
[3]NIHR CLAHRC Greater Manchester, UK

Correspondence to:
William.whittaker@manchester.ac.uk
Manchester Centre for Health Economics, 4.320 Jean McFarlane Building, University of Manchester, Oxford Road, Manchester, M13 9PL

Word count: 5,655

For peer review only

ABSTRACT

Objectives - To understand how the uptake of an extended primary care service in the evenings and weekend varied by day of week and over time. Secondary objectives were to understand patient demographics of users of the service and how these varied by type of appointment and to core hour users.

Design –Observational study.

Setting - Primary care extended access appointments data in 13 centres in Greater Manchester, England during 2016.

Participants – Appointments could be booked by 1,261,326 patients registered with a family practitioner in five Clinical Commissioning Group geographic areas.

Main outcome measures – Primary outcome measure was whether an appointment was used (booked and attended), secondary outcome measures included whether used appointments were pre-booked or booked the same-day, and delivered by a family or nurse practitioner. Additional analyses compared patient demographics to patients reporting the use of core-hour primary care services.

Results - 65.33% of 42,472 appointments were booked and attended (used). Usage of appointments was lowest on a Sunday at 46.73% (18.07 percentage points lower usage than on Mondays (95%CI -32.46 to -3.68)). Pre-booked appointments were more likely to be booked amongst age groups 0 to 9 and to result in patients not attending an appointment. Family practitioner appointments were increasingly less likely to be booked with age in comparison to nurse appointments. Patients attending extended access appointments tended to be younger in comparison to core hour patients.

Conclusions -There is spare capacity in the extended access service, particularly on Sundays, suggesting reconfigurations of the service may be needed to improve efficiency of delivering the service. Patients demographics suggest the service is used by a relatively younger population than core hour services. Patient demographics varied with the types of appointment provided, these findings may help healthcare providers improve usage by tailoring appointment provision to local populations.

Strengths and limitations of this study

- The first study assessing same-day and pre-bookable appointments at evenings and weekends in general practice
- The first study comparing demographics of patients using evening and weekend appointments to patients using core hour appointments
- The first study assessing uptake of evening and weekend appointments over time
- The study covers five areas with differing provision, but may not be generalisable to other settings
- No information on child use of core hours was available, limiting an assessment of this group of the population

For peer review only - http://bmjopen.bmj.com/site/about/guidelines.xhtml

INTRODUCTION

Providing access to health care services when needed is a key target for health systems worldwide.[1] However, patients commonly report difficulties in accessing primary care, particularly outside of core working hours.[2,3] Constrained access to primary care has been a reason for increased patient dissatisfaction and use of emergency care in a variety of countries and settings.[4] Rising use of emergency department services and patient dissatisfaction with access have highlighted a need for better access to primary care.

In England primary care core hour services are provided by General Practitioners (GP) 8am to 6:30pm Monday to Friday either by pre-booking or calling on the same day. The National Health Service (NHS) in England aims to extend access to primary care services in the evening and at weekends as part of their strategy for delivering primary care by 2020/2021.[5] The strategy seeks to enable local commissioners of health care to redesign primary care services and commission extra capacity so that by 2020 *'everyone has access to GP services, including sufficient routine appointments at evenings and weekends to meet locally determined demand, alongside effective access to out-of-hours (OoH) and urgent care services*'. Extended access has been piloted nationally by NHS England since 2013 through the Prime Minister's Challenge Fund, and its successor the GP Access Fund. Wave 1 covered 7.5m people in 2014, rising to 18m (a third of the population of England) by 2015 in Wave 2. In parallel, NHS England Greater Manchester (NHSEGM), now the Greater Manchester Health and Social Care Partnership (GMHSCP), have also piloted extended access appointments with an initial wave in 2014[6], followed by a roll-out throughout Greater Manchester (GM) from 2016 in line with the region's devolution and health and social care strategy[7]. A description of extended access appointments in Greater Manchester is provided in Box 1.

Box 1: Description of Greater Manchester extended access appointments in primary care

<u>What is the service?</u><br>Extended access appointments are appointments in primary care outside of core hours (typically 8am to 6:30pm). Appointments are provided in addition to usual care outside of core family practitioner hours which includes: Walk in Centres, Out-of-Hour services, Accident and Emergency/secondary care, NHS Direct ('111'). The services may have been designed to replace existing Direct Enhanced Services arrangements (additional hours of opening contractually agreed with NHS England).<br><br><u>Who provided the service?</u><br>Extended access appointments are appointments with either a GP or nurse practitioner.<br><br><u>How is the service delivered?</u><br>Extended access appointments are typically face to face appointments delivered individually.

For peer review only

For peer review only - http://bmjopen.bmj.com/site/about/guidelines.xhtml

Where is the service delivered?

Extended access appointments are usually provided through one practice or similar premises which acts as a local 'hub' covering several practices in a given geographic area, with between 1-4 hubs serving the patient population. Hub locations were decided by local health care commissioners (Clinical Commissioning Groups, CCGs). CCGs are responsible for the commissioning of health care to a local, geographically defined population comprising of a number of family practitioner (GP) practices.

When and how much of the service is delivered?

Extended access appointments under this study became operational at varying times over the 2016 calendar year, this varied by CCG.

Tailoring of the service

The type, referral route(s) and timing of appointments are decided by the local CCG. Variations between schemes existed by referral route, whether pre-bookable appointments were available, discipline of appointment (GP or nurse, all face to face), time and day of appointments.

For peer review only

Extended access evaluations to date have found reductions in emergency service use for patients with minor injuries/issues in national[8], sub-national[9], and Greater Manchester[6, 10] schemes. However, the reductions in emergency service use alone may not sufficiently offset the cost of providing an extended service, raising concerns that an extended access service is not a cost-effective use of health system resources.[10] Concerns of opportunity cost (the net benefits of services that could have been provided instead of extended access) are particularly pertinent in times of increasing financial/workforce pressures for many health systems. However, in addition to impacts on secondary care, and particularly in the current absence of an understanding of the long-term health benefits of the service; there are additional aspects of extended access that need to be considered when assessing the value of the service. First, there is a lack of knowledge concerning how the service is being used with little assessment of how best to operationalise extended appointments efficiently (which would have implications for cost-effectiveness). One way to measure efficiency could be uptake of the service, with unbooked appointments signalling inefficient use of resources. Uptake of extended access appointments has been reported as 71% with lower uptake on weekends, particularly Sundays.[8, 11] A sub-national evaluation of 3 schemes found uptake was lower where appointments were not pre-bookable, and lower on Sundays.[11] An evaluation of the initial wave of Greater Manchester 'demonstrators' found uptake of 65.5% and lower uptake on weekends, particularly on Sundays.[6] Studies of uptake have concentrated on high level aggregated descriptive statistics of uptake over time and/or by day of week. Since service provision varies across pilots it is also important to understand how uptake varies across services delivered. Commissioners are tasked with delivering extended access services in light of patient demand[5, page 48], the lack of evidence to inform efficient provision is a substantial gap in knowledge. Second, it is currently unknown what the patient

For peer review only - http://bmjopen.bmj.com/site/about/guidelines.xhtml

demographics are for an extended access service delivering pre-bookable and same-day appointments with existing analyses being for small scale schemes[11] or urgent care appointments only[12], it is also unclear whether patients using the service differ from patients using core hours general practice, understanding these may help inform service provision and infer whether the service is hitting an unmet need and/or reducing pressures in core hours.

Our aim in this paper is threefold. First, using data from Greater Manchester UK schemes throughout 2016 we sought to understand whether the service is being used and whether this varies by day of week and over time. Second, we sought to understand whether there were differences in the services being used by particular age and gender groups. Third, we aimed to compare the users of extended access appointments to users of general practice core hours. The findings will help inform considerations about extending access to general practice and how such a service might be better targeted to particular population groups.

METHODS

Data

The study evaluated the second wave of Greater Manchester schemes that delivered extended access throughout 2016. Out of Greater Manchester's ten Clinical Commissioning Group (CCG) areas, three were already delivering extended access under the national GP Access Fund (GPAF) scheme during the study period (see Figure 1). In line with the GM Health and Social Care Partnership Primary Care Strategy, the other seven CCG areas, not previously in receipt of Wave 1 Prime Minister's Challenge Fund (PMCF) or Wave 2 GPAF funding, received financial support from GMHSCP to implement extended access. However, only five of these seven CCGs were included in the evaluation because two of the CCGs were not captured in the data collection process: one had not implemented the service in 2016; the other did not provide data. A table summarising the five CCG schemes with extended access can be found in Supplementary Table S1. The evaluation was carried out by the National Institute for Health Research Collaboration for Leadership in Applied Health Research and Care Greater Manchester (NIHR CLAHRC GM) and comprised of assessments of activity, a process evaluation (seeking to understand the organisational and operational issues that arose in the implementation of the service and how areas addressed these), and assessments of impacts on other sectors of the healthcare system.[13] This study builds on the assessment of activity contained within the evaluation report.

<Figure 1 here>

For peer review only

We used administrative data on extended access appointments available, booked and attended in CCG schemes in Greater Manchester by calendar date over the year 2016 (January to December). The NIHR CLAHRC GM evaluation team requested a minimum dataset from each CCG (Supplementary Table S2). The aim of this data collection was to inform what new appointment activity was associated with the extended access schemes, whether this new activity was used, and to understand the types of patients using the service (a protocol for the evaluation can be found in Supplementary Text S1). Data on each appointment made available were collected and made available by the responsible CCG. CCGs submitted the data to NIHR CLAHRC GM throughout the period. The data included information on date, use (volume available, booked, booked and used), type of appointment (family practitioner or nurse practitioner, pre-booked (booked on a previous calendar date) or booked same-day), and patient characteristics (the patient's registered practice, age and gender) for booked and attended appointments. Data quality and coverage was dependent on the CCG submitting the data, whilst NIHR CLAHRC GM could raise instances of incomplete data with CCGs it was up to the CCG to respond. Patient characteristics were limited due to issues of patient confidentiality. To maintain anonymity, patient age was given in 10-year age bands. Deprivation statistics were considered although, due to data confidentiality, we were only able to obtain partial patient postcodes. Since partial postcodes do not enable a suitable deprivation measure to be derived, no deprivation analysis was conducted for the analyses.

Appointment data for each CCG area was collected and analysed once a hub became operational. Live dates varied across CCGs, and within CCGs there was variation in hub start dates. A patient residing within their CCG could use any hub services within the CCG.

Data on general practice core hour use was sourced from the GP Patient Survey (GPPS).[14] This is a cross-sectional survey of randomly selected patients from each practice within England. Patients need to be registered with a practice, have an NHS number, and be aged 18 and over to be sampled. Patients are asked questions concerning the use of primary care, perceptions of primary care, and patient demographics.

Analysis

All estimation was conducted in Stata (version 14). We assessed uptake, variations in patient demographics for different types of extended access appointments, and made comparisons of extended access appointment users to core hour users as follows:

For peer review only - http://bmjopen.bmj.com/site/about/guidelines.xhtml

(i) Uptake

Patients could book an appointment which could then be attended or not. Focussing on booked appointments alone would give an indication of the demand for the service, whilst assessments of appointments actually used shed light on actual impacts on service use. We estimated two separate sets of probability models:

1) the probability an appointment was booked, and;
2) the probability an appointment was booked and attended ('used').

Each model was estimated via probit regressions (see Supplementary Text S2 for a technical description of the methods). Regression models were estimated because there are instances of missing data meaning the dataset is not a complete picture of activity in the population. Further, regression models enable adjustment for multiple predictor variables, for example, CCG effects which may be important given the variation in schemes across CCGs (Supplementary Table 1). Probit models were chosen as these estimate differences in the probability (rate) of uptake across categories of the predictor variables. Probit regressions were preferred over logistic regressions as probit estimates give absolute percentage point differences across predictor variables (rather than relative odds-ratios) which is more informative for understanding the scale of uptake.

To assess whether uptake of the service varied across day of the week we included day of week dummies in the regression, with the estimates providing the differences in the rate of uptake relative to the base week day (Monday). This enabled a test for whether uptake was lower on particular days. To assess whether there was evidence of assimilation to the service we included calendar month dummies, if assimilation is evident the estimates on the calendar month dummies should be monotonically increasing over the period. To account for potential confounding caused by variations in the provision and delivery of services across schemes, we included CCG dummies (fixed-effects) in the regression. Robust standard errors were estimated to address concerns of heteroscedasticity and clustered at CCG. Estimates are presented as average marginal effects, which give the average percentage point effect on uptake of the variable relative to the base category across the sample.

Analyses were restricted to four CCG schemes due to the inability to differentiate whether a booked appointment had been attended in one CCG (CCG1).

(ii) Analysis of demographics of patients booking an extended access appointment

For peer review only

Our second set of analyses concentrated on booked appointments (both used and not attended) and exploited variations in service delivery to test whether there were variations in patient demographics for particular types of services. We estimated two separate sets of probability models:

1) The probability an appointment was pre-booked versus same day;
2) The probability an appointment was for a GP versus a nurse practitioner;
3) The probability an appointment was used versus not attended (DNA)

In each model the unit of analyses is the appointment, we included dummies for gender (female) and 10-year age bands of the patient booking the appointment, and day of the week of the appointment, month of the appointment, and CCG. Each model was estimated via probit regression (see Supplementary Text S2 for a technical description of the methods). For all models, estimates are presented as average marginal effects, with standard errors clustered at GP practice of the patient booking the appointment. We are unable to identify whether a person booked multiple appointments in the data and therefore assume independence of observations. Schemes that exclusively provided a certain service (e.g. only GP appointments) were excluded as a result of uptake of the alternative being necessarily zero.

Missing data on booked appointments were treated as missing at random. To evaluate whether or not missing data might have been associated with the covariates in our models (and hence potentially bias our estimates) we generated a binary missing data indicator for each appointment booked. The binary missing data indicator was coded as 1 where missing data existed for either: age, gender, appointment type, booking type, or the GP practice of the patient booking the appointment. This was regressed via a probit model against day of the week, calendar month, and CCG indicators to test whether missing data was related to particular days or months of provision and/or the unit of delivery (CCG).

(iii) Comparisons of extended access appointment users to core hour users

Finally, to inform the potential implications of the service on equity of access to primary care, the demographics of extended access patients that booked and used their appointment were compared to age and gender distributions of patients reporting using their practice within the last year from the January-March 2013 wave of the GP Patient Survey (GPPS).[14] The GPPS is a patient satisfaction survey and as such is not specifically aimed at capturing the volume of use of general practice services, however, in the absence of data on general practice use and demographics of users in England, the survey presents the most appropriate dataset for comparison. Patients reporting in the January to

For peer review only - http://bmjopen.bmj.com/site/about/guidelines.xhtml

March 2013 wave were selected to proxy for patient demographics in usual care. Later waves of the GPPS could cause potential bias as patients reporting having used primary care may have used an extended access service which had been available in areas of Greater Manchester since 2014. The GPPS sample was reduced to patients registered with a practice within one of the four CCG areas under evaluation and further reduced to those patients using primary care over the last year. These patients were determined via responses to Question 1 of the survey: "*When did you last see or speak to a GP from your GP surgery?*", with patients reporting "*In the past 3 months*", "*Between 3 and 6 months ago*", and "*Between 6 and 12 months ago*". The age groupings of these respondents did not align with the age groupings in the appointment data. As such, to ease comparisons, population pyramids are presented to give an overall feel for similarities and/or differences between the two groups of patients. The GPPS may suffer from response bias. We therefore weight practice patient use measures in this survey by the sampling weights provided with the GPPS dataset for non-response related to age and gender.[14]

Patient and public involvement

A panel of patients reviewed an earlier draft of the protocol, where feasible the protocol was adapted in light of patient feedback. The response from patients in relation to the activity analyses focussed on being able to capture variations by day of week and type of appointment, no changes in data capture was necessary following patient responses as these concerns were anticipated to be addressed in the appointment activity analyses (see Supplementary Text S3).

RESULTS

Uptake

A total of 51,806 extended access appointments were provided throughout 2016 by the five CCGs under the Greater Manchester scheme (Table 1), 41.07 appointments per 1,000 of the population (the population covered by these CCGs is 1,261,326). 2,315 of these, across two CCGs, were 'blocked' and not bookable by patients resulting in a total of 49,491 bookable appointments. CCG1 was removed from the analysis since no data on whether the patient attended was provided leaving a sample of 42,472 (49,491 appointments – 7,019 CCG1 appointments). 75.44% of 42,472 bookable appointments were booked and 65.33% were booked and attended. Provision and uptake varied across the CCGs, with CCG2 dominating provision and scale per 1,000 of the population and CCG5 having highest percentage use. The types of appointments differed across the CCG schemes (Table 2). Two of the four schemes provided both GP and nurse appointments, the other GP only, and one scheme provided

For peer review only

only pre-bookable appointments. Of all appointments booked, 53.27% were pre-booked and 81.42% were for a GP appointment.

Table 1 Volume of appointments bookable, booked, used and Did Not Attends (DNAs)

Provider	Provided (Rates per 1,000, Population (P) size)	Blocked	Available	Not booked*	Booked* (% of available)	DNAs** (% of available)	Used* (% of available)
CCG1	7,019 (23.23 per 1,000, P=302,163)	0	7,019	1,500	5,519 (78.63)		
CCG2	32,693 (143.77 per 1,000, P=227,391)	1,911	30,782	7,437	23,345 (75.84)	2,959 (9.61)	20,380 (66.21)
CCG3	3,637 (14.63 per 1,000, P=248,667)	0	3,637	1,587	2,050 (56.37)	202 (5.55)	1,848 (50.81)
CCG4	5,600 (22.92 per 1,000, P=244,325)	0	5,600	1,137	4,463 (79.70)	758 (13.54)	3,699 (66.05)
CCG5	2,857 (11.96 per 1,000, P=238,780)	404	2,453	270	2,183 (88.99)	362 (14.75)	1,820 (74.19)
All schemes	51,806 (41.07 per 1,000, P=1,261,326)	2,315	49,491	11,931	37,560 (75.89)		
Schemes CCG2 to CCG5	44,787 (46.84 per 1,000, P=956,163)	2,315	42,472	10,431	32,041 (75.44)	4,281 (10.08)	27,747 (65.33)^

CCG5 blocked are for administrative purposes.
*Booked are appointments that were used (can be either pre-booked or booked same-day).
**CCG1 did not record DNAs we assume all attended for the GM % booked and used; Missing data on DNA evident for 6 appointments in CCG2, 6 in CCG4, and 1 in CCG5.
^as a percentage of appointments where attendance was captured (42,472 appointments: 49,491 appointments minus 7,019 CCG1 appointments)

Table 2 Volume of appointments by appointment type (GP/nurse) and booking type (pre-booked/same-day)

Provider	Appointments available	GP*	% GP	Nurse*	% Nurse	Appointments booked	Pre-booked**	% Pre-booked	Same-day**	% Same-day
CCG2	30,782	24,252	78.79	6,530	21.21	23,345	9,805	42.00	13,540	58.00
CCG3	3,637	3,637	100.00	0	0.00	2,050	524	25.56	1,526	74.44
CCG4	5,600	5,255	93.84	0	0.00	4,463	2,460	55.12	2,003	44.88
CCG5	2,453	1,438	58.62	1,015	41.38	2,183	2,180	99.86	0	0.00
Total	42,472	34,582	81.42	7,545	17.76	32,041	14,969	46.72	17,069	53.27

Pre-booked appointments are appointments booked on a previous calendar date
Information on pre-booked or same-day only available for booked appointments
*GP/Nurse contains missings for CCG4 (345 appointments)
** CCG5 have 3 appointments with no data on booked time.

Figure 2 plots appointments used, appointments booked and not attended ('DNA'), and appointments not booked over the 2016 calendar year. Use appears to have improved slightly over time. 15,575 appointments (36.67%) occurred on a Saturday, and Sunday was the second most active day (5,878, 13.84%) (Figure 3). 52.93% of appointments on a Sunday were booked (46.73% used), and 75.06%

For peer review only

(63.94% used) on a Saturday. The percentage of appointments booked on weekdays was generally higher at 73.29-86.06% (65.94-75.46% booked and used) compared to weekend appointments at 52.94% (Sundays) and 75.06% (Saturdays) (46.73% (Sundays) and 63.94% (Saturdays) booked and used).

<Figure 2 here>

<Figure 3 here>

The results from the probability models of appointments booked and appointments booked and used are contained in Table 3 (unadjusted analyses are presented in Supplementary Table S3). Estimates for the probability models are interpreted as the percentage point difference in the rate of the outcome measure relative to the base category. For example, the estimate for Tuesday in the 'Probability model for appointment booked' model is interpreted as the rate of appointments booked on a Tuesday were 11.45 percentage points higher than the rate of appointments booked on Monday (the base category). The results broadly confirm the summary statistics with Monday having the lowest percentage of appointments booked of other weekdays (Tuesday-Friday), and Sunday having lowest percentage of appointments booked overall (-18.93 percentage points compared to Monday (95% CI: -35.03 to -2.83)). The results are similar for appointments used. The percentage of appointments booked and used over the year was greater for all months compared to January and appear to be greater in the second half of the year, however, the increase over time is small and inconsistent with lower rates of appointments booked and used in May, June, August and November and December compared to neighbouring months. There were differences in uptake at CCG level with all CCGs having lower percentages of appointments booked and used compared to CCG5. For comparison, Supplementary Table S4 replicates the analyses with estimation via logistic regression and confirms the same sets of estimates that are significantly different from zero in the probit regressions are significant different from one in the logistic regression. The relative size of magnitude of the estimates are also similar across models.

Table 3 Rates (%) and probability models for appointments booked and appointments booked and used

	% booked	Probability model for appointment booked (95% CI)	% booked and used	Probability model for appointment booked and used (95% CI)
**Day of week**				
**Monday (base category)**	73.29		65.94	
**Tuesday**	84.84	11.45 (8.51, 14.38)	75.46	9.60 (7.45, 11.75)

For peer review only - http://bmjopen.bmj.com/site/about/guidelines.xhtml

**Wednesday**	84.06	11.06 (5.89, 16.24)	74.14	8.45 (3.32, 13.58)
**Thursday**	86.06	12.58 (10.05 ,15.10)	73.07	7.04 (4.64, 9.44)
**Friday**	83.21	10.05 (7.12, 12.98)	69.96	4.19 (0.88, 7.51)
**Saturday**	75.06	-0.10 (-8.44, 8.24)	63.94	-3.47 (-10.97, 4.01)
**Sunday**	52.94	-18.93 (-35.03, -2.83)	46.73	-18.07 (-32.46, -3.68)
**Calendar month**				
**January (base category)**	49.52		43.87	
**February**	73.45	22.73 (13.25, 32.20)	64.33	19.80 (13.15, 26.45)
**March**	73.45	20.92 (11.80, 30.04)	63.39	17.56 (12.25, 22.88)
**April**	78.48	26.84 (11.48, 42.20)	68.31	23.42 (11.91, 34.94)
**May**	73.17	22.76 (7.90, 37.62)	63.69	19.21 (8.32, 30.10)
**June**	73.82	22.45 (5.40, 39.50)	65.31	20.45 (7.37, 33.52)
**July**	79.37	29.08 (16.09, 42.07)	68.05	24.13 (13.98, 34.27)
**August**	73.52	22.60 (4.12, 41.07)	64.00	19.11 (4.95, 33.28)
**September**	81.25	30.54 (12.66, 48.43)	69.64	25.26 (11.19, 39.32)
**October**	82.25	32.26 (15.89, 48.62)	72.12	28.11 (15.33, 40.89)
**November**	80.85	29.71 (12.79, 46.62)	69.56	24.75 (11.59, 37.91)
**December**	80.74	29.42 (6.82, 52.01)	67.40	23.01 (5.75, 40.26)
**CCG scheme**				
**CCG1***				
**CCG2**	75.48	-16.63 (-20.09, -13.17)	66.21	-10.43 (-13.17, -7.68)
**CCG3**	56.37	-32.02 (-35.24, -28.80)	50.81	-23.85 (-26.36, -21.35)
**CCG4**	79.70	-11.28 (-14.69, -7.87)	66.05	-9.97 (-12.55, -7.38)
**CCG5 (base category)**	88.99		74.19	
**Sample size**	42,472	42,472	42,472	42,472

Appointments booked are appointments booked, appointments booked and used are appointments that were booked and subsequently attended.
Probability models are probit regressions of appointment status against day of week, calendar month, and CCG scheme. Standard errors are clustered at the CCG-level.
Estimates are presented as average marginal effects which give the percentage point effect of the variable relative to the base category.
*CCG1 did not provide data to enable identification of whether a booked appointment was subsequently attended so does not feature in the analysis

For peer review only

To test whether the percentage of appointments booked and used on a Sunday improved over and above the general trend over the period we replicated our analysis with the inclusion of Sunday-calendar month interaction terms (Supplementary Table S5). The interaction terms were not significantly different from zero suggesting any change over time was similar to that observed Monday to Saturday in the data.

Analysis of demographics of patients booking an extended access appointment

The analyses concerning variations in patient demographics for particular types of appointments is limited to booked appointments (used and DNA) (Figure 1). Of the 37,560 booked appointments,

5,519 were removed due to data reporting by CCG1, giving 32,041 appointments over the remaining four CCGs. Missing data on appointment type, booking type, and patient's gender and age and GP practice amounted to a further 2,881 appointments being excluded from the analyses (Supplementary Table S6) giving a sample of 29,160 booked appointments. Missing data varied by CCG but was similar within CCGs by hub. One CCG provided 100% complete data. Missing data appears to be associated with calendar month, day of week (Wednesday and Sunday), and CCG (Supplementary Table S7).

Of the 31,154 booked appointments with complete data, 46.43% of booked appointments were pre-booked, 82.54% were for a GP, and 12.17% resulted in a patient not attending (Supplementary Table S8). 58.60% of booked appointments were made by female patients. 55.91% of users were aged under 39 (71.48% under 49). Estimates from our second set of analyses concerning patient demographics for types of appointments booked are contained in Table 4. Across all models there were significant CCG effects; this is likely to reflect variations in service delivery due to the variations in relative scale of booking type and appointment type (Table 2).

Table 4 Patient demographics by booking and appointment type

	Pre-booked v Same-day^ (95% CI)	GP v nurse^^ (95% CI)	DNA v used (95%CI)
**Gender**			
**Male (base category)**			
**Female**	-0.73 (-2.01, 0.55)	-4.29 (-6.42, -2.16)	-0.32 (-1.40, 0.77)
**Age band (years)**			
**0-9 (base category)**			
**10-19**	13.39 (10.14, 16.64)	-19.37 (-25.83, -12.91)	0.66 (-1.08, 2.40)
**20-29**	14.45 (11.28, 17.63)	-26.45 (-32.34, -20.56)	0.32 (1.65, 4.74)
**30-39**	14.53 (11.79, 17.26)	-29.45 (-35.79, -23.10)	0.97 (-0.79, 2.74)
**40-49**	15.03 (11.72, 18.35)	-31.86 (-39.36, -24.36)	-3.20 (-4.95, -1.44)
**50-59**	17.71 (14.52, 20.90)	-31.89 (-39.46, -24.32)	-7.04 (-9.07, -5.01)
**60-69**	13.25 (9.09, 17.41)	-34.25 (-43.65, -24.86)	-12.05 (-14.29, -9.81)
**70-79**	12.60 (8.78, 16.42)	-37.40 (-47.70, -27.10)	-10.20 (-13.16, -7.23)
**80-89**	14.00 (9.45, 18.55)	-41.26 (-52.96, -29.55)	-7.86 (-10.91, -4.81)
**90+**	8.83 (-5.79, 23.45)	-41.39 (-63.28, -19.49)	-
**Day of week**			
**Monday (base category)**			
**Tuesday**	20.49 (14.45, 26.53)	-0.63 (-5.05, 3.79)	-0.57 (-2.12, 0.98)

For peer review only - http://bmjopen.bmj.com/site/about/guidelines.xhtml

**Wednesday**	27.02 (23.30, 30.74)	3.32 (0.01, 6.63)	-0.59 (-2.32, 1.14)
**Thursday**	24.39 (19.00, 29.78)	-2.24 (-7.15, 2.67)	2.77 (1.33, 4.21)
**Friday**	25.59 (20.88, 30.30)	1.99 (-1.61, 5.59)	3.47 (1.62, 5.32)
**Saturday**	24.23 (18.91, 29.56)	2.32 (-2.61, 7.24)	2.22 (0.66, 3.77)
**Sunday**	24.98 (20.62, 29.33)	0.75 (-2.31, 3.81)	-0.37 (-2.10, 1.37)
**Appointment type**			
**Nurse (base category)**			
**GP**	-12.97 (-19.49, -6.45)	-	1.11 (-0.63, 2.85)
**DNA status**			
**Used (base category)**			
**DNA**	19.08 (16.11, 22.04)	1.97 (-0.39, 4.32)	-
**Booking type**			
**Same-day (base category)**			
**Pre-booked**	-	-7.46 (-12.29, -2.62)	8.48 (7.24, 9.73)
**CCG scheme**			
**CCG2**	-14.90 (-23.30, -6.50)	15.22 (7.37, 23.07)	2.94 (0.61, 5.27)
**CCG3**	-27.19 (-36.88, -17.51)	-	0.96 (-2.26, 4.18)
**CCG4**	Base	-	0.15 (-2.76, 3.05)
**CCG5**	-	Base	Base
**Sample size**	27,035	23,429	29,120

Pre-booked appointments are appointments booked on a previous calendar date
Probability models are probit regressions of appointment against day of week, calendar month, appointment characteristics, patient age and gender, and CCG scheme. Standard errors are clustered at the patient's GP practice-level. Month estimates omitted from the output but available upon request.
Estimates are presented as average marginal effects which give the percentage point effect of the variable relative to the base category.
^CCG5 had 100% pre-booked and GP appointments (n=2,125 with complete data) and was omitted from this analysis giving a sample of 29,160-2,125=27,035.
^^CCG4 had 100% GP appointments (n=3,704 with complete data) and CCG3 had 100% GP appointments (n=2,027 with complete data) and both were omitted from this analysis giving a sample of 29,160-3,704-2,027=23,429.

58.16% of pre-booked appointments were booked by females patients and 58.09% of same-day appointments were booked by female patients (Supplementary Table S9), we found no significant difference in the type of appointment booked by females compared to males (-0.73 percentage points less likely to be pre-booked compared to males, 95%CI: -2.01 to 0.55). Appointments booked for patients in age groups 10 to 19 to 80 to 89 were more likely to be pre-booked in comparison to age group 0 to 9 years. Tuesday to Sunday appointments were more likely to be pre-booked compared to Monday. GP appointments were less likely to be pre-booked in comparison to nurse appointments (-12.97 percentage points, 95%CI: -19.49 to -6.45). Patients were more likely to turn up to a same-day booked appointment than appointments that had been booked on an earlier calendar date (DNA rates were higher for pre-booked appointments by 19.08 percentage points, 95%CI: 16.11 to 22.04).

For peer review only

57.56% of GP appointments were booked by females (64.59% of nurse appointments), appointments booked by females were significantly less likely to be with a GP compared to males (-4.29 percentage, 95%CI: -6.42 to -2.16). Appointments booked were less likely to be with a GP as the age of the patient booked for the appointment increases compared to age group 0 to 9. There was limited evidence that appointments booked on specific weekdays were more or less likely to be for GP appointments. Appointments that were pre-booked were less likely to be GP appointments. Appointments that were not attended were no more or less likely to be GP appointments (1.97 percentage points higher rate for GP appointments compared to used appointments (95% CI: -0.39 to 4.32)).

58.51% of used appointments were booked by females and 59.13% of not attended (DNA) appointments were booked by females, no significant difference was found in the likelihood that appointments booked by females were not attended compared to males (-0.32 percentage points lower likelihood of not attending (95% CI: -1.40 to 0.77)). Appointments booked by age group 20 to 29 had a higher likelihood of not being attended compared to age group 0-9 though this effect is small (0.32 percentage points (95% CI: 1.65 to 4.74)). Appointments booked by age groups above the age of 40 were significantly more likely to be used compared to age group 0 to 9. Appointments booked for a Thursday, Friday or Saturday were more likely to be not attended compared those booked on a Monday.

Comparisons of extended access appointment to core hour users

To compare patient demographics of extended access users to core hour users we focus on appointments that were used. In total, 84.47% of appointment users were aged under 60 years of age (Table 5 and Figure 4). Patients booking and attending extended access appointments were compared to patients reporting attendance at their practice within the past year in the GPPS and the population registered with a practice. To better reflect the sampling frame of the GPPS (patients aged 18 and over), Figure 5 and Supplementary Table S10 provide the percentages of users in extended access appointments from age 20 plus. 80.40% of extended access appointments users aged 20 plus were under 60 years, whilst the equivalent percentage in the GPPS data lies above age 65. Users of extended access appointments tended to be younger in comparison to core hour patients with a more equal distribution of users aged between 20 to 59 compared to GPPS core hour users. For female users, the peak in the distribution of extended access users occurred at age 20-29, compared to 45 to 54 for users in the GPPS. As the age bands do not align in the datasets, these differences could not be tested, and comparisons should be treated with caution.

For peer review only

<Figure 4 here>

<Figure 5 here>

Table 5 Demographic distributions of extended access users

	Males			Females			All
**Age**	Frequency	%	Cumulative %	Frequency	%	Cumulative %	Cumulative %
**0-9**	1,436	13.53	13.16	1,387	9.26	9.26	11.03
**10-19**	1,018	9.59	23.12	1,489	9.94	19.20	20.82
**20-29**	1,446	13.62	36.74	2,897	19.33	38.53	37.78
**30-39**	1,575	14.84	51.58	2,513	16.77	55.30	53.75
**40-49**	1,617	15.23	66.81	2,435	16.25	71.55	69.58
**50-59**	1,690	15.92	82.73	2,123	14.17	85.72	84.47
**60-69**	1,144	10.78	93.51	1,273	8.50	94.22	93.91
**70-79**	481	4.53	98.04	624	4.16	98.38	98.23
**80-89**	197	1.86	99.90	215	1.43	99.81	99.84
**90+**	12	0.11	100.00	28	0.19	100.00	100.00
**Total**	10,616	100.00		14,984	100.00		

DISCUSSION

Principal findings

Our primary analyses concerned the proportion of extended access appointments used. We found evidence of spare capacity in the extended access service and limited evidence that use improved over the calendar year. The proportion of appointments used was lowest on Sundays. Secondary analyses investigated types of appointments booked (both booked and used and booked and not used) and the patient demographics of users of the service. There were variations in types of patients booking particular types of extended access appointments. Pre-booked appointments were relatively more likely amongst age groups 10 to 89 compared to age group 0 to 9. GP appointments were increasingly less likely to be booked with age. Appointments booked on Thursday to Saturday were relatively more likely to result in non-attendance compared to Mondays. Pre-booked appointments were less likely to be with a GP and more likely to result in non-attendance compared to same-day appointments. In comparison to core hour patients, we found some evidence that patients using extended access appointments were of a younger age relative to patients using primary care services before the scheme. The most obvious difference was found in females aged 20-29 who accounted for almost a quarter of all female extended access appointments users (aged 20+).

For peer review only

Strengths and weaknesses

The study has a number of strengths and weaknesses. Strengths lie in the unique dataset which enabled a detailed look at uptake and users of the service and how this varied with types of appointments provided. In addition, our results are focussed on several Greater Manchester schemes offering variation in delivery which enables comparisons of different service models. However, weaknesses are that the findings may not be generalizable to other areas that may provide an extended access service under alternative specifications and/or populations than those detailed in this study. Missing data on patient age, gender, and registered practice resulted in a loss of 8.99% of appointments and this was found to be vary with day of week, calendar month and CCG. Although data issues were flagged by the research team, we could not enforce areas to provide complete data. We were unable to obtain patient deprivation/ethnicity characteristics to enrich understanding of service use. We were also unable to identify whether there are repeat patients in the data, this may bias the comparison of users of the extended access service to those in core hours if repeated use is associated with gender and/or age. We were not able to identify whether an appointment was cancelled and as such, we assume this was subsequently made available. The GPPS comparison is particularly flawed because the measure of core hour use is self-reported and thus may be affected by recall error, children are not included in the survey and age bands did not match with those obtained for appointment data. Finally, access depends on a range of factors, and without further details on CCG provision related to awareness campaigns and buy-in from providers, we can only distinguish such effects by the CCG fixed-effects adjustment factor in our models.

Implications

The presence of spare capacity for a service seeking to improve access to meet excess demand in core hours raises the question of whether or not extended access appointments really are improving access to primary care. However, of the appointments that were used, our findings suggest that the service may be either i) meeting previously unmet need for younger age groups, ii) meeting previously inappropriately met need should these patients have proceeded to use alternative health services such as emergency departments at hospital, or iii) diverting demand for core hours for this age group, thereby potentially reducing pressure in core hours. It is likely that a mixture of these factors are playing a role. We find 46.43% of appointments used were pre-booked which may signify a large proportion of patients are using the service for non-urgent health needs which may suggest i) or iii) plays an important role. 53.57% of appointments booked and used were same-day appointments which may suggest ii) may also be an important factor. More research is needed to understand the relative scale of these factors, such information would give important insights into the value and

For peer review only

efficiency of the service. These factors are on the assumption that patients accessing the service had a need for seeking health care services, should patients referred to the service not have a need for healthcare then service use may reflect supply induced demand.

The reasoning behind spare capacity in the scheme may be informed by three domains of access: availability, acceptability, affordability.[15] These domains of access highlight the complex contributory factors at play in access, involving both patient and practitioner. The extended access schemes provide additional *availability* in the sense of new appointments. However, new appointments alone may not be sufficient to improve access, should patients or practitioners not be prepared to *accept* the service (this could be due to cultural factors and/or patient preferences for example) or should patients not be aware of the service. A process evaluation of the service is contained in the evaluation report of the service and highlights the variation in approaches taken to communicate the service to patients.[13] Identification is needed of whether poor rates of use are due to a lack of patient demand or, for of particular concern in areas where practice referrals are made, whether a lack of receptionist/practitioner buy-in is influencing patient (and receptionist) awareness of the service (see for example: Healthwatch Manchester, 2017[16]). Policy needs to consider these additional aspects of access to facilitate use and adapt provision accordingly.

Cost-effectiveness analysis seeks to understand whether a service is an efficient use of healthcare resources, this is performed by identifying the opportunity cost of delivering the service (the net benefits of services that could have been provided by allocating the resources elsewhere). Whilst we do not compare the service to a counterfactual use of resources in this study (such as expanding core hours), our findings shed light on a number of aspects relevant for determining cost-effectiveness of the extended access service. First, it is likely that extended access services will result in some positive measure of benefit to patients. Second, a large proportion of extended appointments are pre-booked, suggesting a large amount of activity in the service is for non-urgent care. As such, existing evaluations, that have focussed on emergency care implications, paint only a partial picture of the impacts of extended access. Third, only 65.33% of appointments available were booked and used which raises concerns relating to how efficient the service is. Providers seeking to determine the types of services available under extended access schemes may consider several factors to more efficiently provide the service in relation to our results : (i) whilst the proportion of appointments used was lowest on a Sunday, the volume of appointments used was similar to those during the week, this suggests the proportion of appointments used on a Sunday can be improved by reducing the volume of Sunday appointments (ii) a completely pre-bookable service may reduce access for the very young and result

For peer review only - http://bmjopen.bmj.com/site/about/guidelines.xhtml

in higher rates of appointments booked and not attended. (iii) nurse appointments may help address issues of staffing. DNAs were no greater for this type of appointment. However, GP appointments were relatively more concentrated amongst age group 0 to 9 and males suggesting a potential need for a GP service to address the needs of younger age and male patients.

The evaluation of the national pilots have found uptake of 71% with reportedly lower uptake on weekends, particularly on Sundays.[8] A more detailed evaluation of three schemes under the PMCF pilots found uptake varied by type of service (same day or pre-bookable) available.[11] Weekend uptake was higher in a scheme providing pre-bookable and same-day appointments (87% on Saturdays and 78% on Sundays) compared to two schemes providing only urgent/same-day appointments (25% and 18% and 48% and 41%). The demographics of the scheme providing both pre-bookable and same-day appointments comprised 3% aged under 5, and 57% aged between 20 and 65. The findings in this paper found schemes with pre-bookable and same-day appointments had a lower rate of booked appointments than schemes with only same-day appointments (the proportion of appointments booked in CCG2 to CCG4 are lower than that of CCG5 which provided only same-day appointments (Table 2)). We also found uptake was lower on Sundays (also in line with the national evaluation). However, in contrast we found age group 20-29 to be the most prevalent group of patients using the service (16.96%) and a higher proportion of patients from the 20-69 age group (73.09% compared to 57% aged 20-65). These differences may be due to differences in the patient population, breadth of week covered or scale of the schemes (the relevant scheme evaluated by Windrum et al (2017)[11] was much smaller with three practices and one hub). Evaluations of patient demographics using extended access appointments for urgent care in Sheffield, UK, found users were predominantly female (60.0%) and aged under 60 (85.5%), with 19.05% aged under 5 and 29.35% aged under 16 and whilst ethnicity data had a high proportion of missing data, deprivation was captured finding users from deprived areas dominated use.[12] These findings may reflect the urgent care service delivered in comparison to the current study which offered both same-day and pre-bookable appointments.

Future work

We have not been able to identify implications for core hours or assess whether deprivation or ethnicity are a leading factor of appointment use. Further research is needed to investigate the impacts on demand for core hours services and for deprived populations to shed light on potential implications for access.

For peer review only - http://bmjopen.bmj.com/site/about/guidelines.xhtml

An assessment of the types of treatments and conditions being presented at extended access appointments may help inform impacts of the service on patient health and the scale of potential supply induced demand.

Given the pilot nature of these schemes, uptake may have also reflected supply-side buy-in, and reservations concerning promotion of the schemes may have arisen due to uncertainty in future funding. Future evaluations of uptake, once clarity is provided on long-term securement of the extended access service, may help identify the scale of this possibility.

Competing interests statement:
All authors have completed the ICMJE uniform disclosure form at www.icmje.org/coi_disclosure.pdf and have the following competing interests: funding support from NIHR and from Greater Manchester Health and Social Care Partnership for this work; WW reports grant funding for other work from the Department of Health Policy Research Programme and NIHR. No other relationships or activities that could appear to have influenced the submitted work are declared.

Contributors:
WW is guarantor for the work. WW cleaned the data, conceived and conducted the analysis, interpreted the data and composed the first draft of the work.
LA critically revised the manuscript, participated in the design of the study, and approved the final version to be published.
PN contributed to interpretation of data, revised the initial and subsequent drafts of the paper critically for content and approved the final version to be published.
CO cleaned the activity data in preparation for the analyses, assisted in interpretation of the findings, and approved the final version to be published.
NR contributed to interpretation of data, revised earlier drafts of the paper, and approved the final version to be published.
KR contributed to interpretation of data, revised the initial and subsequent drafts of the paper critically for content and approved the final version to be published.
DH designed and led the overall project, was directly involved in interpretation of data and approved the final version to be published.
All authors had full access to all of the data (including statistical reports and tables) in the study and can take responsibility for the integrity of the data and the accuracy of the data analysis.

Patient consent:
Patient consent was not required as the study reports no identifiable patient data.

Ethics statement:
The study was reviewed by the University of Manchester's internal University Research Ethics Committee and approved under the low risk procedure and thus did not require UREC approval (ID: AMBS/16/05). The study involves anonymised administrative data and did not impact on the type of care patients received, consent by patients using the service was not required.

Funding statement:

For peer review only

This project was funded by the National Institute for Health Research Collaboration for Leadership in Applied Health Research and Care (NIHR CLAHRC) Greater Manchester and the Greater Manchester Health and Social Care Partnership. The NIHR CLAHRC Greater Manchester is a partnership between providers and commissioners from the NHS, industry and the third sector, as well as clinical and research staff from the University of Manchester. The views expressed in this article are those of the author(s) and not necessarily those of the NHS, NIHR or the Department of Health and Social Care.

Transparency declaration:

WW affirms that the manuscript is an honest, accurate, and transparent account of the study being reported; that no important aspects of the study have been omitted; and that any discrepancies from the study as planned have been explained.

Copyright statement:

The Corresponding Author has the right to grant on behalf of all authors and does grant on behalf of all authors, *a worldwide licence* to the Publishers and its licensees in perpetuity, in all forms, formats and media (whether known now or created in the future), to i) publish, reproduce, distribute, display and store the Contribution, ii) translate the Contribution into other languages, create adaptations, reprints, include within collections and create summaries, extracts and/or, abstracts of the Contribution, iii) create any other derivative work(s) based on the Contribution, iv) to exploit all subsidiary rights in the Contribution, v) the inclusion of electronic links from the Contribution to third party material where-ever it may be located; and, vi) licence any third party to do any or all of the above.

Data sharing statement:

Appointment data: No additional data available.

GP Patient Survey: The GP Patient Survey data are available at https://gp-patient.co.uk/SurveysAndReports

Reporting statement:

A STROBE checklist for observational studies has been adhered to with checklist provided as supplementary material.

For peer review only

References:

[1] World Health Organisation. The world health report: health systems financing: the path to universal coverage. [cited 2010]. Available from: http://www.who.int/whr/2010/en/ [Accessed 01/11/2018]

[2] Schoen C, Osborn R, Doty MM, Bishop M, Peugh J, Murukutla N. Towards Higher-Performance Health Systems: Adults' Health Care Experiences In Seven Countries, 2007. *Health Affairs* 2007;26(6):w717-34.

[3] Schoen C, Osborn R, Squires D, Doty MM. Access, Affordability, And Insurance Complexity Are Often Worse In The United States Compared To Ten Other Countries. *Health Affairs* 2013;32(12):2205-2215.

[4] Kraaijvanger N, van Leeuwen H, Rijpsma D, Edwards M. Motives for self-referral to the emergency department: a systematic review of the literature. *BMC Health Serv Res* 2016;16:685

[5] NHS England. General Practice Forward View. [cited 2016 April]. Available from: https://www.england.nhs.uk/wp-content/uploads/2016/04/gpfv.pdf [Accessed 01/11/2018]

[6] NIHR CLAHRC Greater Manchester. NHS Greater Manchester Primary Care Demonstrators Evaluation. [cited 2015 June]. Available from: https://www.research.manchester.ac.uk/portal/files/32297227/FULL_TEXT.PDF [Accessed 01/11/2018]

[7] Greater Manchester Combined Authority. Delivering Integrated Care across Greater Manchester: The Primary Care Contribution. Our Primary Care Strategy 2016-2021. [cited 2016]. Available from: http://www.gmhsc.org.uk/assets/GMHSC-Partnership-Primary-Care-Strategy.pdf [Accessed 01/11/2018]

[8] MacDonald, SQW. Prime Minister's Challenge Fund: Improving Access to General Practice: Second Evaluation Report to September 2015. [cited 2016]. Available from: https://www.england.nhs.uk/wp-content/uploads/2016/10/gp-access-fund-nat-eval-wave1-sml.pdf [Accessed 01/11/2018]

[9] Dalton P, Pathania V. Can increased primary care access reduce demand for emergency care? Evidence from England's 7-day GP opening. *J Health Econ* 2016;49:193-208.

[10] Whittaker W, Anselmi L, Kristensen S, et al. Associations between extending access to primary care and emergency department visits: a difference-in-differences analysis. *PLoS Med* 2016;13(9):e1002113

[11] Windrum P, Guinery J, Siebert P, et al. CHILL Evaluation of 7-Day GP Access. [cited 2017 Jan 1]. Available from: https://www.nottingham.ac.uk/business/businesscentres/chill/documents/chill-7-day-gp-access-report.pdf [Accessed 01/11/2018]

[12] Kelly SJ, Piercy H, Ibbotson R, et al. Who attends out-of-hours general practice appointments? Analysis of a patient cohort accessing new out-of-hours units. *BMJ Open* 2018. doi:10.1136/bmjopen-2017-020308

For peer review only

[13] NIHR CLAHRC Greater Manchester. GM Primary Care 7-Day Access Evaluation. [cited 2017 March]. Available from: https://www.clahrc-gm.nihr.ac.uk/media/Resources/OHC/GM-Primary-Care-7-day-access-report-evaluation.pdf [Accessed 29/05/2019]

[14] Ipsos MORI. Technical Annex for the GP Patient Survey 2012-2013 Annual Report. [cited 2013 June 13]. Available from: https://gp-patient.co.uk/downloads/archive/2013/June/June%202013%20Technical%20Annex.pdf [Accessed 01/11/2018]

[15] McIntyre D, Thiede M, Birch S. Access as a policy-relevant concept in low- and middle-income countries. *Health Econ Policy Law* 2009;4(2):179-193

[16] Healthwatch Manchester. Week spot? Review of Access to the 7 Day GP Service. [cited 2017 November]. Available from: https://d2jlsms9zhgfok.cloudfront.net/2017/12/07142053/Week-Spot-Access-to-7-Day-GP-Service-Review.pdf [Accessed 01/11/2018]

Figures

Figure 1 Flow chart of population coverage

*There are 13 appointments booked that contained no information on attendance

Figure 2 Volume and percentage of appointments used, DNA and not booked by month, all schemes

Excludes CCG1 activity
Used – appointments booked and attended
DNA - appointments booked but not attended

Figure 3 Volume and percentage of appointments used, DNA and not booked by day of week, all schemes

Excludes CCG1 activity
Used – appointments booked and attended
DNA - appointments booked but not attended

Figure 4 Profile of extended appointment users by age and gender

Figure 5 Profile of extended appointment users and core hours users, by age and gender

GPPS: GP Patient Survey (January-March 2013 wave)
GPPS data are patients reporting having spoken to or seen a GP from their practice in the past 12 months

For peer review only - http://bmjopen.bmj.com/site/about/guidelines.xhtml

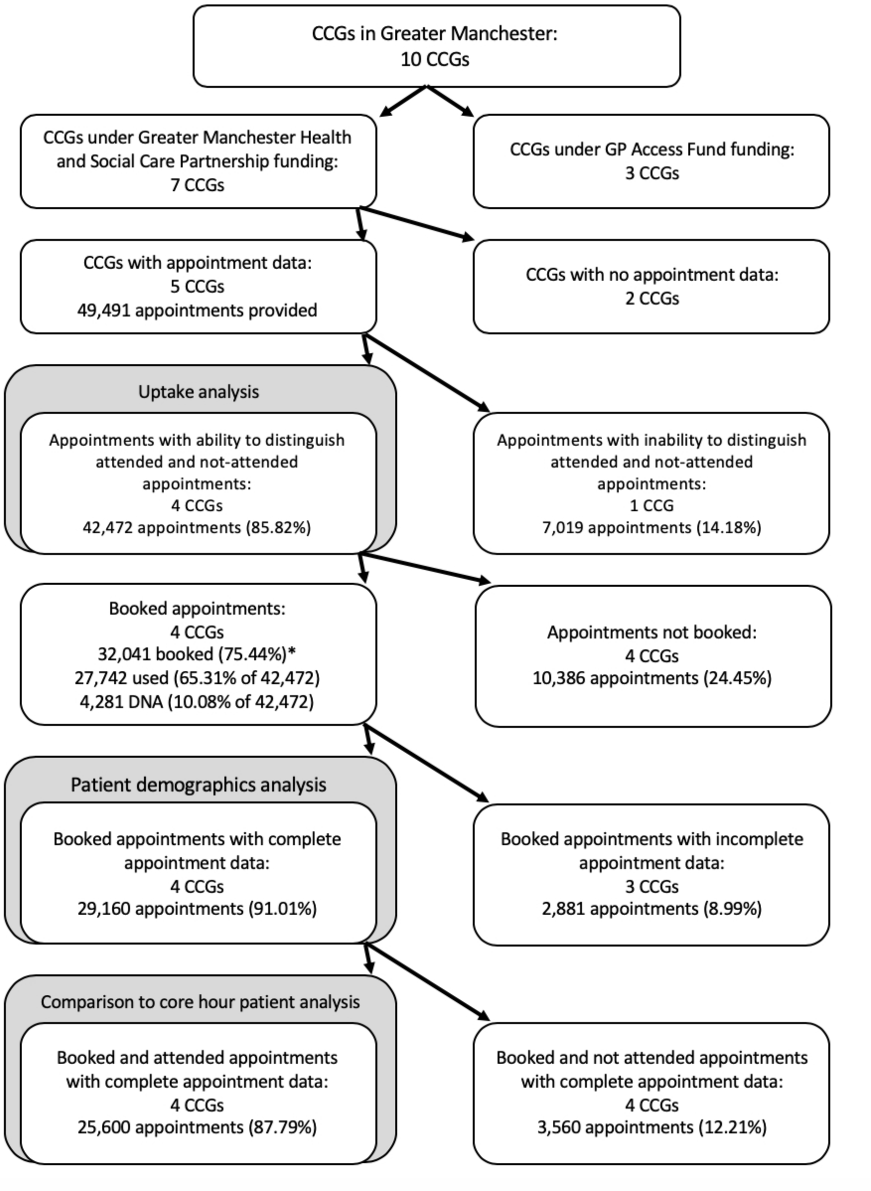

Figure 1 Flow chart of population coverage

156x213mm (144 x 144 DPI)

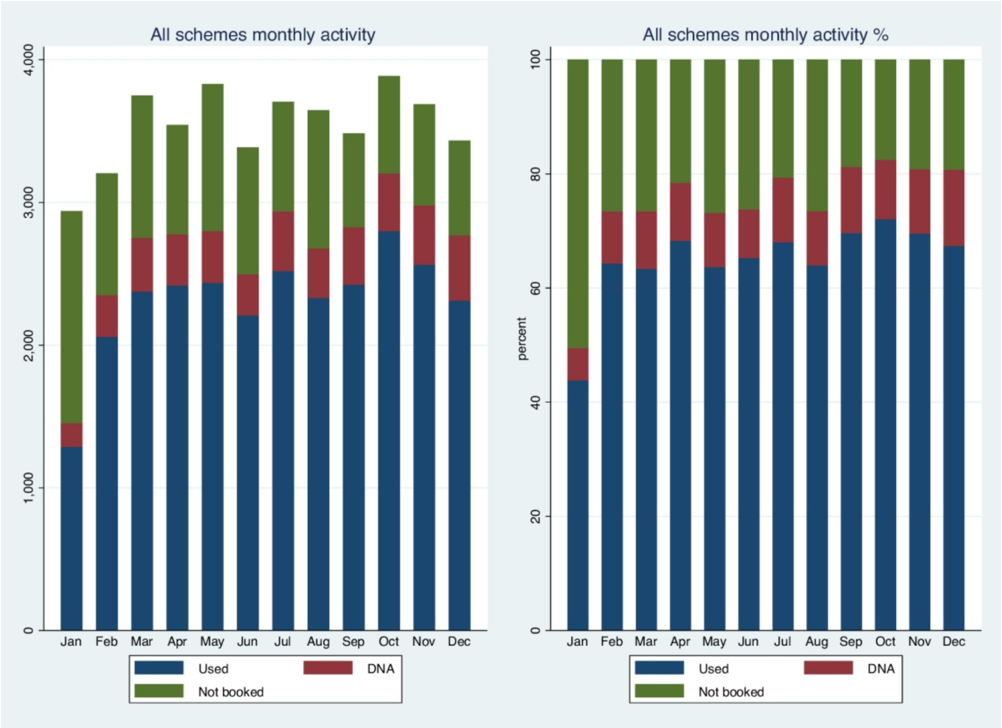

Figure 2 Volume and percentage of appointments used, DNA and not booked by month, all schemes

138x101mm (300 x 300 DPI)

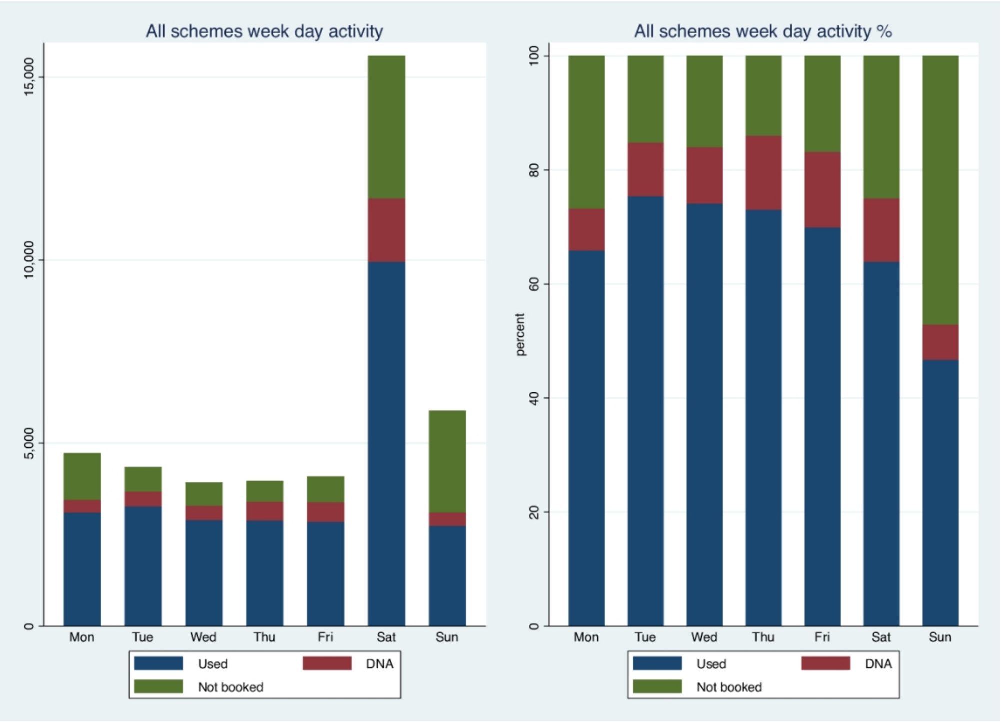

Figure 3 Volume and percentage of appointments used, DNA and not booked by day of week, all schemes

142x102mm (300 x 300 DPI)

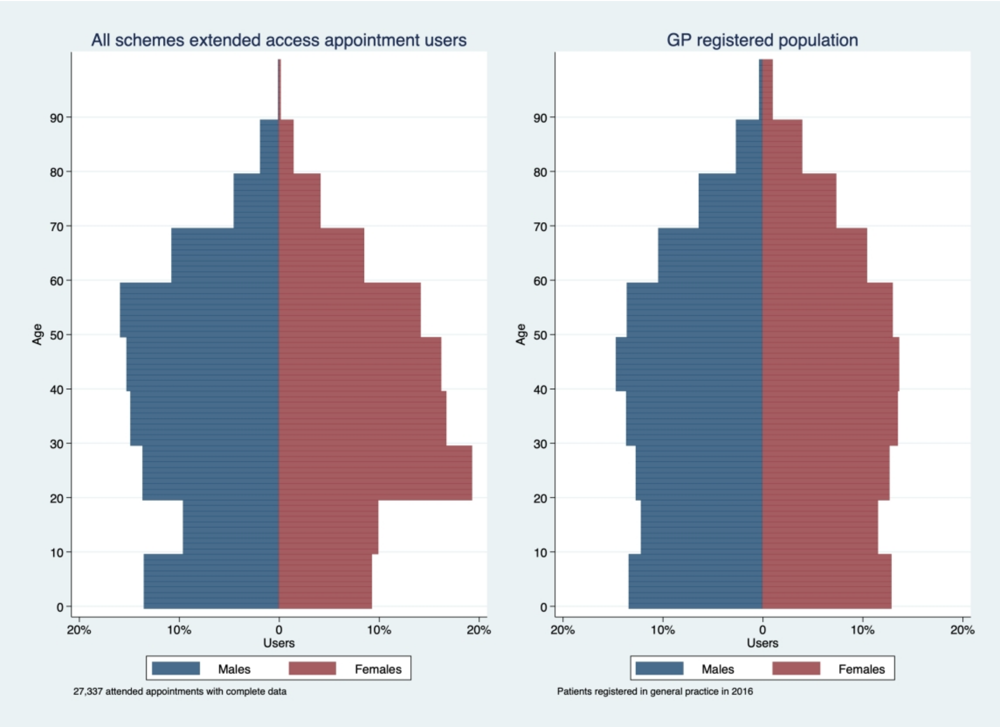

Figure 4 Profile of extended appointment users by age and gender

549x399mm (144 x 144 DPI)

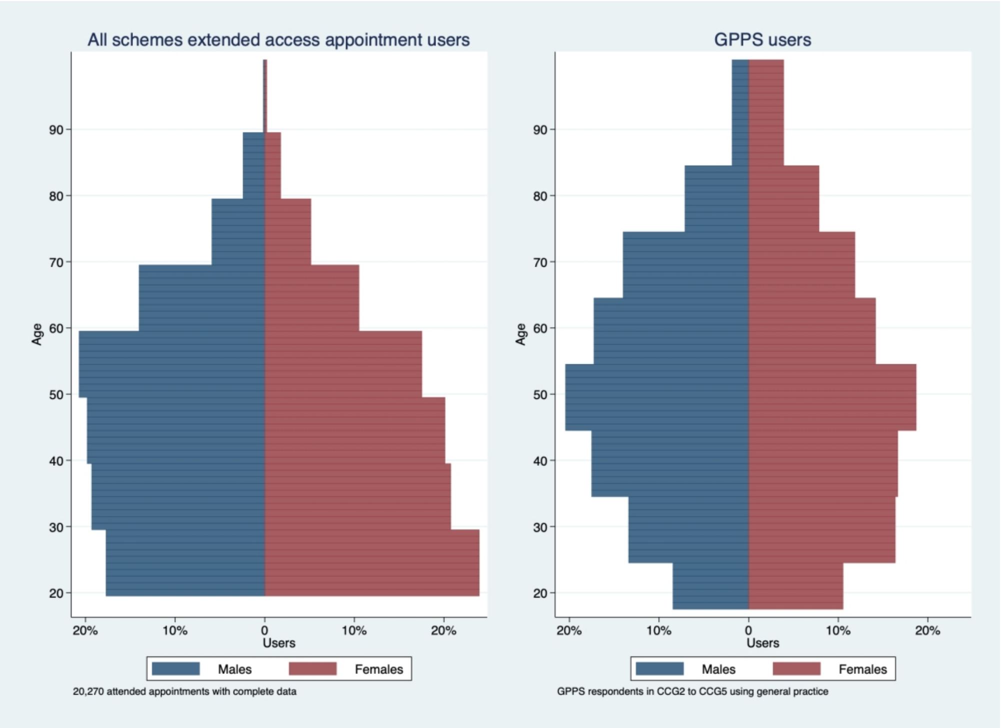

Figure 5 Profile of extended appointment users and core hours users, by age and gender

366x266mm (144 x 144 DPI)

Supplementary Table S1 Summary of 7 Day Access Implementation by Area

Clinical Commissioning Group	Live date (2016)	Service	Self-referral	Monday-Friday opening	Saturday opening	Sunday opening
**CCG1**						
**Hub 1**	2 April	GP and nurse	No	No	09:00-13:00	10:00-13:00
**Hub 2**	2 April	GP and nurse	No	No	09:00-13:00	10:00-13:00
**CCG2**						
**Hub 1**	11 January	GP and nurse	Yes	18:30-21:00	08:00-18:00	10:00-13:00
**Hub 2**	1 January	GP and nurse	Yes	18:30-21:00	08:00-18:00	10:00-13:00
**Hub 3**	2 January	GP and nurse	Yes	18:30-21:00	08:00-18:00	10:00-13:00
**Hub 4**	2 January	GP and nurse	Yes	18:30-21:00	08:00-18:00	10:00-13:00
**CCG3**						
**Hub 1**	5 January	GP only	Yes	18:30-20:00	10:00-14:00	10:00-14:00
**Hub 2**	10 August	GP only	Yes	18:30-20:00	10:00-14:00	No
**CCG4**						
**Hub 1**	3 January	GP only	No	18:30-20:00	09:00-11:45	09:00-12:00
**Hub 2**	30 January	GP only	No	18:15-20:00 (no Thursday or Friday)	09:00-11:45	09:00-11:45
**Hub 3**	25 February	GP only	No	18:30-20:00	09:00-11:45	09:00-12:00
**CCG5**						
**Hub 1**	16 January	GP and nurse	No	No	09:00-13:00	No
**Hub 2**	16 January	GP and nurse	No	No	09:00-13:00	No

For peer review only - http://bmjopen.bmj.com/site/about/guidelines.xhtml

Supplementary Table S2 Activity Capture Minimum Dataset

Data requested from Clinical Commissioning Group	Format
Volume of appointments available	
Date	DD/MM/YYYY
Time of appointment	HH:MM
Discipline	GP/Nurse
Planned/pre-booked vs same day/urgent	Pre-booked/Same day
Method of appointment (face-to-face/phone)	Face-to-face/phone
Volume of appointments booked and booked and attended	
Date	DD/MM/YYYY
Time of appointment	HH:MM
Discipline	GP/Nurse
Age	10 year age bands: 0-9 to 90+
Gender	Male/Female
Registered GP practice	Practice code identifier
Postcode (first part)	First half
Planned/pre-booked vs same day/urgent	Pre-booked/Same day
Method of appointment (face-to-face/phone)	Face-to-face/phone
Method of appointment (face-to-face/phone)	Face-to-face/phone

For peer review only

Supplementary Table S3 Rates of appointment use

	Unadjusted appointment booked (95% CI)	Unadjusted appointment booked and used (95% CI)
**Day of week**		
**Monday (base category)**		
**Tuesday**	11.54 (7.90, 15.19)	9.52 (6.51, 12.53)
**Wednesday**	10.77 (3.57, 17.76)	8.20 (1.75, 14.65)
**Thursday**	12.76 (9.45, 16.08)	7.14 (3.96, 10.31)
**Friday**	9.92 (7.19, 12.65)	4.02 (0.62, 7.42)
**Saturday**	1.76 (-6.96, 10.49)	-2.00 (-9.26, 5.27)
**Sunday**	-20.35 (-36.51, -4.19)	-19.21 (-34.36, -4.05)
**Calendar month**		
**January (base category)**		
**February**	23.93 (15.15, 32.71)	20.46 (13.88, 27.04)
**March**	23.93 (17.22, 30.64)	19.52 (15.68, 23.37)
**April**	28.96 (14.72, 43.19)	24.44 (13.63, 35.25)
**May**	23.65 (11.06, 36.24)	19.82 (10.34, 29.29)
**June**	24.29 (8.32, 40.26)	21.43 (8.97, 33.89)
**July**	29.84 (17.46, 42.23)	24.18 (14.12, 34.24)
**August**	23.99 (6.93, 41.06)	20.12 (6.88, 33.37)
**September**	31.72 (13.78, 49.67)	25.77 (11.45, 40.09)
**October**	32.99 (17.24, 48.74)	28.24 (15.93, 40.56)
**November**	31.32 (14.84, 47.80)	25.69 (12.83, 38.54)
**December**	31.22 (10.11, 52.32)	23.52 (7.00, 40.04)
**CCG scheme**		
**CCG1***		
**CCG2**	-16.17 (-16.17, -16.17)	-8.46 (-8.46, -8.46)
**CCG3**	-32.83 (-32.83, -32.83)	-23.02 (-23.02, -23.02)
**CCG4**	-12.17 (-12.17, -12.17)	-8.62 (-8.62, -8.62)
**CCG5 (base category)**		
**Sample size**	42,472	42,472

Unadjusted estimates obtained via probit regression of appointment status against each set of dummies (estimated separately): day of week, calendar month, and CCG scheme.
Estimates are presented as average marginal effects which give the percentage point effect (when multiplied by 100) of the variable relative to the base category.
*CCG1 did not provide data to enable identification of whether a booked appointment was subsequently attended so does not feature in the analysis

For peer review only

Supplementary Table S4 Comparisons of probit and logit probability model estimates

	Probit model: Probability model for appointment booked^ (95% CI)	Logit model: Probability model for appointment booked^^ (95% CI)	Probit model: Probability model for appointment used^ (95% CI)	Logit model: Probability model for appointment used^^ (95% CI)
**Day of week**				
**Monday (base category)**				
**Tuesday**	11.45 (8.51, 14.38)	2.16 (1.72, 2.71)	9.60 (7.45, 11.75)	1.63 (1.48, 1.80)
**Wednesday**	11.06 (5.89, 16.24)	2.08 (1.44, 3.01)	8.45 (3.32, 13.58)	1.53 (1.18, 1.98)
**Thursday**	12.58 (10.05 ,15.10)	2.38 (2.02, 2.80)	7.04 (4.64, 9.44)	1.42 (1.27, 1.58)
**Friday**	10.05 (7.12, 12.98)	1.91 (1.56, 2.34)	4.19 (0.88, 7.51)	1.23 (1.05, 1.43)
**Saturday**	-0.10 (-8.44, 8.24)	0.99 (0.62, 1.57)	-3.47 (-10.97, 4.01)	0.85 (0.61, 1.20)
**Sunday**	-18.93 (-35.03, -2.83)	0.41 (0.20, 0.85)	-18.07 (-32.46, -3.68)	0.46 (0.25, 0.85)
**Calendar month**				
**January (base category)**				
**February**	22.73 (13.25, 32.20)	2.98 (2.26, 3.94)	19.80 (13.15, 26.45)	2.33 (1.85, 2.93)
**March**	20.92 (11.80, 30.04)	2.70 (2.06, 3.55)	17.56 (12.25, 22.88)	2.11 (1.76, 2.52)
**April**	26.84 (11.48, 42.20)	3.91 (1.99, 7.68)	23.42 (11.91, 34.94)	2.78 (1.74, 4.43)
**May**	22.76 (7.90, 37.62)	3.01 (1.65, 5.50)	19.21 (8.32, 30.10)	2.28 (1.41, 3.49)
**June**	22.45 (5.40, 39.50)	2.96 (1.43, 6.13)	20.45 (7.37, 33.52)	2.41 (1.42, 4.08)
**July**	29.08 (16.09, 42.07)	4.43 (2.75, 7.13)	24.13 (13.98, 34.27)	2.86 (1.94, 4.22)
**August**	22.60 (4.12, 41.07)	2.97 (1.33, 6.63)	19.11 (4.95, 33.28)	2.26 (1.27, 4.02)
**September**	30.54 (12.66, 48.43)	4.89 (2.07, 11.57)	25.26 (11.19, 39.32)	3.02 (1.68, 5.47)
**October**	32.26 (15.89, 48.62)	5.60 (2.61, 12.01)	28.11 (15.33, 40.89)	3.51 (2.06, 5.98)
**November**	29.71 (12.79, 46.62)	4.67 (2.15, 10.14)	24.75 (11.59, 37.91)	2.96 (1.72, 5.10)
**December**	29.42 (6.82, 52.01)	4.62 (1.52, 14.08)	23.01 (5.75, 40.26)	2.73 (1.32, 5.64)
**CCG scheme**				
**CCG1***				
**CCG2**	-16.63 (-20.09, -13.17)	0.35 (0.30, 0.40)	-10.43 (-13.17, -7.68)	0.61 (0.54, 0.68)
**CCG3**	-32.02 (-35.24, -28.80)	0.14 (0.13, 0.15)	-23.85 (-26.36, -21.35)	0.32 (0.30, 0.35)
**CCG4**	-11.28 (-14.69, -7.87)	0.46 (0.38, 0.54)	-9.97 (-12.55, -7.38)	0.62 (0.55, 0.69)
**CCG5 (base category)**				
**Sample size**	42,472	42,472	42,472	42,472

Appointments booked are appointments booked, appointments used are appointments that were booked and subsequently attended.
Probability models are probit and logit regressions of appointment status against day of week, calendar month, and CCG scheme.
Standard errors are clustered at the CCG-level.
^Estimates are presented as average marginal effects which give the percentage point effect of the variable relative to the base category.
^^Estimates are presented as odds-ratios which give the odds relative to the base category.
*CCG1 did not provide data to enable identification of whether a booked appointment was subsequently attended so does not feature in the analysis

For peer review only

Supplementary Table S4 Probability models for appointment use with Sunday interaction with calendar month

	Appointment booked (95% CI)	Appointment used (95% CI)
**Day of week (base: Monday)**		
**Tuesday**	0.430 0.320,0.539)	0.288 (0.237,0.339)
**Wednesday**	0.410 (0.232,0.588)	0.249 (0.104,0.394)
**Thursday**	0.481 (0.408,0.553)	0.204 (0.144,0.265)
**Friday**	0.367 (0.268,0.466)	0.119 (0.031,0.207)
**Saturday**	-0.010 (-0.278,0.258)	-0.098 (-0.303,0.108)
**Sunday**	-0.560 (-0.950,-0.170)	-0.659 (-1.127,-0.191)
**Calendar month (base: January)**		
**February**	0.729 (0.580,0.878)	0.562 (0.436,0.687)
**March**	0.563 (0.377,0.748)	0.418 (0.313,0.524)
**April**	0.764 (0.437,1.091)	0.591 (0.377,0.805)
**May**	0.599 (0.298,0.900)	0.451 (0.248,0.655)
**June**	0.684 (0.324,1.044)	0.552 (0.297,0.807)
**July**	0.929 (0.755,1.103)	0.636 (0.488,0.785)
**August**	0.664 (0.266,1.061)	0.493 (0.211,0.775)
**September**	0.938 (0.515,1.362)	0.650 (0.373,0.928)
**October**	0.986 (0.604,1.367)	0.708 (0.434,0.981)
**November**	0.889 (0.495,1.283)	0.622 (0.341,0.903)
**December**	0.895 (0.311,1.479)	0.578 (0.195,0.961)
**Sunday*Calendar month**		
**February**	-0.407 (-0.685,-0.128)	-0.229 (-0.475,0.017)
**March**	0.238 (-0.036,0.512)	0.334 (0.026,0.642)
**April**	0.213 (-0.502,0.929)	0.260 (-0.486,1.006)
**May**	0.307 (-0.420,1.033)	0.368 (-0.349,1.086)
**June**	-0.205 (-0.874,0.464)	-0.044 (-0.708,0.619)
**July**	-0.197 (-1.042,0.648)	0.107 (-0.670,0.884)
**August**	-0.078 (-0.841,0.686)	0.084 (-0.670,0.838)
**September**	0.013 (-0.693,0.719)	0.214 (-0.573,1.002)
**October**	0.131 (-0.439,0.701)	0.389 (-0.198,0.975)
**November**	0.099 (-0.522,0.721)	0.303 (-0.290,0.895)
**December**	-0.001 (-0.984,0.981)	0.290 (-0.573,1.154)
**CCG scheme* (base: CCG5)**		
**CCG2**	-0.590 (-0.703,-0.477)	-0.298 (-0.373,-0.223)
**CCG3**	-1.134 (-1.214,-1.053)	-0.678 (-0.742,-0.614)
**CCG4**	-0.395 (-0.497,-0.292)	-0.281 (-0.347,-0.214)
**Constant**	0.516 (-0.046,1.077)	0.210 (-0.205,0.625)
**Sample size**	42,472	42,472

Appointments booked are appointments booked, appointments booked and used are appointments that were booked and subsequently attended.

For peer review only

Probit regression of appointment status against day of week, calendar month, Sunday interaction with calendar month, and CCG scheme. Standard errors are clustered at the CCG-level.
Estimates are not presented as average marginal effects due to the inability to identify the marginal effect of an interaction term.
*CCG1 did not provide data to enable identification of whether a booked appointment was subsequently attended so does not feature in the analysis

For peer review only

Supplementary Table S6 Missing appointment data for booked appointments

CCG		Total booked	Missing patient gender	Missing patient age	Missing appointment type	Missing booking type	Missing patient's GP practice	Any missing appointment data
**CCG2**		23,345	73 (0.31%)	246 (1.05%)	0 (0.00%)	0 (0.00%)	1,902 (8.15%)	2,041 (8.74%)
	**Hub 1**	5,220	7 (0.13%)	76 (1.46%)	0 (0.00%)	0 (0.00%)	416 (7.97%)	461 (8.83%)
	**Hub 2**	6,435	37 (0.57%)	66 (1.03%)	0 (0.00%)	0 (0.00%)	504 (7.83%)	545 (8.47%)
	**Hub 3**	5,229	16 (0.31%)	70 (1.34%)	0 (0.00%)	0 (0.00%)	470 (8.99%)	493 (9.43%)
	**Hub 4**	6,461	13 (0.20%)	34 (0.53%)	0 (0.00%)	0 (0.00%)	512 (7.92%)	542 (8.39%)
**CCG4**		4,463	227 (5.09%)	227 (5.09%)	345 (7.73%)	0 (0.00%)	457 (10.24%)	759 (17.01%)
	**Hub 1**	1,878	90 (4.79%)	90 (4.79%)	141 (7.51%)	0 (0.00%)	225 (11.98%)	355 (18.90%)
	**Hub 2**	1,060	60 (5.66%)	60 (5.66%)	83 (7.83%)	0 (0.00%)	104 (9.81%)	176 (16.60%)
	**Hub 3**	1,525	77 (5.05%)	77 (5.05%)	121 (7.93%)	0 (0.00%)	128 (8.39%)	228 (14.95%)
**CCG5**		2,183	11 (0.50%)	51 (2.34%)	0 (0.00%)	3 (0.14%)	0 (0.00%)	58 (2.66%)
	**Hub 1**	1,094	8 (0.73%)	19 (1.74%)	0 (0.00%)	0 (0.00%)	0 (0.00%)	22 (2.01%)
	**Hub 2**	1,089	3 (0.28%)	32 (2.94%)	0 (0.00%)	3 (0.28%)	0 (0.00%)	36 (3.31%)
**CCG3**		2,050	0 (0.00%)	0 (0.00%)	0 (0.00%)	0 (0.00%)	23 (1.12%)	23 (1.12%)
	**Hub 1**	1,806	0 (0.00%)	0 (0.00%)	0 (0.00%)	0 (0.00%)	16 (0.89%)	16 (0.89%)
	**Hub 2**	244	0 (0.00%)	0 (0.00%)	0 (0.00%)	0 (0.00%)	7 (2.87%)	7 (2.87%)
**Total**		32,041	311 (0.97%)	524 (1.64%)	345 (1.08%)	3 (0.01%)	2,382 (7.43%)	2,881 (8.99%)

For peer review only

Supplementary Table S7 Probability model for appointment containing missing data

	Missing appointment data^ (95% CI)
**Day of week (base: Monday)**	
**Tuesday**	0.49 (-1.51, 2.49)
**Wednesday**	1.24 (0.05, 2.42)
**Thursday**	0.22 (-0.40, 0.85)
**Friday**	0.87 (-0.29, 2.02)
**Saturday**	0.74 (-0.08, 1.55)
**Sunday**	1.43 (0.49, 2.37)
**Calendar month (base: January)**	
**February**	-0.54 (-0.93, -0.16)
**March**	-3.11 (-3.99, -2.23)
**April**	-2.53 (-4.06, -0.99)
**May**	-1.41 (-3.13, 0.30)
**June**	-0.87 (-2.64, 0.90)
**July**	-2.73 (-5.85, 0.38)
**August**	-4.11 (-5.17, -3.05)
**September**	-2.30 (-7.49, 2.88)
**October**	-3.10 (-6.15, -0.05)
**November**	-3.18 (-8.20, 1.84)
**December**	-3.40 (-9.87, 3.07)
**CCG scheme (base: CCG5)**	
**CCG2**	-6.76 (-7.12, -6.40)
**CCG3**	-
**CCG4**	-21.90 (-21.98, -21.82)
**Sample size**	29,858

^ CCG3 had 100% data completion (n=2,183)

Probit regression of missing data indicator (gender, age, appointment type, booking type, DNA status, patient's GP practice) against day of week, calendar month, and CCG scheme.

Estimates are presented as average marginal effects which give the percentage point effect (when multiplied by 100) of the variable relative to the base category.

For peer review only

Supplementary Table S8 Booked appointment data: summary statistics

Appointment characteristic	Frequency	Percent	Cumulative percentage
**Day of week**			
**Monday**	3,145	10.79	10.79
**Tuesday**	3,335	11.44	22.22
**Wednesday**	2,970	10.19	32.41
**Thursday**	3,101	10.63	43.04
**Friday**	3,064	10.51	53.55
**Saturday**	10,744	36.84	90.39
**Sunday**	2,801	9.61	100.00
**Calendar month**			
**January**	1,310	4.49	4.49
**February**	2,115	7.25	11.75
**March**	2,522	8.65	20.39
**April**	2,530	8.68	29.07
**May**	2,522	8.65	37.72
**June**	2,240	7.68	45.40
**July**	2,695	9.24	54.64
**August**	2,487	8.53	63.17
**September**	2,568	8.81	71.98
**October**	2,936	10.07	82.05
**November**	2,710	9.29	91.34
**December**	2,525	8.66	100.00
**CCG**			
**CCG2**	21,304	73.06	73.06
**CCG3**	2,027	6.95	80.01
**CCG4**	3,704	12.70	92.71
**CCG5**	2,125	7.29	100.00
**Appointment type**			
**GP**	24,068	82.54	82.54
**Nurse**	5,092	17.46	100.00
**Booking type**			
**Pre-booked**	13,538	46.43	46.43
**Same-day**	15,622	53.57	100.00
**Attended status**			
**DNA**	3,548	12.17	12.17
**Attended**	25,612	87.83	100.00
**Gender**			
**Male**	12,071	41.40	41.40
**Female**	17,089	58.60	100.00
**Age**			
**0-9**	3,233	11.09	11.09
**10-19**	2,939	10.08	21.17
**20-29**	5,299	18.17	39.34
**30-39**	4,832	16.57	55.91
**40-49**	4,542	15.58	71.48
**50-59**	4,133	14.17	85.66
**60-69**	2,530	8.68	94.33
**70-79**	1,169	4.01	98.34
**80-89**	443	1.52	99.86
**90+**	40	0.14	100.00
**Total**	29,160	100.00	

Appointment characteristics for appointments booked and used with complete (non-missing) data

For peer review only

For peer review only

Supplementary Table S9 Booked appointment data: summary statistics by appointment type

Appointment characteristic	Pre-booked	%	Same-day	%	GP	%	Nurse	%	Used	%	DNA	%
**Day of week**												
**Monday**	650	5.70	2,495	15.97	1,893	10.32	476	9.35	2,854	11.16	281	7.92
**Tuesday**	1,380	12.09	1,955	12.51	1,975	10.77	546	10.72	3,001	11.74	332	9.36
**Wednesday**	1,401	12.28	1,569	10.04	1,940	10.58	414	8.13	2,651	10.37	317	8.93
**Thursday**	1,437	12.59	1,664	10.65	1,907	10.40	559	10.98	2,667	10.43	432	12.18
**Friday**	1,449	12.70	1,593	10.20	1,945	10.61	458	8.99	2,606	10.19	455	12.82
**Saturday**	3,862	33.84	4,779	30.59	7,076	38.59	2,189	43.17	9,283	36.30	1,444	40.70
**Sunday**	1,234	10.81	1,567	10.03	1,601	8.73	441	8.66	2,510	9.82	287	8.09
**Calendar month**												
**January**	404	3.54	798	5.11	992	5.41	41	0.81	1,157	4.52	151	4.26
**February**	785	6.88	1,156	7.40	1,643	8.96	66	1.30	1,857	7.26	256	7.22
**March**	1,162	10.18	1,204	7.71	1,905	10.39	57	1.12	2,183	8.54	338	9.53
**April**	1,055	9.24	1,270	8.13	1,569	8.56	478	9.39	2,210	8.64	318	8.96
**May**	986	8.64	1,357	8.69	1,532	8.35	532	10.45	2,192	8.57	323	9.10
**June**	869	7.61	1,202	7.69	1,368	7.46	488	9.58	1,982	7.75	253	7.13
**July**	1,036	9.08	1,427	9.13	1,615	8.81	629	12.35	2,320	9.07	374	10.54
**August**	935	8.19	1,369	8.76	1,454	7.93	610	11.98	2,168	8.48	313	8.82
**September**	1,096	9.60	1,289	8.25	1,482	8.08	628	12.33	2,267	8.87	299	8.43
**October**	1,085	9.51	1,626	10.41	1,623	8.85	685	13.45	2,635	10.30	296	8.34
**November**	999	8.75	1,545	9.89	1,562	8.52	549	10.78	2,409	9.42	298	8.40
**December**	1,001	8.77	1,379	8.83	1,592	8.68	329	6.46	2,192	8.57	329	9.27
**CCG**												
**CCG2**	8,922	78.17	12,382	79.26	17,057	93.02	4,247	83.41	18,640	72.89	2,632	74.18
**CCG3**	519	4.55	1,508	9.65	-	-	-	-	1,828	7.15	197	5.55
**CCG4**	1,972	17.28	1,732	11.09	-	-	-	-	3,297	12.89	404	11.39
**CCG5**	-	-			1,280	6.98	845	16.59	1,807	7.07	315	8.88
**Appointment type**												
**GP**	9,181	80.44	13,607	87.10	-	-	-	-	21,082	82.44	2,960	83.43
**Nurse**	2,232	19.56	2,015	12.90	-	-	-	-	4,490	17.56	588	16.57
**Booking type**												
**Pre-booked**	-	-	-	-	7,970	43.46	3,077	60.43	11,279	44.11	2,244	63.25

For peer review only

**Same-day**	-	-	-	-	10,367	56.54	2,015	39.57	14,293	55.89	1,304	36.75
**Attended status**												
**DNA**	1,929	16.90	1,304	8.35	2,359	12.86	588	11.55	-	-	-	-
**Used**	9,484	83.10	14,318	91.65	15,978	87.14	4,504	88.45	-	-	-	-
**Gender**												
**Male**	4,775	41.84	6,547	41.91	7,782	42.44	1,803	35.41	10,609	41.49	1,450	40.87
**Female**	6,638	58.16	9,075	58.09	10,555	57.56	3,289	64.59	14,963	58.51	2,098	59.13
**Age**												
**0-9**	831	7.28	2,202	14.10	2,293	12.50	73	1.43	2,825	11.05	408	11.50
**10-19**	1,158	10.15	1,594	10.20	1,961	10.69	302	5.93	2,508	9.81	431	12.15
**20-29**	2,178	19.08	2,731	17.48	3,424	18.67	885	17.38	4,343	16.98	956	26.94
**30-39**	1,962	17.19	2,445	15.65	2,978	16.24	934	18.34	4,093	16.01	739	20.83
**40-49**	1,859	16.29	2,329	14.91	2,733	14.90	965	18.95	4,054	15.85	488	13.75
**50-59**	1,783	15.62	2,041	13.06	2,515	13.72	893	17.54	3,813	14.91	320	9.02
**60-69**	991	8.68	1,382	8.85	1,502	8.19	583	11.45	2,418	9.46	112	3.16
**70-79**	462	4.05	645	4.13	671	3.66	302	5.93	1,106	4.33	63	1.78
**80-89**	177	1.55	228	1.46	239	1.30	141	2.77	412	1.61	31	0.87
**90+**	12	0.11	25	0.16	21	0.11	14	0.27	-	-	-	-
**Total**	11,413	100.00	15,662	100.00	18,337	100.00	5,092	100.00	25,572	100.00	3,548	100.00

Appointment characteristics for appointments booked and used with complete (non-missing) data

For peer review only

Supplementary Table S10 Demographic distributions of extended access appointment users

	Males			Females			All
	Frequency	%	Cumulative %	Frequency	%	Cumulative %	Cumulative %
**Extended access**							
**Age**							
**20-29**	1,446	17.71	17.71	2,897	23.93	23.93	21.43
**30-39**	1,575	19.30	37.01	2,513	20.75	44.68	41.60
**40-49**	1,617	19.81	56.82	2,435	20.11	64.79	61.59
**50-59**	1,690	20.71	77.53	2,123	17.53	82.32	80.40
**60-69**	1,144	14.02	91.55	1,273	10.51	92.83	92.32
**70-79**	481	5.89	97.44	624	5.15	97.98	97.77
**80-89**	197	2.41	99.85	215	1.78	99.76	99.80
**90+**	12	0.15	100.00	28	0.23	100.00	100.00
**Total**	8,162	100.00		12,108	100.00		
**GP Patient Survey users***							
**Age**							
**18-24**	298	8.45	8.45	514	10.53	10.53	9.55
**25-34**	472	13.39	21.83	798	16.35	26.88	24.51
**35-44**	618	17.52	39.36	812	16.64	43.52	41.56
**45-54**	720	20.43	59.79	912	18.69	62.21	61.07
**55-64**	608	17.26	77.04	691	14.15	76.36	76.68
**65-74**	493	14.00	91.04	578	11.85	88.21	89.54
**75-84**	251	7.11	98.15	383	7.85	96.06	97.04
**85+**	65	1.85	100.00	192	3.94	100.00	100.00
**Total**	3,524	100.00		4,881			

*GPPS weighted responses for patients from practices in CCGs 2 to 5, rounded to nearest whole number

For peer review only

MANCHESTER 1824
The University of Manchester

NHS
National Institute for Health Research
Collaboration for Leadership in Applied Health Research and Care (CLAHRC) for Greater Manchester

GM PRIMARY CARE 7 DAY ACCESS EVALUATION: RESEARCH PROTOCOL

Background

In June 2014, the 12 Greater Manchester Clinical Commissioning Groups (CCGs) agreed to the Healthier Together Primary Care Standard that by the end of 2015, "everyone in Greater Manchester who needs medical help will have same-day access to primary care services, supported by diagnostic tests, seven days a week"[1]. At the same time seven day access has been made one of eight early implementation priorities under the Greater Manchester devolution agreement[2].

This protocol describes the proposed evaluation of seven day access to primary care to be conducted on behalf of NHS England (Greater Manchester) by CLAHRC Greater Manchester. CLAHRC GM previously conducted the evaluation of the NHS England Primary Care Demonstrator services, and the proposed evaluation seeks to build on the strengths of the previous evaluation. The proposed mixed methods evaluation will combine a focus on **activity**, **process** and **outcomes** in order to provide a comprehensive assessment of the implementation and impact of the new services. The evaluation will focus primarily on the 7 CCG areas receiving support from NHS England as part of the devolution agreement. The remaining 5 CCG areas have pre-existing funding and support to provide 7 day access via either the Wave 1 Prime Minister's Challenge Fund (PMCF) or Wave 2 General Practice Access Fund (GPAF) (see **Table 1**). The majority of these are already 'live'; therefore while they will not make up part of the formal evaluation, intelligence gathered in the PMCF/GPAF areas can be used to support learning and development in the seven areas where 7-day primary care access is now being introduced.

Table 1: 7-Day Access Areas in Greater Manchester

PMCF/GPAF Areas (not evaluated)	New 7-Day Access Areas (to be evaluated)
Bury	Bolton
Wigan	Heywood, Middleton and Rochdale
North Manchester	Oldham
Central Manchester	Salford
South Manchester	Stockport
	Trafford
	Tameside & Glossop

[1] https://healthiertogethergm.nhs.uk/what-healthier-together/primary-care/
[2] http://www.gmhealthandsocialcaredevo.org.uk/assets/GM-Strategic-Plan-Final.pdf p14

For peer review only

The 7 CCGs submitted their initial plans for the delivery of seven day primary care access to NHS England in mid-2015. The schemes were expected to start in December 2015, although some have indicated a later start date. All areas are adopting a ‘hub’ approach to providing additional hours, in which the CCG area is split into geographic areas, and the extended access services are provided from a ‘hub’ location in each area. CCG plans submitted to NHS England promise 43 hubs (including PMCF/GPAF hubs) operational by December 2015 with a further 9 hubs live in 2016 (see figure 1).

Figure 1

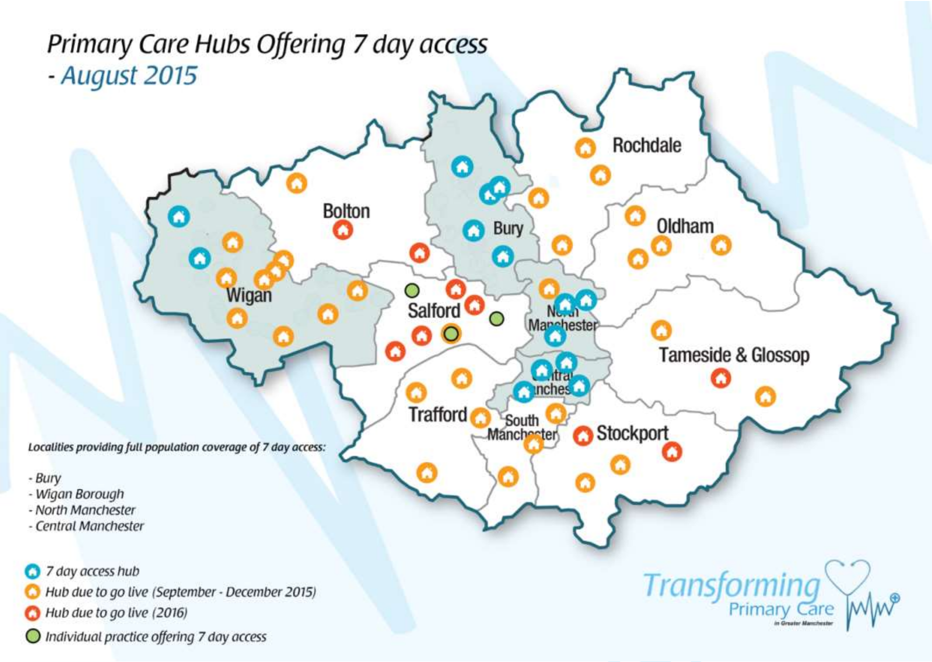

Description of Evaluation

The evaluation seeks to understand the implementation and impact of seven day primary care services across Greater Manchester. The evaluation will combine qualitative and quantitative analysis, supported by a measure of activity across the 7 areas to be evaluated.

The evaluation will seek to answer the following questions (see **Table 2**);

Table 2: Evaluation Questions and Modes of Evaluation

1. What new activity is associated with seven day primary care services in each area, and at what cost?	**ACTIVITY**
2. How are these new services utilised by patients? What is the nature of demand for these new services?	
3. How is the uptake of 7 day primary care affected by the socio-economic characteristics of the patient population?	
4. What organisational and operational issues arise in the implementation of 7 day access to primary care, and how have areas addressed these issues?	**PROCESS**

5. What is the impact of 7 day access to primary care on;<br>a. Identified prevalence of disease,<br>b. Attendances/admissions at Accident & Emergency,<br>c. Use of Out-of-Hours services?	**OUTCOME**
6. How has the change in access been received by<br>a. Patients,<br>b. Staff in primary care?	

As is indicated above, questions 1-3 relate to the **activity** analysis (i.e. what was done). Question 4 relates to the **process** analysis (i.e. how was it done). Questions 5 and 6 relate to the **outcome** analysis (i.e. what was the impact of the new activity).

It should be emphasised that the ability to answer these questions depends directly on the availability of relevant data. It is important that data can be captured by the evaluation team in a timely manner, can be presented in a format which allows for analysis, and that this can be done subject to current information governance regulation. Some data is routinely collected, other data can be collected by the evaluation team with the facilitation of the commissioners and providers in each area, and some can only be produced by the providers themselves (see **Table 3**);

Table 3: Evaluation Questions and Data Sources

1. What new activity is associated with seven day primary care services in each area, and at what cost?	**Activity data supplied by each area**
2. How are these new services utilised by patients? What is the nature of demand for these new services?	**Activity data supplied by each area**
3. How is the uptake of 7 day primary care affected by the socio-economic characteristics of the patient population?	**Public data combined with activity data.**
4. What organisational and operational issues arise in the implementation of 7 day access to primary care, and how have areas addressed these issues?	**Evaluation generated data. Source: qualitative interviews.**
5. What is the impact of 7 day access to primary care on;<br>a. Identified prevalence of disease,<br>b. Attendances/admissions at Accident & Emergency,<br>c. Use of Out-of-Hours services?	**Routinely collected data. Source:<br>(a) QOF, via CCGs,<br>(b) SUS, via AHSN,<br>(c) OOH activity, supplied by NHS England/CCGs.**
6. How has the change in access been received by<br>a. Patients,<br>b. Staff in primary care?	**Evaluation generated data. Source:<br>(a) GPPS, via HSCIC.<br>(b) staff survey administered by evaluation team via CCGs.**

The evaluation period will begin at the point at which the NHS England hubs go live on 1st January 2016 and continue for 12 months.

For peer review only

The evaluation has conducted preliminary work to establish reliable sources of data, in discussion with NHS England (Greater Manchester) and the Greater Manchester Academic Health Science Network (AHSN). The provision of activity data from each area has not yet been agreed: - this research protocol document is intended to provide information and thereby initiate the process of agreeing the content and process of activity capture in dialogue with each area.

Further details on the data capture and analysis is provided below.

1. Activity Capture

The activity capture in the present evaluation has been designed in order to minimise the additional workload that data extraction places on individual sites, while also seeking to ensure that the data collected can be collected reliably and consistently across all 7 areas.

To this end, a minimum data set (**Table 4** below) has been defined, to be agreed with all participating CCGs. Data should be reported according to a monthly schedule, which will support formative evaluation and shared learning across sites. Also data will be needed from NHS England/CCGs on relevant DES arrangements in each area, to establish activity baselines.

Table 4: Activity Capture Minimum Dataset

**1. Utilisation:**
a. No. of appointments available
i. By day
1. Time of appointment
ii. By discipline
iii. Planned/pre-booked vs same day/urgent
iv. Method of appointment (face-to-face/phone)
b. No. of appointments booked
i. By day
1. Time of appointment
ii. By discipline
iii. Age
iv. Gender
v. Registered GP practice
vi. Postcode
vii. Planned/pre-booked vs same day/urgent
viii. Method of appointment (face-to-face/phone)
c. No. of appointments used (Utilisation)
i. By day
1. Time of appointment
ii. By discipline
iii. Age
iv. Gender
v. Registered GP practice
vi. Postcode (first part)
vii. Planned/pre-booked vs same day/urgent
viii. Method of appointment (face-to-face/phone)

For peer review only

**2. Practice-level population data:**	
	a. Practice level population
	b. Practice codes
	c. Whether a GP practice or not
	d. Registered patient age band
	e. Deprivation of practice population
	f. Gender breakdown of practice population

The aim of the activity capture evaluation is to:

1. **Assess the ability of each area to provide additional appointments i.e.**
   a. **How many additional appointments were available, by whom and by what means?**
   b. **How many additional appointments were booked, urgent, and how many resulted in DNA?**
2. **Assess the types of patients utilizing additional appointments i.e.**
   a. **Were patients attending/DNA representative of the practice's total list (in terms of age, gender, and IMD)?**

For utilisation data (Items 1a-1c, Table 3), the evaluation relies on the ability of each area to collect, collate and provide data routinely. Preliminary conversations with NHS England and some local CCGs suggest that this data should be available – however, this requires confirmation with all areas.[3]

CCG level population data (Item 2, Table 3) can be extracted by the CLAHRC evaluation team from individual practice registration data.

2. Process Evaluation

Semi-structured interviews will be conducted with senior leadership (CCG lead + GP lead in each area). 2 leads x 7 areas= 14 interviews, conducted at start and end of intervention period = 28 interviews. These will be supplemented with key informant interviews (estimated 6-7), including leads in the PMCF/GPAF areas, and, where appropriate, additional interviews with key individuals in each area.

The process evaluation will seek to understand the organisational and operational issues in the implementation of 7 day access to primary care faced in each area, and also how each area addressed these issues. A brief interview schedule can be found in **Appendix 1**.

[3] In addition, the following data would enhance the analysis by providing insight into the reasons for and nature of the additional consultations.

1. Reason for attendance
2. Outcome of consultation
   a. Discharged with advice
   b. Referral to ….. (list providers)
   c. Treatment/prescription issued
   d. Follow-up appointment
   e. Diagnostics
   f. Other

However, we recognise this may be difficult or impossible to provide without incurring substantial and unrealistic data collection costs on the areas.

For peer review only

3. Outcome Evaluation

The outcome evaluation will concentrate on four areas; (A) Health Outcomes, (B) Impact on Service Utilisation, (C) Staff Experience and (D) Patient Experience.

A. **Impact on Health Outcomes** will be primarily measured using selected QOF data on disease prevalence. QOF data on disease prevalence will be analysed to examine the effect of the intervention on the identification and management of long-term conditions. This data will be supplied by local CCGs. Data will be captured for three years prior to the intervention (2013-16) and at two points during the intervention itself. A full list of QOF data categories can be found in **Appendix 2**.

B. **Impact on Service Utilisation** will be measured using routinely-collected SUS data on hospital activity, plus activity data from relevant out-of-hours providers supplied by NHS England/CCGs.

SUS data will be extracted for all areas of Greater Manchester (seven 7 day access sites and 5 PMCF/GPAF areas) on a quarterly basis. The specific items to be analysed can be found in **Appendix 3.**

The aim of the service utilisation evaluation is to:

(1). Assess the effects of additional appointments on hospital activity:

a) A&E attendances:

i) Minor patient-initiated A&E attendances

ii) Total A&E attendances

iii) Waiting time to assessment, treatment, and total A&E duration

b) Admissions:

i) Total admissions

ii) Admissions for ambulatory care-sensitive conditions

(2). Assess whether (1) varies by dosage/volume of additional appointments per 1000

(3). Assess whether (1) varies by additional appointments' users characteristics (age, gender, deprivation)

(1) will be measured using SUS data for the intervention period (quarters 1 to 4 of 2016) compared to 2015 i.e. a before and after analysis. All activity will be measured at the practice level, by quarter, and per 1000 of a practices registered population. A pooled cross-sectional dataset will be used. For each type of hospital activity the dependent variable will be regressed on binary year dummies. The estimated coefficient on the intervention (2016) dummy provide a statistical test for significant differences in the intervention period. Count models will be estimated that recognise and incorporate the over-dispersed distribution of hospital activity. Sensitivity analysis using alternative model specifications will be carried out.

The diagnosis code (DIAG_NN) in the admissions data will be used to identify ambulatory care sensitive conditions as follows:

- Ambulatory care-sensitive conditions admissions (ICD)
- Vaccine-preventable:
  Influenza and pneumonia; Other vaccine-preventable conditions
- Chronic:
  Asthma; Congestive heart failure; Diabetes complications; Chronic obstructive pulmonary disease (COPD); Angina; Iron-deficiency anaemia; Hypertension; Nutritional deficiencies

For peer review only

- Acute:

  Dehydration and gastroenteritis; Pyelonephritis; Perforated/bleeding ulcer; Cellulitis; Pelvic inflammatory disease; Ear, nose and throat infections; Dental conditions; Convulsions and epilepsy; Gangrene

Where available, the difference in hospital activity will be compared against comparator practices and tests for significant differences measured using a *controlled* before and after analysis framework (difference-in-differences).

Items (2) and (3) depend on the quality and coverage of the activity capture. Using data from the activity capture we will generate a measure of intensity of treatment (dosage - additional appointments per 1000 of a practice's registered population) and replicate (1) by dosage. Information on the types of patients using additional appointments from the activity capture will be used to identify the groups where hospital activity may be most concentrated. For example, if additional appointments are concentrated amongst females then it may be that female hospital activity is where the impacts of additional appointments may be seen.

C. **Staff Experience** The evaluation team will develop an online survey to capture information on the experience of primary care staff. This administration of this survey will require the cooperation of local provider organisations.
D. **Patient Experience** We will analyse General Practice Patient Survey (GPPS) data, adopting a difference-in-difference (DiD) methodology, focusing on survey items addressing overall quality of care and convenience, subject to data availability (see note below). Individual CCGs may choose to conduct their own patient surveys, and guidance can be provided by the evaluation team on the administration of these.[4]

[4] Note: While the relevant period of GPPS data will be July-September 2016, which should be released in early 2017. If the release of this data is delayed by HSCIC, to avoid delaying report submission at end of March 2017 then patient experience analysis may have to be supplied latter as a separate addendum to the report.

For peer review only

Evaluation Timeline

	JAN 16	FEB 16	MAR 16	APR 16	MAY 16	JUN 16	JUL 16	AUG 16	SEP 16	OCT 16	NOV 16	DEC 16	JAN 17	FEB 17	MAR 17
Research Approvals	■	■	■	■	■	■									
Activity Capture						■	■	■	■	■	■	■			
Process Evaluation						■	■	■			■	■			
Outcome Evaluation						■			■			■	■	■	
Patient Experience													■	■	
Staff Experience									■	■	■				
Findings (Interim and Final)										■					■

This timeline assumes that;

- The minimum activity dataset can be provided on a monthly basis by CCGs.
- Information on relevant DES activity can be supplied by NHS England/CCGs.
- The evaluation team has access to key individuals in each area for interviews.
- CCGs are able to provide selected QOF data on disease prevalence.
- The AHSN is able to supply regular extracts of SUS data in the appropriate format.
- NHSE is able to supply data on OOH activity in the appropriate format.
- CCGs are able to assist with the dissemination of an online staff survey.
- GP Patient Survey results are released by HSCIC in a timely manner.

For peer review only

Appendix 1

Interview Schedule

1. Describe the primary care access arrangements in your area outside standard hours prior to this initiative, and describe the 7-day access arrangements in place now.
2. What is your role in delivering this change?
3. What is/was required to establish 7-day access to primary care in your locality?
4. What steps have been taken so far?
5. How have you communicated the changes to patients?
6. What challenges have been encountered (IT, IG, communications and engagement, workforce, finance, infrastructure)?
7. How have you tackled these challenges?
8. What do you expect will be the impact of this change in access (on patients, staff and other parts of the health and social care system)?
9. How would you measure ‘success’ in this change?
10. How sustainable are the changes made in your area?

For peer review only

Appendix 2

AST001. The contractor establishes and maintains a register of patients with asthma, excluding patients with asthma who have been prescribed no asthma-related drugs in the preceding 12 months.

AF001. The contractor establishes and maintains a register of patients with atrial fibrillation.

CAN001. The contractor establishes and maintains a register of all cancer patients defined as a "register of patients with a diagnosis of cancer excluding non-melanotic skin cancers diagnosed on or after 1 April 2003".

CKD001. The contractor establishes and maintains a register of patients aged 18 or over with CKD (US National Kidney Foundation: Stage 3 to 5 CKD).

COPD001. The contractor establishes and maintains a register of patients with COPD.

DEM001. The contractor establishes and maintains a register of patients diagnosed with dementia.

DEP003. The percentage of patients aged 18 or over with a new diagnosis of depression in the preceding 1 April to 31 March, who have been reviewed not earlier than 10 days after and not later than 56 days after the date of diagnosis.

DM017. The contractor establishes and maintains a register of all patients aged 17 or over with diabetes mellitus, which specifies the type of diabetes where a diagnosis has been confirmed.

EP001. The contractor establishes and maintains a register of patients aged 18 or over receiving drug treatment for epilepsy.

HF001. The contractor establishes and maintains a register of patients with heart failure.

HYP001. The contractor establishes and maintains a register of patients with established hypertension.

LD003. The contractor establishes and maintains a register of patients with learning disabilities.

MH001. The contractor establishes and maintains a register of patients with schizophrenia, bipolar affective disorder and other psychoses and other patients on lithium therapy.

OST004. The contractor establishes and maintains a register of patients: 1. Aged 50 or over and who have not attained the age of 75 with a record of a fragility fracture on or after 1 April 2012 and a diagnosis of osteoporosis confirmed on DXA scan, and 2. Aged 75 or over with a record of a fragility fracture on or after 1 April 2014 and a diagnosis of osteoporosis.

PC001. The contractor establishes and maintains a register of all patients in need of palliative care/support irrespective of age.

PAD001. The contractor establishes and maintains a register of patients with peripheral arterial disease.

RA001. The contractor establishes and maintains a register of patients aged 16 or over with rheumatoid arthritis.

CHD001. The contractor establishes and maintains a register of patients with coronary heart disease.

STIA001. The contractor establishes and maintains a register of patients with stroke or TIA.

For peer review only

Appendix 3

Relevant SUS data to be analysed

Derived variable	SUS variable name	SUS variable code
**A&E DATA**		
**KEY BASE DATA**		
**A&E department type**	A&E department type	AEDEPTTYPE
**Provider code**	Provider code	PROCODET
**Arrival date**	A&E arrival date	ARRIVALDATE
**Arrival time**	A&E arrival time	ARRIVALTIME
**Arrival mode**	Arrival mode	AEARRIVALMODE
**Where incident took place**	Incident location type	AEINCLOCTYPE
**GP practice**	Code of GP practice	GPPRAC
**CCG of GP practice**	CCG of patient's GP practice	CCG_GP_PRACTICE
**CCG of Treatment**	CCG of Treatment	CCG_TREATMENT
**Self-referrals for minor conditions**	Referral mode,<br>SUS PbR derived healthcare resource group code	AEREFSOURCE<br>SUSHRG
**Cost**	Trust derived HRG value	HRGNHS
**GP referrals**	Referral mode	AEREFSOURCE
**Arrival age**	Age on arrival	ARRIVALAGE
**Sex**	Sex of patient	SEX
**Ethnicity**	Ethnic category	ETHNOS
**Deprivation**	Lower Super Output Area (to obtain IMD)	LSOA11
**Diagnosis**	A&E diagnosis	DIAG_NN
**EFFECT ON ADMISSIONS/WORKLOAD**		
**Admissions from A&E**	Attendance disposal	AEATTENDISP
**A&E Waiting times**		
**A&E time to assessment (waiting time)**	A&E Duration to assessment	INITDUR
**A&E time to treatment (waiting time)**	A&E Duration to treatment	TRETDUR
**A&E total duration**	A&E duration to departure	DEPDUR
**ADMISSIONS DATA**		
**KEY BASE DATA**		
**Provider code**	Provider code	PROCODET
**Arrival date**	Date of admission	ADMIDATE
**Discharge date**	Date of discharge	DISDATE
**GP practice**	Code of GP practice	GPPRAC
**CCG of GP practice**	CCG of patient's GP practice	CCG_GP_PRACTICE
**CCG of Treatment**	CCG of Treatment	CCG_TREATMENT
**Cost**	Trust derived HRG value	HRGNHS
**Arrival age**	Age on arrival	ADMIAGE
**Sex**	Sex of patient	SEX
**Ethnicity**	Ethnic category	ETHNOS
**Deprivation**	Lower Super Output Area (to obtain IMD)	LSOA11
**Ambulatory care-sensitive conditions admissions (ICD)**	All Diagnosis codes	DIAG_NN

For peer review only

Supplementary Text S2

(i) Uptake

Patients could book an appointment which could then be attended or not. Focussing on booked appointments alone would give an indication of the demand for the service, whilst assessments of appointments actually used shed light on realised demand and is more insightful for evaluating actual impacts on service use. We estimated two separate sets of probability models:

1) the probability an appointment was booked, and
2) the probability an appointment was booked and attended ('used')

We assessed differences in the rate of uptake by estimating probability models (probit regressions) of appointment use (y_{ij}):

$$y_{ij} = \beta_1 D_{ij} + \beta_2 M_{ij} + \beta_3 CCG_j + e_{ij} \qquad [1]$$

where the unit of analysis is available appointment i in CCG j.

Regression models were estimated because there are instances of missing data meaning the dataset is not a complete picture of activity in the population. Further, regression models enable adjustment for CCG effects which may be important given the variation in schemes across CCGs (Supplementary Table 1), Probit models were chosen as these estimate differences in the probability (rate) of uptake across categories of the predictor variables. Probit regressions were preferred over logistic regressions as probit estimates give absolute percentage differences across predictor variables (rather than relative odds-ratios via logistic regressions) which is more informative for understanding the scale of uptake.

To assess whether uptake of the service varied across day of the week we included day of week dummies in the regression (D_i), with the estimates ($\hat{\beta}_1$) providing the differences in the rate of uptake relative to the base week day (Monday); this enables a test for whether uptake is lower on particular days. To assess whether there is evidence of assimilation to the service we include calendar month dummies (M_i). If assimilation is evident the estimates on the calendar month dummies should be monotonically increasing over the period. To account for potential confounding caused by variations in the provision and delivery of services across schemes we include CCG dummies (fixed-effects) in the regression (CCG_j). Standard errors are clustered at CCG level.

Estimates are presented as average marginal effects (using Stata's margins command), which give the average percentage point effect of the variable relative to the base category across the sample.

(ii) Analysis of demographics of patients booking an extended access appointment

Our second set of analyses concentrated on booked appointments and exploited variations in service delivery to test whether there were associations in the demand for booking particular types of services (booking type (pre-booked or same day) and appointment type (GP or nurse)) and for whether an appointment was used or DNA; with day of week, calendar month, the patient booking the appointments age and gender, and CCG. We estimated three separate sets of probability models:

1) The probability an appointment was pre-booked versus same day;
2) The probability an appointment was for a GP versus for a nurse practitioner;
3) The probability an appointment was used or not attended (DNA)

We assessed differences in the rate of appointment type by estimating probability models (probit regressions) of appointment type (y_{ij}):

$$y_{ij} = \beta_1 D_{ij} + \beta_2 M_{ij} + \beta_3 DNA_{ij} + \beta_4 Female_{ij} + \beta_5 Age_{ij} + \beta_6 CCG_j + e_{ij} \qquad [2]$$

where the unit of analysis is the type of booked appointment i in CCG j. $Female_{ij}$ is a dummy variable indicating the gender of the patient booked for appointment i in CCG j, and Age_{ij} is a set of 10 age-band dummies indicating the age of the patient. For all models estimated, estimates are presented as average marginal effects and standard errors are clustered at GP practice level where the practice is the patient who has booked the appointments registered practice. Schemes that exclusively provided a certain service (e.g. only GP appointments) are excluded from the relevant analysis since uptake of the alternative is necessarily zero.

For peer review only

Supplementary Text S3

CLAHRC Patient Panel Protocol Review
Collated Feedback and Responses

**Feedback from panel**	**Project team response**
The interview document [referring to the interview schedule] seems ok.	No response required.
The survey document I would change slightly as per the attachment:<br><br>5. Which of the following do you feel (may have-deleted) has benefited from the implementation of seven day access to primary care?<br><br>6. Remove mentions of 'efforts to' from the response options e.g. efforts to reduce demand on A&E services.	
This is a very comprehensive, well thought out document. It is a very comprehensive protocol indeed.<br><br>Appointments must be booked in advance, but what if changes take place on the part of either the patient or the GP hub nearer the time of the appointment?<br><br>Evidence of use and pattern of use at weekends is crucial! Evenings will prove popular, Saturday mornings will indicate some demand but I am unsure about the demand on Saturday afternoons and especially Sundays. I am referring to planned appointments and not Emergency appointments!	No response required.<br><br>No change to protocol as this situation is not specific to the additional availability, as appointments are also booked in advance within core services.<br><br>Agree that utilisation by day of week and time of day is important. This will be monitored via the activity data.
The 'don't know' response option should be removed, in all cases, from the staff survey.	No change to staff survey, as 'don't know' response is required to ensure that those who select an alternative option do so as there is an element of certainty about their choice. Important for data quality.
The following questions are suggested to be added to the interview schedule:<br><br>a. How effective has the 7 day access scheme been in terms of number of patients visiting during extended hours?<br>b. Describe the patterns of demand during the extended hours and the average cost per 10 minute slot?	No change to interview schedule, as captured via the activity data.<br><br>No change to interview schedule, as captured via the activity data.

c. Indicate staff morale during the extended hours?	No change to interview schedule, as captured via the staff survey.

For peer review only

STROBE Statement—Checklist of items that should be included in reports of ***cohort studies***

	Item No	Recommendation	Location in manuscript
**Title and abstract**	1	(*a*) Indicate the study's design with a commonly used term in the title or the abstract	Title
		(*b*) Provide in the abstract an informative and balanced summary of what was done and what was found	Abstract
**Introduction**			
Background/rationale	2	Explain the scientific background and rationale for the investigation being reported	Introduction: Paragraphs 1, 2 & 3
Objectives	3	State specific objectives, including any prespecified hypotheses	Introduction: Paragraph 4
**Methods**			
Study design	4	Present key elements of study design early in the paper	Introduction: Paragraph 2<br>Methods: Paragraphs 1, 2, 3, & 4
Setting	5	Describe the setting, locations, and relevant dates, including periods of recruitment, exposure, follow-up, and data collection	Methods: Paragraphs 1, 2, 3, & 12<br>Figure 1<br>Supplementary Table S1
Participants	6	(*a*) Give the eligibility criteria, and the sources and methods of selection of participants. Describe methods of follow-up	Methods: Paragraphs 1, 2, 3, & 12<br>Figure 1
		(*b*) For matched studies, give matching criteria and number of exposed and unexposed	N/A
Variables	7	Clearly define all outcomes, exposures, predictors, potential confounders, and effect modifiers. Give diagnostic criteria, if applicable	Methods: Paragraphs 5, 6, 7, 8, 9, 10, 11 & 12
Data sources/ measurement	8*	For each variable of interest, give sources of data and details of methods of assessment (measurement). Describe comparability of assessment methods if there is more than one group	Methods: Paragraphs 1, 2, 3, & 12<br>Supplementary Table S2
Bias	9	Describe any efforts to address potential sources of bias	Methods: Paragraphs 7, 10, 11, & 12
Study size	10	Explain how the study size was arrived at	Methods: Paragraphs 1, 3 & 12<br>Results: Paragraph 1 & 5<br>Figure 1
Quantitative variables	11	Explain how quantitative variables were handled in the analyses. If applicable, describe which groupings were chosen and why	Methods: Paragraphs 6, 7, 8, 9, 10, 11, & 12<br>Supplementary Table S2
Statistical methods	12	(*a*) Describe all statistical methods, including those used to control for confounding	Methods: Paragraphs 6, 7, 8, 9, 10, 11, & 12

For peer review only

			Supplementary Text S2
		(*b*) Describe any methods used to examine subgroups and interactions	Results:<br>Paragraph 4
		(*c*) Explain how missing data were addressed	Methods:<br>Paragraph 11<br>Results:<br>Paragraph 5<br>Supplementary Tables S6 & S7
		(*d*) If applicable, explain how loss to follow-up was addressed	N/A
		(*e*) Describe any sensitivity analyses	Results:<br>Paragraph 3
**Results**			
Participants	13*	(a) Report numbers of individuals at each stage of study—eg numbers potentially eligible, examined for eligibility, confirmed eligible, included in the study, completing follow-up, and analysed	Results:<br>Paragraph 1, 5, & 9<br>Tables 1, 2 & 5<br>Figure 1
		(b) Give reasons for non-participation at each stage	Results:<br>Paragraphs 1 & 5<br>Tables 1, 2, 3 & 4<br>Footnotes
		(c) Consider use of a flow diagram	Figure 1
Descriptive data	14*	(a) Give characteristics of study participants (eg demographic, clinical, social) and information on exposures and potential confounders	Results:<br>Paragraphs 6, 7, 8, 9, & 10<br>Supplementary Tables S8, S9 & S10
		(b) Indicate number of participants with missing data for each variable of interest	Results:<br>Paragraph 5<br>Supplementary Table S6
		(c) Summarise follow-up time (eg, average and total amount)	N/A
Outcome data	15*	Report numbers of outcome events or summary measures over time	Results:<br>Paragraph 3<br>Figure 2 & 3<br>Supplementary Table S3
Main results	16	(*a*) Give unadjusted estimates and, if applicable, confounder-adjusted estimates and their precision (eg, 95% confidence interval). Make clear which confounders were adjusted for and why they were included	Results:<br>Paragraphs 1, 2, 3, 4, 5, 6, 7, 8, & 9<br>Tables 1, 2, 3, 4 & 5<br>Supplementary Tables S3 to S10
		(*b*) Report category boundaries when continuous variables were categorized	N/A
		(*c*) If relevant, consider translating estimates of relative risk into absolute risk for a meaningful time period	Methods:<br>Paragraph 7<br>Supplementary Text S2

For peer review only - http://bmjopen.bmj.com/site/about/guidelines.xhtml

Other analyses	17	Report other analyses done—eg analyses of subgroups and interactions, and sensitivity analyses	Results: Paragraph 4
**Discussion**			
Key results	18	Summarise key results with reference to study objectives	Discussion: Paragraph 1
Limitations	19	Discuss limitations of the study, taking into account sources of potential bias or imprecision. Discuss both direction and magnitude of any potential bias	Discussion: Paragraph 2
Interpretation	20	Give a cautious overall interpretation of results considering objectives, limitations, multiplicity of analyses, results from similar studies, and other relevant evidence	Discussion: Paragraphs 3, 4, 5, & 6
Generalisability	21	Discuss the generalisability (external validity) of the study results	Discussion: Paragraph 2
**Other information**			
Funding	22	Give the source of funding and the role of the funders for the present study and, if applicable, for the original study on which the present article is based	Funding statement

*Give information separately for exposed and unexposed groups.

Note: An Explanation and Elaboration article discusses each checklist item and gives methodological background and published examples of transparent reporting. The STROBE checklist is best used in conjunction with this article (freely available on the Web sites of PLoS Medicine at http://www.plosmedicine.org/, Annals of Internal Medicine at http://www.annals.org/, and Epidemiology at http://www.epidem.com/). Information on the STROBE Initiative is available at http://www.strobe-statement.org.

For peer review only

For peer review only - http://bmjopen.bmj.com/site/about/guidelines.xhtml

BMJ Open

An investigation of the demand for a 7-day (extended access) primary care service: an observational study from pilot schemes in England

Journal:	*BMJ Open*
Manuscript ID	bmjopen-2018-028138.R2
Article Type:	Research
Date Submitted by the Author:	29-Jul-2019
Complete List of Authors:	Whittaker, William; University of Manchester, Manchester Centre for Health Economics<br>Anselmi, Laura; University of Manchester, Manchester Centre for Health Economics<br>Nelson, Pauline; University of Manchester Alliance Manchester Business School; NIHR CLAHRC Greater Manchester, UK<br>O'Donnell, Caroline; NIHR CLAHRC Greater Manchester, UK<br>Ross, Natalie; University of Manchester Alliance Manchester Business School<br>Rothwell, Katy; NIHR CLAHRC Greater Manchester, UK<br>Hodgson, Damian; University of Manchester Alliance Manchester Business School; NIHR CLAHRC Greater Manchester, UK
**Primary Subject Heading**:	Health services research
Secondary Subject Heading:	Health policy, Health economics
Keywords:	PRIMARY CARE, ACCESS, HEALTH ECONOMICS, Health policy < HEALTH SERVICES ADMINISTRATION & MANAGEMENT

SCHOLARONE™
Manuscripts

An investigation of the demand for a 7-day (extended access) primary care service: an observational study from pilot schemes in England

Whittaker W[1], Anselmi L[1], Nelson P[2,3], O'Donnell C[3], Ross N[2], Rothwell K[3], Hodgson D[2,3].

[1]Manchester Centre for Health Economics, University of Manchester, UK
[2]Alliance Manchester Business School, University of Manchester, UK
[3]NIHR CLAHRC Greater Manchester, UK

Correspondence to:
William.whittaker@manchester.ac.uk
Manchester Centre for Health Economics, 4.320 Jean McFarlane Building, University of Manchester, Oxford Road, Manchester, M13 9PL

Word count: 5,637

For peer review only

ABSTRACT

Objectives - To understand how the uptake of an extended primary care service in the evenings and weekend varied by day of week and over time. Secondary objectives were to understand patient demographics of users of the service and how these varied by type of appointment and to core hour users.
Design –Observational study.
Setting - Primary care extended access appointments data in 13 centres in Greater Manchester, England during 2016.
Participants – Appointments could be booked by 1,261,326 patients registered with a family practitioner in five Clinical Commissioning Group geographic areas.
Main outcome measures – Primary outcome measure was whether an appointment was used (booked and attended), secondary outcome measures included whether used appointments were pre-booked or booked the same-day, and delivered by a family or nurse practitioner. Additional analyses compared patient demographics to patients reporting the use of core-hour primary care services.
Results - 65.33% of 42,472 appointments were booked and attended (used). Usage of appointments was lowest on a Sunday at 46.73% (18.07 percentage points lower usage than on Mondays (95%CI -32.46 to -3.68)). Pre-booked appointments were more likely to be booked amongst age groups 0 to 9 and to result in patients not attending an appointment. Family practitioner appointments were increasingly less likely to be booked with age in comparison to nurse appointments. Patients attending extended access appointments tended to be younger in comparison to core hour patients.
Conclusions -There is spare capacity in the extended access service, particularly on Sundays, suggesting reconfigurations of the service may be needed to improve efficiency of delivering the service. Patients demographics suggest the service is used by a relatively younger population than core hour services. Patient demographics varied with the types of appointment provided, these findings may help healthcare providers improve usage by tailoring appointment provision to local populations.

Strengths and limitations of this study

- The first study assessing same-day and pre-bookable appointments at evenings and weekends in general practice
- The first study comparing demographics of patients using evening and weekend appointments to patients using core hour appointments
- The first study assessing uptake of evening and weekend appointments over time
- The study covers five areas with differing provision, but may not be generalisable to other settings
- No information on child use of core hours was available, limiting an assessment of this group of the population

For peer review only - http://bmjopen.bmj.com/site/about/guidelines.xhtml

INTRODUCTION

Providing access to health care services when needed is a key target for health systems worldwide.[1] However, patients commonly report difficulties in accessing primary care, particularly outside of core working hours.[2,3] Constrained access to primary care has been a reason for increased patient dissatisfaction and use of emergency care in a variety of countries and settings.[4] Rising use of emergency department services and patient dissatisfaction with access have highlighted a need for better access to primary care.

In England primary care core hour services are provided by General Practitioners (GP) 8am to 6:30pm Monday to Friday either by pre-booking or calling on the same day. The National Health Service (NHS) in England aims to extend access to primary care services in the evening and at weekends as part of their strategy for delivering primary care by 2020/2021.[5] The strategy seeks to enable local commissioners of health care to redesign primary care services and commission extra capacity so that by 2020 *'everyone has access to GP services, including sufficient routine appointments at evenings and weekends to meet locally determined demand, alongside effective access to out-of-hours (OoH) and urgent care services*'. Extended access has been piloted nationally by NHS England since 2013 through the Prime Minister's Challenge Fund, and its successor the GP Access Fund. Wave 1 covered 7.5m people in 2014, rising to 18m (a third of the population of England) by 2015 in Wave 2. In parallel, NHS England Greater Manchester (NHSEGM), now the Greater Manchester Health and Social Care Partnership (GMHSCP), have also piloted extended access appointments with an initial wave in 2014[6], followed by a roll-out throughout Greater Manchester (GM) from 2016 in line with the region's devolution and health and social care strategy[7]. A description of extended access appointments in Greater Manchester is provided in Box 1.

Box 1: Description of Greater Manchester extended access appointments in primary care

<u>What is the service?</u><br>Extended access appointments are appointments in primary care outside of core hours (typically 8am to 6:30pm). Appointments are provided in addition to usual care outside of core family practitioner hours which includes: Walk in Centres, Out-of-Hour services, Accident and Emergency/secondary care, NHS Direct ('111'). The services may have been designed to replace existing Direct Enhanced Services arrangements (additional hours of opening contractually agreed with NHS England).<br><br><u>Who provided the service?</u><br>Extended access appointments are appointments with either a GP or nurse practitioner.<br><br><u>How is the service delivered?</u><br>Extended access appointments are typically face to face appointments delivered individually.

For peer review only

<u>Where is the service delivered?</u>

Extended access appointments are usually provided through one practice or similar premises which acts as a local ‘hub’ covering several practices in a given geographic area, with between 1-4 hubs serving the patient population. Hub locations were decided by local health care commissioners (Clinical Commissioning Groups, CCGs). CCGs are responsible for the commissioning of health care to a local, geographically defined population comprising of a number of family practitioner (GP) practices.

<u>When and how much of the service is delivered?</u>

Extended access appointments under this study became operational at varying times over the 2016 calendar year, this varied by CCG.

<u>Tailoring of the service</u>

The type, referral route(s) and timing of appointments are decided by the local CCG. Variations between schemes existed by referral route, whether pre-bookable appointments were available, discipline of appointment (GP or nurse, all face to face), time and day of appointments.

Extended access evaluations to date have found reductions in emergency service use for patients with minor injuries/issues in national[8], sub-national[9], and Greater Manchester[6, 10] schemes. However, the reductions in emergency service use alone may not sufficiently offset the cost of providing an extended service, raising concerns that an extended access service is not a cost-effective use of health system resources.[10] Concerns of opportunity cost (the net benefits of services that could have been provided instead of extended access) are particularly pertinent in times of increasing financial/workforce pressures for many health systems. However, in addition to impacts on secondary care, and particularly in the current absence of an understanding of the long-term health benefits of the service; there are additional aspects of extended access that need to be considered when assessing the value of the service. First, there is a lack of knowledge concerning how the service is being used with little assessment of how best to operationalise extended appointments efficiently (which would have implications for cost-effectiveness). One way to measure efficiency could be uptake of the service, with unbooked appointments signalling inefficient use of resources. Uptake of extended access appointments has been reported as 71% with lower uptake on weekends, particularly Sundays.[8, 11] A sub-national evaluation of 3 schemes found uptake was lower where appointments were not pre-bookable, and lower on Sundays.[11] An evaluation of the initial wave of Greater Manchester ‘demonstrators’ found uptake of 65.5% and lower uptake on weekends, particularly on Sundays.[6] Studies of uptake have concentrated on high level aggregated descriptive statistics of uptake over time and/or by day of week. Since service provision varies across pilots it is also important to understand how uptake varies across services delivered. Commissioners are tasked with delivering extended access services in light of patient demand[5, page 48], the lack of evidence to inform efficient provision is a substantial gap in knowledge. Second, it is currently unknown what the patient

For peer review only - http://bmjopen.bmj.com/site/about/guidelines.xhtml

demographics are for an extended access service delivering pre-bookable and same-day appointments with existing analyses being for small scale schemes[11] or urgent care appointments only[12], it is also unclear whether patients using the service differ from patients using core hours general practice, understanding these may help inform service provision and infer whether the service is hitting an unmet need and/or reducing pressures in core hours.

Our aim in this paper is threefold. First, using data from Greater Manchester UK schemes throughout 2016 we sought to understand whether the service is being used and whether this varies by day of week and over time. Second, we sought to understand whether there were differences in the services being used by particular age and gender groups. Third, we aimed to compare the users of extended access appointments to users of general practice core hours. The findings from these exploratory analyses will help inform considerations about extending access to general practice and how such a service might be better targeted to particular population groups.

METHODS

Data

The study evaluated the second wave of Greater Manchester schemes that delivered extended access throughout 2016. Out of Greater Manchester's ten Clinical Commissioning Group (CCG) areas, three were already delivering extended access under the national GP Access Fund (GPAF) scheme during the study period (see Figure 1). In line with the GM Health and Social Care Partnership Primary Care Strategy, the other seven CCG areas, not previously in receipt of Wave 1 Prime Minister's Challenge Fund (PMCF) or Wave 2 GPAF funding, received financial support from GMHSCP to implement extended access. However, only five of these seven CCGs were included in the evaluation because two of the CCGs were not captured in the data collection process: one had not implemented the service in 2016; the other did not provide data. A table summarising the five CCG schemes with extended access can be found in Supplementary Table S1. The evaluation was carried out by the National Institute for Health Research Collaboration for Leadership in Applied Health Research and Care Greater Manchester (NIHR CLAHRC GM) and comprised of assessments of activity, a process evaluation (seeking to understand the organisational and operational issues that arose in the implementation of the service and how areas addressed these), and assessments of impacts on other sectors of the healthcare system.[13] This study builds on the assessment of activity contained within the evaluation report.

<Figure 1 here>

For peer review only

We used administrative data on extended access appointments available, booked and attended in CCG schemes in Greater Manchester by calendar date over the year 2016 (January to December). The NIHR CLAHRC GM evaluation team requested a minimum dataset from each CCG (Supplementary Table S2). The aim of this data collection was to inform what new appointment activity was associated with the extended access schemes, whether this new activity was used, and to understand the types of patients using the service (a protocol for the evaluation can be found in Supplementary Text S1). Data on each appointment made available were collected and made available by the responsible CCG. CCGs submitted the data to NIHR CLAHRC GM throughout the period. The data included information on date, use (volume available, booked, booked and used), type of appointment (family practitioner or nurse practitioner, pre-booked (booked on a previous calendar date) or booked same-day), and patient characteristics (the patient's registered practice, age and gender) for booked and attended appointments. Data quality and coverage was dependent on the CCG submitting the data, whilst NIHR CLAHRC GM could raise instances of incomplete data with CCGs it was up to the CCG to respond. Patient characteristics were limited due to issues of patient confidentiality. To maintain anonymity, patient age was given in 10-year age bands. Deprivation statistics were considered although, due to data confidentiality, we were only able to obtain partial patient postcodes. Since partial postcodes do not enable a suitable deprivation measure to be derived, no deprivation analysis was conducted for the analyses. We were unable to obtain health status or outcomes of patients attending the service.

Appointment data for each CCG area was collected and analysed once a hub became operational. Live dates varied across CCGs, and within CCGs there was variation in hub start dates. A patient residing within their CCG could use any hub services within the CCG.

Data on general practice core hour use was sourced from the GP Patient Survey (GPPS).[14] This is a cross-sectional survey of randomly selected patients from each practice within England. Patients need to be registered with a practice, have an NHS number, and be aged 18 and over to be sampled. Patients are asked questions concerning the use of primary care, perceptions of primary care, and patient demographics.

Analysis

All estimation was conducted in Stata (version 14). We assessed uptake, variations in patient demographics for different types of extended access appointments, and made comparisons of extended access appointment users to core hour users as follows:

For peer review only

(i) Uptake

Patients could book an appointment which could then be attended or not. Focussing on booked appointments alone would give an indication of the demand for the service, whilst assessments of appointments actually used shed light on actual impacts on service use. We estimated two separate sets of probability models:

1) the probability an appointment was booked, and;
2) the probability an appointment was booked and attended ('used').

Each model was estimated via probit regressions (see Supplementary Text S2 for a technical description of the methods). Regression models were estimated to enable adjustment for multiple predictor variables, for example, CCG effects which may be important given the variation in schemes across CCGs (Supplementary Table 1). Probit models were used to estimate differences in the probability (rate) of uptake across categories of the predictor variables. The estimates give absolute percentage point differences across predictor variables which is more informative than relative-odds ratios for understanding the scale of uptake.

To assess whether uptake of the service varied across day of the week we included day of week dummies in the regression, with the estimates providing the differences in the rate of uptake relative to the base week day (Monday). This enabled a test for whether uptake was lower on particular days. To assess whether there was evidence of assimilation to the service we included calendar month dummies, if assimilation is evident the estimates on the calendar month dummies should be monotonically increasing over the period. To account for potential confounding caused by variations in the provision and delivery of services across schemes, we included CCG dummies (fixed-effects) in the regression. Robust standard errors were estimated to address concerns of heteroscedasticity and clustered at CCG. Estimates are presented as average marginal effects, which give the average percentage point effect on uptake of the variable relative to the base category across the sample.

Analyses were restricted to four CCG schemes due to the inability to differentiate whether a booked appointment had been attended in one CCG (CCG1).

(ii) Analysis of demographics of patients booking an extended access appointment

Our second set of analyses concentrated on booked appointments (both used and not attended) and exploited variations in service delivery to test whether there were variations in patient demographics for particular types of services. We estimated three separate sets of probability models:

For peer review only - http://bmjopen.bmj.com/site/about/guidelines.xhtml

1) The probability an appointment was pre-booked versus same day;
2) The probability an appointment was for a GP versus a nurse practitioner;
3) The probability an appointment was used versus not attended (DNA)

In each model the unit of analyses is the appointment, we included dummies for gender (female) and 10-year age bands of the patient booking the appointment, and day of the week of the appointment, month of the appointment, and CCG. Each model was estimated via probit regression (see Supplementary Text S2 for a technical description of the methods). For all models, estimates are presented as average marginal effects, with standard errors clustered at GP practice of the patient booking the appointment. We are unable to identify whether a person booked multiple appointments in the data and therefore assume independence of observations. Schemes that exclusively provided a certain service (e.g. only GP appointments) were excluded as a result of uptake of the alternative being necessarily zero.

Missing data on booked appointments were treated as missing at random. To evaluate whether or not missing data might have been associated with the covariates in our models (and hence potentially bias our estimates) we generated a binary missing data indicator for each appointment booked. The binary missing data indicator was coded as 1 where missing data existed for either: age, gender, appointment type, booking type, or the GP practice of the patient booking the appointment. This was regressed via a probit model against day of the week, calendar month, and CCG indicators to test whether missing data was related to particular days or months of provision and/or the unit of delivery (CCG).

(iii) Comparisons of extended access appointment users to core hour users

Finally, to inform the potential implications of the service on equity of access to primary care, the demographics of extended access patients that booked and used their appointment were compared to age and gender distributions of patients reporting using their practice within the last year from the January-March 2013 wave of the GP Patient Survey (GPPS).[14] The GPPS is a patient satisfaction survey and as such is not specifically aimed at capturing the volume of use of general practice services, however, in the absence of data on general practice use and demographics of users in England, the survey presents the most appropriate dataset for comparison. Patients reporting in the January to March 2013 wave were selected to proxy for patient demographics in usual care. Later waves of the GPPS could cause potential bias as patients reporting having used primary care may have used an extended access service which had been available in areas of Greater Manchester since 2014. The

For peer review only - http://bmjopen.bmj.com/site/about/guidelines.xhtml

GPPS sample was reduced to patients registered with a practice within one of the four CCG areas under evaluation and further reduced to those patients using primary care over the last year. These patients were determined via responses to Question 1 of the survey: "*When did you last see or speak to a GP from your GP surgery?*", with patients reporting "*In the past 3 months*", "*Between 3 and 6 months ago*", and "*Between 6 and 12 months ago*". The age groupings of these respondents did not align with the age groupings in the appointment data. As such, to ease comparisons, population pyramids are presented to give an overall feel for similarities and/or differences between the two groups of patients. The GPPS may suffer from response bias. We therefore weight practice patient use measures in this survey by the sampling weights provided with the GPPS dataset for non-response related to age and gender.[14]

Patient and public involvement

A panel of patients reviewed an earlier draft of the protocol, where feasible the protocol was adapted in light of patient feedback. The response from patients in relation to the activity analyses focussed on being able to capture variations by day of week and type of appointment, no changes in data capture was necessary following patient responses as these concerns were anticipated to be addressed in the appointment activity analyses (see Supplementary Text S3).

RESULTS

Uptake

A total of 51,806 extended access appointments were provided throughout 2016 by the five CCGs under the Greater Manchester scheme (Table 1), 41.07 appointments per 1,000 of the population (the population covered by these CCGs is 1,261,326). 2,315 of these, across two CCGs, were 'blocked' and not bookable by patients resulting in a total of 49,491 bookable appointments. CCG1 was removed from the analysis since no data on whether the patient attended was provided leaving a sample of 42,472 (49,491 appointments – 7,019 CCG1 appointments). 75.44% of 42,472 bookable appointments were booked and 65.33% were booked and attended. Provision and uptake varied across the CCGs, with CCG2 dominating provision and scale per 1,000 of the population and CCG5 having highest percentage use. The types of appointments differed across the CCG schemes (Table 2). Two of the four schemes provided both GP and nurse appointments, the other GP only, and one scheme provided only pre-bookable appointments. Of all appointments booked, 53.27% were pre-booked and 81.42% were for a GP appointment.

Table 1 Volume of appointments bookable, booked, used and Did Not Attends (DNAs)

Provider	Provided (Rates per 1,000, Population (P) size)	Blocked	Available	Not booked*	Booked* (% of available)	DNAs** (% of available)	Used* (% of available)
CCG1	7,019 (23.23 per 1,000, P=302,163)	0	7,019	1,500	5,519 (78.63)		
CCG2	32,693 (143.77 per 1,000, P=227,391)	1,911	30,782	7,437	23,345 (75.84)	2,959 (9.61)	20,380 (66.21)
CCG3	3,637 (14.63 per 1,000, P=248,667)	0	3,637	1,587	2,050 (56.37)	202 (5.55)	1,848 (50.81)
CCG4	5,600 (22.92 per 1,000, P=244,325)	0	5,600	1,137	4,463 (79.70)	758 (13.54)	3,699 (66.05)
CCG5	2,857 (11.96 per 1,000, P=238,780)	404	2,453	270	2,183 (88.99)	362 (14.75)	1,820 (74.19)
All schemes	51,806 (41.07 per 1,000, P=1,261,326)	2,315	49,491	11,931	37,560 (75.89)		
Schemes CCG2 to CCG5	44,787 (46.84 per 1,000, P=956,163)	2,315	42,472	10,431	32,041 (75.44)	4,281 (10.08)	27,747 (65.33)^

CCG5 blocked are for administrative purposes.
*Booked are appointments that were used (can be either pre-booked or booked same-day).
**CCG1 did not record DNAs we assume all attended for the GM % booked and used; Missing data on DNA evident for 6 appointments in CCG2, 6 in CCG4, and 1 in CCG5.
^as a percentage of appointments where attendance was captured (42,472 appointments: 49,491 appointments minus 7,019 CCG1 appointments)

Table 2 Volume of appointments by appointment type (GP/nurse) and booking type (pre-booked/same-day)

Provider	Appointments available	GP*	% GP	Nurse*	% Nurse	Appointments booked	Pre-booked**	% Pre-booked	Same-day**	% Same-day
CCG2	30,782	24,252	78.79	6,530	21.21	23,345	9,805	42.00	13,540	58.00
CCG3	3,637	3,637	100.00	0	0.00	2,050	524	25.56	1,526	74.44
CCG4	5,600	5,255	93.84	0	0.00	4,463	2,460	55.12	2,003	44.88
CCG5	2,453	1,438	58.62	1,015	41.38	2,183	2,180	99.86	0	0.00
Total	42,472	34,582	81.42	7,545	17.76	32,041	14,969	46.72	17,069	53.27

Pre-booked appointments are appointments booked on a previous calendar date
Information on pre-booked or same-day only available for booked appointments
*GP/Nurse contains missings for CCG4 (345 appointments)
** CCG5 have 3 appointments with no data on booked time.

Figure 2 plots appointments used, appointments booked and not attended ('DNA'), and appointments not booked over the 2016 calendar year. Use appears to have improved slightly over time. 15,575 appointments (36.67%) occurred on a Saturday, and Sunday was the second most active day (5,878, 13.84%) (Figure 3). 52.93% of appointments on a Sunday were booked (46.73% used), and 75.06% (63.94% used) on a Saturday. The percentage of appointments booked on weekdays was generally higher at 73.29-86.06% (65.94-75.46% booked and used) compared to weekend appointments at 52.94% (Sundays) and 75.06% (Saturdays) (46.73% (Sundays) and 63.94% (Saturdays) booked and used).

For peer review only - http://bmjopen.bmj.com/site/about/guidelines.xhtml

For peer review only

<Figure 2 here>

<Figure 3 here>

The results from the probability models of appointments booked and appointments booked and used are contained in Table 3 (unadjusted analyses are presented in Supplementary Table S3). Estimates for the probability models are interpreted as the percentage point difference in the rate of the outcome measure relative to the base category. For example, the estimate for Tuesday in the 'Probability model for appointment booked' model is interpreted as the rate of appointments booked on a Tuesday were 11.45 percentage points higher than the rate of appointments booked on Monday (the base category). The results broadly confirm the summary statistics with Monday having the lowest percentage of appointments booked of other weekdays (Tuesday-Friday), and Sunday having lowest percentage of appointments booked overall (-18.93 percentage points compared to Monday (95% CI: -35.03 to -2.83)). The results are similar for appointments used. The percentage of appointments booked and used over the year was greater for all months compared to January and appear to be greater in the second half of the year, however, the increase over time is small and inconsistent with lower rates of appointments booked and used in May, June, August and November and December compared to neighbouring months. There were differences in uptake at CCG level with all CCGs having lower percentages of appointments booked and used compared to CCG5. For comparison, Supplementary Table S4 replicates the analyses with estimation via logistic regression and confirms the same sets of estimates that are significantly different from zero in the probit regressions are significant different from one in the logistic regression. The relative size of magnitude of the estimates are also similar across models.

Table 3 Rates (%) and probability models for appointments booked and appointments booked and used

	% booked	Probability model for appointment booked (95% CI)	% booked and used	Probability model for appointment booked and used (95% CI)
**Day of week**				
**Monday (base category)**	73.29		65.94	
**Tuesday**	84.84	11.45 (8.51, 14.38)	75.46	9.60 (7.45, 11.75)
**Wednesday**	84.06	11.06 (5.89, 16.24)	74.14	8.45 (3.32, 13.58)
**Thursday**	86.06	12.58 (10.05 ,15.10)	73.07	7.04 (4.64, 9.44)
**Friday**	83.21	10.05 (7.12, 12.98)	69.96	4.19 (0.88, 7.51)
**Saturday**	75.06	-0.10 (-8.44, 8.24)	63.94	-3.47 (-10.97, 4.01)
**Sunday**	52.94	-18.93 (-35.03, -2.83)	46.73	-18.07 (-32.46, -3.68)

For peer review only - http://bmjopen.bmj.com/site/about/guidelines.xhtml

**Calendar month**				
**January (base category)**	49.52		43.87	
**February**	73.45	22.73 (13.25, 32.20)	64.33	19.80 (13.15, 26.45)
**March**	73.45	20.92 (11.80, 30.04)	63.39	17.56 (12.25, 22.88)
**April**	78.48	26.84 (11.48, 42.20)	68.31	23.42 (11.91, 34.94)
**May**	73.17	22.76 (7.90, 37.62)	63.69	19.21 (8.32, 30.10)
**June**	73.82	22.45 (5.40, 39.50)	65.31	20.45 (7.37, 33.52)
**July**	79.37	29.08 (16.09, 42.07)	68.05	24.13 (13.98, 34.27)
**August**	73.52	22.60 (4.12, 41.07)	64.00	19.11 (4.95, 33.28)
**September**	81.25	30.54 (12.66, 48.43)	69.64	25.26 (11.19, 39.32)
**October**	82.25	32.26 (15.89, 48.62)	72.12	28.11 (15.33, 40.89)
**November**	80.85	29.71 (12.79, 46.62)	69.56	24.75 (11.59, 37.91)
**December**	80.74	29.42 (6.82, 52.01)	67.40	23.01 (5.75, 40.26)
**CCG scheme**				
**CCG1***				
**CCG2**	75.48	-16.63 (-20.09, -13.17)	66.21	-10.43 (-13.17, -7.68)
**CCG3**	56.37	-32.02 (-35.24, -28.80)	50.81	-23.85 (-26.36, -21.35)
**CCG4**	79.70	-11.28 (-14.69, -7.87)	66.05	-9.97 (-12.55, -7.38)
**CCG5 (base category)**	88.99		74.19	
**Sample size**	42,472	42,472	42,472	42,472

Appointments booked are appointments booked, appointments booked and used are appointments that were booked and subsequently attended.
Probability models are probit regressions of appointment status against day of week, calendar month, and CCG scheme. Standard errors are clustered at the CCG-level.
Estimates are presented as average marginal effects which give the percentage point effect of the variable relative to the base category.
*CCG1 did not provide data to enable identification of whether a booked appointment was subsequently attended so does not feature in the analysis

To test whether the percentage of appointments booked and used on a Sunday improved over and above the general trend over the period we replicated our analysis with the inclusion of Sunday-calendar month interaction terms (Supplementary Table S5). The interaction terms were not significantly different from zero suggesting any change over time was similar to that observed Monday to Saturday in the data.

Analysis of demographics of patients booking an extended access appointment

The analyses concerning variations in patient demographics for particular types of appointments is limited to booked appointments (used and DNA) (Figure 1). Of the 37,560 booked appointments, 5,519 were removed due to data reporting by CCG1, giving 32,041 appointments over the remaining four CCGs. Missing data on appointment type, booking type, and patient's gender and age and GP practice amounted to a further 2,881 appointments being excluded from the analyses (Supplementary Table S6) giving a sample of 29,160 booked appointments. Missing data varied by CCG but was similar

For peer review only - http://bmjopen.bmj.com/site/about/guidelines.xhtml

within CCGs by hub. One CCG provided 100% complete data. Missing data appears to be associated with calendar month, day of week (Wednesday and Sunday), and CCG (Supplementary Table S7).

Of the 31,154 booked appointments with complete data, 46.43% of booked appointments were pre-booked, 82.54% were for a GP, and 12.17% resulted in a patient not attending (Supplementary Table S8). 58.60% of booked appointments were made by female patients. 55.91% of users were aged under 39 (71.48% under 49). Estimates from our second set of analyses concerning patient demographics for types of appointments booked are contained in Table 4. Across all models there were significant CCG effects; this is likely to reflect variations in service delivery due to the variations in relative scale of booking type and appointment type (Table 2).

Table 4 Patient demographics by booking and appointment type

	**Pre-booked v Same-day^ (95% CI)**	**GP v nurse^^ (95% CI)**	**DNA v used (95%CI)**
**Gender**			
**Male (base category)**			
**Female**	-0.73 (-2.01, 0.55)	-4.29 (-6.42, -2.16)	-0.32 (-1.40, 0.77)
**Age band (years)**			
**0-9 (base category)**			
**10-19**	13.39 (10.14, 16.64)	-19.37 (-25.83, -12.91)	0.66 (-1.08, 2.40)
**20-29**	14.45 (11.28, 17.63)	-26.45 (-32.34, -20.56)	0.32 (1.65, 4.74)
**30-39**	14.53 (11.79, 17.26)	-29.45 (-35.79, -23.10)	0.97 (-0.79, 2.74)
**40-49**	15.03 (11.72, 18.35)	-31.86 (-39.36, -24.36)	-3.20 (-4.95, -1.44)
**50-59**	17.71 (14.52, 20.90)	-31.89 (-39.46, -24.32)	-7.04 (-9.07, -5.01)
**60-69**	13.25 (9.09, 17.41)	-34.25 (-43.65, -24.86)	-12.05 (-14.29, -9.81)
**70-79**	12.60 (8.78, 16.42)	-37.40 (-47.70, -27.10)	-10.20 (-13.16, -7.23)
**80-89**	14.00 (9.45, 18.55)	-41.26 (-52.96, -29.55)	-7.86 (-10.91, -4.81)
**90+**	8.83 (-5.79, 23.45)	-41.39 (-63.28, -19.49)	-
**Day of week**			
**Monday (base category)**			
**Tuesday**	20.49 (14.45, 26.53)	-0.63 (-5.05, 3.79)	-0.57 (-2.12, 0.98)
**Wednesday**	27.02 (23.30, 30.74)	3.32 (0.01, 6.63)	-0.59 (-2.32, 1.14)
**Thursday**	24.39 (19.00, 29.78)	-2.24 (-7.15, 2.67)	2.77 (1.33, 4.21)
**Friday**	25.59 (20.88, 30.30)	1.99 (-1.61, 5.59)	3.47 (1.62, 5.32)
**Saturday**	24.23 (18.91, 29.56)	2.32 (-2.61, 7.24)	2.22 (0.66, 3.77)
**Sunday**	24.98 (20.62, 29.33)	0.75 (-2.31, 3.81)	-0.37 (-2.10, 1.37)

For peer review only - http://bmjopen.bmj.com/site/about/guidelines.xhtml

**Appointment type**			
**Nurse (base category)**			
**GP**	-12.97 (-19.49, -6.45)	-	1.11 (-0.63, 2.85)
**DNA status**			
**Used (base category)**			
**DNA**	19.08 (16.11, 22.04)	1.97 (-0.39, 4.32)	-
**Booking type**			
**Same-day (base category)**			
**Pre-booked**	-	-7.46 (-12.29, -2.62)	8.48 (7.24, 9.73)
**CCG scheme**			
**CCG2**	-14.90 (-23.30, -6.50)	15.22 (7.37, 23.07)	2.94 (0.61, 5.27)
**CCG3**	-27.19 (-36.88, -17.51)	-	0.96 (-2.26, 4.18)
**CCG4**	Base	-	0.15 (-2.76, 3.05)
**CCG5**	-	Base	Base
**Sample size**	27,035	23,429	29,120

Pre-booked appointments are appointments booked on a previous calendar date
Probability models are probit regressions of appointment against day of week, calendar month, appointment characteristics, patient age and gender, and CCG scheme. Standard errors are clustered at the patient's GP practice-level. Month estimates omitted from the output but available upon request.
Estimates are presented as average marginal effects which give the percentage point effect of the variable relative to the base category.
^CCG5 had 100% pre-booked and GP appointments (n=2,125 with complete data) and was omitted from this analysis giving a sample of 29,160-2,125=27,035.
^^CCG4 had 100% GP appointments (n=3,704 with complete data) and CCG3 had 100% GP appointments (n=2,027 with complete data) and both were omitted from this analysis giving a sample of 29,160-3,704-2,027=23,429.

For peer review only

58.16% of pre-booked appointments were booked by females patients and 58.09% of same-day appointments were booked by female patients (Supplementary Table S9), we found no significant difference in the type of appointment booked by females compared to males (-0.73 percentage points less likely to be pre-booked compared to males, 95%CI: -2.01 to 0.55). Appointments booked for patients in age groups 10 to 19 to 80 to 89 were more likely to be pre-booked in comparison to age group 0 to 9 years. Tuesday to Sunday appointments were more likely to be pre-booked compared to Monday. GP appointments were less likely to be pre-booked in comparison to nurse appointments (-12.97 percentage points, 95%CI: -19.49 to -6.45). Patients were more likely to turn up to a same-day booked appointment than appointments that had been booked on an earlier calendar date (DNA rates were higher for pre-booked appointments by 19.08 percentage points, 95%CI: 16.11 to 22.04).

57.56% of GP appointments were booked by females (64.59% of nurse appointments), appointments booked by females were significantly less likely to be with a GP compared to males (-4.29 percentage, 95%CI: -6.42 to -2.16). Appointments booked were less likely to be with a GP as the age of the patient booked for the appointment increases compared to age group 0 to 9. There was limited evidence that

appointments booked on specific weekdays were more or less likely to be for GP appointments. Appointments that were pre-booked were less likely to be GP appointments. Appointments that were not attended were no more or less likely to be GP appointments (1.97 percentage points higher rate for GP appointments compared to used appointments (95% CI: -0.39 to 4.32)).

58.51% of used appointments were booked by females and 59.13% of not attended (DNA) appointments were booked by females, no significant difference was found in the likelihood that appointments booked by females were not attended compared to males (-0.32 percentage points lower likelihood of not attending (95% CI: -1.40 to 0.77)). Appointments booked by age group 20 to 29 had a higher likelihood of not being attended compared to age group 0-9 though this effect is small (0.32 percentage points (95% CI: 1.65 to 4.74)). Appointments booked by age groups above the age of 40 were significantly more likely to be used compared to age group 0 to 9. Appointments booked for a Thursday, Friday or Saturday were more likely to be not attended compared those booked on a Monday.

Comparisons of extended access appointment to core hour users

To compare patient demographics of extended access users to core hour users we focus on appointments that were used. In total, 84.47% of appointment users were aged under 60 years of age (Table 5 and Figure 4). Patients booking and attending extended access appointments were compared to patients reporting attendance at their practice within the past year in the GPPS and the population registered with a practice. To better reflect the sampling frame of the GPPS (patients aged 18 and over), Figure 5 and Supplementary Table S10 provide the percentages of users in extended access appointments from age 20 plus. 80.40% of extended access appointments users aged 20 plus were under 60 years, whilst the equivalent percentage in the GPPS data lies above age 65. Users of extended access appointments tended to be younger in comparison to core hour patients with a more equal distribution of users aged between 20 to 59 compared to GPPS core hour users. For female users, the peak in the distribution of extended access users occurred at age 20-29, compared to 45 to 54 for users in the GPPS. As the age bands do not align in the datasets, these differences could not be tested, and comparisons should be treated with caution.

<Figure 4 here>

<Figure 5 here>

Table 5 Demographic distributions of extended access users

For peer review only - http://bmjopen.bmj.com/site/about/guidelines.xhtml

Age	Males Frequency	%	Cumulative %	Females Frequency	%	Cumulative %	All Cumulative %
**0-9**	1,436	13.53	13.16	1,387	9.26	9.26	11.03
**10-19**	1,018	9.59	23.12	1,489	9.94	19.20	20.82
**20-29**	1,446	13.62	36.74	2,897	19.33	38.53	37.78
**30-39**	1,575	14.84	51.58	2,513	16.77	55.30	53.75
**40-49**	1,617	15.23	66.81	2,435	16.25	71.55	69.58
**50-59**	1,690	15.92	82.73	2,123	14.17	85.72	84.47
**60-69**	1,144	10.78	93.51	1,273	8.50	94.22	93.91
**70-79**	481	4.53	98.04	624	4.16	98.38	98.23
**80-89**	197	1.86	99.90	215	1.43	99.81	99.84
**90+**	12	0.11	100.00	28	0.19	100.00	100.00
**Total**	10,616	100.00		14,984	100.00		

For peer review only

DISCUSSION

Principal findings

Our primary analyses concerned the proportion of extended access appointments used. We found evidence of spare capacity in the extended access service and limited evidence that use improved over the calendar year. The proportion of appointments used was lowest on Sundays. Secondary analyses investigated types of appointments booked (both booked and used and booked and not used) and the patient demographics of users of the service. There were variations in types of patients booking particular types of extended access appointments. Pre-booked appointments were relatively more likely amongst age groups 10 to 89 compared to age group 0 to 9. GP appointments were increasingly less likely to be booked with age. Appointments booked on Thursday to Saturday were relatively more likely to result in non-attendance compared to Mondays. Pre-booked appointments were less likely to be with a GP and more likely to result in non-attendance compared to same-day appointments. In comparison to core hour patients, we found some evidence that patients using extended access appointments were of a younger age relative to patients using primary care services before the scheme. The most obvious difference was found in females aged 20-29 who accounted for almost a quarter of all female extended access appointments users (aged 20+).

Strengths and weaknesses

The study has a number of strengths and weaknesses. Strengths lie in the unique dataset which enabled a detailed look at uptake and users of the service and how this varied with types of appointments provided. In addition, our results are focussed on several Greater Manchester schemes

For peer review only - http://bmjopen.bmj.com/site/about/guidelines.xhtml

offering variation in delivery which enables comparisons of different service models. However, weaknesses are that the findings may not be generalizable to other areas that may provide an extended access service under alternative specifications and/or populations than those detailed in this study. Missing data on patient age, gender, and registered practice resulted in a loss of 8.99% of appointments and this was found to be vary with day of week, calendar month and CCG. Although data issues were flagged by the research team, we could not enforce areas to provide complete data. We were unable to obtain patient deprivation/ethnicity characteristics to enrich understanding of service use. We were also unable to identify whether there are repeat patients in the data, this may bias the comparison of users of the extended access service to those in core hours if repeated use is associated with gender and/or age. We were not able to identify whether an appointment was cancelled and as such, we assume this was subsequently made available. The GPPS comparison is particularly flawed because the measure of core hour use is self-reported and thus may be affected by recall error, children are not included in the survey and age bands did not match with those obtained for appointment data making it not possible to draw direct comparisons of age groups. Finally, access depends on a range of factors, and without further details on CCG provision related to awareness campaigns and buy-in from providers, we can only distinguish such effects by the CCG fixed-effects adjustment factor in our models.

Implications

The presence of spare capacity for a service seeking to improve access to meet excess demand in core hours raises the question of whether or not extended access appointments really are improving access to primary care. However, of the appointments that were used, our findings suggest that the service may be either i) meeting previously unmet need for younger age groups, ii) meeting previously inappropriately met need should these patients have proceeded to use alternative health services such as emergency departments at hospital, or iii) diverting demand for core hours for this age group, thereby potentially reducing pressure in core hours. It is likely that a mixture of these factors are playing a role. We find 46.43% of appointments used were pre-booked which may signify a large proportion of patients are using the service for non-urgent health needs which may suggest i) or iii) plays an important role. 53.57% of appointments booked and used were same-day appointments which may suggest ii) may also be an important factor. More research is needed to understand the relative scale of these factors, such information would give important insights into the value and efficiency of the service. These factors are on the assumption that patients accessing the service had a need for seeking health care services, should patients referred to the service not have a need for healthcare then service use may reflect supply induced demand.

For peer review only - http://bmjopen.bmj.com/site/about/guidelines.xhtml

The reasoning behind spare capacity in the scheme may be informed by three domains of access: availability, acceptability, affordability.[15] These domains of access highlight the complex contributory factors at play in access, involving both patient and practitioner. The extended access schemes provide additional *availability* in the sense of new appointments. However, new appointments alone may not be sufficient to improve access, should patients or practitioners not be prepared to *accept* the service (this could be due to cultural factors and/or patient preferences for example) or should patients not be aware of the service. A process evaluation of the service is contained in the evaluation report of the service and highlights the variation in approaches taken to communicate the service to patients.[13] Identification is needed of whether poor rates of use are due to a lack of patient demand or, of particular concern in areas where practice referrals are made, whether a lack of receptionist/practitioner buy-in is influencing patient (and receptionist) awareness of the service (see for example: Healthwatch Manchester, 2017[16]). Policy needs to consider these additional aspects of access to facilitate use and adapt provision accordingly.

Cost-effectiveness analysis seeks to understand whether a service is an efficient use of healthcare resources, this is performed by identifying the opportunity cost of delivering the service (the net benefits of services that could have been provided by allocating the resources elsewhere). Whilst we do not compare the service to a counterfactual use of resources in this study (such as expanding core hours), our findings shed light on a number of aspects relevant for determining cost-effectiveness of the extended access service. First, it is likely that extended access services will result in some positive measure of benefit to patients. Second, a large proportion of extended appointments are pre-booked, suggesting a large amount of activity in the service is for non-urgent care. As such, existing evaluations, that have focussed on emergency care implications, paint only a partial picture of the impacts of extended access. Third, only 65.33% of appointments available were booked and used which raises concerns relating to how efficient the service is. Providers seeking to determine the types of services available under extended access schemes may consider several factors to more efficiently provide the service in relation to our results : (i) whilst the proportion of appointments used was lowest on a Sunday, the volume of appointments used was similar to those during the week, this suggests the proportion of appointments used on a Sunday can be improved by reducing the volume of Sunday appointments (ii) a completely pre-bookable service may reduce access for the very young and result in higher rates of appointments booked and not attended. (iii) nurse appointments may help address issues of staffing. DNAs were no greater for this type of appointment. However, GP appointments

For peer review only - http://bmjopen.bmj.com/site/about/guidelines.xhtml

were relatively more concentrated amongst age group 0 to 9 and males suggesting a potential need for a GP service to address the needs of younger age and male patients.

The evaluation of the national pilots have found uptake of 71% with reportedly lower uptake on weekends, particularly on Sundays.[8] A more detailed evaluation of three schemes under the PMCF pilots found uptake varied by type of service (same day or pre-bookable) available.[11] Weekend uptake was higher in a scheme providing pre-bookable and same-day appointments (87% on Saturdays and 78% on Sundays) compared to two schemes providing only urgent/same-day appointments (25% and 18% and 48% and 41%). The demographics of the scheme providing both pre-bookable and same-day appointments comprised 3% aged under 5, and 57% aged between 20 and 65. The findings in this paper found schemes with pre-bookable and same-day appointments had a lower rate of booked appointments than schemes with only same-day appointments (the proportion of appointments booked in CCG2 to CCG4 are lower than that of CCG5 which provided only same-day appointments (Table 2)). We also found uptake was lower on Sundays (also in line with the national evaluation). However, in contrast we found age group 20-29 to be the largest group of patients using the service (17%) and a higher proportion of patients from the 20-69 age group (73% compared to 57% aged 20-65). These differences may be due to differences in the patient population, breadth of week covered or scale of the schemes (the relevant scheme evaluated by Windrum et al (2017)[11] was much smaller with three practices and one hub). Evaluations of extended access appointments for urgent care in Sheffield, UK, found users were predominantly female (60.0%) and aged under 60 (85.5%), 19.0% were aged under 5 and 29.3% aged under 16, users were also concentrated in more deprived areas.[12] These findings may reflect the urgent care service delivered in comparison to the current study which offered both same-day and pre-bookable appointments.

Future work

We have not been able to identify implications for core hours or assess whether deprivation or ethnicity are a leading factor of appointment use. Further research is needed to investigate the impacts on demand for core hours services and for deprived populations to shed light on potential implications for access.

An assessment of the types of treatments and conditions being presented at extended access appointments may help inform impacts of the service on patient health and the scale of potential supply induced demand.

For peer review only

Given the pilot nature of these schemes, uptake may have also reflected supply-side buy-in, and reservations concerning promotion of the schemes may have arisen due to uncertainty in future funding. Future evaluations of uptake, once clarity is provided on long-term securement of the extended access service, may help identify the scale of this possibility.

Competing interests statement:
All authors have completed the ICMJE uniform disclosure form at www.icmje.org/coi_disclosure.pdf and have the following competing interests: funding support from NIHR and from Greater Manchester Health and Social Care Partnership for this work; WW reports grant funding for other work from the Department of Health Policy Research Programme and NIHR. No other relationships or activities that could appear to have influenced the submitted work are declared.

Contributors:
WW is guarantor for the work. WW cleaned the data, conceived and conducted the analysis, interpreted the data and composed the first draft of the work and proceeding revisions.
LA critically revised the manuscript, participated in the design of the study, and approved the final version to be published.
PN contributed to interpretation of data, revised the initial and subsequent drafts of the paper critically for content and approved the final version to be published.
CO cleaned the activity data in preparation for the analyses, assisted in interpretation of the findings, and approved the final version to be published.
NR contributed to interpretation of data, revised earlier drafts of the paper, and approved the final version to be published.
KR contributed to interpretation of data, revised the initial and subsequent drafts of the paper critically for content and approved the final version to be published.
DH designed and led the overall project, was directly involved in interpretation of data and approved the final version to be published.
All authors had full access to all of the data (including statistical reports and tables) in the study and can take responsibility for the integrity of the data and the accuracy of the data analysis.

Patient consent:
Patient consent was not required as the study reports no identifiable patient data.

Ethics statement:
The study was reviewed by the University of Manchester's internal University Research Ethics Committee and approved under the low risk procedure and thus did not require UREC approval (ID: AMBS/16/05). The study involves anonymised administrative data and did not impact on the type of care patients received, consent by patients using the service was not required.

Funding statement:
This project was funded by the National Institute for Health Research Collaboration for Leadership in Applied Health Research and Care (NIHR CLAHRC) Greater Manchester and the Greater Manchester Health and Social Care Partnership. The NIHR CLAHRC Greater Manchester is a partnership between providers and commissioners from the NHS, industry and the third sector, as well as clinical and research staff from the University of Manchester. The views expressed in this article are those of the author(s) and not necessarily those of the NHS, NIHR or the Department of Health and Social Care.

For peer review only

Transparency declaration:
WW affirms that the manuscript is an honest, accurate, and transparent account of the study being reported; that no important aspects of the study have been omitted; and that any discrepancies from the study as planned have been explained.

Copyright statement:
The Corresponding Author has the right to grant on behalf of all authors and does grant on behalf of all authors, *a worldwide licence* to the Publishers and its licensees in perpetuity, in all forms, formats and media (whether known now or created in the future), to i) publish, reproduce, distribute, display and store the Contribution, ii) translate the Contribution into other languages, create adaptations, reprints, include within collections and create summaries, extracts and/or, abstracts of the Contribution, iii) create any other derivative work(s) based on the Contribution, iv) to exploit all subsidiary rights in the Contribution, v) the inclusion of electronic links from the Contribution to third party material where-ever it may be located; and, vi) licence any third party to do any or all of the above.

Data sharing statement:
Appointment data: No additional data available.
GP Patient Survey: The GP Patient Survey data are available at https://gp-patient.co.uk/SurveysAndReports

Reporting statement:
A STROBE checklist for observational studies has been adhered to with checklist provided as supplementary material.

For peer review only

References:

[1] World Health Organisation. The world health report: health systems financing: the path to universal coverage. [cited 2010]. Available from: http://www.who.int/whr/2010/en/ [Accessed 01/11/2018]

[2] Schoen C, Osborn R, Doty MM, Bishop M, Peugh J, Murukutla N. Towards Higher-Performance Health Systems: Adults' Health Care Experiences In Seven Countries, 2007. *Health Affairs* 2007;26(6):w717-34.

[3] Schoen C, Osborn R, Squires D, Doty MM. Access, Affordability, And Insurance Complexity Are Often Worse In The United States Compared To Ten Other Countries. *Health Affairs* 2013;32(12):2205-2215.

[4] Kraaijvanger N, van Leeuwen H, Rijpsma D, Edwards M. Motives for self-referral to the emergency department: a systematic review of the literature. *BMC Health Serv Res* 2016;16:685

[5] NHS England. General Practice Forward View. [cited 2016 April]. Available from: https://www.england.nhs.uk/wp-content/uploads/2016/04/gpfv.pdf [Accessed 01/11/2018]

[6] NIHR CLAHRC Greater Manchester. NHS Greater Manchester Primary Care Demonstrators Evaluation. [cited 2015 June]. Available from: https://www.research.manchester.ac.uk/portal/files/32297227/FULL_TEXT.PDF [Accessed 01/11/2018]

[7] Greater Manchester Combined Authority. Delivering Integrated Care across Greater Manchester: The Primary Care Contribution. Our Primary Care Strategy 2016-2021. [cited 2016]. Available from: http://www.gmhsc.org.uk/assets/GMHSC-Partnership-Primary-Care-Strategy.pdf [Accessed 01/11/2018]

[8] MacDonald, SQW. Prime Minister's Challenge Fund: Improving Access to General Practice: Second Evaluation Report to September 2015. [cited 2016]. Available from: https://www.england.nhs.uk/wp-content/uploads/2016/10/gp-access-fund-nat-eval-wave1-sml.pdf [Accessed 01/11/2018]

[9] Dalton P, Pathania V. Can increased primary care access reduce demand for emergency care? Evidence from England's 7-day GP opening. *J Health Econ* 2016;49:193-208.

[10] Whittaker W, Anselmi L, Kristensen S, et al. Associations between extending access to primary care and emergency department visits: a difference-in-differences analysis. *PLoS Med* 2016;13(9):e1002113

[11] Windrum P, Guinery J, Siebert P, et al. CHILL Evaluation of 7-Day GP Access. [cited 2017 Jan 1]. Available from: https://www.nottingham.ac.uk/business/businesscentres/chill/documents/chill-7-day-gp-access-report.pdf [Accessed 01/11/2018]

[12] Kelly SJ, Piercy H, Ibbotson R, et al. Who attends out-of-hours general practice appointments? Analysis of a patient cohort accessing new out-of-hours units. *BMJ Open* 2018. doi:10.1136/bmjopen-2017-020308

For peer review only

[13] NIHR CLAHRC Greater Manchester. GM Primary Care 7-Day Access Evaluation. [cited 2017 March]. Available from: https://www.clahrc-gm.nihr.ac.uk/media/Resources/OHC/GM-Primary-Care-7-day-access-report-evaluation.pdf [Accessed 29/05/2019]

[14] Ipsos MORI. Technical Annex for the GP Patient Survey 2012-2013 Annual Report. [cited 2013 June 13]. Available from: https://gp-patient.co.uk/downloads/archive/2013/June/June%202013%20Technical%20Annex.pdf [Accessed 01/11/2018]

[15] McIntyre D, Thiede M, Birch S. Access as a policy-relevant concept in low- and middle-income countries. *Health Econ Policy Law* 2009;4(2):179-193

[16] Healthwatch Manchester. Week spot? Review of Access to the 7 Day GP Service. [cited 2017 November]. Available from: https://d2jlsms9zhgfok.cloudfront.net/2017/12/07142053/Week-Spot-Access-to-7-Day-GP-Service-Review.pdf [Accessed 01/11/2018]

Figures

Figure 1 Flow chart of population coverage

*There are 13 appointments booked that contained no information on attendance

Figure 2 Volume and percentage of appointments used, DNA and not booked by month, all schemes

Excludes CCG1 activity
Used – appointments booked and attended
DNA - appointments booked but not attended

Figure 3 Volume and percentage of appointments used, DNA and not booked by day of week, all schemes

Excludes CCG1 activity
Used – appointments booked and attended
DNA - appointments booked but not attended

Figure 4 Profile of extended appointment users by age and gender

Figure 5 Profile of extended appointment users and core hours users, by age and gender

GPPS: GP Patient Survey (January-March 2013 wave)
GPPS data are patients reporting having spoken to or seen a GP from their practice in the past 12 months

For peer review only - http://bmjopen.bmj.com/site/about/guidelines.xhtml

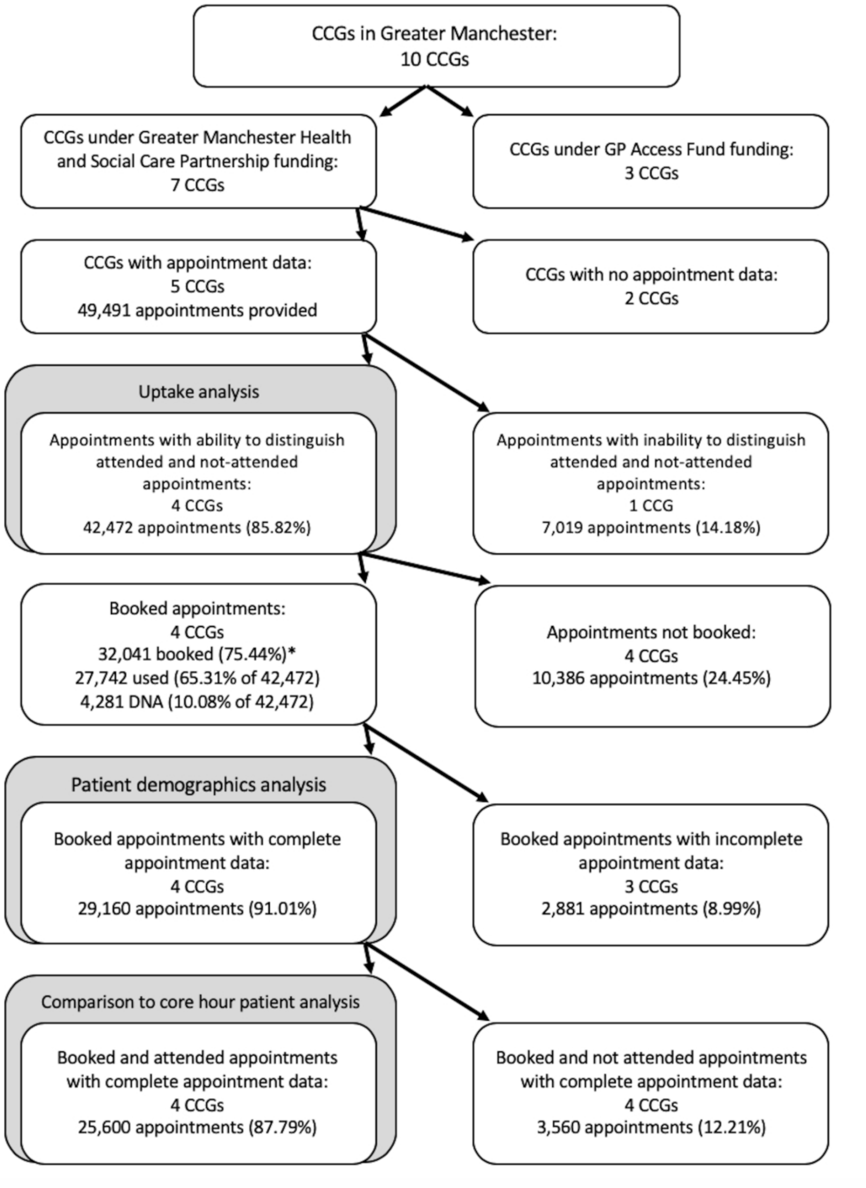

Figure 1 Flow chart of population coverage

156x213mm (300 x 300 DPI)

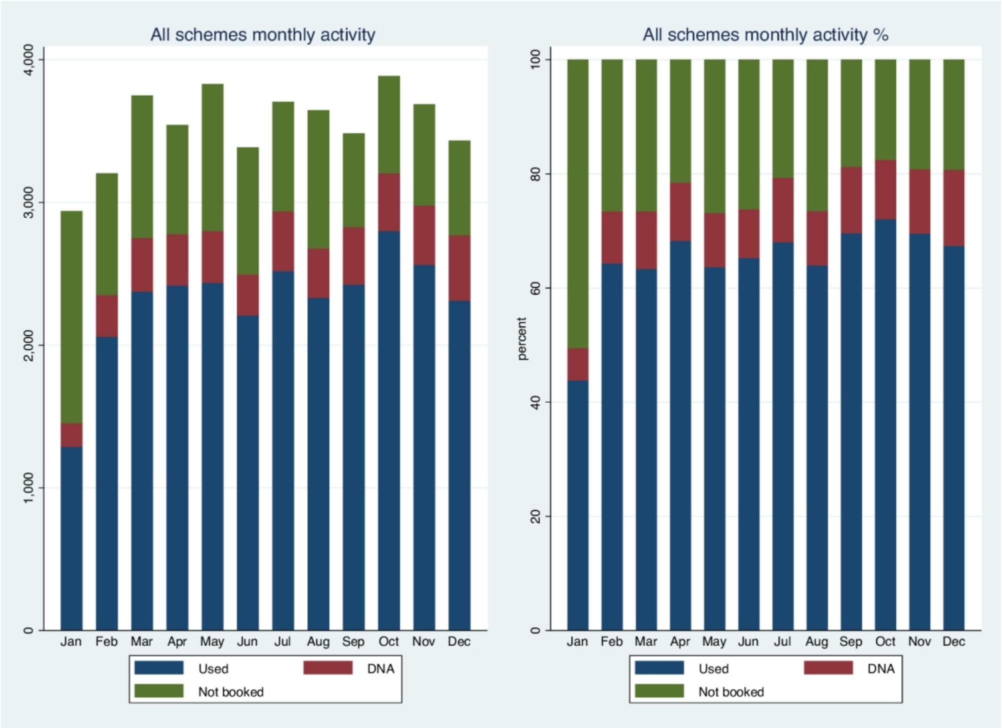

Figure 2 Volume and percentage of appointments used, DNA and not booked by month, all schemes

138x101mm (300 x 300 DPI)

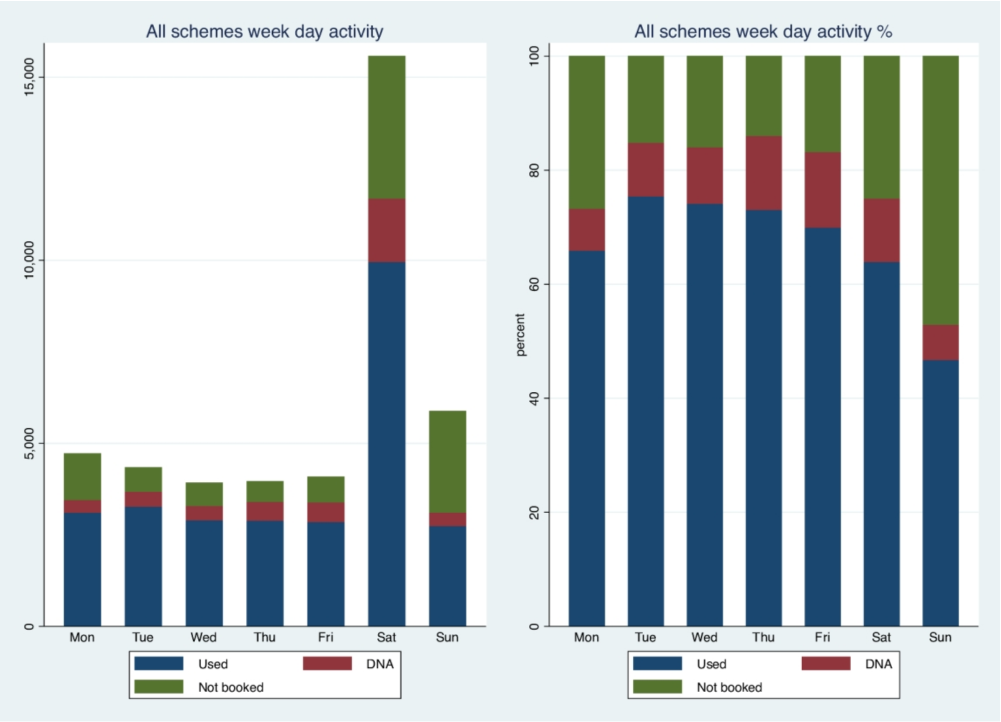

Figure 3 Volume and percentage of appointments used, DNA and not booked by day of week, all schemes

142x102mm (300 x 300 DPI)

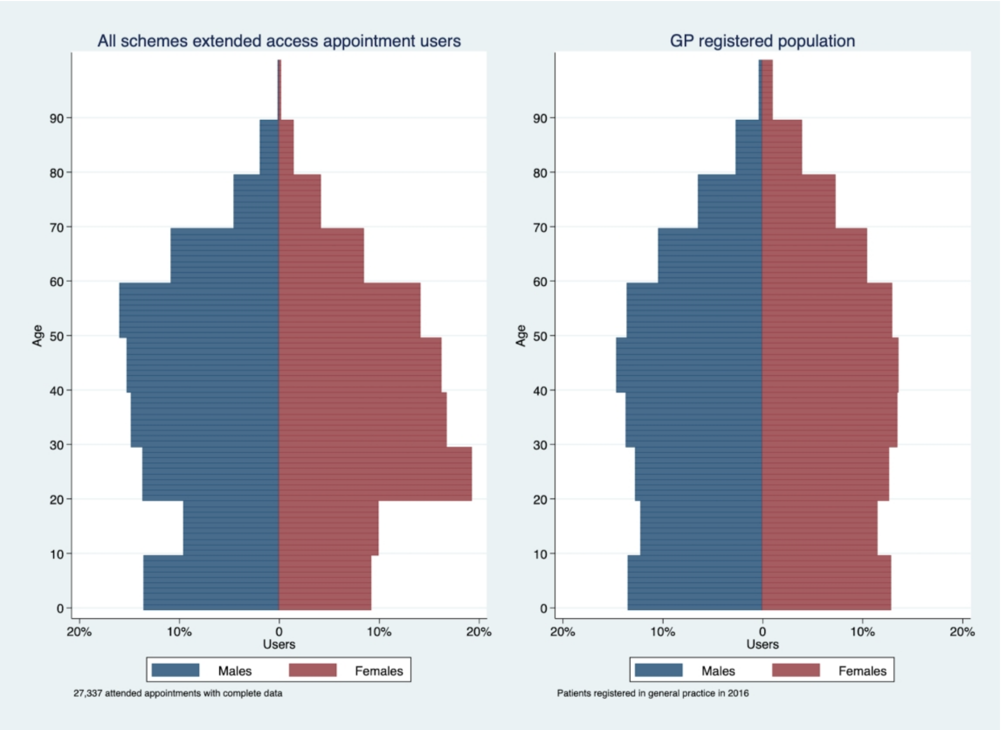

Figure 4 Profile of extended appointment users by age and gender

173x126mm (300 x 300 DPI)

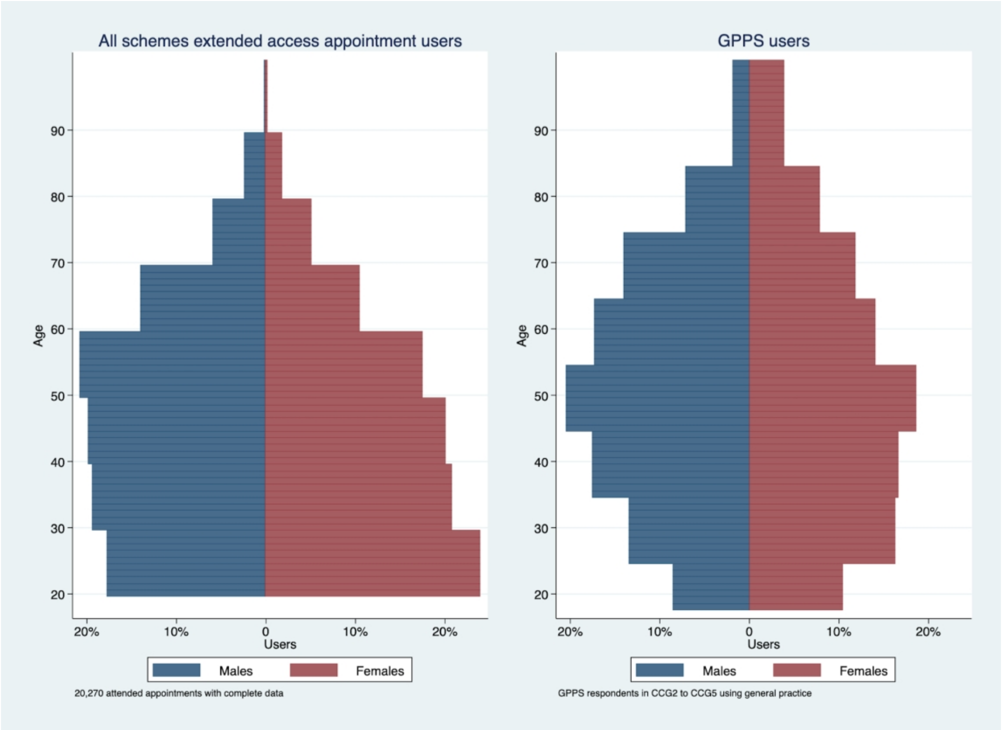

Figure 5 Profile of extended appointment users and core hours users, by age and gender

173x126mm (300 x 300 DPI)

Supplementary Table S1 Summary of 7 Day Access Implementation by Area

Clinical Commissioning Group	Live date (2016)	Service	Self-referral	Monday-Friday opening	Saturday opening	Sunday opening
**CCG1**						
**Hub 1**	2 April	GP and nurse	No	No	09:00-13:00	10:00-13:00
**Hub 2**	2 April	GP and nurse	No	No	09:00-13:00	10:00-13:00
**CCG2**						
**Hub 1**	11 January	GP and nurse	Yes	18:30-21:00	08:00-18:00	10:00-13:00
**Hub 2**	1 January	GP and nurse	Yes	18:30-21:00	08:00-18:00	10:00-13:00
**Hub 3**	2 January	GP and nurse	Yes	18:30-21:00	08:00-18:00	10:00-13:00
**Hub 4**	2 January	GP and nurse	Yes	18:30-21:00	08:00-18:00	10:00-13:00
**CCG3**						
**Hub 1**	5 January	GP only	Yes	18:30-20:00	10:00-14:00	10:00-14:00
**Hub 2**	10 August	GP only	Yes	18:30-20:00	10:00-14:00	No
**CCG4**						
**Hub 1**	3 January	GP only	No	18:30-20:00	09:00-11:45	09:00-12:00
**Hub 2**	30 January	GP only	No	18:15-20:00 (no Thursday or Friday)	09:00-11:45	09:00-11:45
**Hub 3**	25 February	GP only	No	18:30-20:00	09:00-11:45	09:00-12:00
**CCG5**						
**Hub 1**	16 January	GP and nurse	No	No	09:00-13:00	No
**Hub 2**	16 January	GP and nurse	No	No	09:00-13:00	No

For peer review only

Supplementary Table S2 Activity Capture Minimum Dataset

Data requested from Clinical Commissioning Group	Format
Volume of appointments available	
Date	DD/MM/YYYY
Time of appointment	HH:MM
Discipline	GP/Nurse
Planned/pre-booked vs same day/urgent	Pre-booked/Same day
Method of appointment (face-to-face/phone)	Face-to-face/phone
Volume of appointments booked and booked and attended	
Date	DD/MM/YYYY
Time of appointment	HH:MM
Discipline	GP/Nurse
Age	10 year age bands: 0-9 to 90+
Gender	Male/Female
Registered GP practice	Practice code identifier
Postcode (first part)	First half
Planned/pre-booked vs same day/urgent	Pre-booked/Same day
Method of appointment (face-to-face/phone)	Face-to-face/phone
Method of appointment (face-to-face/phone)	Face-to-face/phone

For peer review only

Supplementary Table S3 Rates of appointment use

	Unadjusted appointment booked (95% CI)	Unadjusted appointment booked and used (95% CI)
**Day of week**		
**Monday (base category)**		
**Tuesday**	11.54 (7.90, 15.19)	9.52 (6.51, 12.53)
**Wednesday**	10.77 (3.57, 17.76)	8.20 (1.75, 14.65)
**Thursday**	12.76 (9.45, 16.08)	7.14 (3.96, 10.31)
**Friday**	9.92 (7.19, 12.65)	4.02 (0.62, 7.42)
**Saturday**	1.76 (-6.96, 10.49)	-2.00 (-9.26, 5.27)
**Sunday**	-20.35 (-36.51, -4.19)	-19.21 (-34.36, -4.05)
**Calendar month**		
**January (base category)**		
**February**	23.93 (15.15, 32.71)	20.46 (13.88, 27.04)
**March**	23.93 (17.22, 30.64)	19.52 (15.68, 23.37)
**April**	28.96 (14.72, 43.19)	24.44 (13.63, 35.25)
**May**	23.65 (11.06, 36.24)	19.82 (10.34, 29.29)
**June**	24.29 (8.32, 40.26)	21.43 (8.97, 33.89)
**July**	29.84 (17.46, 42.23)	24.18 (14.12, 34.24)
**August**	23.99 (6.93, 41.06)	20.12 (6.88, 33.37)
**September**	31.72 (13.78, 49.67)	25.77 (11.45, 40.09)
**October**	32.99 (17.24, 48.74)	28.24 (15.93, 40.56)
**November**	31.32 (14.84, 47.80)	25.69 (12.83, 38.54)
**December**	31.22 (10.11, 52.32)	23.52 (7.00, 40.04)
**CCG scheme**		
**CCG1***		
**CCG2**	-16.17 (-16.17, -16.17)	-8.46 (-8.46, -8.46)
**CCG3**	-32.83 (-32.83, -32.83)	-23.02 (-23.02, -23.02)
**CCG4**	-12.17 (-12.17, -12.17)	-8.62 (-8.62, -8.62)
**CCG5 (base category)**		
**Sample size**	42,472	42,472

Unadjusted estimates obtained via probit regression of appointment status against each set of dummies (estimated separately): day of week, calendar month, and CCG scheme.
Estimates are presented as average marginal effects which give the percentage point effect (when multiplied by 100) of the variable relative to the base category.
*CCG1 did not provide data to enable identification of whether a booked appointment was subsequently attended so does not feature in the analysis

For peer review only

Supplementary Table S4 Comparisons of probit and logit probability model estimates

	Probit model: Probability model for appointment booked^ (95% CI)	Logit model: Probability model for appointment booked^^ (95% CI)	Probit model: Probability model for appointment used^ (95% CI)	Logit model: Probability model for appointment used^^ (95% CI)
**Day of week**				
**Monday (base category)**				
**Tuesday**	11.45 (8.51, 14.38)	2.16 (1.72, 2.71)	9.60 (7.45, 11.75)	1.63 (1.48, 1.80)
**Wednesday**	11.06 (5.89, 16.24)	2.08 (1.44, 3.01)	8.45 (3.32, 13.58)	1.53 (1.18, 1.98)
**Thursday**	12.58 (10.05 ,15.10)	2.38 (2.02, 2.80)	7.04 (4.64, 9.44)	1.42 (1.27, 1.58)
**Friday**	10.05 (7.12, 12.98)	1.91 (1.56, 2.34)	4.19 (0.88, 7.51)	1.23 (1.05, 1.43)
**Saturday**	-0.10 (-8.44, 8.24)	0.99 (0.62, 1.57)	-3.47 (-10.97, 4.01)	0.85 (0.61, 1.20)
**Sunday**	-18.93 (-35.03, -2.83)	0.41 (0.20, 0.85)	-18.07 (-32.46, -3.68)	0.46 (0.25, 0.85)
**Calendar month**				
**January (base category)**				
**February**	22.73 (13.25, 32.20)	2.98 (2.26, 3.94)	19.80 (13.15, 26.45)	2.33 (1.85, 2.93)
**March**	20.92 (11.80, 30.04)	2.70 (2.06, 3.55)	17.56 (12.25, 22.88)	2.11 (1.76, 2.52)
**April**	26.84 (11.48, 42.20)	3.91 (1.99, 7.68)	23.42 (11.91, 34.94)	2.78 (1.74, 4.43)
**May**	22.76 (7.90, 37.62)	3.01 (1.65, 5.50)	19.21 (8.32, 30.10)	2.28 (1.41, 3.49)
**June**	22.45 (5.40, 39.50)	2.96 (1.43, 6.13)	20.45 (7.37, 33.52)	2.41 (1.42, 4.08)
**July**	29.08 (16.09, 42.07)	4.43 (2.75, 7.13)	24.13 (13.98, 34.27)	2.86 (1.94, 4.22)
**August**	22.60 (4.12, 41.07)	2.97 (1.33, 6.63)	19.11 (4.95, 33.28)	2.26 (1.27, 4.02)
**September**	30.54 (12.66, 48.43)	4.89 (2.07, 11.57)	25.26 (11.19, 39.32)	3.02 (1.68, 5.47)
**October**	32.26 (15.89, 48.62)	5.60 (2.61, 12.01)	28.11 (15.33, 40.89)	3.51 (2.06, 5.98)
**November**	29.71 (12.79, 46.62)	4.67 (2.15, 10.14)	24.75 (11.59, 37.91)	2.96 (1.72, 5.10)
**December**	29.42 (6.82, 52.01)	4.62 (1.52, 14.08)	23.01 (5.75, 40.26)	2.73 (1.32, 5.64)
**CCG scheme**				
**CCG1***				
**CCG2**	-16.63 (-20.09, -13.17)	0.35 (0.30, 0.40)	-10.43 (-13.17, -7.68)	0.61 (0.54, 0.68)
**CCG3**	-32.02 (-35.24, -28.80)	0.14 (0.13, 0.15)	-23.85 (-26.36, -21.35)	0.32 (0.30, 0.35)
**CCG4**	-11.28 (-14.69, -7.87)	0.46 (0.38, 0.54)	-9.97 (-12.55, -7.38)	0.62 (0.55, 0.69)
**CCG5 (base category)**				
**Sample size**	42,472	42,472	42,472	42,472

Appointments booked are appointments booked, appointments used are appointments that were booked and subsequently attended.
Probability models are probit and logit regressions of appointment status against day of week, calendar month, and CCG scheme.
Standard errors are clustered at the CCG-level.
^Estimates are presented as average marginal effects which give the percentage point effect of the variable relative to the base category.
^^Estimates are presented as odds-ratios which give the odds relative to the base category.
*CCG1 did not provide data to enable identification of whether a booked appointment was subsequently attended so does not feature in the analysis

For peer review only

Supplementary Table S5 Probability models for appointment use with Sunday interaction with calendar month

	Appointment booked (95% CI)	Appointment used (95% CI)
**Day of week (base: Monday)**		
**Tuesday**	0.430 0.320,0.539)	0.288 (0.237,0.339)
**Wednesday**	0.410 (0.232,0.588)	0.249 (0.104,0.394)
**Thursday**	0.481 (0.408,0.553)	0.204 (0.144,0.265)
**Friday**	0.367 (0.268,0.466)	0.119 (0.031,0.207)
**Saturday**	-0.010 (-0.278,0.258)	-0.098 (-0.303,0.108)
**Sunday**	-0.560 (-0.950,-0.170)	-0.659 (-1.127,-0.191)
**Calendar month (base: January)**		
**February**	0.729 (0.580,0.878)	0.562 (0.436,0.687)
**March**	0.563 (0.377,0.748)	0.418 (0.313,0.524)
**April**	0.764 (0.437,1.091)	0.591 (0.377,0.805)
**May**	0.599 (0.298,0.900)	0.451 (0.248,0.655)
**June**	0.684 (0.324,1.044)	0.552 (0.297,0.807)
**July**	0.929 (0.755,1.103)	0.636 (0.488,0.785)
**August**	0.664 (0.266,1.061)	0.493 (0.211,0.775)
**September**	0.938 (0.515,1.362)	0.650 (0.373,0.928)
**October**	0.986 (0.604,1.367)	0.708 (0.434,0.981)
**November**	0.889 (0.495,1.283)	0.622 (0.341,0.903)
**December**	0.895 (0.311,1.479)	0.578 (0.195,0.961)
**Sunday*Calendar month**		
**February**	-0.407 (-0.685,-0.128)	-0.229 (-0.475,0.017)
**March**	0.238 (-0.036,0.512)	0.334 (0.026,0.642)
**April**	0.213 (-0.502,0.929)	0.260 (-0.486,1.006)
**May**	0.307 (-0.420,1.033)	0.368 (-0.349,1.086)
**June**	-0.205 (-0.874,0.464)	-0.044 (-0.708,0.619)
**July**	-0.197 (-1.042,0.648)	0.107 (-0.670,0.884)
**August**	-0.078 (-0.841,0.686)	0.084 (-0.670,0.838)
**September**	0.013 (-0.693,0.719)	0.214 (-0.573,1.002)
**October**	0.131 (-0.439,0.701)	0.389 (-0.198,0.975)
**November**	0.099 (-0.522,0.721)	0.303 (-0.290,0.895)
**December**	-0.001 (-0.984,0.981)	0.290 (-0.573,1.154)
**CCG scheme* (base: CCG5)**		
**CCG2**	-0.590 (-0.703,-0.477)	-0.298 (-0.373,-0.223)
**CCG3**	-1.134 (-1.214,-1.053)	-0.678 (-0.742,-0.614)
**CCG4**	-0.395 (-0.497,-0.292)	-0.281 (-0.347,-0.214)
**Constant**	0.516 (-0.046,1.077)	0.210 (-0.205,0.625)
**Sample size**	42,472	42,472

Appointments booked are appointments booked, appointments booked and used are appointments that were booked and subsequently attended.

For peer review only

Probit regression of appointment status against day of week, calendar month, Sunday interaction with calendar month, and CCG scheme. Standard errors are clustered at the CCG-level.
Estimates are not presented as average marginal effects due to the inability to identify the marginal effect of an interaction term.
*CCG1 did not provide data to enable identification of whether a booked appointment was subsequently attended so does not feature in the analysis

For peer review only

Supplementary Table S6 Missing appointment data for booked appointments

CCG		Total booked	Missing patient gender	Missing patient age	Missing appointment type	Missing booking type	Missing patient's GP practice	Any missing appointment data
**CCG2**		23,345	73 (0.31%)	246 (1.05%)	0 (0.00%)	0 (0.00%)	1,902 (8.15%)	2,041 (8.74%)
	**Hub 1**	5,220	7 (0.13%)	76 (1.46%)	0 (0.00%)	0 (0.00%)	416 (7.97%)	461 (8.83%)
	**Hub 2**	6,435	37 (0.57%)	66 (1.03%)	0 (0.00%)	0 (0.00%)	504 (7.83%)	545 (8.47%)
	**Hub 3**	5,229	16 (0.31%)	70 (1.34%)	0 (0.00%)	0 (0.00%)	470 (8.99%)	493 (9.43%)
	**Hub 4**	6,461	13 (0.20%)	34 (0.53%)	0 (0.00%)	0 (0.00%)	512 (7.92%)	542 (8.39%)
**CCG4**		4,463	227 (5.09%)	227 (5.09%)	345 (7.73%)	0 (0.00%)	457 (10.24%)	759 (17.01%)
	**Hub 1**	1,878	90 (4.79%)	90 (4.79%)	141 (7.51%)	0 (0.00%)	225 (11.98%)	355 (18.90%)
	**Hub 2**	1,060	60 (5.66%)	60 (5.66%)	83 (7.83%)	0 (0.00%)	104 (9.81%)	176 (16.60%)
	**Hub 3**	1,525	77 (5.05%)	77 (5.05%)	121 (7.93%)	0 (0.00%)	128 (8.39%)	228 (14.95%)
**CCG5**		2,183	11 (0.50%)	51 (2.34%)	0 (0.00%)	3 (0.14%)	0 (0.00%)	58 (2.66%)
	**Hub 1**	1,094	8 (0.73%)	19 (1.74%)	0 (0.00%)	0 (0.00%)	0 (0.00%)	22 (2.01%)
	**Hub 2**	1,089	3 (0.28%)	32 (2.94%)	0 (0.00%)	3 (0.28%)	0 (0.00%)	36 (3.31%)
**CCG3**		2,050	0 (0.00%)	0 (0.00%)	0 (0.00%)	0 (0.00%)	23 (1.12%)	23 (1.12%)
	**Hub 1**	1,806	0 (0.00%)	0 (0.00%)	0 (0.00%)	0 (0.00%)	16 (0.89%)	16 (0.89%)
	**Hub 2**	244	0 (0.00%)	0 (0.00%)	0 (0.00%)	0 (0.00%)	7 (2.87%)	7 (2.87%)
**Total**		32,041	311 (0.97%)	524 (1.64%)	345 (1.08%)	3 (0.01%)	2,382 (7.43%)	2,881 (8.99%)

For peer review only

Supplementary Table S7 Probability model for appointment containing missing data

	Missing appointment data^ (95% CI)
**Day of week (base: Monday)**	
**Tuesday**	0.49 (-1.51, 2.49)
**Wednesday**	1.24 (0.05, 2.42)
**Thursday**	0.22 (-0.40, 0.85)
**Friday**	0.87 (-0.29, 2.02)
**Saturday**	0.74 (-0.08, 1.55)
**Sunday**	1.43 (0.49, 2.37)
**Calendar month (base: January)**	
**February**	-0.54 (-0.93, -0.16)
**March**	-3.11 (-3.99, -2.23)
**April**	-2.53 (-4.06, -0.99)
**May**	-1.41 (-3.13, 0.30)
**June**	-0.87 (-2.64, 0.90)
**July**	-2.73 (-5.85, 0.38)
**August**	-4.11 (-5.17, -3.05)
**September**	-2.30 (-7.49, 2.88)
**October**	-3.10 (-6.15, -0.05)
**November**	-3.18 (-8.20, 1.84)
**December**	-3.40 (-9.87, 3.07)
**CCG scheme (base: CCG5)**	
**CCG2**	-6.76 (-7.12, -6.40)
**CCG3**	-
**CCG4**	-21.90 (-21.98, -21.82)
**Sample size**	29,858

^ CCG3 had 100% data completion (n=2,183)
Probit regression of missing data indicator (gender, age, appointment type, booking type, DNA status, patient's GP practice) against day of week, calendar month, and CCG scheme.
Estimates are presented as average marginal effects which give the percentage point effect (when multiplied by 100) of the variable relative to the base category.

For peer review only

Supplementary Table S8 Booked appointment data: summary statistics

Appointment characteristic	Frequency	Percent	Cumulative percentage
**Day of week**			
**Monday**	3,145	10.79	10.79
**Tuesday**	3,335	11.44	22.22
**Wednesday**	2,970	10.19	32.41
**Thursday**	3,101	10.63	43.04
**Friday**	3,064	10.51	53.55
**Saturday**	10,744	36.84	90.39
**Sunday**	2,801	9.61	100.00
**Calendar month**			
**January**	1,310	4.49	4.49
**February**	2,115	7.25	11.75
**March**	2,522	8.65	20.39
**April**	2,530	8.68	29.07
**May**	2,522	8.65	37.72
**June**	2,240	7.68	45.40
**July**	2,695	9.24	54.64
**August**	2,487	8.53	63.17
**September**	2,568	8.81	71.98
**October**	2,936	10.07	82.05
**November**	2,710	9.29	91.34
**December**	2,525	8.66	100.00
**CCG**			
**CCG2**	21,304	73.06	73.06
**CCG3**	2,027	6.95	80.01
**CCG4**	3,704	12.70	92.71
**CCG5**	2,125	7.29	100.00
**Appointment type**			
**GP**	24,068	82.54	82.54
**Nurse**	5,092	17.46	100.00
**Booking type**			
**Pre-booked**	13,538	46.43	46.43
**Same-day**	15,622	53.57	100.00
**Attended status**			
**DNA**	3,548	12.17	12.17
**Attended**	25,612	87.83	100.00
**Gender**			
**Male**	12,071	41.40	41.40
**Female**	17,089	58.60	100.00
**Age**			
**0-9**	3,233	11.09	11.09
**10-19**	2,939	10.08	21.17
**20-29**	5,299	18.17	39.34
**30-39**	4,832	16.57	55.91
**40-49**	4,542	15.58	71.48
**50-59**	4,133	14.17	85.66
**60-69**	2,530	8.68	94.33
**70-79**	1,169	4.01	98.34
**80-89**	443	1.52	99.86
**90+**	40	0.14	100.00
**Total**	29,160	100.00	

Appointment characteristics for appointments booked and used with complete (non-missing) data

For peer review only

For peer review only

Supplementary Table S9 Booked appointment data: summary statistics by appointment type

Appointment characteristic	Pre-booked	%	Same-day	%	GP	%	Nurse	%	Used	%	DNA	%
**Day of week**												
**Monday**	650	5.70	2,495	15.97	1,893	10.32	476	9.35	2,854	11.16	281	7.92
**Tuesday**	1,380	12.09	1,955	12.51	1,975	10.77	546	10.72	3,001	11.74	332	9.36
**Wednesday**	1,401	12.28	1,569	10.04	1,940	10.58	414	8.13	2,651	10.37	317	8.93
**Thursday**	1,437	12.59	1,664	10.65	1,907	10.40	559	10.98	2,667	10.43	432	12.18
**Friday**	1,449	12.70	1,593	10.20	1,945	10.61	458	8.99	2,606	10.19	455	12.82
**Saturday**	3,862	33.84	4,779	30.59	7,076	38.59	2,189	43.17	9,283	36.30	1,444	40.70
**Sunday**	1,234	10.81	1,567	10.03	1,601	8.73	441	8.66	2,510	9.82	287	8.09
**Calendar month**												
**January**	404	3.54	798	5.11	992	5.41	41	0.81	1,157	4.52	151	4.26
**February**	785	6.88	1,156	7.40	1,643	8.96	66	1.30	1,857	7.26	256	7.22
**March**	1,162	10.18	1,204	7.71	1,905	10.39	57	1.12	2,183	8.54	338	9.53
**April**	1,055	9.24	1,270	8.13	1,569	8.56	478	9.39	2,210	8.64	318	8.96
**May**	986	8.64	1,357	8.69	1,532	8.35	532	10.45	2,192	8.57	323	9.10
**June**	869	7.61	1,202	7.69	1,368	7.46	488	9.58	1,982	7.75	253	7.13
**July**	1,036	9.08	1,427	9.13	1,615	8.81	629	12.35	2,320	9.07	374	10.54
**August**	935	8.19	1,369	8.76	1,454	7.93	610	11.98	2,168	8.48	313	8.82
**September**	1,096	9.60	1,289	8.25	1,482	8.08	628	12.33	2,267	8.87	299	8.43
**October**	1,085	9.51	1,626	10.41	1,623	8.85	685	13.45	2,635	10.30	296	8.34
**November**	999	8.75	1,545	9.89	1,562	8.52	549	10.78	2,409	9.42	298	8.40
**December**	1,001	8.77	1,379	8.83	1,592	8.68	329	6.46	2,192	8.57	329	9.27
**CCG**												
**CCG2**	8,922	78.17	12,382	79.26	17,057	93.02	4,247	83.41	18,640	72.89	2,632	74.18
**CCG3**	519	4.55	1,508	9.65	-	-	-	-	1,828	7.15	197	5.55
**CCG4**	1,972	17.28	1,732	11.09	-	-	-	-	3,297	12.89	404	11.39
**CCG5**	-	-			1,280	6.98	845	16.59	1,807	7.07	315	8.88
**Appointment type**												
**GP**	9,181	80.44	13,607	87.10	-	-	-	-	21,082	82.44	2,960	83.43
**Nurse**	2,232	19.56	2,015	12.90	-	-	-	-	4,490	17.56	588	16.57
**Booking type**												
**Pre-booked**	-	-	-	-	7,970	43.46	3,077	60.43	11,279	44.11	2,244	63.25

For peer review only

**Same-day**	-	-	-	-	10,367	56.54	2,015	39.57	14,293	55.89	1,304	36.75
**Attended status**												
**DNA**	1,929	16.90	1,304	8.35	2,359	12.86	588	11.55	-	-	-	-
**Used**	9,484	83.10	14,318	91.65	15,978	87.14	4,504	88.45	-	-	-	-
**Gender**												
**Male**	4,775	41.84	6,547	41.91	7,782	42.44	1,803	35.41	10,609	41.49	1,450	40.87
**Female**	6,638	58.16	9,075	58.09	10,555	57.56	3,289	64.59	14,963	58.51	2,098	59.13
**Age**												
**0-9**	831	7.28	2,202	14.10	2,293	12.50	73	1.43	2,825	11.05	408	11.50
**10-19**	1,158	10.15	1,594	10.20	1,961	10.69	302	5.93	2,508	9.81	431	12.15
**20-29**	2,178	19.08	2,731	17.48	3,424	18.67	885	17.38	4,343	16.98	956	26.94
**30-39**	1,962	17.19	2,445	15.65	2,978	16.24	934	18.34	4,093	16.01	739	20.83
**40-49**	1,859	16.29	2,329	14.91	2,733	14.90	965	18.95	4,054	15.85	488	13.75
**50-59**	1,783	15.62	2,041	13.06	2,515	13.72	893	17.54	3,813	14.91	320	9.02
**60-69**	991	8.68	1,382	8.85	1,502	8.19	583	11.45	2,418	9.46	112	3.16
**70-79**	462	4.05	645	4.13	671	3.66	302	5.93	1,106	4.33	63	1.78
**80-89**	177	1.55	228	1.46	239	1.30	141	2.77	412	1.61	31	0.87
**90+**	12	0.11	25	0.16	21	0.11	14	0.27	-	-	-	-
**Total**	11,413	100.00	15,662	100.00	18,337	100.00	5,092	100.00	25,572	100.00	3,548	100.00

Appointment characteristics for appointments booked and used with complete (non-missing) data

For peer review only

Supplementary Table S10 Demographic distributions of extended access appointment users

	Males			Females			All
	Frequency	%	Cumulative %	Frequency	%	Cumulative %	Cumulative %
**Extended access**							
**Age**							
**20-29**	1,446	17.71	17.71	2,897	23.93	23.93	21.43
**30-39**	1,575	19.30	37.01	2,513	20.75	44.68	41.60
**40-49**	1,617	19.81	56.82	2,435	20.11	64.79	61.59
**50-59**	1,690	20.71	77.53	2,123	17.53	82.32	80.40
**60-69**	1,144	14.02	91.55	1,273	10.51	92.83	92.32
**70-79**	481	5.89	97.44	624	5.15	97.98	97.77
**80-89**	197	2.41	99.85	215	1.78	99.76	99.80
**90+**	12	0.15	100.00	28	0.23	100.00	100.00
**Total**	8,162	100.00		12,108	100.00		
**GP Patient Survey users***							
**Age**							
**18-24**	298	8.45	8.45	514	10.53	10.53	9.55
**25-34**	472	13.39	21.83	798	16.35	26.88	24.51
**35-44**	618	17.52	39.36	812	16.64	43.52	41.56
**45-54**	720	20.43	59.79	912	18.69	62.21	61.07
**55-64**	608	17.26	77.04	691	14.15	76.36	76.68
**65-74**	493	14.00	91.04	578	11.85	88.21	89.54
**75-84**	251	7.11	98.15	383	7.85	96.06	97.04
**85+**	65	1.85	100.00	192	3.94	100.00	100.00
**Total**	3,524	100.00		4,881			

*GPPS weighted responses for patients from practices in CCGs 2 to 5, rounded to nearest whole number

For peer review only

The University of Manchester
National Institute for Health Research
Collaboration for Leadership in Applied Health Research and Care (CLAHRC) for Greater Manchester

GM PRIMARY CARE 7 DAY ACCESS EVALUATION: RESEARCH PROTOCOL

Background

In June 2014, the 12 Greater Manchester Clinical Commissioning Groups (CCGs) agreed to the Healthier Together Primary Care Standard that by the end of 2015, "everyone in Greater Manchester who needs medical help will have same-day access to primary care services, supported by diagnostic tests, seven days a week"[1]. At the same time seven day access has been made one of eight early implementation priorities under the Greater Manchester devolution agreement[2].

This protocol describes the proposed evaluation of seven day access to primary care to be conducted on behalf of NHS England (Greater Manchester) by CLAHRC Greater Manchester. CLAHRC GM previously conducted the evaluation of the NHS England Primary Care Demonstrator services, and the proposed evaluation seeks to build on the strengths of the previous evaluation. The proposed mixed methods evaluation will combine a focus on **activity**, **process** and **outcomes** in order to provide a comprehensive assessment of the implementation and impact of the new services. The evaluation will focus primarily on the 7 CCG areas receiving support from NHS England as part of the devolution agreement. The remaining 5 CCG areas have pre-existing funding and support to provide 7 day access via either the Wave 1 Prime Minister's Challenge Fund (PMCF) or Wave 2 General Practice Access Fund (GPAF) (see **Table 1**). The majority of these are already 'live'; therefore while they will not make up part of the formal evaluation, intelligence gathered in the PMCF/GPAF areas can be used to support learning and development in the seven areas where 7-day primary care access is now being introduced.

Table 1: 7-Day Access Areas in Greater Manchester

PMCF/GPAF Areas (not evaluated)	New 7-Day Access Areas (to be evaluated)
Bury	Bolton
Wigan	Heywood, Middleton and Rochdale
North Manchester	Oldham
Central Manchester	Salford
South Manchester	Stockport
	Trafford
	Tameside & Glossop

[1] https://healthiertogethergm.nhs.uk/what-healthier-together/primary-care/
[2] http://www.gmhealthandsocialcaredevo.org.uk/assets/GM-Strategic-Plan-Final.pdf p14

For peer review only

The 7 CCGs submitted their initial plans for the delivery of seven day primary care access to NHS England in mid-2015. The schemes were expected to start in December 2015, although some have indicated a later start date. All areas are adopting a 'hub' approach to providing additional hours, in which the CCG area is split into geographic areas, and the extended access services are provided from a 'hub' location in each area. CCG plans submitted to NHS England promise 43 hubs (including PMCF/GPAF hubs) operational by December 2015 with a further 9 hubs live in 2016 (see figure 1).

Figure 1

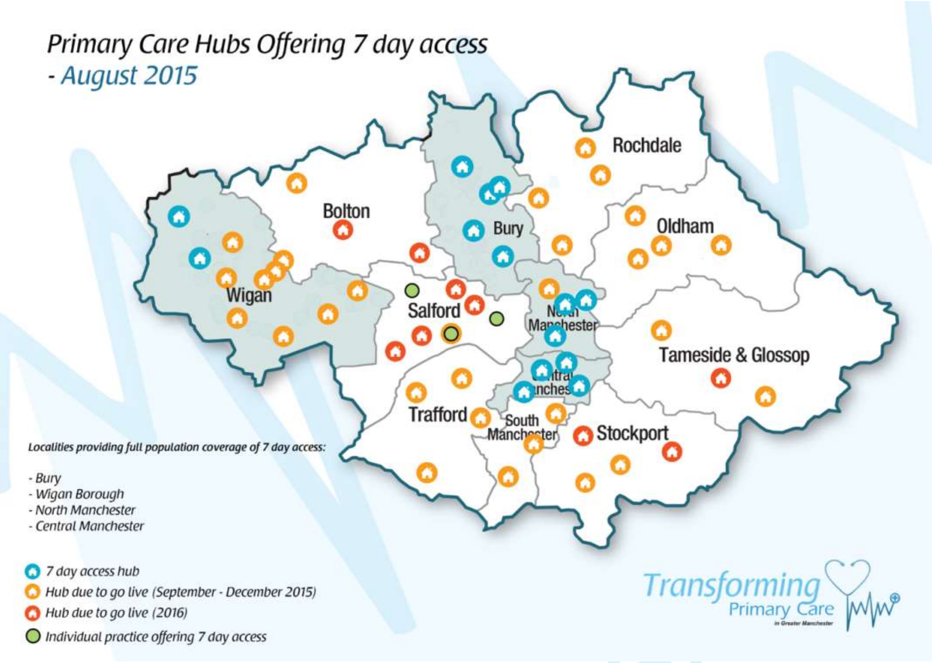

Description of Evaluation

The evaluation seeks to understand the implementation and impact of seven day primary care services across Greater Manchester. The evaluation will combine qualitative and quantitative analysis, supported by a measure of activity across the 7 areas to be evaluated.

The evaluation will seek to answer the following questions (see **Table 2**);

Table 2: Evaluation Questions and Modes of Evaluation

1. What new activity is associated with seven day primary care services in each area, and at what cost?	**ACTIVITY**
2. How are these new services utilised by patients? What is the nature of demand for these new services?	
3. How is the uptake of 7 day primary care affected by the socio-economic characteristics of the patient population?	
4. What organisational and operational issues arise in the implementation of 7 day access to primary care, and how have areas addressed these issues?	**PROCESS**

5. What is the impact of 7 day access to primary care on;<br>a. Identified prevalence of disease,<br>b. Attendances/admissions at Accident & Emergency,<br>c. Use of Out-of-Hours services?	**OUTCOME**
6. How has the change in access been received by<br>a. Patients,<br>b. Staff in primary care?	

As is indicated above, questions 1-3 relate to the **activity** analysis (i.e. what was done). Question 4 relates to the **process** analysis (i.e. how was it done). Questions 5 and 6 relate to the **outcome** analysis (i.e. what was the impact of the new activity).

It should be emphasised that the ability to answer these questions depends directly on the availability of relevant data. It is important that data can be captured by the evaluation team in a timely manner, can be presented in a format which allows for analysis, and that this can be done subject to current information governance regulation. Some data is routinely collected, other data can be collected by the evaluation team with the facilitation of the commissioners and providers in each area, and some can only be produced by the providers themselves (see **Table 3**);

Table 3: Evaluation Questions and Data Sources

1. What new activity is associated with seven day primary care services in each area, and at what cost?	**Activity data supplied by each area**
2. How are these new services utilised by patients? What is the nature of demand for these new services?	**Activity data supplied by each area**
3. How is the uptake of 7 day primary care affected by the socio-economic characteristics of the patient population?	**Public data combined with activity data.**
4. What organisational and operational issues arise in the implementation of 7 day access to primary care, and how have areas addressed these issues?	**Evaluation generated data. Source: qualitative interviews.**
5. What is the impact of 7 day access to primary care on;<br>a. Identified prevalence of disease,<br>b. Attendances/admissions at Accident & Emergency,<br>c. Use of Out-of-Hours services?	**Routinely collected data. Source:**<br>**(a) QOF, via CCGs,**<br>**(b) SUS, via AHSN,**<br>**(c) OOH activity, supplied by NHS England/CCGs.**
6. How has the change in access been received by<br>a. Patients,<br>b. Staff in primary care?	**Evaluation generated data. Source:**<br>**(a) GPPS, via HSCIC.**<br>**(b) staff survey administered by evaluation team via CCGs.**

The evaluation period will begin at the point at which the NHS England hubs go live on 1st January 2016 and continue for 12 months.

For peer review only

The evaluation has conducted preliminary work to establish reliable sources of data, in discussion with NHS England (Greater Manchester) and the Greater Manchester Academic Health Science Network (AHSN). The provision of activity data from each area has not yet been agreed: - this research protocol document is intended to provide information and thereby initiate the process of agreeing the content and process of activity capture in dialogue with each area.

Further details on the data capture and analysis is provided below.

1. Activity Capture

The activity capture in the present evaluation has been designed in order to minimise the additional workload that data extraction places on individual sites, while also seeking to ensure that the data collected can be collected reliably and consistently across all 7 areas.

To this end, a minimum data set (**Table 4** below) has been defined, to be agreed with all participating CCGs. Data should be reported according to a monthly schedule, which will support formative evaluation and shared learning across sites. Also data will be needed from NHS England/CCGs on relevant DES arrangements in each area, to establish activity baselines.

Table 4: Activity Capture Minimum Dataset

**1. Utilisation:**
a. No. of appointments available
i. By day
1. Time of appointment
ii. By discipline
iii. Planned/pre-booked vs same day/urgent
iv. Method of appointment (face-to-face/phone)
b. No. of appointments booked
i. By day
1. Time of appointment
ii. By discipline
iii. Age
iv. Gender
v. Registered GP practice
vi. Postcode
vii. Planned/pre-booked vs same day/urgent
viii. Method of appointment (face-to-face/phone)
c. No. of appointments used (Utilisation)
i. By day
1. Time of appointment
ii. By discipline
iii. Age
iv. Gender
v. Registered GP practice
vi. Postcode (first part)
vii. Planned/pre-booked vs same day/urgent
viii. Method of appointment (face-to-face/phone)

For peer review only

**2. Practice-level population data:**	
a.	Practice level population
b.	Practice codes
c.	Whether a GP practice or not
d.	Registered patient age band
e.	Deprivation of practice population
f.	Gender breakdown of practice population

The aim of the activity capture evaluation is to:

1. **Assess the ability of each area to provide additional appointments i.e.**
   a. **How many additional appointments were available, by whom and by what means?**
   b. **How many additional appointments were booked, urgent, and how many resulted in DNA?**
2. **Assess the types of patients utilizing additional appointments i.e.**
   a. **Were patients attending/DNA representative of the practice's total list (in terms of age, gender, and IMD)?**

For utilisation data (Items 1a-1c, Table 3), the evaluation relies on the ability of each area to collect, collate and provide data routinely. Preliminary conversations with NHS England and some local CCGs suggest that this data should be available – however, this requires confirmation with all areas.[3]

CCG level population data (Item 2, Table 3) can be extracted by the CLAHRC evaluation team from individual practice registration data.

2. Process Evaluation

Semi-structured interviews will be conducted with senior leadership (CCG lead + GP lead in each area). 2 leads x 7 areas= 14 interviews, conducted at start and end of intervention period = 28 interviews. These will be supplemented with key informant interviews (estimated 6-7), including leads in the PMCF/GPAF areas, and, where appropriate, additional interviews with key individuals in each area.

The process evaluation will seek to understand the organisational and operational issues in the implementation of 7 day access to primary care faced in each area, and also how each area addressed these issues. A brief interview schedule can be found in **Appendix 1**.

For peer review only

[3] In addition, the following data would enhance the analysis by providing insight into the reasons for and nature of the additional consultations.

1. Reason for attendance
2. Outcome of consultation
   a. Discharged with advice
   b. Referral to ….. (list providers)
   c. Treatment/prescription issued
   d. Follow-up appointment
   e. Diagnostics
   f. Other

However, we recognise this may be difficult or impossible to provide without incurring substantial and unrealistic data collection costs on the areas.

3. Outcome Evaluation

The outcome evaluation will concentrate on four areas; (A) Health Outcomes, (B) Impact on Service Utilisation, (C) Staff Experience and (D) Patient Experience.

A. **Impact on Health Outcomes** will be primarily measured using selected QOF data on disease prevalence. QOF data on disease prevalence will be analysed to examine the effect of the intervention on the identification and management of long-term conditions. This data will be supplied by local CCGs. Data will be captured for three years prior to the intervention (2013-16) and at two points during the intervention itself. A full list of QOF data categories can be found in **Appendix 2**.

B. **Impact on Service Utilisation** will be measured using routinely-collected SUS data on hospital activity, plus activity data from relevant out-of-hours providers supplied by NHS England/CCGs.

SUS data will be extracted for all areas of Greater Manchester (seven 7 day access sites and 5 PMCF/GPAF areas) on a quarterly basis. The specific items to be analysed can be found in **Appendix 3.**

The aim of the service utilisation evaluation is to:

(1). Assess the effects of additional appointments on hospital activity:

a) A&E attendances:

i) Minor patient-initiated A&E attendances

ii) Total A&E attendances

iii) Waiting time to assessment, treatment, and total A&E duration

b) Admissions:

i) Total admissions

ii) Admissions for ambulatory care-sensitive conditions

(2). Assess whether (1) varies by dosage/volume of additional appointments per 1000

(3). Assess whether (1) varies by additional appointments' users characteristics (age, gender, deprivation)

(1) will be measured using SUS data for the intervention period (quarters 1 to 4 of 2016) compared to 2015 i.e. a before and after analysis. All activity will be measured at the practice level, by quarter, and per 1000 of a practices registered population. A pooled cross-sectional dataset will be used. For each type of hospital activity the dependent variable will be regressed on binary year dummies. The estimated coefficient on the intervention (2016) dummy provide a statistical test for significant differences in the intervention period. Count models will be estimated that recognise and incorporate the over-dispersed distribution of hospital activity. Sensitivity analysis using alternative model specifications will be carried out.

The diagnosis code (DIAG_NN) in the admissions data will be used to identify ambulatory care sensitive conditions as follows:

- Ambulatory care-sensitive conditions admissions (ICD)
- Vaccine-preventable:

  Influenza and pneumonia; Other vaccine-preventable conditions
- Chronic:

  Asthma; Congestive heart failure; Diabetes complications; Chronic obstructive pulmonary disease (COPD); Angina; Iron-deficiency anaemia; Hypertension; Nutritional deficiencies

For peer review only

- Acute:

  Dehydration and gastroenteritis; Pyelonephritis; Perforated/bleeding ulcer; Cellulitis; Pelvic inflammatory disease; Ear, nose and throat infections; Dental conditions; Convulsions and epilepsy; Gangrene

Where available, the difference in hospital activity will be compared against comparator practices and tests for significant differences measured using a *controlled* before and after analysis framework (difference-in-differences).

Items (2) and (3) depend on the quality and coverage of the activity capture. Using data from the activity capture we will generate a measure of intensity of treatment (dosage - additional appointments per 1000 of a practice's registered population) and replicate (1) by dosage. Information on the types of patients using additional appointments from the activity capture will be used to identify the groups where hospital activity may be most concentrated. For example, if additional appointments are concentrated amongst females then it may be that female hospital activity is where the impacts of additional appointments may be seen.

C. **Staff Experience** The evaluation team will develop an online survey to capture information on the experience of primary care staff. This administration of this survey will require the cooperation of local provider organisations.
D. **Patient Experience** We will analyse General Practice Patient Survey (GPPS) data, adopting a difference-in-difference (DiD) methodology, focusing on survey items addressing overall quality of care and convenience, subject to data availability (see note below). Individual CCGs may choose to conduct their own patient surveys, and guidance can be provided by the evaluation team on the administration of these.[4]

[4] Note: While the relevant period of GPPS data will be July-September 2016, which should be released in early 2017. If the release of this data is delayed by HSCIC, to avoid delaying report submission at end of March 2017 then patient experience analysis may have to be supplied latter as a separate addendum to the report.

For peer review only

Evaluation Timeline

	JAN 16	FEB 16	MAR 16	APR 16	MAY 16	JUN 16	JUL 16	AUG 16	SEP 16	OCT 16	NOV 16	DEC 16	JAN 17	FEB 17	MAR 17
Research Approvals	■	■	■	■	■	■									
Activity Capture						■	■	■	■	■	■	■			
Process Evaluation						■	■	■			■	■			
Outcome Evaluation						■			■			■	■	■	
Patient Experience													■	■	
Staff Experience									■	■	■				
Findings (Interim and Final)										■					■

This timeline assumes that;

- The minimum activity dataset can be provided on a monthly basis by CCGs.
- Information on relevant DES activity can be supplied by NHS England/CCGs.
- The evaluation team has access to key individuals in each area for interviews.
- CCGs are able to provide selected QOF data on disease prevalence.
- The AHSN is able to supply regular extracts of SUS data in the appropriate format.
- NHSE is able to supply data on OOH activity in the appropriate format.
- CCGs are able to assist with the dissemination of an online staff survey.
- GP Patient Survey results are released by HSCIC in a timely manner.

For peer review only

Appendix 1

Interview Schedule

1. Describe the primary care access arrangements in your area outside standard hours prior to this initiative, and describe the 7-day access arrangements in place now.
2. What is your role in delivering this change?
3. What is/was required to establish 7-day access to primary care in your locality?
4. What steps have been taken so far?
5. How have you communicated the changes to patients?
6. What challenges have been encountered (IT, IG, communications and engagement, workforce, finance, infrastructure)?
7. How have you tackled these challenges?
8. What do you expect will be the impact of this change in access (on patients, staff and other parts of the health and social care system)?
9. How would you measure 'success' in this change?
10. How sustainable are the changes made in your area?

For peer review only

Appendix 2

AST001. The contractor establishes and maintains a register of patients with asthma, excluding patients with asthma who have been prescribed no asthma-related drugs in the preceding 12 months.

AF001. The contractor establishes and maintains a register of patients with atrial fibrillation.

CAN001. The contractor establishes and maintains a register of all cancer patients defined as a "register of patients with a diagnosis of cancer excluding non-melanotic skin cancers diagnosed on or after 1 April 2003".

CKD001. The contractor establishes and maintains a register of patients aged 18 or over with CKD (US National Kidney Foundation: Stage 3 to 5 CKD).

COPD001. The contractor establishes and maintains a register of patients with COPD.

DEM001. The contractor establishes and maintains a register of patients diagnosed with dementia.

DEP003. The percentage of patients aged 18 or over with a new diagnosis of depression in the preceding 1 April to 31 March, who have been reviewed not earlier than 10 days after and not later than 56 days after the date of diagnosis.

DM017. The contractor establishes and maintains a register of all patients aged 17 or over with diabetes mellitus, which specifies the type of diabetes where a diagnosis has been confirmed.

EP001. The contractor establishes and maintains a register of patients aged 18 or over receiving drug treatment for epilepsy.

HF001. The contractor establishes and maintains a register of patients with heart failure.

HYP001. The contractor establishes and maintains a register of patients with established hypertension.

LD003. The contractor establishes and maintains a register of patients with learning disabilities.

MH001. The contractor establishes and maintains a register of patients with schizophrenia, bipolar affective disorder and other psychoses and other patients on lithium therapy.

OST004. The contractor establishes and maintains a register of patients: 1. Aged 50 or over and who have not attained the age of 75 with a record of a fragility fracture on or after 1 April 2012 and a diagnosis of osteoporosis confirmed on DXA scan, and 2. Aged 75 or over with a record of a fragility fracture on or after 1 April 2014 and a diagnosis of osteoporosis.

PC001. The contractor establishes and maintains a register of all patients in need of palliative care/support irrespective of age.

PAD001. The contractor establishes and maintains a register of patients with peripheral arterial disease.

RA001. The contractor establishes and maintains a register of patients aged 16 or over with rheumatoid arthritis.

CHD001. The contractor establishes and maintains a register of patients with coronary heart disease.

STIA001. The contractor establishes and maintains a register of patients with stroke or TIA.

For peer review only

Appendix 3

Relevant SUS data to be analysed

Derived variable	SUS variable name	SUS variable code
**A&E DATA**		
**Key base data**		
**A&E department type**	A&E department type	AEDEPTTYPE
**Provider code**	Provider code	PROCODET
**Arrival date**	A&E arrival date	ARRIVALDATE
**Arrival time**	A&E arrival time	ARRIVALTIME
**Arrival mode**	Arrival mode	AEARRIVALMODE
**Where incident took place**	Incident location type	AEINCLOCTYPE
**GP practice**	Code of GP practice	GPPRAC
**CCG of GP practice**	CCG of patient's GP practice	CCG_GP_PRACTICE
**CCG of Treatment**	CCG of Treatment	CCG_TREATMENT
**Self-referrals for minor conditions**	Referral mode,<br>SUS PbR derived healthcare resource group code	AEREFSOURCE<br>SUSHRG
**Cost**	Trust derived HRG value	HRGNHS
**GP referrals**	Referral mode	AEREFSOURCE
**Arrival age**	Age on arrival	ARRIVALAGE
**Sex**	Sex of patient	SEX
**Ethnicity**	Ethnic category	ETHNOS
**Deprivation**	Lower Super Output Area (to obtain IMD)	LSOA11
**Diagnosis**	A&E diagnosis	DIAG_NN
**Effect on admissions/workload**		
**Admissions from A&E**	Attendance disposal	AEATTENDISP
**A&E Waiting times**		
**A&E time to assessment (waiting time)**	A&E Duration to assessment	INITDUR
**A&E time to treatment (waiting time)**	A&E Duration to treatment	TRETDUR
**A&E total duration**	A&E duration to departure	DEPDUR
**ADMISSIONS DATA**		
**Key base data**		
**Provider code**	Provider code	PROCODET
**Arrival date**	Date of admission	ADMIDATE
**Discharge date**	Date of discharge	DISDATE
**GP practice**	Code of GP practice	GPPRAC
**CCG of GP practice**	CCG of patient's GP practice	CCG_GP_PRACTICE
**CCG of Treatment**	CCG of Treatment	CCG_TREATMENT
**Cost**	Trust derived HRG value	HRGNHS
**Arrival age**	Age on arrival	ADMIAGE
**Sex**	Sex of patient	SEX
**Ethnicity**	Ethnic category	ETHNOS
**Deprivation**	Lower Super Output Area (to obtain IMD)	LSOA11
**Ambulatory care-sensitive conditions admissions (ICD)**	All Diagnosis codes	DIAG_NN

For peer review only - http://bmjopen.bmj.com/site/about/guidelines.xhtml

Supplementary Text S2

(i) Uptake

Patients could book an appointment which could then be attended or not. Focussing on booked appointments alone would give an indication of the demand for the service, whilst assessments of appointments actually used shed light on realised demand and is more insightful for evaluating actual impacts on service use. We estimated two separate sets of probability models:

1) the probability an appointment was booked, and
2) the probability an appointment was booked and attended ('used')

We assessed differences in the rate of uptake by estimating probability models (probit regressions) of appointment use (y_{ij}):

$$y_{ij} = \beta_1 D_{ij} + \beta_2 M_{ij} + \beta_3 CCG_j + e_{ij} \quad [1]$$

where the unit of analysis is available appointment i in CCG j.

Regression models were estimated to enable adjustment for CCG effects which may be important given the variation in schemes across CCGs (Supplementary Table 1), Probit models were used to estimate differences in the probability (rate) of uptake across categories of the predictor variables. The estimates give absolute percentage point differences across predictor variables which is more informative than relative odds-ratios for understanding the scale of uptake.

To assess whether uptake of the service varied across day of the week we included day of week dummies in the regression (D_i), with the estimates ($\hat{\beta}_1$) providing the differences in the rate of uptake relative to the base week day (Monday); this enables a test for whether uptake is lower on particular days. To assess whether there is evidence of assimilation to the service we include calendar month dummies (M_i). If assimilation is evident the estimates on the calendar month dummies should be monotonically increasing over the period. To account for potential confounding caused by variations in the provision and delivery of services across schemes we include CCG dummies (fixed-effects) in the regression (CCG_j). Standard errors are clustered at CCG level.

Estimates are presented as average marginal effects (using Stata's margins command), which give the average percentage point effect of the variable relative to the base category across the sample.

(ii) Analysis of demographics of patients booking an extended access appointment

For peer review only

Our second set of analyses concentrated on booked appointments and exploited variations in service delivery to test whether there were associations in the demand for booking particular types of services (booking type (pre-booked or same day) and appointment type (GP or nurse)) and for whether an appointment was used or DNA; with day of week, calendar month, the patient booking the appointments age and gender, and CCG. We estimated three separate sets of probability models:

1) The probability an appointment was pre-booked versus same day;
2) The probability an appointment was for a GP versus for a nurse practitioner;
3) The probability an appointment was used or not attended (DNA)

We assessed differences in the rate of appointment type by estimating probability models (probit regressions) of appointment type (y_{ij}):

$$y_{ij} = \beta_1 D_{ij} + \beta_2 M_{ij} + \beta_3 DNA_{ij} + \beta_4 Female_{ij} + \beta_5 Age_{ij} + \beta_6 CCG_j + e_{ij} \qquad [2]$$

where the unit of analysis is the type of booked appointment i in CCG j. $Female_{ij}$ is a dummy variable indicating the gender of the patient booked for appointment i in CCG j, and Age_{ij} is a set of 10 age-band dummies indicating the age of the patient. For all models estimated, estimates are presented as average marginal effects and standard errors are clustered at GP practice level where the practice is the patient who has booked the appointments registered practice. Schemes that exclusively provided a certain service (e.g. only GP appointments) are excluded from the relevant analysis since uptake of the alternative is necessarily zero.

For peer review only

Supplementary Text S3

CLAHRC Patient Panel Protocol Review
Collated Feedback and Responses

**Feedback from panel**	**Project team response**
The interview document [referring to the interview schedule] seems ok.	No response required.
The survey document I would change slightly as per the attachment:<br><br>5. Which of the following do you feel (may have-deleted) has benefited from the implementation of seven day access to primary care?<br><br>6. Remove mentions of 'efforts to' from the response options e.g. efforts to reduce demand on A&E services.	
This is a very comprehensive, well thought out document. It is a very comprehensive protocol indeed.<br><br>Appointments must be booked in advance, but what if changes take place on the part of either the patient or the GP hub nearer the time of the appointment?<br><br>Evidence of use and pattern of use at weekends is crucial! Evenings will prove popular, Saturday mornings will indicate some demand but I am unsure about the demand on Saturday afternoons and especially Sundays. I am referring to planned appointments and not Emergency appointments!	No response required.<br><br>No change to protocol as this situation is not specific to the additional availability, as appointments are also booked in advance within core services.<br><br>Agree that utilisation by day of week and time of day is important. This will be monitored via the activity data.
The 'don't know' response option should be removed, in all cases, from the staff survey.	No change to staff survey, as 'don't know' response is required to ensure that those who select an alternative option do so as there is an element of certainty about their choice. Important for data quality.
The following questions are suggested to be added to the interview schedule:<br><br>a. How effective has the 7 day access scheme been in terms of number of patients visiting during extended hours?<br>b. Describe the patterns of demand during the extended hours and the average cost per 10 minute slot?	No change to interview schedule, as captured via the activity data.<br><br>No change to interview schedule, as captured via the activity data.

c. Indicate staff morale during the extended hours?	No change to interview schedule, as captured via the staff survey.

For peer review only

STROBE Statement—Checklist of items that should be included in reports of ***cohort studies***

	Item No	Recommendation	Location in manuscript
**Title and abstract**	1	(*a*) Indicate the study's design with a commonly used term in the title or the abstract	Title
		(*b*) Provide in the abstract an informative and balanced summary of what was done and what was found	Abstract
**Introduction**			
Background/rationale	2	Explain the scientific background and rationale for the investigation being reported	Introduction: Paragraphs 1, 2 & 3
Objectives	3	State specific objectives, including any prespecified hypotheses	Introduction: Paragraph 4
**Methods**			
Study design	4	Present key elements of study design early in the paper	Introduction: Paragraph 2<br>Methods: Paragraphs 1, 2, 3, & 4
Setting	5	Describe the setting, locations, and relevant dates, including periods of recruitment, exposure, follow-up, and data collection	Methods: Paragraphs 1, 2, 3, & 12<br>Figure 1<br>Supplementary Table S1
Participants	6	(*a*) Give the eligibility criteria, and the sources and methods of selection of participants. Describe methods of follow-up	Methods: Paragraphs 1, 2, 3, & 12<br>Figure 1
		(*b*) For matched studies, give matching criteria and number of exposed and unexposed	N/A
Variables	7	Clearly define all outcomes, exposures, predictors, potential confounders, and effect modifiers. Give diagnostic criteria, if applicable	Methods: Paragraphs 5, 6, 7, 8, 9, 10, 11 & 12
Data sources/ measurement	8*	For each variable of interest, give sources of data and details of methods of assessment (measurement). Describe comparability of assessment methods if there is more than one group	Methods: Paragraphs 1, 2, 3, & 12<br>Supplementary Table S2
Bias	9	Describe any efforts to address potential sources of bias	Methods: Paragraphs 7, 10, 11, & 12
Study size	10	Explain how the study size was arrived at	Methods: Paragraphs 1, 3 & 12<br>Results: Paragraph 1 & 5<br>Figure 1
Quantitative variables	11	Explain how quantitative variables were handled in the analyses. If applicable, describe which groupings were chosen and why	Methods: Paragraphs 6, 7, 8, 9, 10, 11, & 12<br>Supplementary Table S2
Statistical methods	12	(*a*) Describe all statistical methods, including those used to control for confounding	Methods: Paragraphs 6, 7, 8, 9, 10, 11, & 12

For peer review only

			Supplementary Text S2
		(*b*) Describe any methods used to examine subgroups and interactions	Results: Paragraph 4
		(*c*) Explain how missing data were addressed	Methods: Paragraph 11 Results: Paragraph 5 Supplementary Tables S6 & S7
		(*d*) If applicable, explain how loss to follow-up was addressed	N/A
		(*e*) Describe any sensitivity analyses	Results: Paragraph 3
**Results**			
Participants	13*	(a) Report numbers of individuals at each stage of study—eg numbers potentially eligible, examined for eligibility, confirmed eligible, included in the study, completing follow-up, and analysed	Results: Paragraph 1, 5, & 9 Tables 1, 2 & 5 Figure 1
		(b) Give reasons for non-participation at each stage	Results: Paragraphs 1 & 5 Tables 1, 2, 3 & 4 Footnotes
		(c) Consider use of a flow diagram	Figure 1
Descriptive data	14*	(a) Give characteristics of study participants (eg demographic, clinical, social) and information on exposures and potential confounders	Results: Paragraphs 6, 7, 8, 9, & 10 Supplementary Tables S8, S9 & S10
		(b) Indicate number of participants with missing data for each variable of interest	Results: Paragraph 5 Supplementary Table S6
		(c) Summarise follow-up time (eg, average and total amount)	N/A
Outcome data	15*	Report numbers of outcome events or summary measures over time	Results: Paragraph 3 Figure 2 & 3 Supplementary Table S3
Main results	16	(*a*) Give unadjusted estimates and, if applicable, confounder-adjusted estimates and their precision (eg, 95% confidence interval). Make clear which confounders were adjusted for and why they were included	Results: Paragraphs 1, 2, 3, 4, 5, 6, 7, 8, & 9 Tables 1, 2, 3, 4 & 5 Supplementary Tables S3 to S10
		(*b*) Report category boundaries when continuous variables were categorized	N/A
		(*c*) If relevant, consider translating estimates of relative risk into absolute risk for a meaningful time period	Methods: Paragraph 7 Supplementary Text S2

For peer review only - http://bmjopen.bmj.com/site/about/guidelines.xhtml

Other analyses	17	Report other analyses done—eg analyses of subgroups and interactions, and sensitivity analyses	Results: Paragraph 4
**Discussion**			
Key results	18	Summarise key results with reference to study objectives	Discussion: Paragraph 1
Limitations	19	Discuss limitations of the study, taking into account sources of potential bias or imprecision. Discuss both direction and magnitude of any potential bias	Discussion: Paragraph 2
Interpretation	20	Give a cautious overall interpretation of results considering objectives, limitations, multiplicity of analyses, results from similar studies, and other relevant evidence	Discussion: Paragraphs 3, 4, 5, & 6
Generalisability	21	Discuss the generalisability (external validity) of the study results	Discussion: Paragraph 2
**Other information**			
Funding	22	Give the source of funding and the role of the funders for the present study and, if applicable, for the original study on which the present article is based	Funding statement

*Give information separately for exposed and unexposed groups.

Note: An Explanation and Elaboration article discusses each checklist item and gives methodological background and published examples of transparent reporting. The STROBE checklist is best used in conjunction with this article (freely available on the Web sites of PLoS Medicine at http://www.plosmedicine.org/, Annals of Internal Medicine at http://www.annals.org/, and Epidemiology at http://www.epidem.com/). Information on the STROBE Initiative is available at http://www.strobe-statement.org.

For peer review only
